# Supplementary figures and images for: Reconstructed influenza A/H3N2 infection histories reveal variation in incidence and antibody dynamics over the life course
Source: medRxiv. 2024 Apr 5:2024.03.18.24304371. Originally published 2024 Mar 18. Preprint. [Version 2] doi: 10.1101/2024.03.18.24304371 (PMC10984066; doi:10.1101/2024.03.18.24304371)

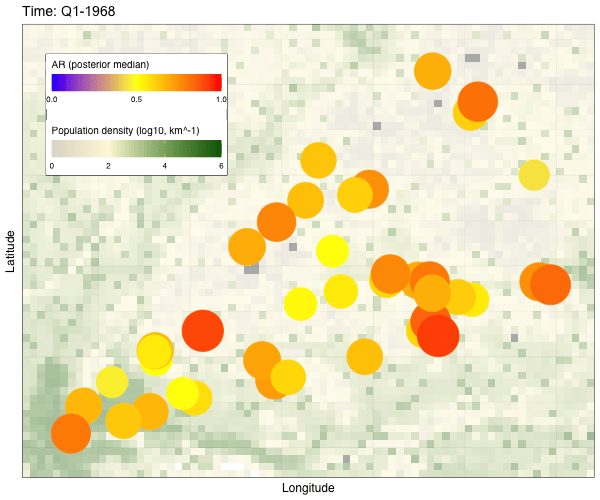

Supplement: Supplement 1 [file media-1.gif]
