# Supplementary material for: Reconstructed influenza A/H3N2 infection histories reveal variation in incidence and antibody dynamics over the life course
Source: medRxiv. 2024 Apr 5:2024.03.18.24304371. Originally published 2024 Mar 18. Preprint. [Version 2] doi: 10.1101/2024.03.18.24304371 (PMC10984066; doi:10.1101/2024.03.18.24304371)
Supplement: Supplement 2 [file NIHPP2024.03.18.24304371v2-supplement-2.pdf]

medRxiv preprint doi: https://doi.org/10.1101/2024.03.18.24304371; this version posted April 5, 2024. The copyright holder for this preprint (which was not certified by peer review) is the author/funder, who has granted medRxiv a license to display the preprint in perpetuity.
It is made available under a CC-BY 4.0 International license .

# Supplementary text, figures and tables

medRxiv preprint doi: https://doi.org/10.1101/2024.03.18.24304371; this version posted April 5, 2024. The copyright holder for this preprint (which was not certified by peer review) is the author/funder, who has granted medRxiv a license to display the preprint in perpetuity.
It is made available under a CC-BY 4.0 International license .

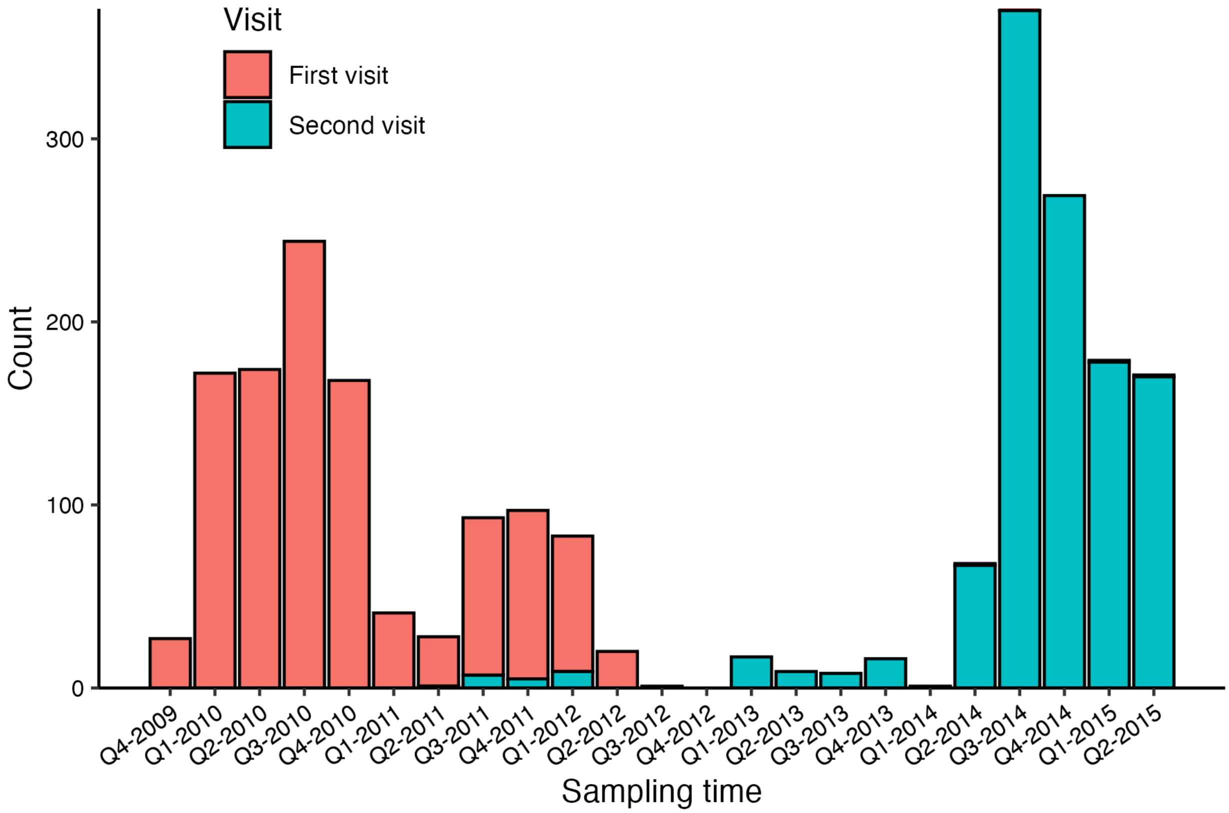


**Figure S1: Distribution of serum sampling times from the Fluscape cohort.** First and second visits refer to an individual's serum sample order, which may differ from the sample collection round of the overall study.

medRxiv preprint doi: https://doi.org/10.1101/2024.03.18.24304371; this version posted April 5, 2024. The copyright holder for this preprint (which was not certified by peer review) is the author/funder, who has granted medRxiv a license to display the preprint in perpetuity.
It is made available under a CC-BY 4.0 International license .

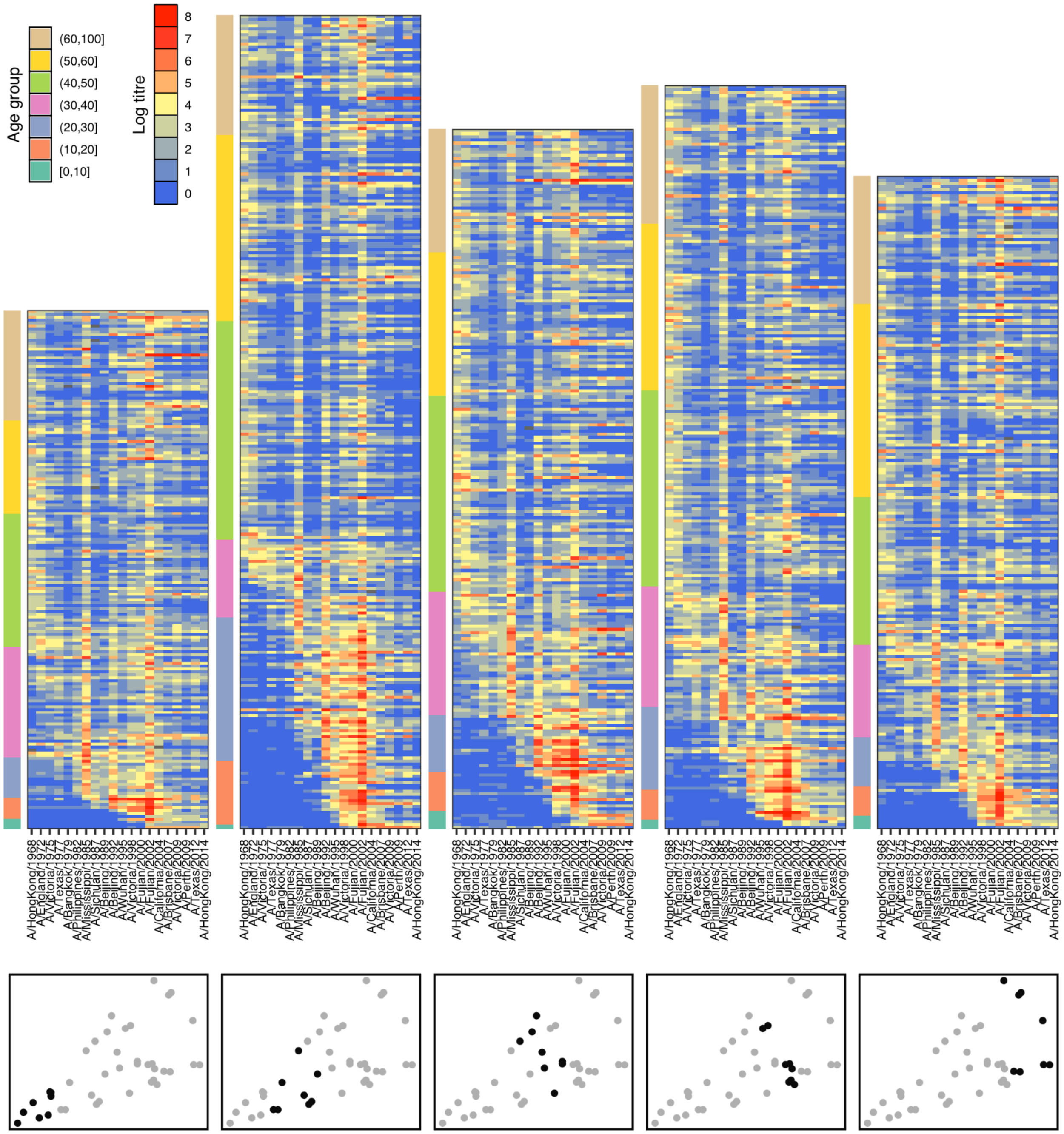


**Figure S2: Distribution of log HI titres by study location at first serum sample.** Each cell represents the log HI titre for one individual measured against one strain, shown on the x-axis. Locations were grouped into quintiles based on increasing distance from Guangzhou city center (bottom panels). Individuals were grouped by age and plotted with increasing age. Colors to the left of each subplot show age group.

medRxiv preprint doi: https://doi.org/10.1101/2024.03.18.24304371; this version posted April 5, 2024. The copyright holder for this preprint (which was not certified by peer review) is the author/funder, who has granted medRxiv a license to display the preprint in perpetuity.
It is made available under a CC-BY 4.0 International license .

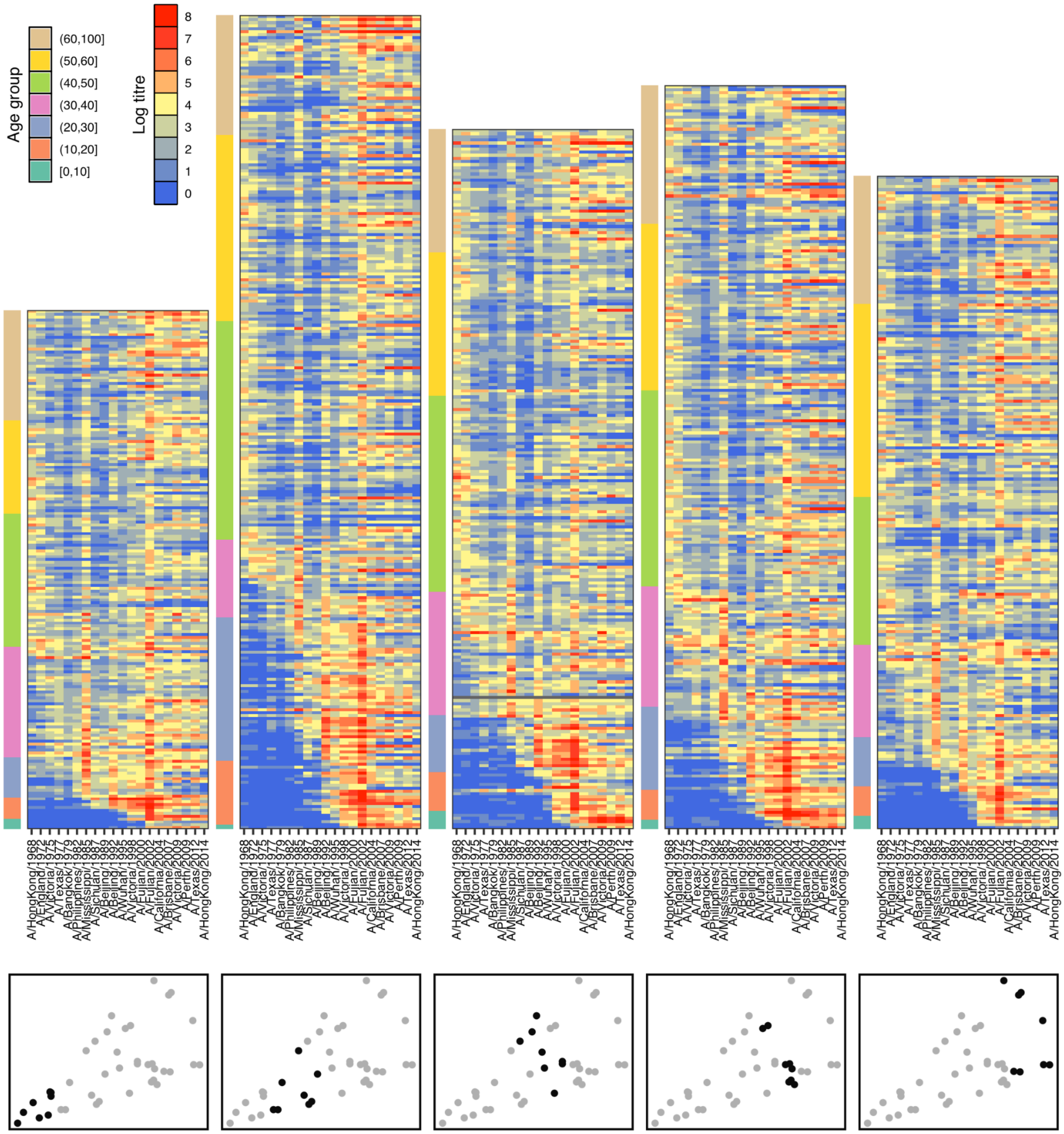


**Figure S3: Distribution of log HI titres by study location at second serum sample.** Each cell represents the log HI titre for one individual measured against one strain, shown on the x-axis. Locations were grouped into quintiles based on increasing distance from Guangzhou city center (bottom panels). Individuals were grouped by age and plotted with increasing age. Colors to the left of each subplot show age group.

medRxiv preprint doi: https://doi.org/10.1101/2024.03.18.24304371; this version posted April 5, 2024. The copyright holder for this preprint (which was not certified by peer review) is the author/funder, who has granted medRxiv a license to display the preprint in perpetuity.
It is made available under a CC-BY 4.0 International license .

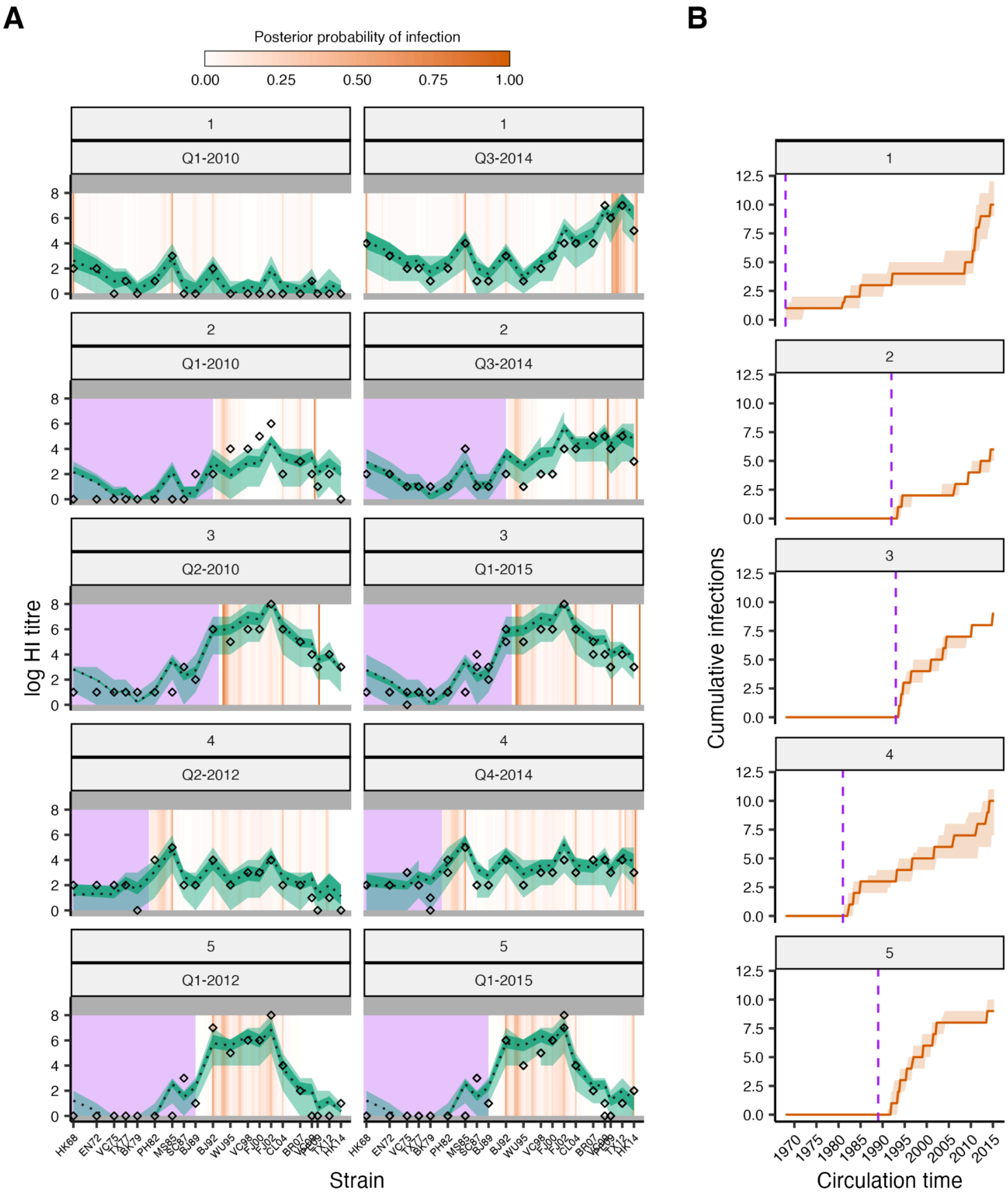


**Figure S4: Example inferred latent antibody titres and infection histories.** (**A**) Model-predicted titres compared to observed HI titres at each sampling time for 5 randomly selected individuals. Rows represent individuals. Subplots show antibody titres based on serum samples taken at that time. X-axis represents a position along the antigenic summary path. Black diamonds show observed titres. Black line and green shaded regions show posterior median and 95% credible intervals (CrI) on model-predicted latent titres (dark green) and 95% prediction intervals (light green). Orange bars show posterior probability of infection in that 3-month window. Gray rectangles denote the limit of detection of the HI assay. Purple rectangles show time periods before birth. (**B**) Posterior median and 95% CrI for the cumulative number of infections over time from birth (purple dashed line).

medRxiv preprint doi: https://doi.org/10.1101/2024.03.18.24304371; this version posted April 5, 2024. The copyright holder for this preprint (which was not certified by peer review) is the author/funder, who has granted medRxiv a license to display the preprint in perpetuity.
It is made available under a CC-BY 4.0 International license .

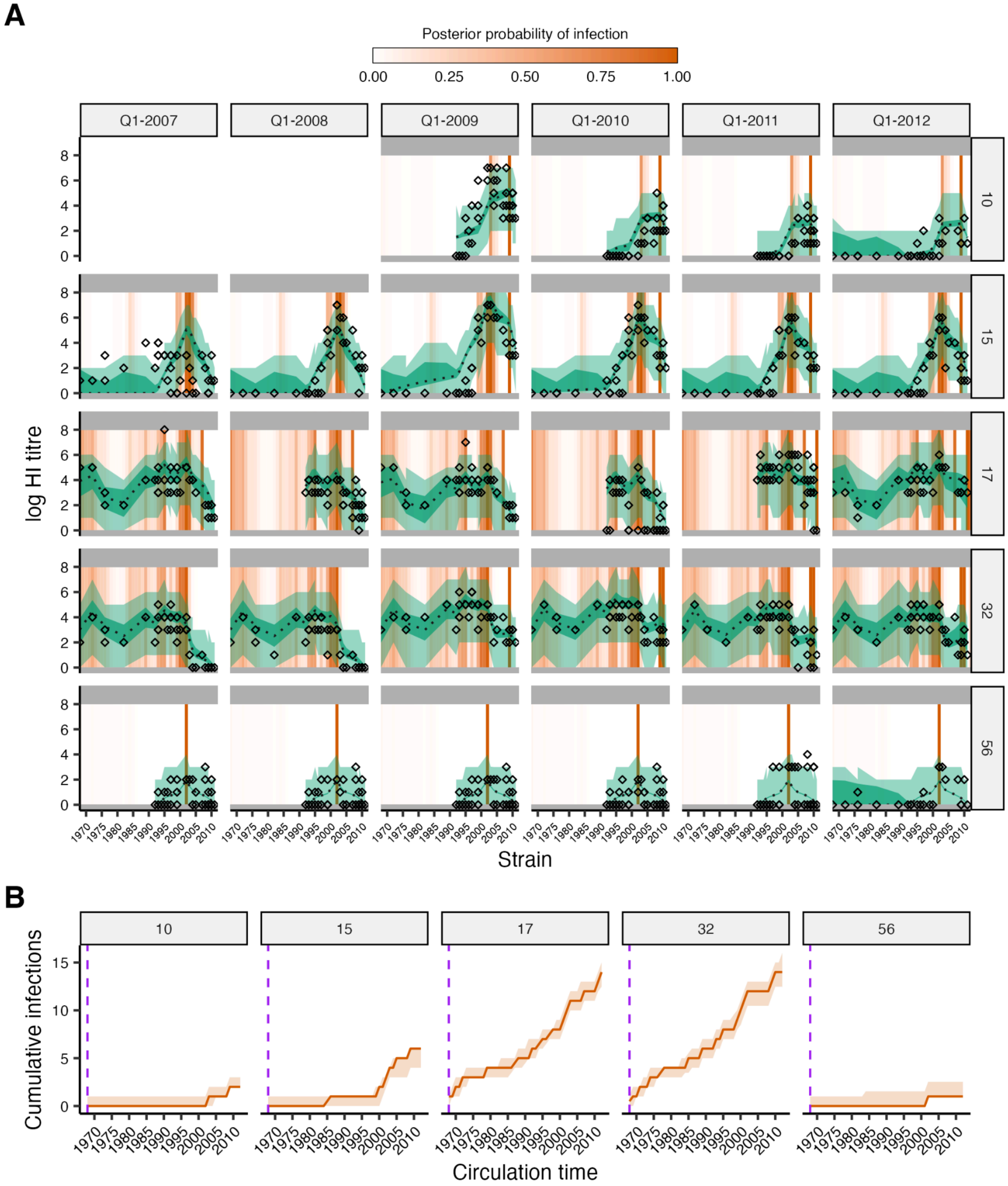


**Figure S5: Model fit to data from Ha Nam, Viet Nam.** (**A**) Model-predicted titres compared to observed HI titres at each sampling time for five randomly selected individuals, as in Fig S4. Diamonds show titre measurements; green shaded region shows 95% CrI and 95% prediction intervals; dashed line shows posterior median; orange bars show posterior probability of infection in a given time window. (**B**) Posterior median and 95% credible intervals (CrI) for the cumulative number of infections over time from birth (orange). Note that date of birth information was not available for these individuals.

medRxiv preprint doi: https://doi.org/10.1101/2024.03.18.24304371; this version posted April 5, 2024. The copyright holder for this preprint (which was not certified by peer review) is the author/funder, who has granted medRxiv a license to display the preprint in perpetuity.
It is made available under a CC-BY 4.0 International license .

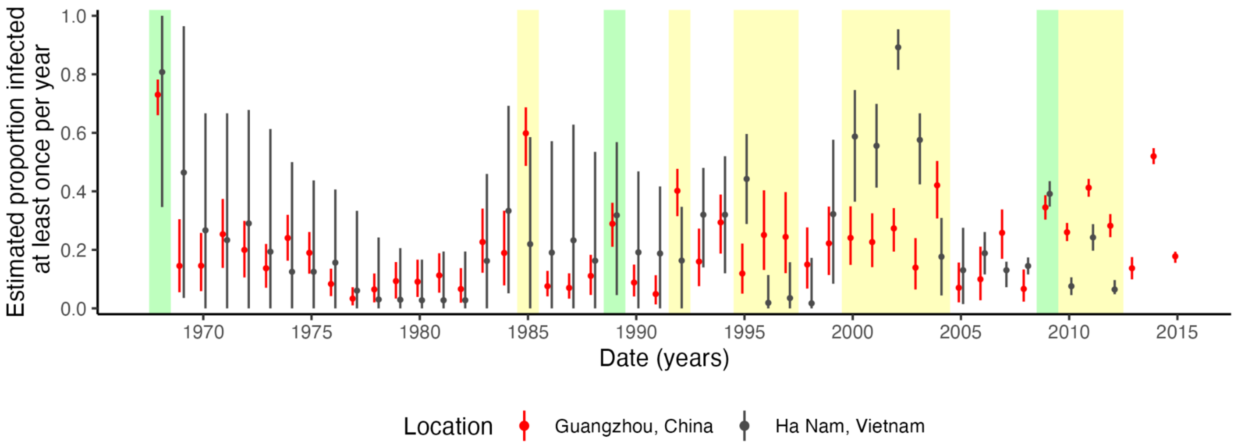


**Figure S6: Comparison of annual attack rates using data from Ha Nam, Viet Nam and the Fluscape study in Guangzhou, China.** Annual attack rates were defined here as the proportion of individuals who experienced at least one infection per year. Green shaded regions show notable time periods where the two datasets return similar attack rate estimates, whereas yellow shaded regions show time periods with notably different attack rate estimates. Attack rates in 1968, 1989 and 2009 were remarkably similar between the two locations. Some time periods showed high uncertainty for the Ha Nam dataset, as few individuals in the sample were alive during that time (e.g., 1969-1980). Attack rate estimates were very different from 2000 to 2004, with much higher attack rates estimated for the Ha Nam cohort. This might reflect a genuine different in A/H3N2 epidemiology during that time, but may also be partially driven by systematic biases in titre measurements to strains isolated during that time period – the fits to the Fluscape data include a positive offset term for titres against A/Fujian/2002, which leads to lower attack rate estimates in that time period, whereas fits to the Ha Nam data do not. The time period from 2010-2012 is also interesting, showing similar relative patterns but much higher attack rate estimates overall in the Fluscape cohort.

medRxiv preprint doi: https://doi.org/10.1101/2024.03.18.24304371; this version posted April 5, 2024. The copyright holder for this preprint (which was not certified by peer review) is the author/funder, who has granted medRxiv a license to display the preprint in perpetuity.
It is made available under a CC-BY 4.0 International license .

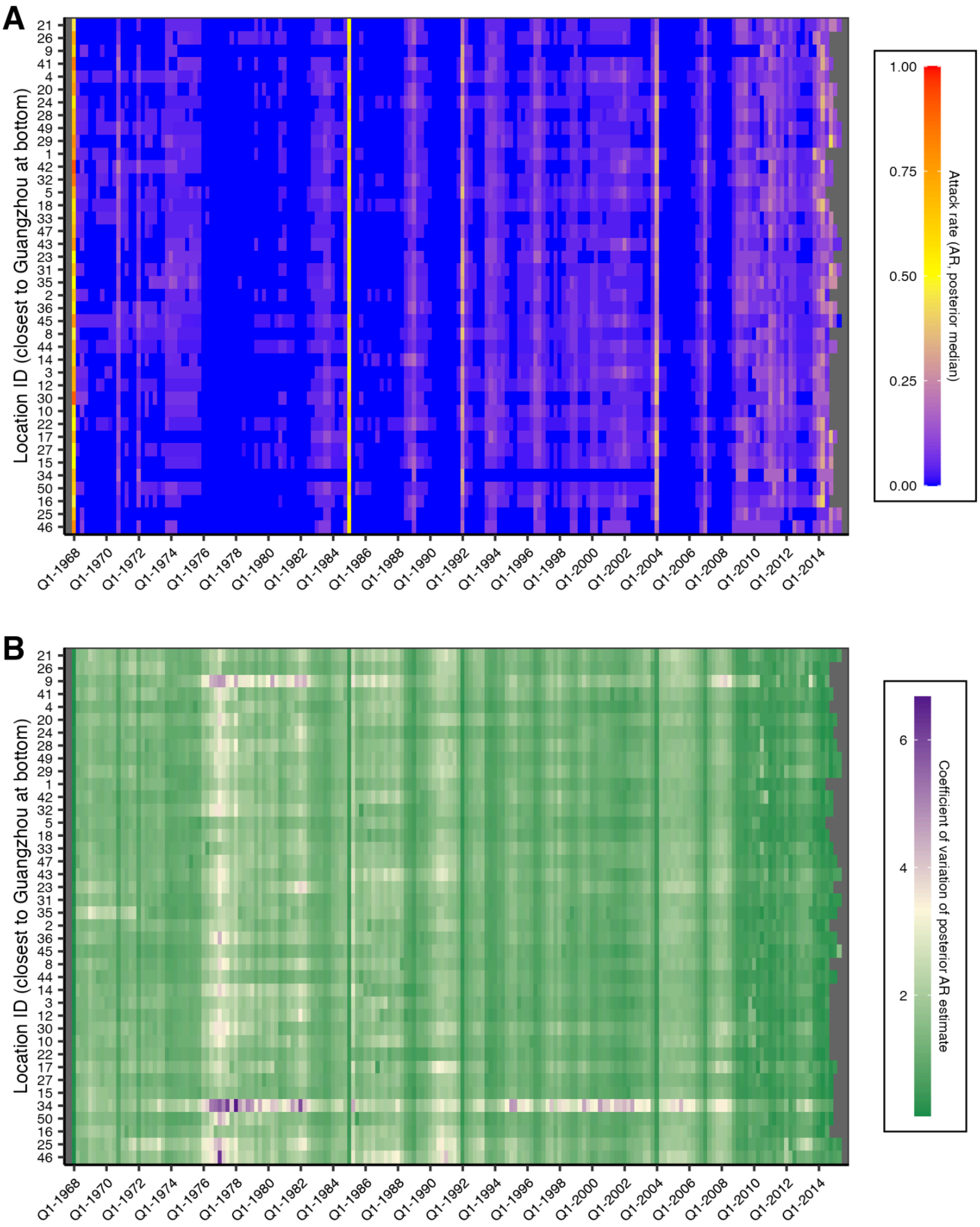


**Figure S7: Quarterly attack rates across the 40 Fluscape study locations.** Each row represents one study location ordered by increasing distance from Guangzhou city center. Each column represents a 3 month period. Cells are shaded by (**A**) the posterior median inferred attack rate or (**B**) the coefficient of variation of the posterior quarterly attack rate estimate for each location.

medRxiv preprint doi: https://doi.org/10.1101/2024.03.18.24304371; this version posted April 5, 2024. The copyright holder for this preprint (which was not certified by peer review) is the author/funder, who has granted medRxiv a license to display the preprint in perpetuity.
It is made available under a CC-BY 4.0 International license .

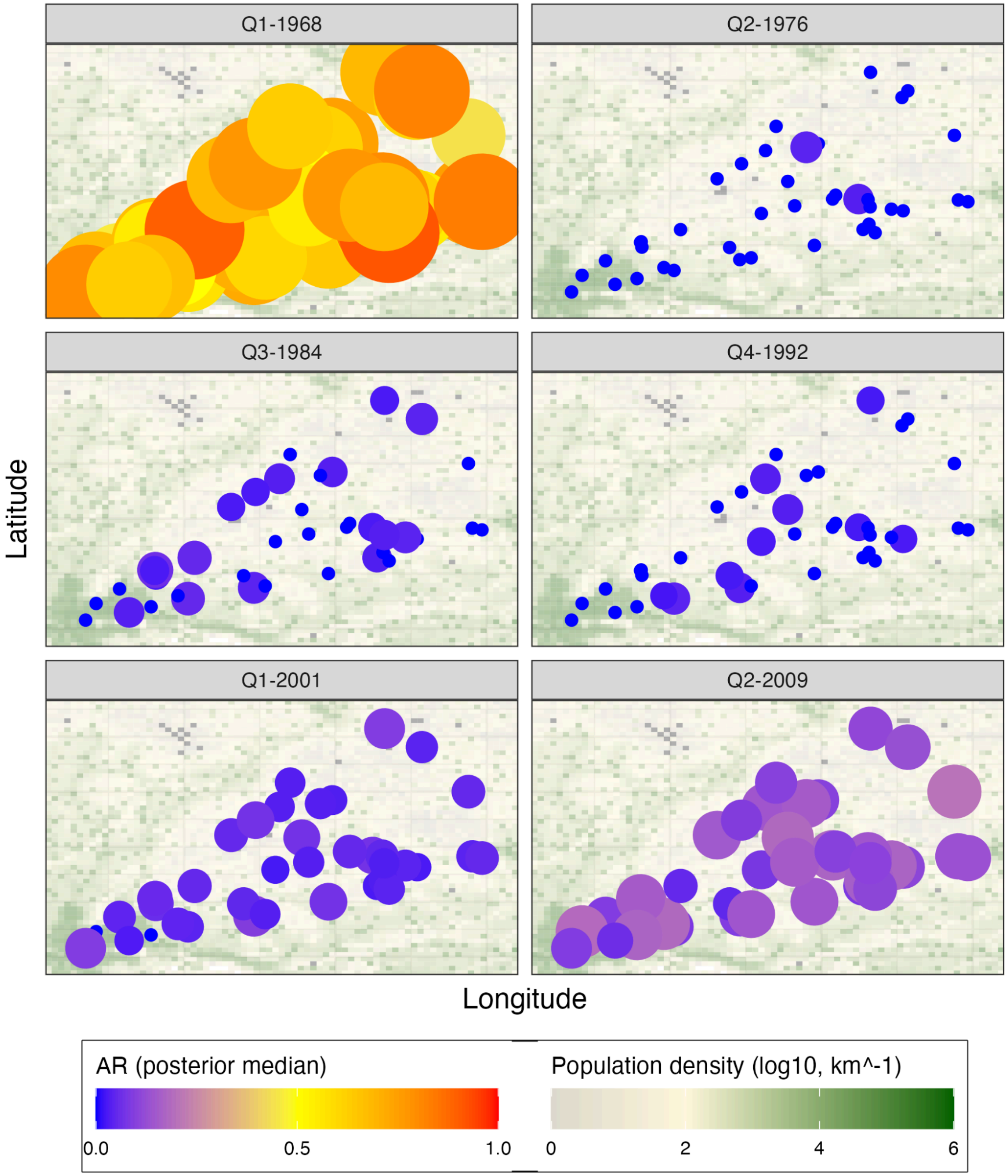


**Figure S8: Distribution of quarterly attack rates by location over time.** Each panel is one frame from a full animation available in Supplementary Material 3. Each colored point shows the inferred attack rate in each of the 40 locations, with size and shading reflecting the posterior median attack rate. Underlying the plot is a map of the study area, with each grid cell shaded by its $log_{10}$ population density.

medRxiv preprint doi: https://doi.org/10.1101/2024.03.18.24304371; this version posted April 5, 2024. The copyright holder for this preprint (which was not certified by peer review) is the author/funder, who has granted medRxiv a license to display the preprint in perpetuity.
It is made available under a CC-BY 4.0 International license .

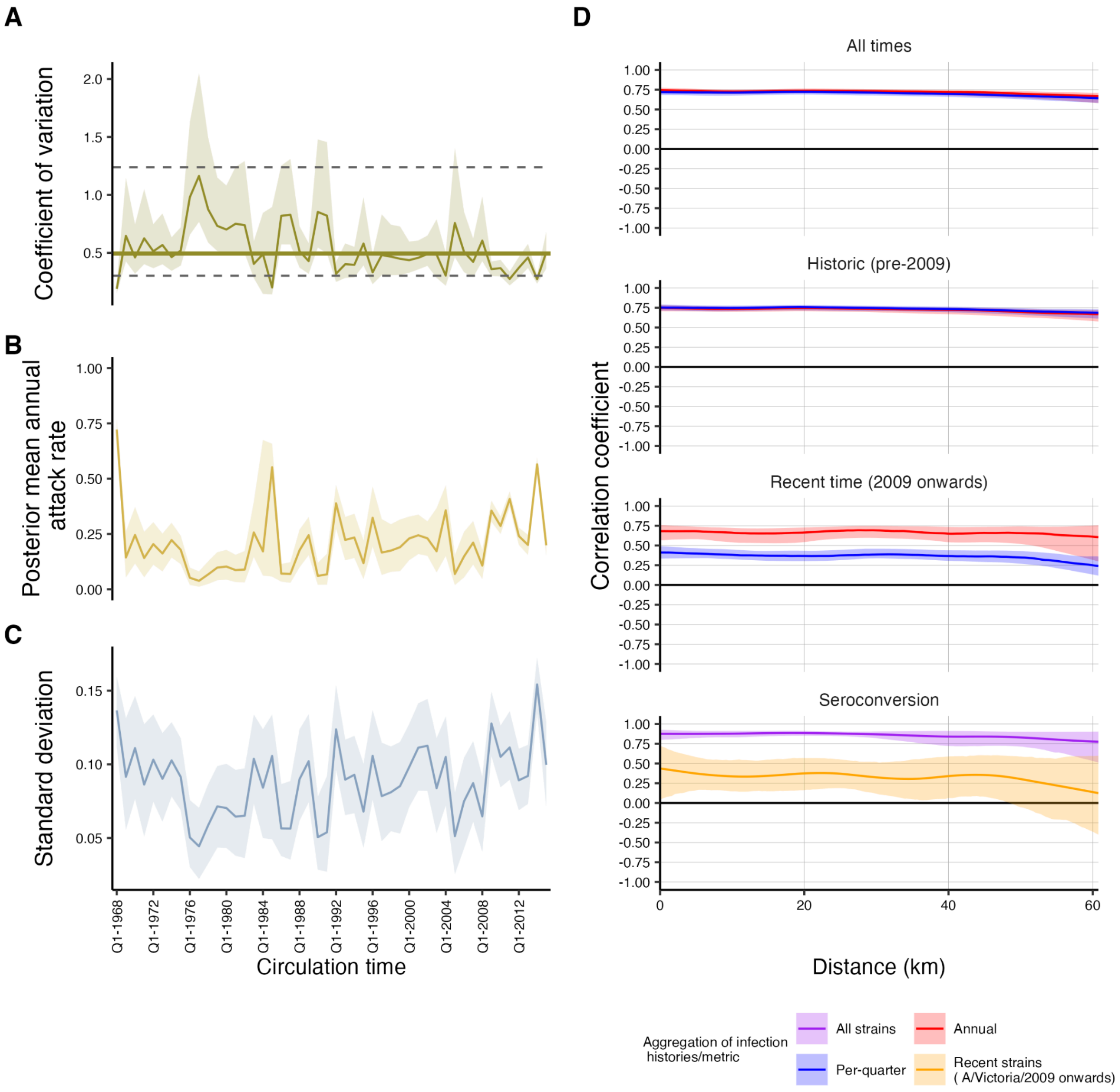


**Figure S9: Spatial variation in annual attack rate estimates over time and correlation between nearby locations. (A)** Coefficient of variation, (**B**) overall mean and (**C**) standard deviation of posterior median annual attack rate estimates from the 40 study locations. Solid lines and shaded regions show posterior medians and 95% credible intervals. In (**A**), the solid horizontal line shows the overall mean coefficient of variation across all time. Dashed horizontal lines show 95% quantiles of simulated coefficients of variation under the assumption that attack rates are the same across space. (**D**) Fitted spline correlograms showing spatio-temporal correlation in attack rates and proportion seroconverted with increasing distance. The first three plots show the spline correlogram calculated using the *Scnf* function from the *ncf* R-package for each of 100 posterior samples for the 40 location-specific attack rates. Solid lines and shaded regions show median and 95% quantiles of the predicted covariance function for these 100 samples, coloured by the level of aggregation used to calculate the attack rates. Each subplot shows the same calculation using either attack rates from all times, prior to Q1-2009 or Q1-2009 onwards. For the final plot ("Seroconversion"*)*, we calculated the spatial correlation in the proportion seroconverted in each of the 40 study locations, treating strain isolation time as the

medRxiv preprint doi: https://doi.org/10.1101/2024.03.18.24304371; this version posted April 5, 2024. The copyright holder for this preprint (which was not certified by peer review) is the author/funder, who has granted medRxiv a license to display the preprint in perpetuity.
It is made available under a CC-BY 4.0 International license .

time variable. Solid lines and shaded regions show median and 95% quantiles of 1000 bootstrapped observations. We repeated the analysis using either seroconversion to all strains, or only A/Victoria/2009, A/Perth/2009, A/Texas/2012 and A/HongKong/2014.

medRxiv preprint doi: https://doi.org/10.1101/2024.03.18.24304371; this version posted April 5, 2024. The copyright holder for this preprint (which was not certified by peer review) is the author/funder, who has granted medRxiv a license to display the preprint in perpetuity.
It is made available under a CC-BY 4.0 International license .

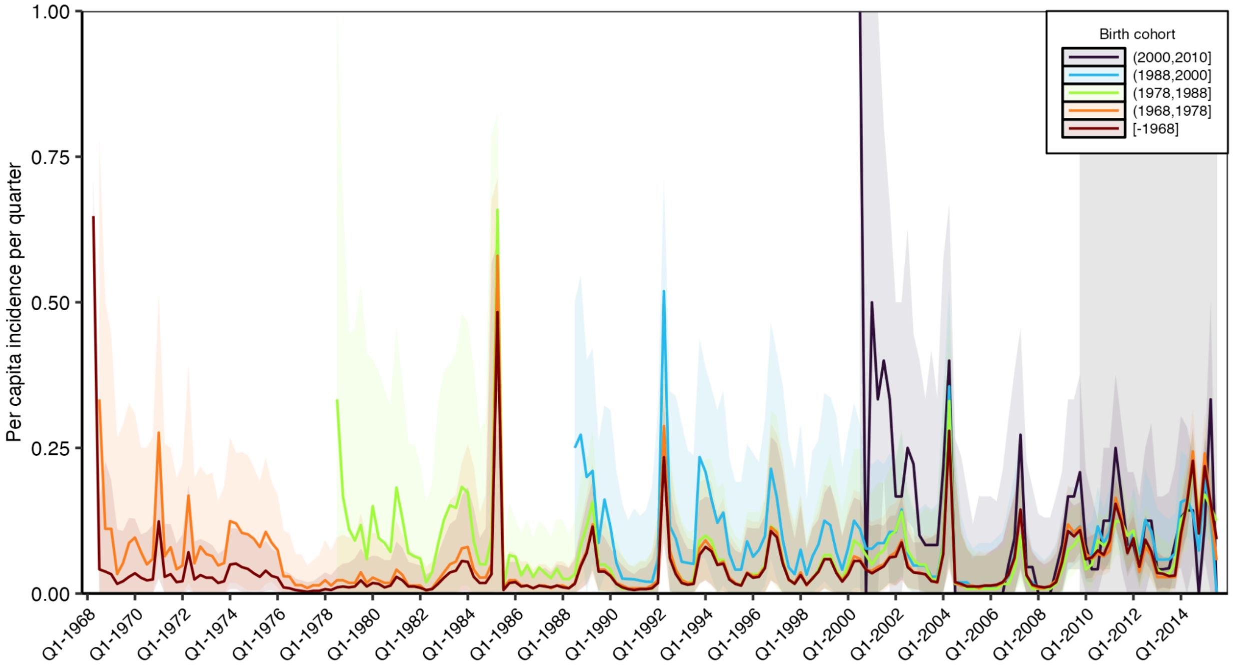


**Figure S10: Quarterly attack rates by birth cohort.** Model predicted per-capita incidence per quarter stratified into 5 birth cohorts. Attack rates were estimated by dividing the number of inferred infections by the number alive in each birth cohort in each 3 month period. Solid lines show the posterior median estimate from 1000 posterior samples. Shaded regions show 95% credible intervals from 1000 posterior samples. Gray shaded box shows duration of the Fluscape study.

medRxiv preprint doi: https://doi.org/10.1101/2024.03.18.24304371; this version posted April 5, 2024. The copyright holder for this preprint (which was not certified by peer review) is the author/funder, who has granted medRxiv a license to display the preprint in perpetuity.
It is made available under a CC-BY 4.0 International license .

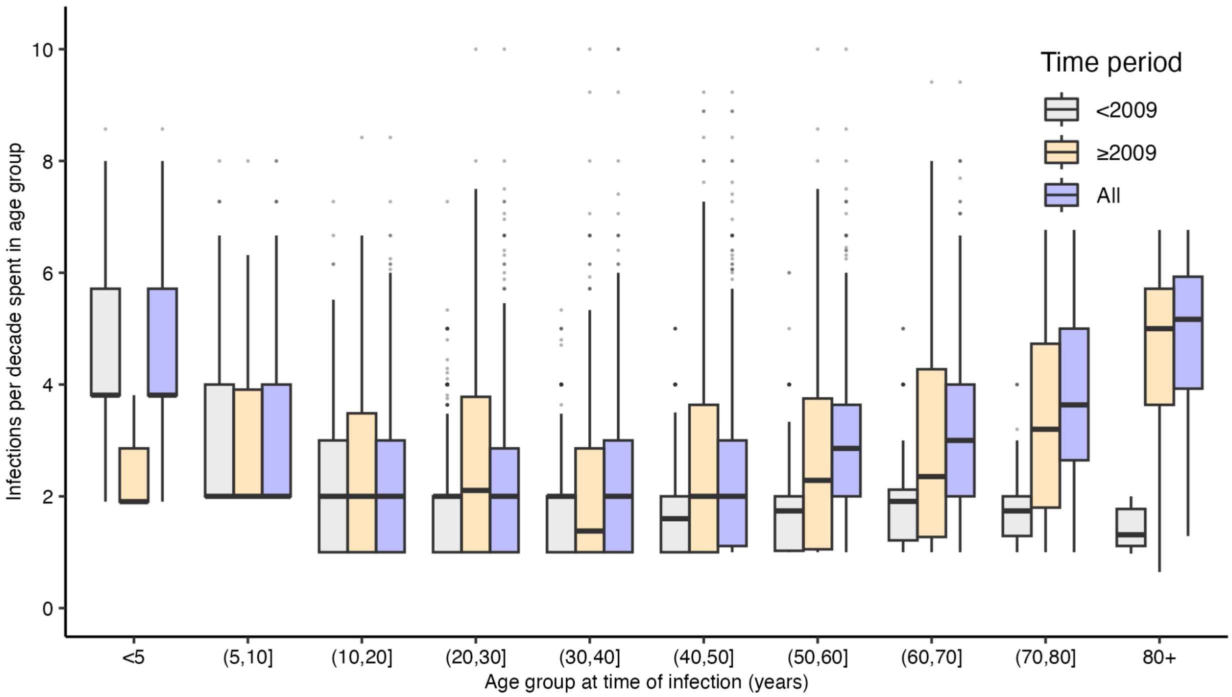


**Figure S11: Posterior median number of infections per 10 year period stratified by age group at the time of infection, including either all time periods (blue), time periods during the Fluscape study period (Q4-2009 onward; orange) or only time periods prior to the Fluscape study (prior to Q4-2009; grey).** Excludes infection states for individuals who spent less than 2 years in that age group. We present infection rate estimates using only infections from time periods prior to the first serum sample in Q4-2009 in Figure 3C. This is because there are many individuals representing the oldest age group at time of infection for time periods post Q4-2009, but relatively few from pre Q4-2009 (as individuals who were very old in historical time periods are no longer alive). In contrast, younger age groups are better represented across historical time periods (as those individuals are still alive at the time of sampling). Combining this biased representation of older individuals with much higher estimated incidence rates in recent time periods weighs the infection rate estimates for the older age groups much higher simply because most of their infections come from this time period. Therefore, we present age-stratified infection rate estimates using only pre Q4-2009 infections in the main text.

medRxiv preprint doi: https://doi.org/10.1101/2024.03.18.24304371; this version posted April 5, 2024. The copyright holder for this preprint (which was not certified by peer review) is the author/funder, who has granted medRxiv a license to display the preprint in perpetuity.
It is made available under a CC-BY 4.0 International license .

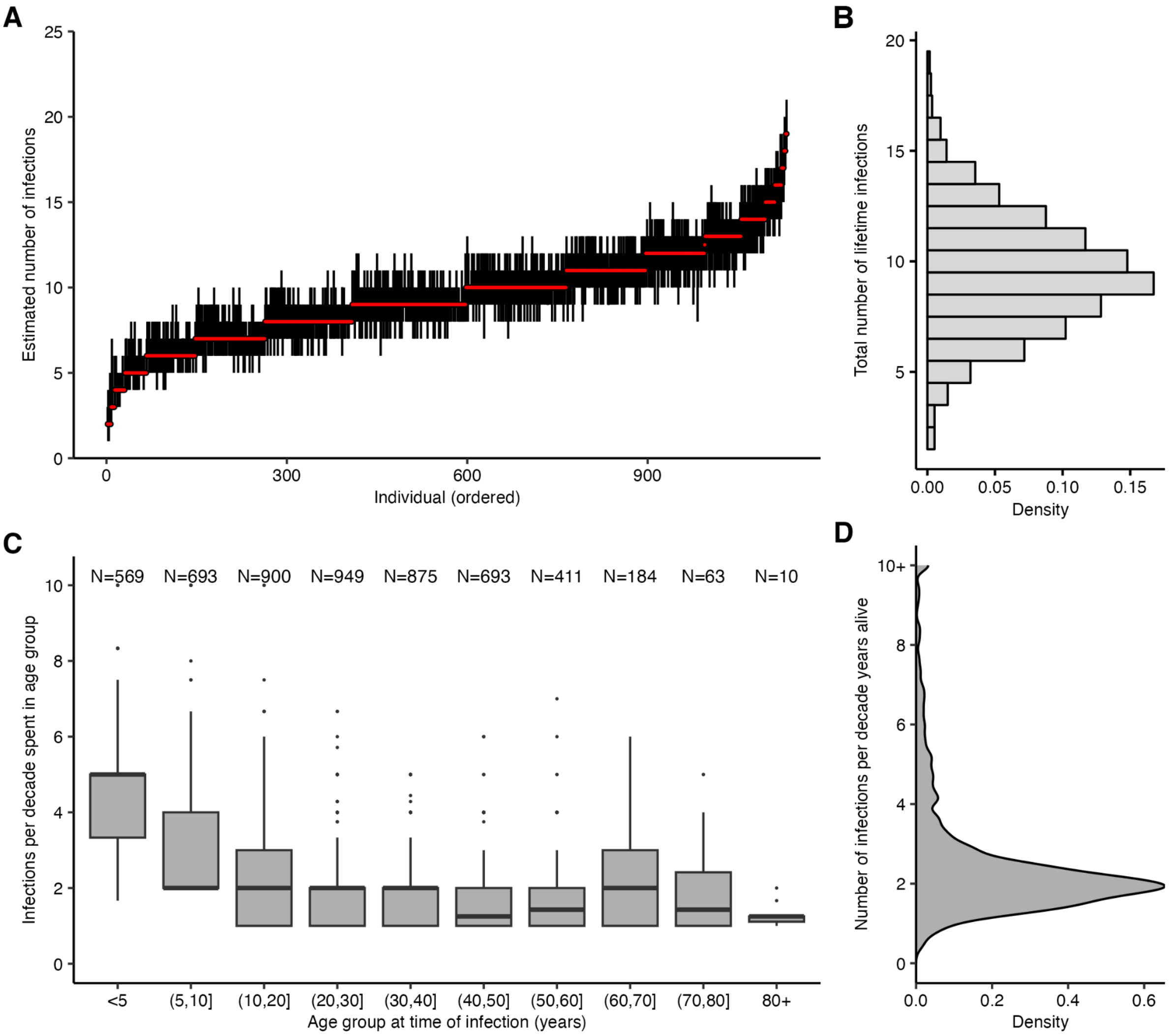


**Figure S12: Age-specific patterns of infection under an alternative infection history model using estimates from the model version described in Supplementary Text 1.** Results shown are identical to those in Figure 3, but assuming that (i) individuals can only be infected once per year (i.e., annual resolution infection histories rather than quarterly); (ii) the infection history model is placed upon an individual's total number of lifetime infections and not their per-time probability of infection (see [35] for further detail on implications of different prior assumptions); (iii) we did not remove runs of continuous infections from the posteriors. (**A**) Pointrange plot shows median and 95% CrI on the total number of lifetime infections for each individual, ordered by increasing age. (**B**) Distribution of the total number of infections across all individuals based on the posterior median total number of infections. (**C**) Posterior median number of infections per 10 year period stratified by age group at the time of infection, excluding individuals who spent less than 2 years in that age group, and including only time periods prior to Q4-2009. Text shows sample size within each age group – note this does not sum to the number of individuals in the sample, as individuals contribute to multiple age groups during their lifetime. (**D**) Posterior median number of infections per 10 years alive across all individuals. Under this prior, we estimated that individuals are infected 2.08 times per 10 year period (posterior median; 95% CrI: 1.04-7.62).

medRxiv preprint doi: https://doi.org/10.1101/2024.03.18.24304371; this version posted April 5, 2024. The copyright holder for this preprint (which was not certified by peer review) is the author/funder, who has granted medRxiv a license to display the preprint in perpetuity.
It is made available under a CC-BY 4.0 International license .

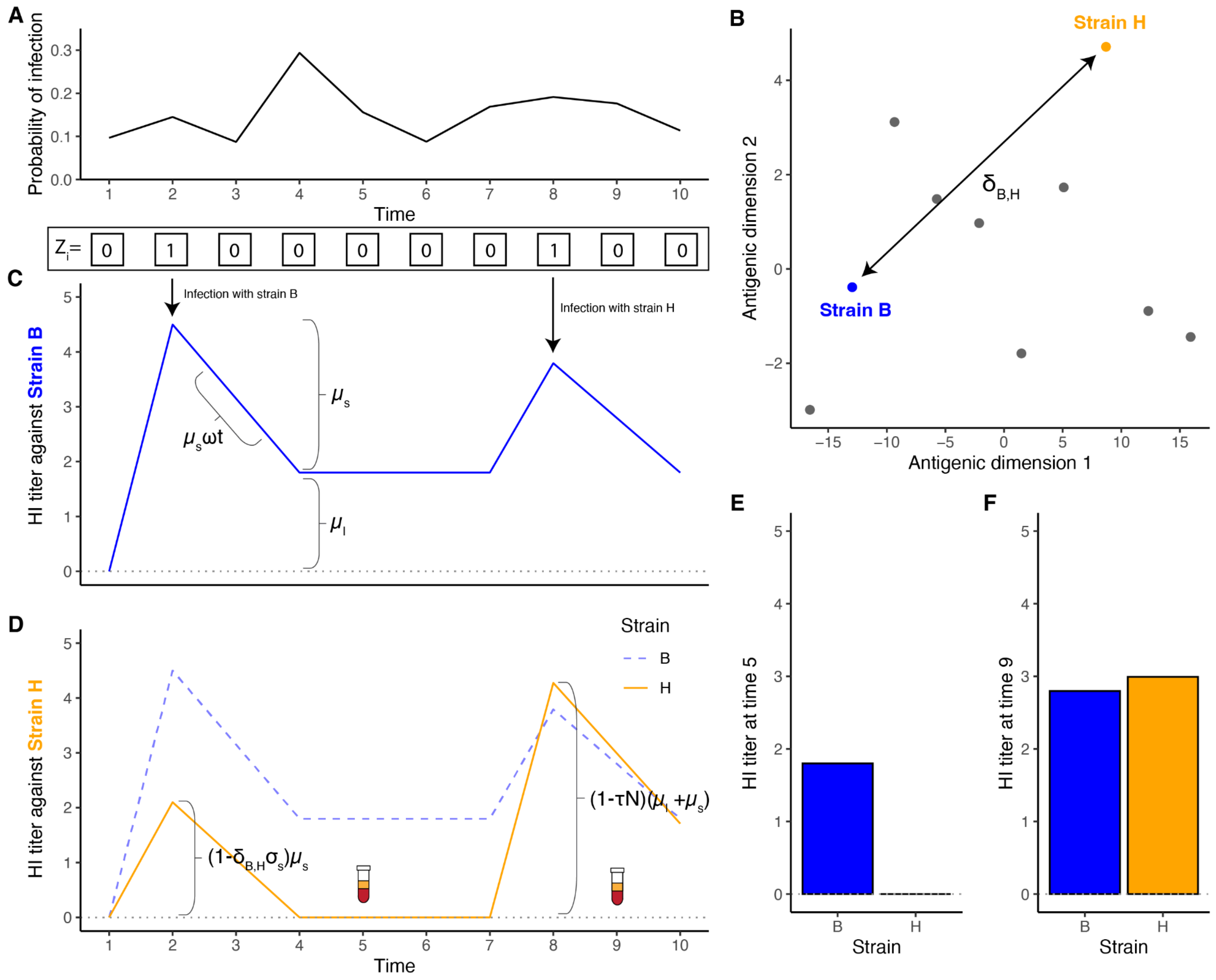


**Figure S13: Schematic of the full *serosolver* model representing a single individual infected with two strains, B and H, over a 10 year time period.** (**A**) Example, randomly generated population-level infection probabilities. At the population level, the model describes a per-time-period probability of infection applied to the whole whole population. These probabilities are used to simulate a vector of latent binary infection states, $Z_i$, for each individual as a series of independent Bernoulli trials (shown as a vector of 1s and 0s). (**B**) The antigenic relatedness of A/H3N2 strains is given by an antigenic map, where the degree of cross reactivity between any two strains is given by their euclidean distance on the map. (**C**) Antibody levels against the infecting strain (strain B) are boosted and wane, given by the summation of transient short-term boosting and persistent long-term boosting. (**D**) Infection with strain B also induces cross-reactive antibodies against all other strains, here showing antibody levels to strain H. The degree of cross-reactivity is proportional to the antigenic distance between the infecting and measured strain. Later on, the individual is infected again, this time with strain H, inducing further antibody boosting and waning. An antigenic seniority parameter, τ, reduces each successive boost as a function of the number of previous infections, N. Snapshots of these underlying antibody kinetics are observed through serum samples (blood vials; (**E**) and (**F**)) distributed according to a truncated, discretized normal distribution with standard deviation parameter ε.

medRxiv preprint doi: https://doi.org/10.1101/2024.03.18.24304371; this version posted April 5, 2024. The copyright holder for this preprint
(which was not certified by peer review) is the author/funder, who has granted medRxiv a license to display the preprint in perpetuity.
It is made available under a CC-BY 4.0 International license .

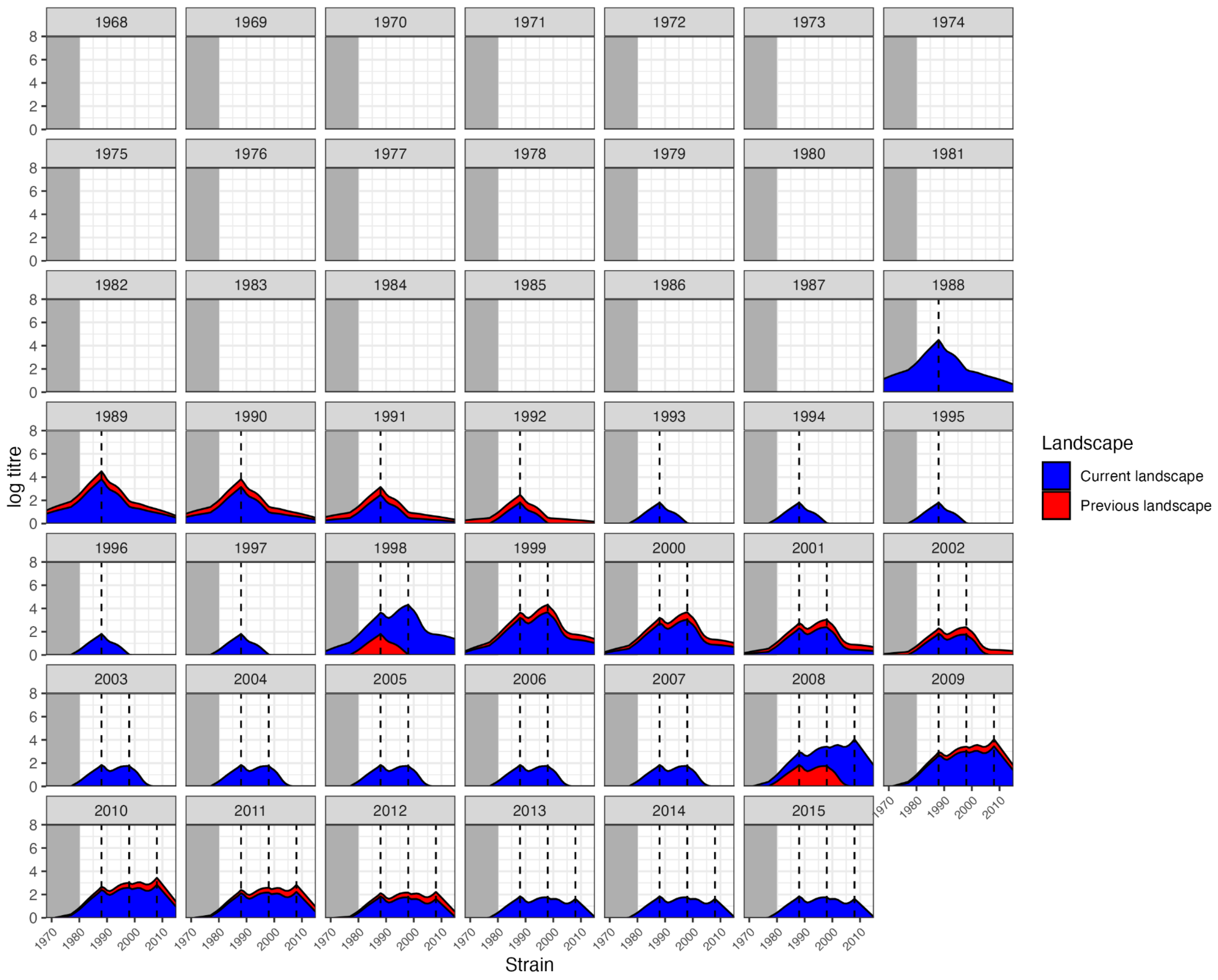


**Figure S14: Simulated antibody landscapes and infection histories over time for one individual using the antibody kinetics model.** Each subplot shows the antibody landscape for that time period. The blue region gives the antibody landscape in that time period, whereas the red region gives the antibody landscape in the preceding time period. The x-axis of each subplot gives the identity of the strain assumed to be circulating in that year. The grey region shows the time period and thus strains that circulated before the individual was born. Vertical dashed lines give the timing/strains the individual was infected with.

medRxiv preprint doi: https://doi.org/10.1101/2024.03.18.24304371; this version posted April 5, 2024. The copyright holder for this preprint (which was not certified by peer review) is the author/funder, who has granted medRxiv a license to display the preprint in perpetuity.
It is made available under a CC-BY 4.0 International license .

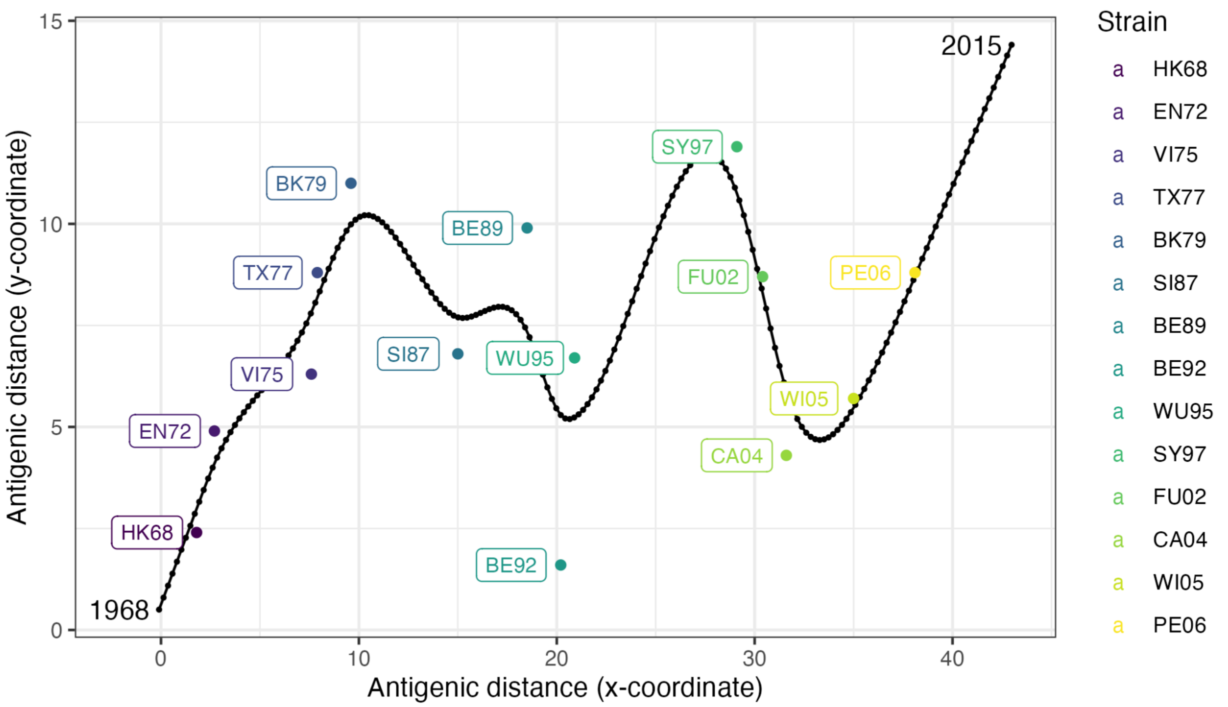


**Figure S15: Antigenic coordinates of measured strains and strains assumed to be circulating in each time period.** Each colored point shows the location of the labeled strain on the antigenic map given in [37]. Strains which are further apart are less antigenically similar, and therefore exhibit less cross-reactivity following a seroresponse. The smoothing spline shows the inferred coordinates of each strain *j* assumed to have circulated in each 3-month time period, where each black point shows the assumed location in successive time periods. First, a cubic smoothing spline was fitted to the locations of the measured strain with smoothing parameter 0.3. Second, a linear model was fitted to predict the x-coordinate as a function of the strain isolation time. Finally, we generated predicted x-coordinates for each possible *j* given the circulation time from the linear model, and then used the predicted x-coordinate to predict the y-coordinate from the fitted smoothing spline. The antigenic distance between each pair of strains *k* and *j* was then calculated based on their Euclidean distance.

medRxiv preprint doi: https://doi.org/10.1101/2024.03.18.24304371; this version posted April 5, 2024. The copyright holder for this preprint (which was not certified by peer review) is the author/funder, who has granted medRxiv a license to display the preprint in perpetuity.
It is made available under a CC-BY 4.0 International license .

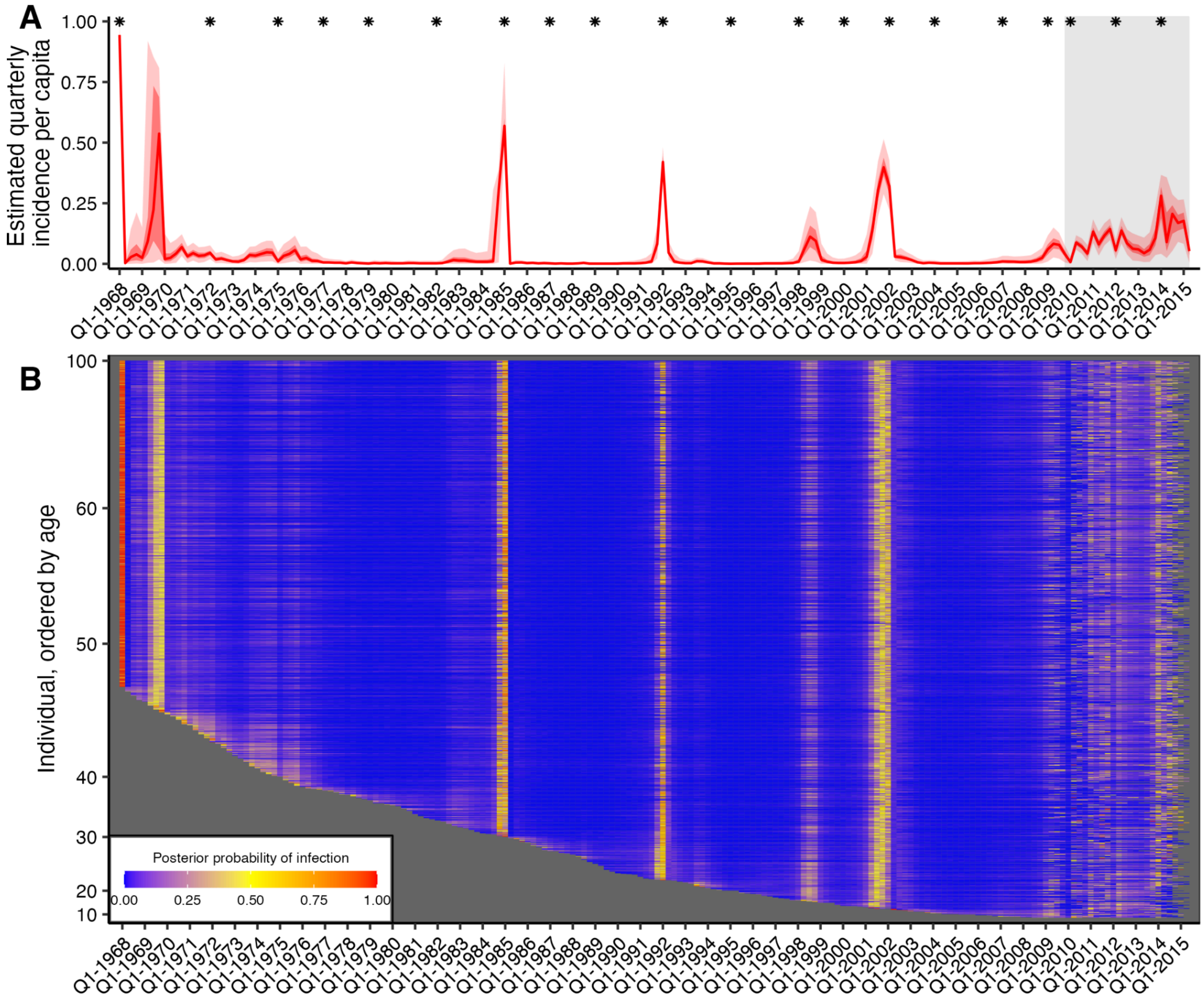


**Figure S16: Quarterly incidence and individual infection histories from the Fluscape dataset without strain-specific measurement offsets.** Identical to Figure 2, but without the inclusion of strain-specific measurement offsets in the observation model. (**A**) Model predicted per-capita incidence per quarter. Attack rates were estimated by dividing the number of inferred infections by the number alive in each 3 month period. Red line shows the posterior median estimate from 1000 posterior samples. Dark and light red shaded regions show 50% and 95% credible intervals respectively from 1000 posterior samples. Gray shaded box shows duration of the Fluscape study. Asterisks mark times from which a sample circulating strain was tested. (**B**) Inferred infection histories for each individual. Each row represents an individual ordered by increasing age in years. Each column represents the time of a potential infection. Cells are shaded based on the number of the posterior samples with an infection at that time divided by the total number of posterior samples for that infection state.

medRxiv preprint doi: https://doi.org/10.1101/2024.03.18.24304371; this version posted April 5, 2024. The copyright holder for this preprint
(which was not certified by peer review) is the author/funder, who has granted medRxiv a license to display the preprint in perpetuity.
It is made available under a CC-BY 4.0 International license .

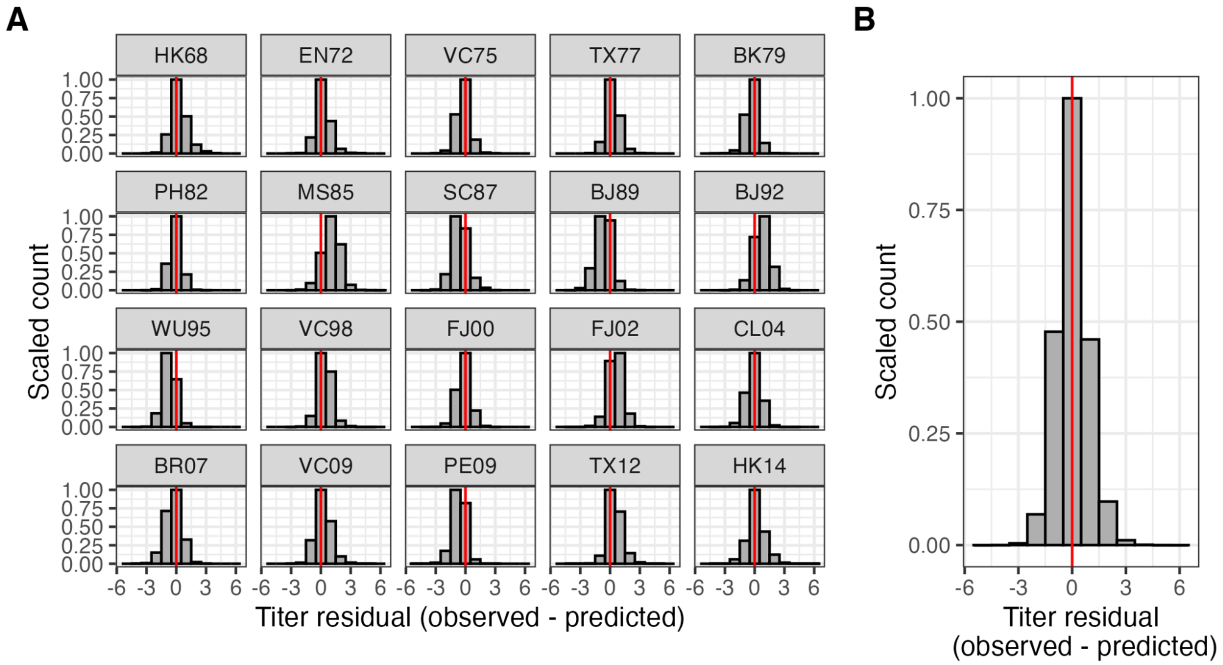


**Figure S17**: **Distribution of antibody titre prediction errors (observed - model predicted) when fitting the *serosolver* model ignoring strain-specific measurement offsets.** (**A**) Distribution of titre prediction errors stratified by tested A/H3N2 strain. (**B**) Overall distribution of titre prediction errors across all measured viruses. Vertical red line shows *x=0*; buckets to the right of the red line suggest underestimation of titres; histograms to the left of the red line buckets suggest overestimation of titres.

medRxiv preprint doi: https://doi.org/10.1101/2024.03.18.24304371; this version posted April 5, 2024. The copyright holder for this preprint (which was not certified by peer review) is the author/funder, who has granted medRxiv a license to display the preprint in perpetuity.
It is made available under a CC-BY 4.0 International license .

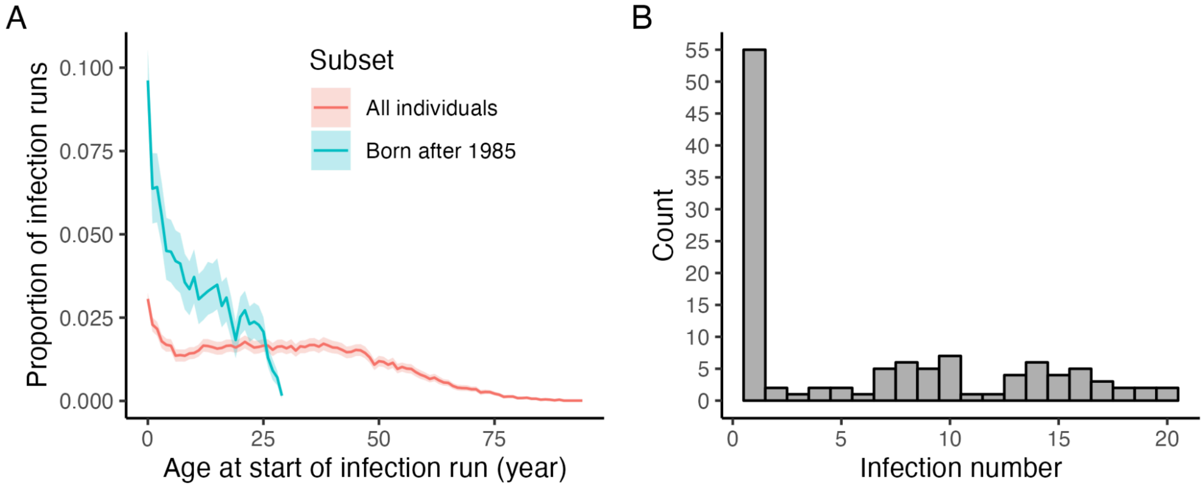


**Figure S18: Runs of repeated infections are disproportionately more likely to occur early in life.** (**A**) Proportion of infection episodes which are estimated as runs of consecutive infections by age at start of infection run, using either all infection episodes or only those from individuals born after 1985. (**B**) Number of inferred infection episodes which are estimated as runs of consecutive infections stratified by the infection order in each individual's infection history. For example, if the run is the first infection an individual has experienced, this is given an infection number of one. Of the 10,558 distinct infection episodes, 757 (posterior median; 95% CrI: 676-861) were runs of two consecutive infections, 79 (posterior median; 95% CrI: 62-97) were runs of 3 consecutive infections and 19 (posterior median; 95% CrI: 13-27) were runs of 4 or more.

medRxiv preprint doi: https://doi.org/10.1101/2024.03.18.24304371; this version posted April 5, 2024. The copyright holder for this preprint (which was not certified by peer review) is the author/funder, who has granted medRxiv a license to display the preprint in perpetuity.
It is made available under a CC-BY 4.0 International license .

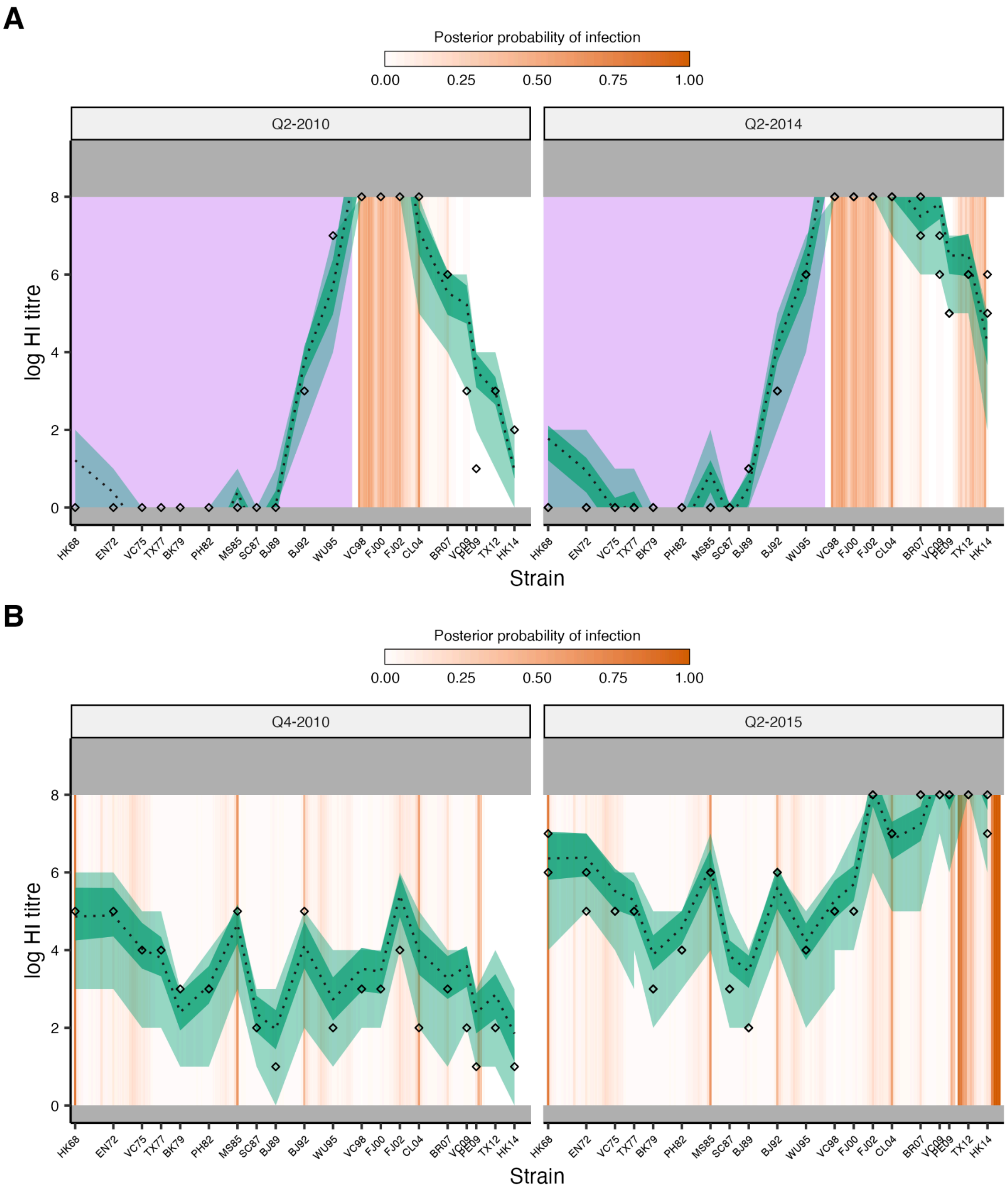


**Figure S19: Model fits demonstrating typical profiles where consecutive infection runs were imputed.** Model-predicted titres compared to observed HI titres at each sampling time for two individuals, as in Fig S4. Diamonds show titre measurements; green shaded region shows 95% CrI and 95% prediction intervals; dashed line shows posterior median; orange bars show posterior probability of infection in a given time window. Purple rectangles show time periods prior to birth. (**A**) Example of an individual estimated to have experienced multiple consecutive infections immediately following birth to explain high titres. (**B**) Example of an individual estimated to have experienced multiple consecutive infections between the two serum sampling times to explain the drastic increase in titres to recently circulating strains.

medRxiv preprint doi: https://doi.org/10.1101/2024.03.18.24304371; this version posted April 5, 2024. The copyright holder for this preprint (which was not certified by peer review) is the author/funder, who has granted medRxiv a license to display the preprint in perpetuity.
It is made available under a CC-BY 4.0 International license .

<< Separate file >>

**Figure S20: Observed antibody profiles for all individuals in the Fluscape cohort.** Each subplot shows antibody levels measured against each of the 20 H3N2 strains. The x-axis shows the isolation year of the measured strain. The areas are shaded by sample number, showing titre measurements from the first (blue) and second (red) samples. Grey rectangles mark strains which circulated before that individual was born. The vertical colored lines show the timing of the serum samples relative to the strain isolation times. Plots where the red region extends above the blue region reflect antibody boosting between the first and second serum sample. Where multiple titres were measured against the same strain from the same serum sample, we plotted the mean of the log titres.

<< Separate file >>

**Figure S21: Observed changes in antibody titre for all individuals in the Fluscape cohort.** As in Fig S20, but showing fold-change in titre against each strain between samples. Each subplot shows the change in antibody levels measured against each of the 20 H3N2 strains. The x-axis gives the isolation year of the measured strain. The vertical grey line shows the timing of birth or 1968, whichever was later. Bars are shaded orange to denote antibody boosting and green to denote antibody waning. Horizontal dash lines indicate 2-fold boosting or waning.

medRxiv preprint doi: https://doi.org/10.1101/2024.03.18.24304371; this version posted April 5, 2024. The copyright holder for this preprint (which was not certified by peer review) is the author/funder, who has granted medRxiv a license to display the preprint in perpetuity.
It is made available under a CC-BY 4.0 International license .

**Table S1: self-reported vaccination status at time of first study visit (top) and between study visits (bottom).** Percentages exclude individuals who declined to answer, were unsure, or had missing data.

| **Self-reported vaccination at time of first serum sample (N=1005)** | | | | |
|---|---|---|---|---|
| Never vaccinated | This year | Last year | 2-5 years | 5+ years |
| 88.3% | 1.09% | 3.08% | 2.78% | 4.77% |
| **Reported vaccination since last study visit** | | | | |
| Visit 2 (N=822) | | Visit 3 (N=1020) | | Visit 4 (N=1119) |
| 1.70% | | 1.57% | | 0.536% |

medRxiv preprint doi: https://doi.org/10.1101/2024.03.18.24304371; this version posted April 5, 2024. The copyright holder for this preprint (which was not certified by peer review) is the author/funder, who has granted medRxiv a license to display the preprint in perpetuity.
It is made available under a CC-BY 4.0 International license .

**Table S2: Estimated attack rates and infection patterns 2010-2014 prior to removing runs of consecutive infections.** Percentages shown are posterior median and 95% credible intervals. Attack rate was defined as the proportion of individuals who were infected at least once in that year. "Reinfected" gives the percentage of people that were infected more than once in a year. Bottom table shows the percentage of individuals that were infected 0, 1, 2, 3, 4 or 5 times between 2010-2014 inclusive.

| Year | Measured strain | Estimated attack rate (%) | Reinfected within same year (%) |
|---|---|---|---|
| 2010 | A/Perth/2009 | 28.8% (25.2%-32.1%) | 4.25% (3.01%-5.4%) |
| 2011 | - | 42.9% (39.9%-46.2%) | 5.01% (3.49%-6.98%) |
| 2012 | A/Texas/2012 | 24.7% (20.9%-28.6%) | 2.26% (1.17%-3.61%) |
| 2013 | - | 20.8% (16.5%-24.9%) | 8.41% (7.09%-9.97%) |
| 2014 | A/Hong Kong/2014 | 57.9% (55.0%-60.5%) | 6.02% (5.00%-7.16%) |

| **Proportion of individuals with different total number of infections from 2010-2014** | | | | | |
|---|---|---|---|---|---|
| 0 | 1 | 2 | 3 | 4 | 5 |
| 12.2% (11.2%-13.4%) | 32.7% (30.7%-34.7%) | 32.7% (30.8%-35.2%) | 16.8% (15.0%-18.6%) | 4.78% (3.67%-5.93%) | 0.531% (0.177%-0.973 %) |

medRxiv preprint doi: https://doi.org/10.1101/2024.03.18.24304371; this version posted April 5, 2024. The copyright holder for this preprint (which was not certified by peer review) is the author/funder, who has granted medRxiv a license to display the preprint in perpetuity.
It is made available under a CC-BY 4.0 International license .

# Supplementary Text 1

## 1. Estimation of strain-specific measurement offsets

Our initial approach was to fit the full *serosolver* model exactly as described in the Materials and Methods, but without the inclusion of strain-specific measurement offsets (i.e., set all $\chi_j$ to 0). Although convergence diagnostics were acceptable, we noted that estimated attack rates were extremely high (or low) in some time periods (Fig S16). These unusually high or low estimates were associated with systematic under- or over-estimation of expected log HI titres compared to the observed values (Fig S17). For example, model predicted titres are systematically lower than observations for the viruses that circulated in years 1985 and 2002, but systematically higher for years 1995 and 2010. These strain-specific biases are potentially problematic for the attack rate estimates, as the model can only account for elevated titres against a particular strain through adding more infections. If some viruses have systematically higher antibody titres simply due to measurement bias, then the model may incorrectly infer more infections than occurred.

These systematic measurement biases have a number of possible biological explanations. Serum potency and virus avidity, resulting in different haemagglutinin (HA) reactivity [88,89], are known sources of variation when performing antigenic cartography using ferret sera. This effect was noted by Fonville et al. when using locally-weighted multiple linear regression to fit antibody landscape surfaces to antibody titre data. Fonville et al. found that some viruses had systematically under-predicted values (e.g., NL/620/89) whereas others were systematically overestimated (e.g., Victoria/361/11) [37]. This phenomenon was also described by Bedford et al. when simultaneously performing antigenic cartography alongside phylogenetic tree reconstruction using both genetic and HI titre data [90]. Bedford et al. found models that explicitly estimated parameters for “virus avidity” to represent the contribution of virus-specific effects to observed titres were better supported than models that did not.

Including a strain-specific offset term as part of the observation process enables the antibody kinetics and infection model described here to account for these systematic biases. In lieu of generating these strain-specific offset terms from experimental data, we would ideally jointly infer these offsets alongside the infection histories and antibody kinetics parameters. However, our attempts to do so using the infection history prior used in the main text were unsuccessful

medRxiv preprint doi: https://doi.org/10.1101/2024.03.18.24304371; this version posted April 5, 2024. The copyright holder for this preprint (which was not certified by peer review) is the author/funder, who has granted medRxiv a license to display the preprint in perpetuity. It is made available under a CC-BY 4.0 International license .

and we could not generate converged chains. This is likely because the offset terms and attack rate estimates were highly correlated; elevated titres against a strain that circulated in a given year may be explained either by high seroresponse rates or systematic overestimation of titres to that strain.

Instead, we generated estimates for the measurement offsets using a modified version of the main text model. We fit the same antibody kinetics and infection histories to the full Fluscape dataset, but with three changes: (1) the observation model included an additional, estimated strain-specific offset parameter, $\chi_j$, for each measured strain as described in the Materials and Methods; (2) we estimated infection histories at an annual rather than 3-monthly resolution to reduce the parameter space to be explored; (3) we used a different infection history prior (prior version 3 in *serosolver*) which led to much better convergence and identifiability of the offset terms at the expense of less interpretable attack rate estimates. This alternative prior version places a Beta-Binomial prior on each individual's expected number of lifetime infections and, unlike the prior used in the main text, assumes that an individual's probability of infection in a given time period is unrelated to any other individual:

$$P(\mathbf{Z}) = \prod_{i=1}^{n} P(\mathbf{Z_i}) = \prod_{i=1}^{n} \int_0^1 P(\mathbf{Z_i}|\mathbf{\Lambda_i} = \lambda) P(\mathbf{\Lambda_i} = \lambda) d\lambda = \prod_{i=1}^{n} \frac{B(\alpha + k_i, \beta + m_i + k_i)}{B(\alpha, \beta)}$$

Where $k_i$ is the total number of infections experienced by individual $i$, and $m_i$ is the number of time periods individual $i$ could be infected. This prior is suitable for fitting antibody landscapes to each individual's antibody profile, but generates less interpretable attack rate estimates [35]. Model fitting was exactly the same as described in the main text, but 6 MCMC chains were run for only 12,000,000 iterations with the first 2,000,000 discarded as burn-in. Each strain-specific offset term was estimated under a multi-level model, assuming that each offset term in $\boldsymbol{\chi}$ was drawn from the same normal distribution with mean 0 and unknown standard deviation.

Fig S22 shows that the model-predicted titres provided a better fit to the data with the added offset terms. We used the maximum posterior probability estimates for each $\chi$ as a fixed parameter for the model used in the main text (Table S3). Note that the model without the offset terms produced very similar antibody kinetics parameter estimates (Table S4), as well as age- and location-specific attack rate patterns, to those shown in the main text.

medRxiv preprint doi: https://doi.org/10.1101/2024.03.18.24304371; this version posted April 5, 2024. The copyright holder for this preprint (which was not certified by peer review) is the author/funder, who has granted medRxiv a license to display the preprint in perpetuity.
It is made available under a CC-BY 4.0 International license .

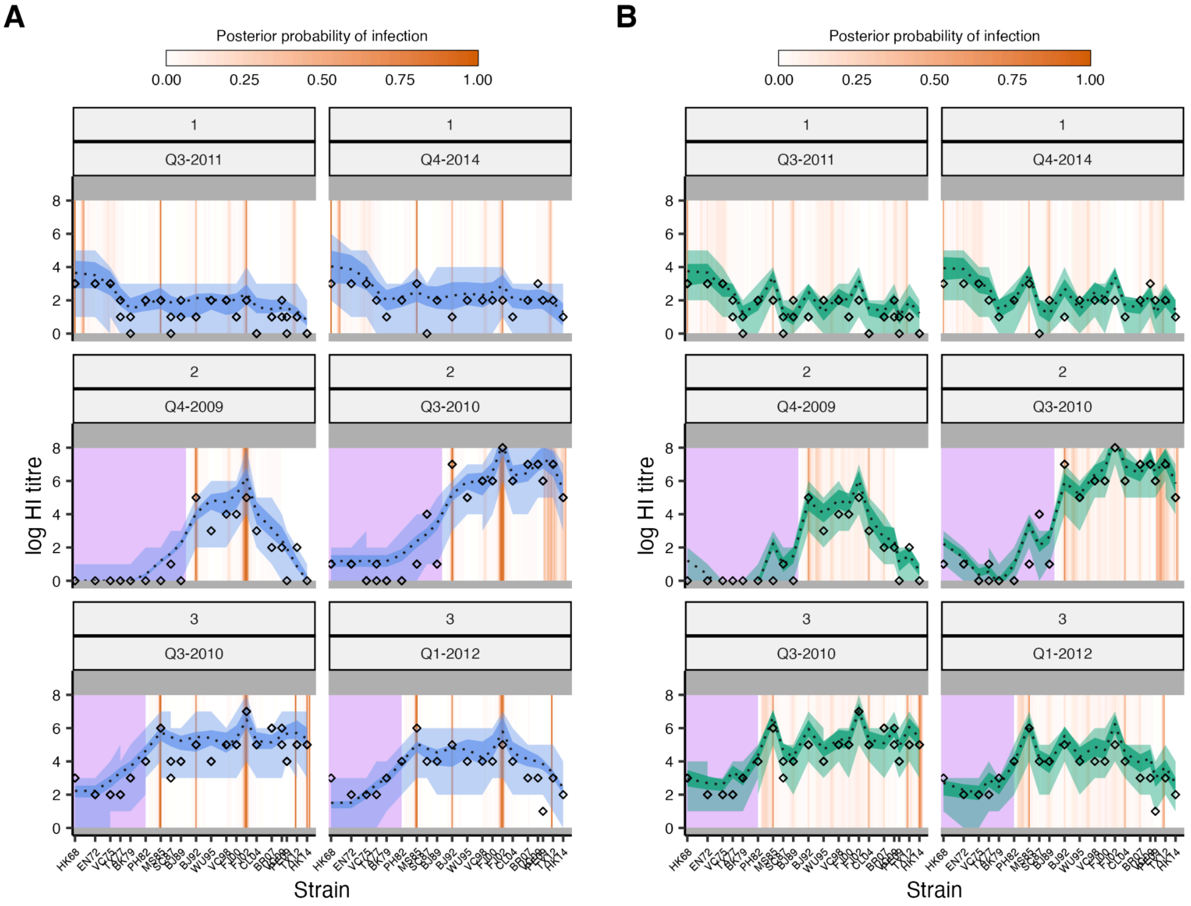


**Figure S22: Comparison of estimated antibody landscapes from the same model fit with and without strain-specific measurement offsets.** Rows represent individuals. Subplots show antibody titres based on serum samples taken at that time. X-axis represents a position along the antigenic summary path. Black diamonds show observed titres. Black line and blue or green shaded regions show posterior median and 95% credible intervals (CrI) on model-predicted latent titres (dark blue/green) and 95% prediction intervals (light blue/green). Orange bars show posterior probability of infection in that 3-month window. Grey rectangles denote the limit of detection of the HI assay. Purple rectangles show time periods prior to birth. (**A**) Model-predicted titres compared to observed HI titres at each sampling time for three randomly selected individuals, as in Fig S5, but from fitting the model without strain-specific measurement offsets. (**B**) as in (**A**), but with the estimated strain-specific measurement offsets included.

medRxiv preprint doi: https://doi.org/10.1101/2024.03.18.24304371; this version posted April 5, 2024. The copyright holder for this preprint (which was not certified by peer review) is the author/funder, who has granted medRxiv a license to display the preprint in perpetuity.
It is made available under a CC-BY 4.0 International license .

**Table S3: Strain-specific measurement offset terms used in the main model fits.** Values shown are maximum posterior probability estimates from a less flexible version of the model fit to the same data (described in Supplementary Text 1). Values shown to three significant figures.

| Strain | Fixed value |
| --- | --- |
| A/HongKong/1968 | 1.216 |
| A/England/1972 | 0.387 |
| A/Victoria/1975 | -0.643 |
| A/Texas/1977 | -0.476 |
| A/Bangkok/1979 | -1.392 |
| A/Philippines/1982 | -1.139 |
| A/Mississippi/1985 | 0.139 |
| A/Sichuan/1987 | -1.941 |
| A/Beijing/1989 | -2.302 |
| A/Beijing/1992 | -0.386 |
| A/Wuhan/1995 | -1.847 |
| A/Victoria/1998 | -0.387 |
| A/Fujian/2000 | -0.573 |
| A/Fujian/2002 | 0.746 |
| A/California/2004 | -0.515 |
| A/Brisbane/2007 | -0.655 |
| A/Victoria/2009 | 0.028 |
| A/Perth/2009 | -0.984 |
| A/Texas/2012 | 0.217 |
| A/HongKong/2014 | 0.027 |

medRxiv preprint doi: https://doi.org/10.1101/2024.03.18.24304371; this version posted April 5, 2024. The copyright holder for this preprint (which was not certified by peer review) is the author/funder, who has granted medRxiv a license to display the preprint in perpetuity.
It is made available under a CC-BY 4.0 International license .

**Table S4: Estimated antibody kinetics parameters under the model without strain-specific measurement offsets.**

| Parameter | Description | Estimate (posterior median; 95% credible intervals) |
|---|---|---|
| $\mu_l$ | Long-term boosting | 1.54 (1.50-1.58) |
| $\mu_s$ | Short-term boosting | 1.95 (1.82-2.07) |
| $\tau$ | Antigenic seniority term | 0.0329 (0.0307-0.0357) |
| $\omega$ | Waning rate parameter for the short-term response (per 3-months) | 0.872 (0.776-1.02) |
| $\sigma_l$ | Long-term cross reactivity | 0.0896 (0.0889-0.0898) |
| $\sigma_s$ | Short-term cross reactivity | 5.17e-05 (5.7e-06-0.000278) |
| $\varepsilon$ | Standard deviation of observations | 0.874 (0.868-0.881) |

medRxiv preprint doi: https://doi.org/10.1101/2024.03.18.24304371; this version posted April 5, 2024. The copyright holder for this preprint (which was not certified by peer review) is the author/funder, who has granted medRxiv a license to display the preprint in perpetuity.
It is made available under a CC-BY 4.0 International license .

# Supplementary Text 2

## 2. Scenario analyses using simulation-recovery experiments

### 2.1 Overview

The full *serosolver* model framework is complex, with many components and parameters to be estimated or fixed. A concern is therefore the identifiability of the estimated model parameters and the potential biases introduced by inappropriate assumptions for the fixed components. To assess the ability of the *serosolver* framework to accurately recover infection histories and antibody kinetics parameters, we simulated a dataset matching the dimensions of the Fluscape survey and performed extensive simulation-recovery experiments testing the robustness of the framework to each step of our inference pipeline. The aim of this analysis was to test how simplifying assumptions and model misspecification might bias our estimates relative to the known ground truth.

### 2.2 Simulation settings

We generated a simulated serosurvey of 1000 individuals of random ages uniformly sampled between 5 and 75 years. We simulated attack rates in 3-month time windows over a 46-year time period loosely based on estimates from the real data – per-quarter attack rates were randomly drawn from a log-normal distribution with mean 0.0375 and standard deviation 1.5 (on the natural scale). We assumed an attack rate of 0.6 in the first time period to represent the pandemic wave of H3N2 in 1968. Random infection histories for each individual were then simulated for each 3-month time period as Bernoulli trials with infection probability given by the simulated attack rates. Based on these infection histories, we then simulated each individual's latent antibody kinetics using assumed model parameter values (Table S5). To reflect the Fluscape serosurvey, we assumed that each individual had two serum samples taken at random times from the last 24 time periods of the simulation, with two titre measurements at each sample against 24 strains uniformly distributed across the simulation period. A crucial addition to the simulation is the inclusion of strain-specific measurement offsets – measurements against each strain were assumed to be shifted relative to the true titre, where these measurement shifts were normally distributed with mean 0 and standard deviation 0.5. We generated random measurement shifts for each measured strain drawn from this distribution. Finally, we used the same antigenic map as described in the main text Materials and Methods.

medRxiv preprint doi: https://doi.org/10.1101/2024.03.18.24304371; this version posted April 5, 2024. The copyright holder for this preprint (which was not certified by peer review) is the author/funder, who has granted medRxiv a license to display the preprint in perpetuity.
It is made available under a CC-BY 4.0 International license .

**Table S5: Description of antibody kinetics parameter values used for the simulation.** Uniform priors were used for all parameters.

| Parameter | Description | Value | Prior lower bound | Prior upper bound | Estimate using the correctly specified model (posterior median; 95% credible intervals) |
|---|---|---|---|---|---|
| $\mu_l$ | Long-term boosting | 1.80 | 0 | 8 | 1.74 (1.71-1.77) |
| $\mu_s$ | Short-term boosting | 2.70 | 0 | 8 | 2.74 (2.53-2.91) |
| $\tau$ | Antigenic seniority term | 0.05 | 0 | 1 | 0.0511 (0.0486-0.0541) |
| $\omega$ | Waning rate parameter for the short-term response (per 3-months) | 0.20 | 0 | 1 | 0.216 (0.188-0.231) |
| $\sigma_l$ | Long-term cross reactivity | 0.10 | 0 | 1 | 0.101 (0.100-0.102) |
| $\sigma_s$ | Short-term cross reactivity | 0.03 | 0 | 1 | 0.0310 (0.0293-0.0329) |
| $\varepsilon$ | Standard deviation of observations | 1.00 | 0 | 25 | 1.00 (0.995-1.01) |

medRxiv preprint doi: https://doi.org/10.1101/2024.03.18.24304371; this version posted April 5, 2024. The copyright holder for this preprint (which was not certified by peer review) is the author/funder, who has granted medRxiv a license to display the preprint in perpetuity.
It is made available under a CC-BY 4.0 International license .

## 2.3 Scenario analyses

### 2.3.1 Re-estimating the strain-specific measurement offsets

The first step of the inference pipeline is to estimate the strain-specific measurement offset terms, where some strains have systematically higher or lower titres measured in the HI assay after accounting for differences in time-since-infection, exposure history and random measurement error. Following the same approach as outlined in Supplementary Text 1, we fitted the *serosolver* model to the simulated data to re-estimate the strain-specific measurement offsets. The recovered parameter estimates were close to those used to simulate the data, though there were some systematic biases (Fig S23). Most notably the total number of infections was overestimated, the antibody boosting parameters were underestimated and the cross-reactivity parameters were overestimated. The estimated measurement offset parameters were also biased but all in the right direction (Fig S24). Relative to the prior ranges (uniform between -3 and 3), the parameter estimates were close to their true values, giving us confidence that our estimates for the measurement offset terms were still informative and could thus be used as fixed values for subsequent model fits.

medRxiv preprint doi: https://doi.org/10.1101/2024.03.18.24304371; this version posted April 5, 2024. The copyright holder for this preprint (which was not certified by peer review) is the author/funder, who has granted medRxiv a license to display the preprint in perpetuity.
It is made available under a CC-BY 4.0 International license .

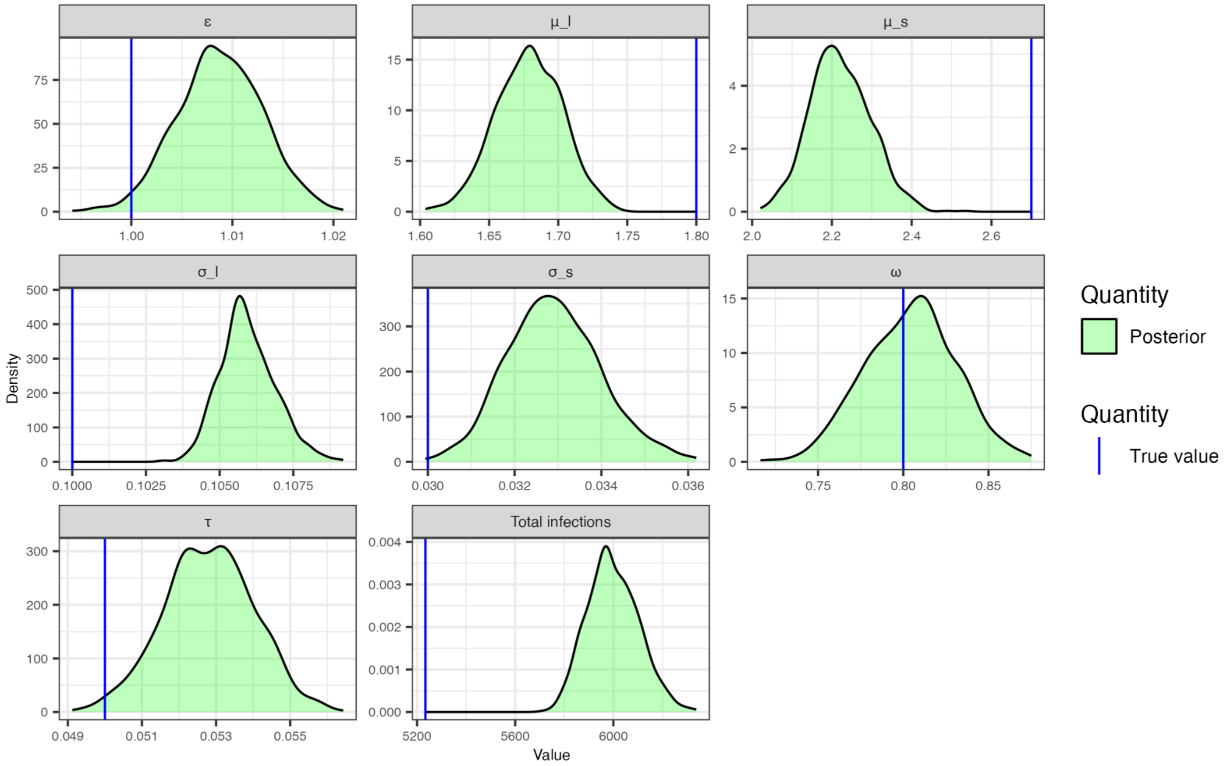


**Figure S23:** Estimated posterior distributions for antibody kinetics parameters (green shaded region) using the fitted model described in Supplementary Text 1 compared to true values used in the simulation (blue line).

medRxiv preprint doi: https://doi.org/10.1101/2024.03.18.24304371; this version posted April 5, 2024. The copyright holder for this preprint (which was not certified by peer review) is the author/funder, who has granted medRxiv a license to display the preprint in perpetuity.
It is made available under a CC-BY 4.0 International license .

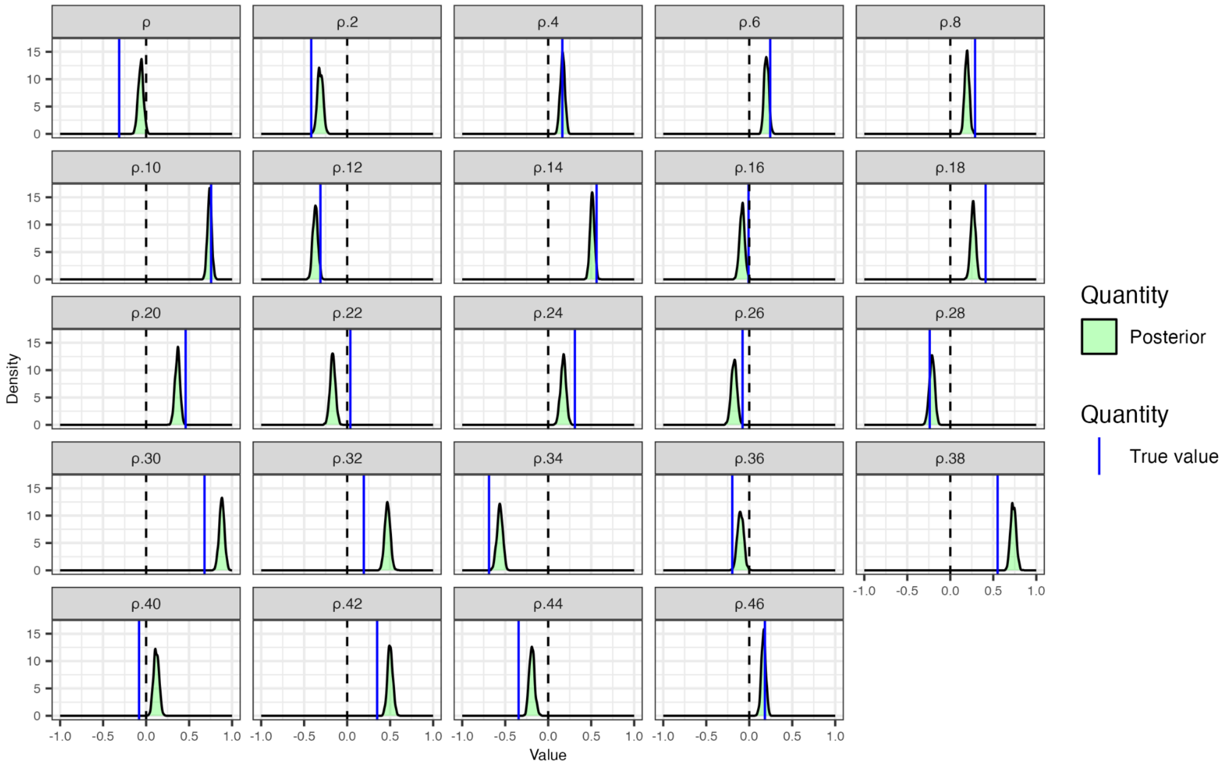


**Figure S24:** Estimated posterior distributions for strain-specific measurement offsets (green shaded region) using the fitted model described in Supplementary Text 1 compared to true values used in the simulation (blue line).

medRxiv preprint doi: https://doi.org/10.1101/2024.03.18.24304371; this version posted April 5, 2024. The copyright holder for this preprint (which was not certified by peer review) is the author/funder, who has granted medRxiv a license to display the preprint in perpetuity. It is made available under a CC-BY 4.0 International license .

### 2.3.2 Correctly specified model

Using the re-estimated strain-specific measurement offsets estimated from Section 2.3.1, we fit the *serosolver* model to the simulated data under the assumption that the model was correctly specified. This provides a sense check that the inference framework is able to accurately recover the antibody kinetics parameter estimates, attack rates and infection histories when the true generative model is known. 5 MCMC chains were run for 1,000,000 iterations with a 200,000 iteration burn in period. The model accurately re-estimated the true simulation parameter values (Table S5), though with a slight underestimation of the long-term boosting parameter (Fig S25).

medRxiv preprint doi: https://doi.org/10.1101/2024.03.18.24304371; this version posted April 5, 2024. The copyright holder for this preprint (which was not certified by peer review) is the author/funder, who has granted medRxiv a license to display the preprint in perpetuity.
It is made available under a CC-BY 4.0 International license .

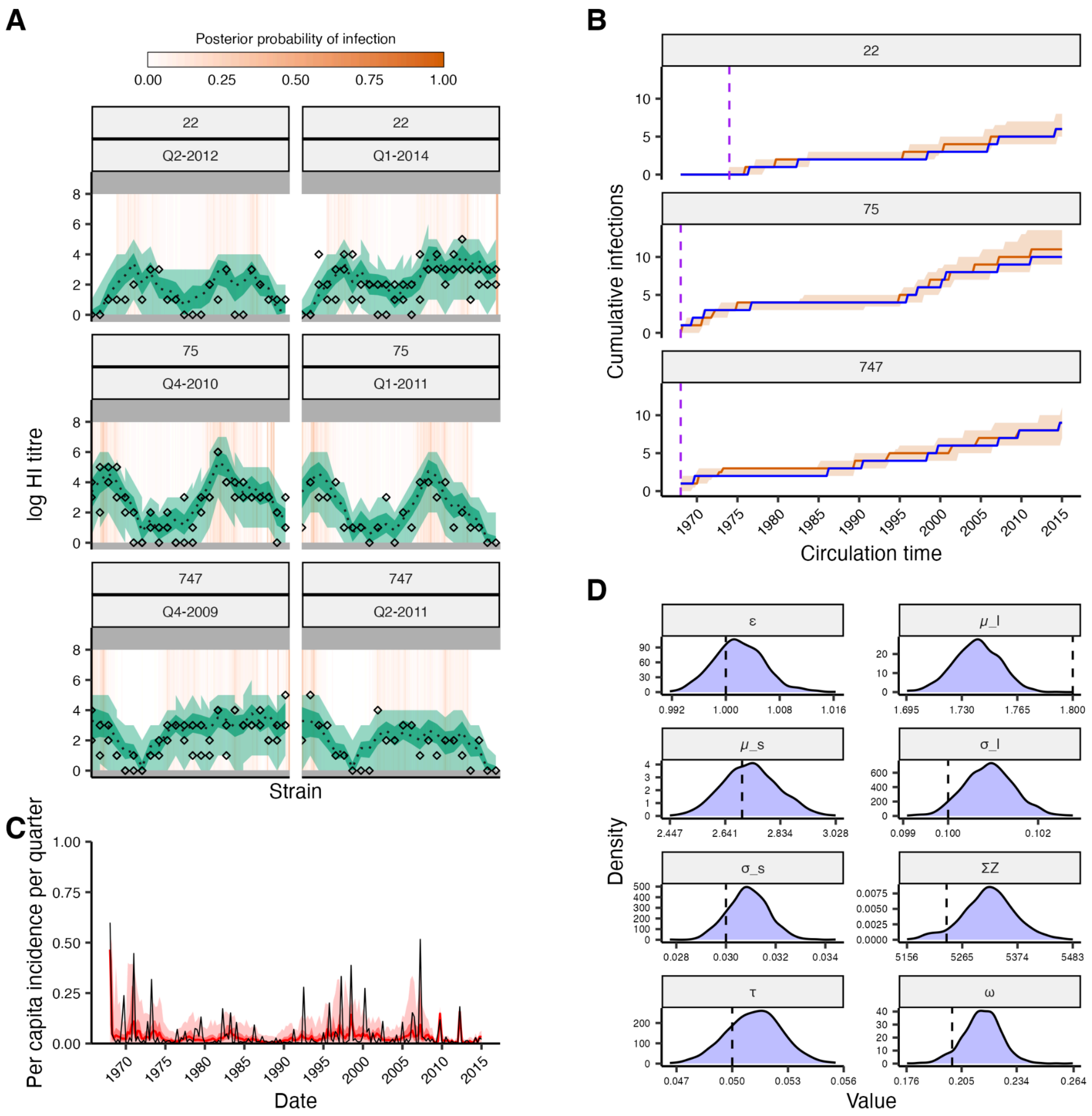


**Figure S25: Assessment of model fitting accuracy based on simulated data.** Results shown are from fitting the full model to simulated infection histories and antibody titres with known parameters. (**A**) Model-predicted titres compared to observed HI titres at each sampling time for three individuals. (**B**) Posterior median and 95% credible intervals (CrI) for the cumulative number of infections over time from birth (orange). Blue solid line shows the true, known cumulative number of infections. Purple dashed line shows the time of birth. (**C**) Posterior estimated per-capita per-3-month attack rates. Red line and shaded region shows posterior median and 95% CrI. Grey line shows the true values used for the simulation. (**D**) Shaded regions show posterior distributions of estimated antibody kinetics parameters. Dashed lines show the true value used for simulation. Note the x-axis range is small relative to the prior ranges in Table S4.

medRxiv preprint doi: https://doi.org/10.1101/2024.03.18.24304371; this version posted April 5, 2024. The copyright holder for this preprint (which was not certified by peer review) is the author/funder, who has granted medRxiv a license to display the preprint in perpetuity.
It is made available under a CC-BY 4.0 International license .

### 2.3.3 Fitting the model ignoring strain-specific measurement offsets

To demonstrate the importance of considering the strain-specific measurement offsets, we compare the model estimates from Section 2.3.2 to results from fitting the model without accounting for the strain-specific measurement offsets, matching the workflow of the main text model. Both versions of the model were able to accurately re-estimate individual-level infection histories and fit to the antibody data well (Fig S25A/B & Fig S26A/B). However, the version without the measurement offset term led to biased attack rate estimates in some time periods (Fig S26C, e.g., 1969, 1975-1980, 1995-2000, 2005-2010). Furthermore, the version without the measurement offset terms led to greater bias in the estimated antibody kinetics parameters, particularly the observation error parameter, $\varepsilon$ (Fig S26D). Overall, these results demonstrate that attempting to account for strain-specific measurement biases, even if incompletely, leads to parameter estimates which are closer to the true values than if these biases are ignored.

medRxiv preprint doi: https://doi.org/10.1101/2024.03.18.24304371; this version posted April 5, 2024. The copyright holder for this preprint (which was not certified by peer review) is the author/funder, who has granted medRxiv a license to display the preprint in perpetuity.
It is made available under a CC-BY 4.0 International license .

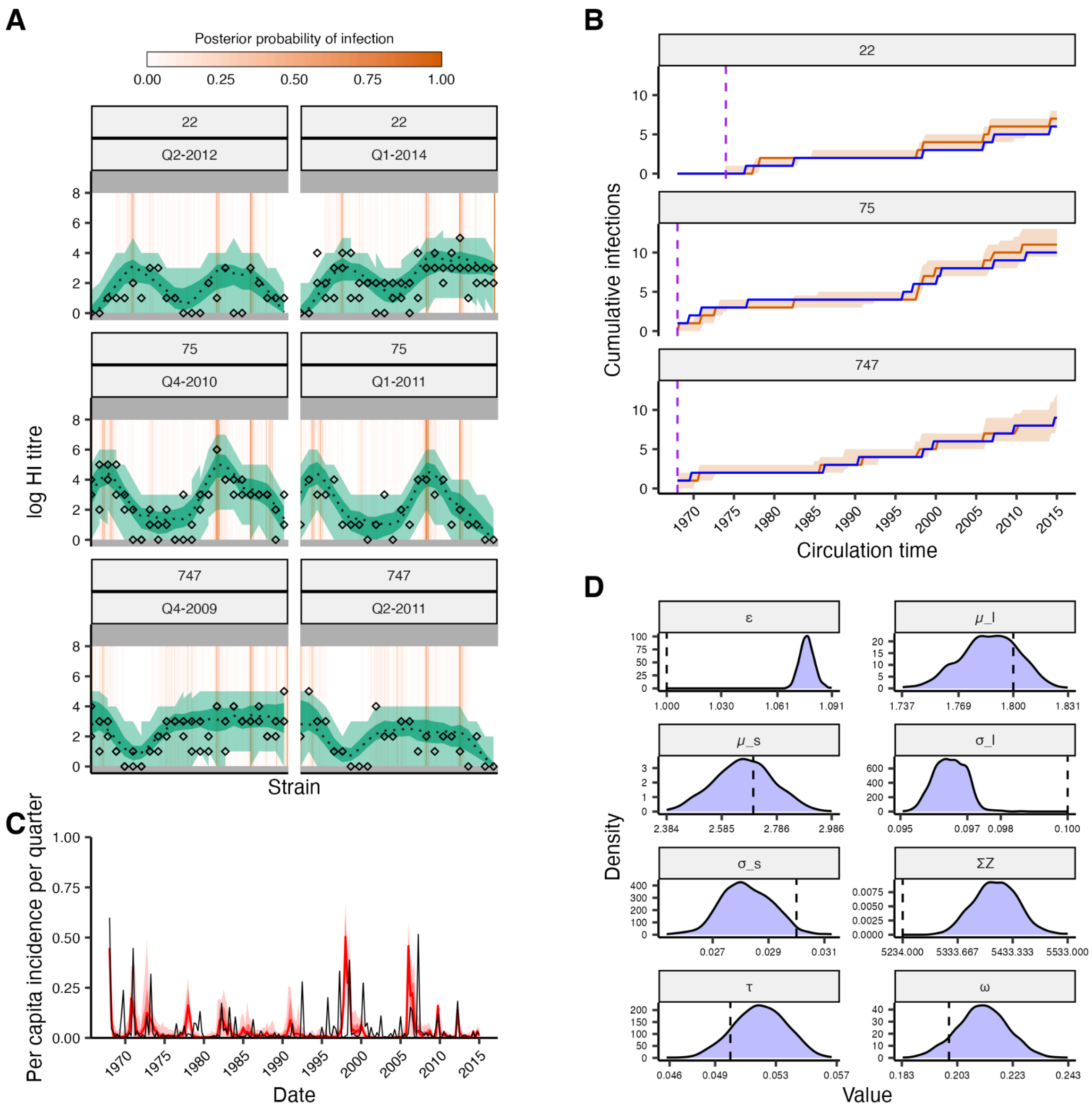


**Figure S26: Assessment of model fitting accuracy based on simulated data when strain-specific measurement offsets are ignored.** Results shown are from fitting the full model to simulated infection histories and antibody titres with known parameters where strain-specific measurement offsets are used in the simulation, but ignored in the fitted model. (**A**) Model-predicted titres compared to observed HI titres at each sampling time for three individuals. (**B**) Posterior median and 95% credible intervals (CrI) for the cumulative number of infections over time from birth (orange). Blue solid line shows the true, known cumulative number of infections. Purple dashed line shows the time of birth. (**C**) Posterior estimated per-capita per-3-month attack rates. Red line and shaded region shows posterior median and 95% CrI. Grey line shows the true values used for the simulation. (**D**) Shaded regions show posterior distributions of estimated antibody kinetics parameters. Dashed lines show the true value used for simulation. Note the x-axis range is small relative to the prior ranges in Table S5.

medRxiv preprint doi: https://doi.org/10.1101/2024.03.18.24304371; this version posted April 5, 2024. The copyright holder for this preprint (which was not certified by peer review) is the author/funder, who has granted medRxiv a license to display the preprint in perpetuity.
It is made available under a CC-BY 4.0 International license .

### 2.3.6 Misspecifying the antigenic map

We do not know the strains which each individual was potentially exposed to, only the most likely antigenic cluster circulating in each time period. Thus, an individual's infection history inferred using *serosolver* depends on the assumed strain an individual could be infected with in each time period, and how it contributes cross-reactive antibodies to their antibody profile. In theory, we might jointly estimate the antigenic map coordinates alongside the other model parameters, but at present this is computationally infeasible and thus we assume a fixed antigenic map for model fitting. As our estimates rely on the assumed position of each strain on the antigenic map, we performed sensitivity analyses to test how our estimates are affected by misspecifying the antigenic map.

First, we refit the model as described in Section 2.3.1 to the same simulated dataset, but instead of using the antigenic map used to simulate the data, we used an alternative antigenic map produced by Bedford et al for model fitting [90] (comparison in Fig S27). As with the antigenic map used in the main text, to generate a single antigenic position representing each strain we fitted a cubic smoothing spline through the antigenic coordinates of all strains on the map (here, smoothing parameter = 0.8). This scenario tests the assumption that the antigenic map used for fitting does not match the antigenic map underlying the data generating process. Parameter estimates, infection histories and attack rates were all largely accurate despite using the wrong antigenic map, though the timing of some elevated attack rates were slightly off (e.g., 1973, 1992, 2007). There were some small biases in the long-term boosting, cross-reactivity and observation error parameters (Fig S28). This suggests that although our quantitative estimates are affected by the assumed antigenic map, the overall trends are largely preserved.

medRxiv preprint doi: https://doi.org/10.1101/2024.03.18.24304371; this version posted April 5, 2024. The copyright holder for this preprint (which was not certified by peer review) is the author/funder, who has granted medRxiv a license to display the preprint in perpetuity.
It is made available under a CC-BY 4.0 International license .

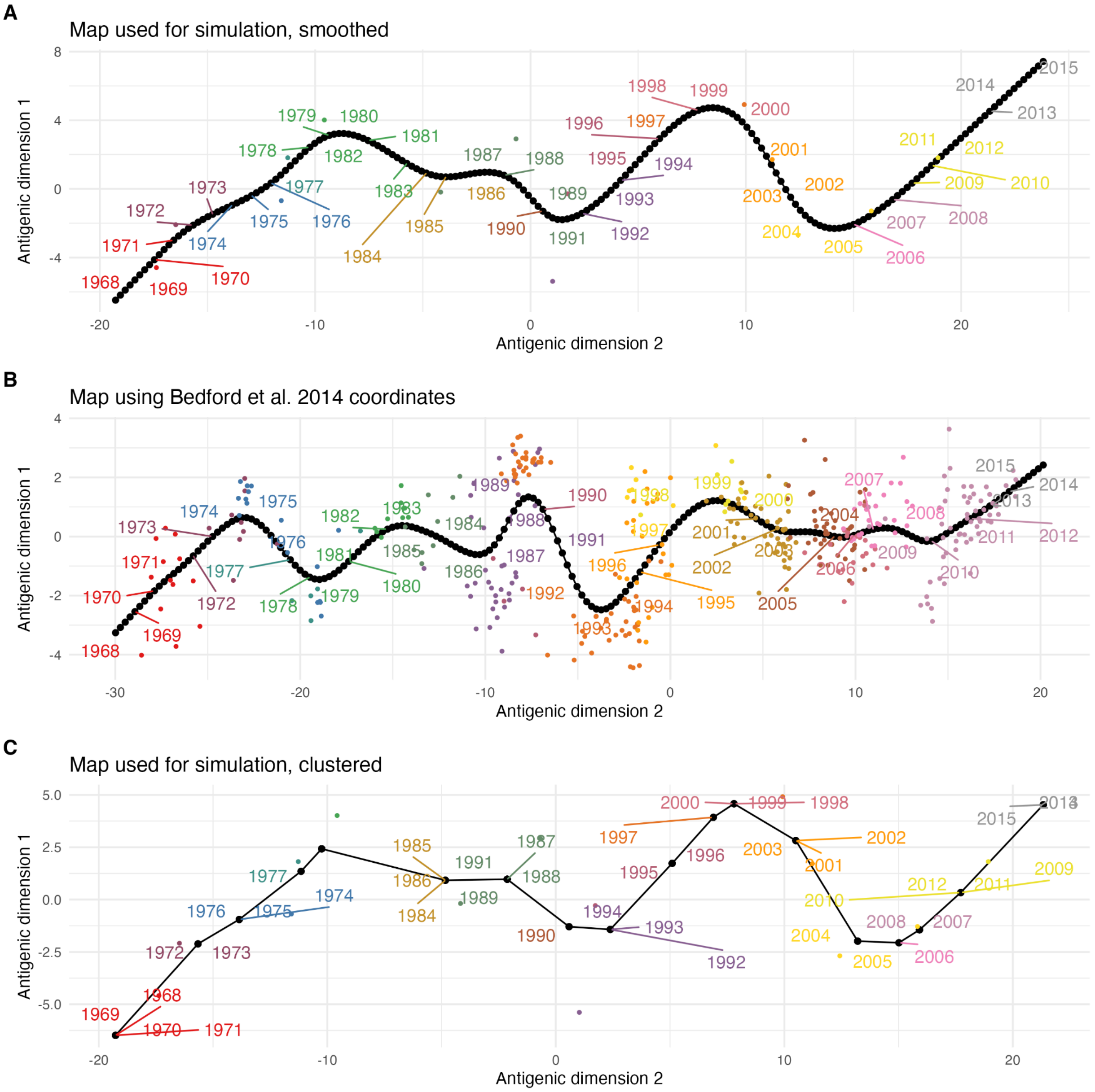


**Figure S27: Antigenic maps used for the simulation (A) compared to the map produced using data from Bedford et al. (B), and the map which assumes a punctuated path through antigenic space (C).** Axes show arbitrary antigenic dimensions. Coloured points show the position of individual strains – all labels of the same color correspond to the same antigenic cluster. Black line shows the fitted antigenic summary path for each map. Black dots show the antigenic position of the strain corresponding to each time period; (**A**) and (**B**) assume continuous evolution through antigenic space whereas (**C**) assumes that the same position is used for all strains within a cluster.

medRxiv preprint doi: https://doi.org/10.1101/2024.03.18.24304371; this version posted April 5, 2024. The copyright holder for this preprint
(which was not certified by peer review) is the author/funder, who has granted medRxiv a license to display the preprint in perpetuity.
It is made available under a CC-BY 4.0 International license .

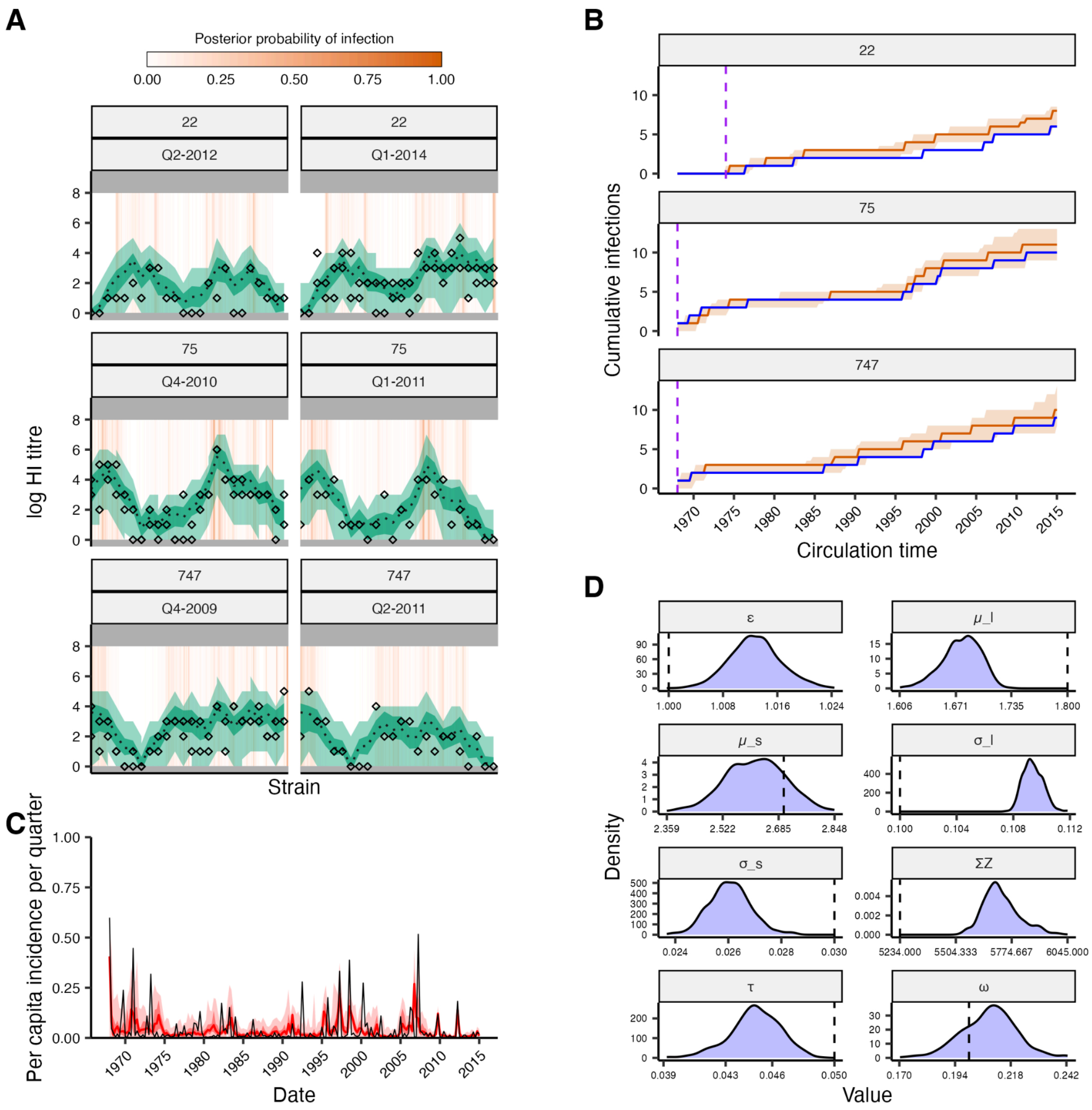


**Fig S28: Assessment of model fitting accuracy when the antigenic map used for model fitting does not match the one used for simulation.** Results shown are from fitting the full model to simulated infection histories and antibody titres with known parameters. (**A**) Model-predicted titres compared to observed HI titres at each sampling time for three individuals. (**B**) Posterior median and 95% credible intervals (CrI) for the cumulative number of infections over time from birth (orange). Blue solid line shows the true, known cumulative number of infections. Purple dashed line shows the time of birth. (**C**) Posterior estimated per-capita per-3-month attack rates. Red line and shaded region shows posterior median and 95% CrI. Grey line shows the true values used for the simulation. (**D**) Shaded regions show posterior distributions of estimated antibody kinetics parameters. Dashed lines show the true value used for simulation. Note the x-axis range is small relative to the prior ranges in Table S4.

medRxiv preprint doi: https://doi.org/10.1101/2024.03.18.24304371; this version posted April 5, 2024. The copyright holder for this preprint (which was not certified by peer review) is the author/funder, who has granted medRxiv a license to display the preprint in perpetuity.
It is made available under a CC-BY 4.0 International license .

Second, we generated an alternative simulated dataset using an antigenic map where antigenic evolution was assumed to follow punctuated jumps between clusters with all strains belonging to the same cluster having the same antigenic coordinates matching the first strain isolated from each cluster (Fig S27C), and then refit the model described in Section 2.3.1 using the smoothed antigenic map rather than the clustered map used for the simulation. This scenario tests the impact of our assumption that sequential strains follow a smooth and continuous path through antigenic space despite the true data generating process following punctuated jumps between clusters. We used the estimates from Du et al. to determine the time periods of cluster dominance – Du et al. predicted which A/H3N2 antigenic cluster likely circulated in China in each time period based on HA sequence data. For example, where Du et al. present a time range e.g., BE92 (1992-1995), we assumed that cluster circulated up to and not including the final time point in the range e.g., assume that BE92 circulated from January 1992 up to and including December 1994. We do not have cluster information after the PE09 cluster, and thus we assumed a new cluster emerged in 2013 (i.e., the PE09 cluster dominated for 4 years). Du et al. demonstrate that clusters are almost entirely dominant in the period in which they are circulating, though they rarely reach 100% frequency.

Parameter, infection history and attack rate estimates were mostly close to their true values despite misspecifying the antigenic map, with some differences in the estimated attack rates. Infection histories and model fits matched their true values well (Fig S29A&B), though the timing of elevated attack rates did not always align (Fig S29C). For example, the model missed the high attack rate in 1971, likely attributing these infections to the consecutive high attack rate periods from 1968. The high attack rates in 1992 and 2007 were also slightly misaligned, though the model was able to detect high incidence around those time periods. This misalignment is expected given how the antigenic map was misspecified. In the clustered map, we assumed that all strains in a cluster had the antigenic coordinates matching the first strain. However, in the smoothed map, each strain was instead assumed to be antigenically different from the previous one. Thus, when fitting with the smooth map, the model tends to attribute infections in a cluster to the first strain, as its antigenic coordinates are the closest to their true values used in the simulation. This tends to shift attack rates in those cluster periods earlier. Reassuringly, the model was still able to accurately recover the total number of infections in the simulation.

Some of the antibody kinetics parameters were slightly biased, with notable overestimation of the observation error parameter and waning rate, and slight underestimation of the long-term

medRxiv preprint doi: https://doi.org/10.1101/2024.03.18.24304371; this version posted April 5, 2024. The copyright holder for this preprint (which was not certified by peer review) is the author/funder, who has granted medRxiv a license to display the preprint in perpetuity.
It is made available under a CC-BY 4.0 International license .

cross-reactivity parameter (Fig S29D). Bias in the waning rate parameter estimates is unsurprising given that attack rates were estimated to be slightly later than the truth for recent time periods which provide the model with information on these short-term kinetics. If infections are estimated later than their true timing, then a higher waning rate is required to reach the same low titres in a shorter time since infection. Bias in the observation error parameter is also unsurprising, as bias from model misspecification in the measurement offset term estimation stage (Section 2.3.1) becomes compounded when fitting the full model.

medRxiv preprint doi: https://doi.org/10.1101/2024.03.18.24304371; this version posted April 5, 2024. The copyright holder for this preprint (which was not certified by peer review) is the author/funder, who has granted medRxiv a license to display the preprint in perpetuity.
It is made available under a CC-BY 4.0 International license .

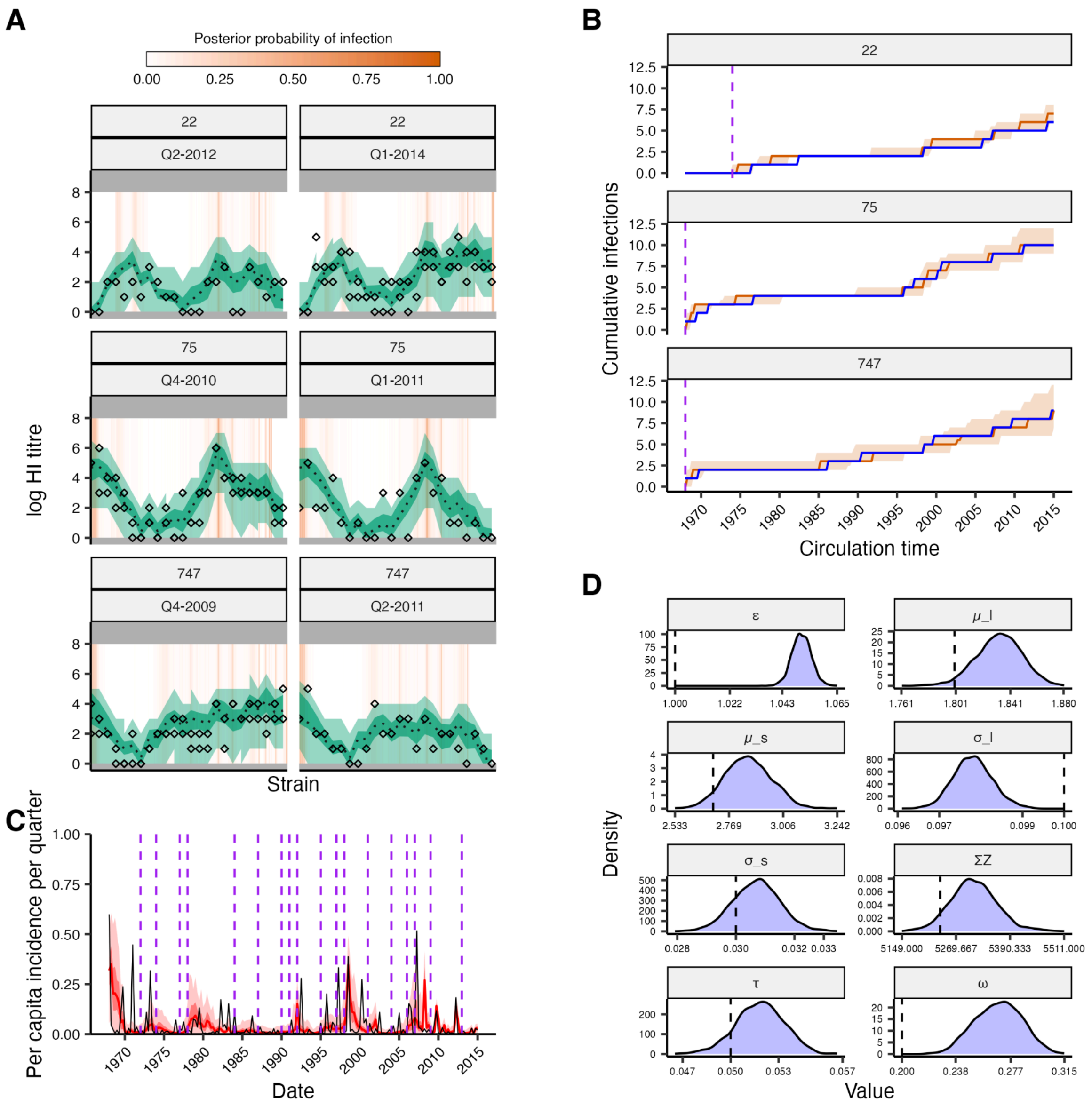


**Fig S29: Assessment of model fitting accuracy based when fitting a model with a smoothed antigenic map to data simulated using a punctuated antigenic map.** (**A**) Model-predicted titres compared to observed HI titres at each sampling time for three individuals. (**B**) Posterior median and 95% credible intervals (CrI) for the cumulative number of infections over time from birth (orange). Blue solid line shows the true, known cumulative number of infections. Purple dashed line shows the time of birth. (**C**) Posterior estimated per-capita per-3-month attack rates. Red line and shaded region shows posterior median and 95% CrI. Grey line shows the true values used for the simulation. Purple dashed lines show cluster transitions. (**D**) Shaded regions show posterior distributions of estimated antibody kinetics parameters. Dashed lines show the true value used for simulation. Note the x-axis range is small relative to the prior ranges in Table S5.

medRxiv preprint doi: https://doi.org/10.1101/2024.03.18.24304371; this version posted April 5, 2024. The copyright holder for this preprint (which was not certified by peer review) is the author/funder, who has granted medRxiv a license to display the preprint in perpetuity.
It is made available under a CC-BY 4.0 International license .

<< Separate file >>

**Supplementary Material 3: Distribution of quarterly attack rates by location over time.** Each colored point shows the inferred attack rate in each of the 40 locations, with size and shading reflecting the posterior median attack rate. Underlying the plot is a map of the study area, with each grid cell shaded by its log10 population density.

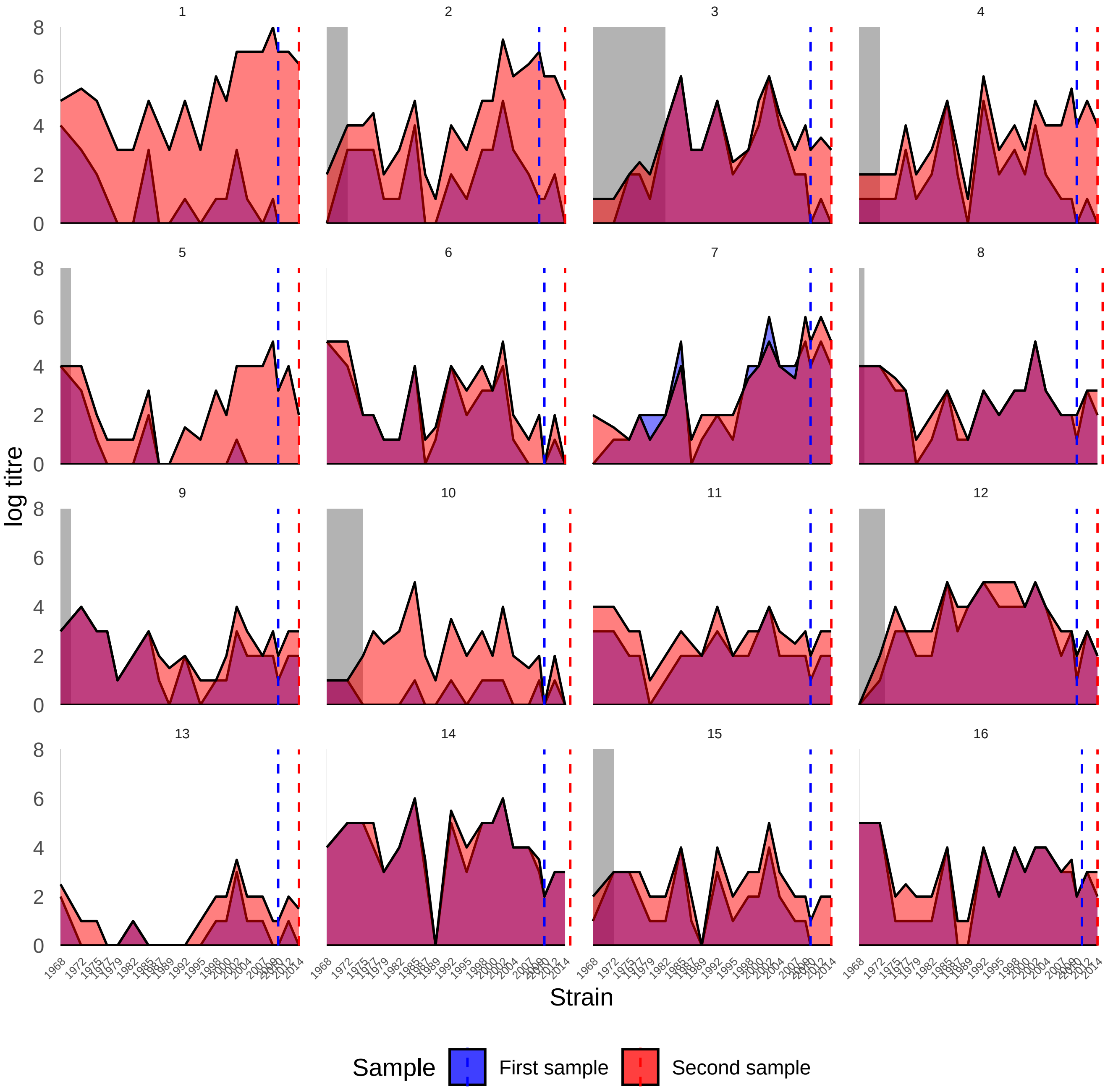

1
2
3
4
5
6
7
8
9
10
11
12
13
14
15
16
log titre
Strain
Sample
First sample
Second sample

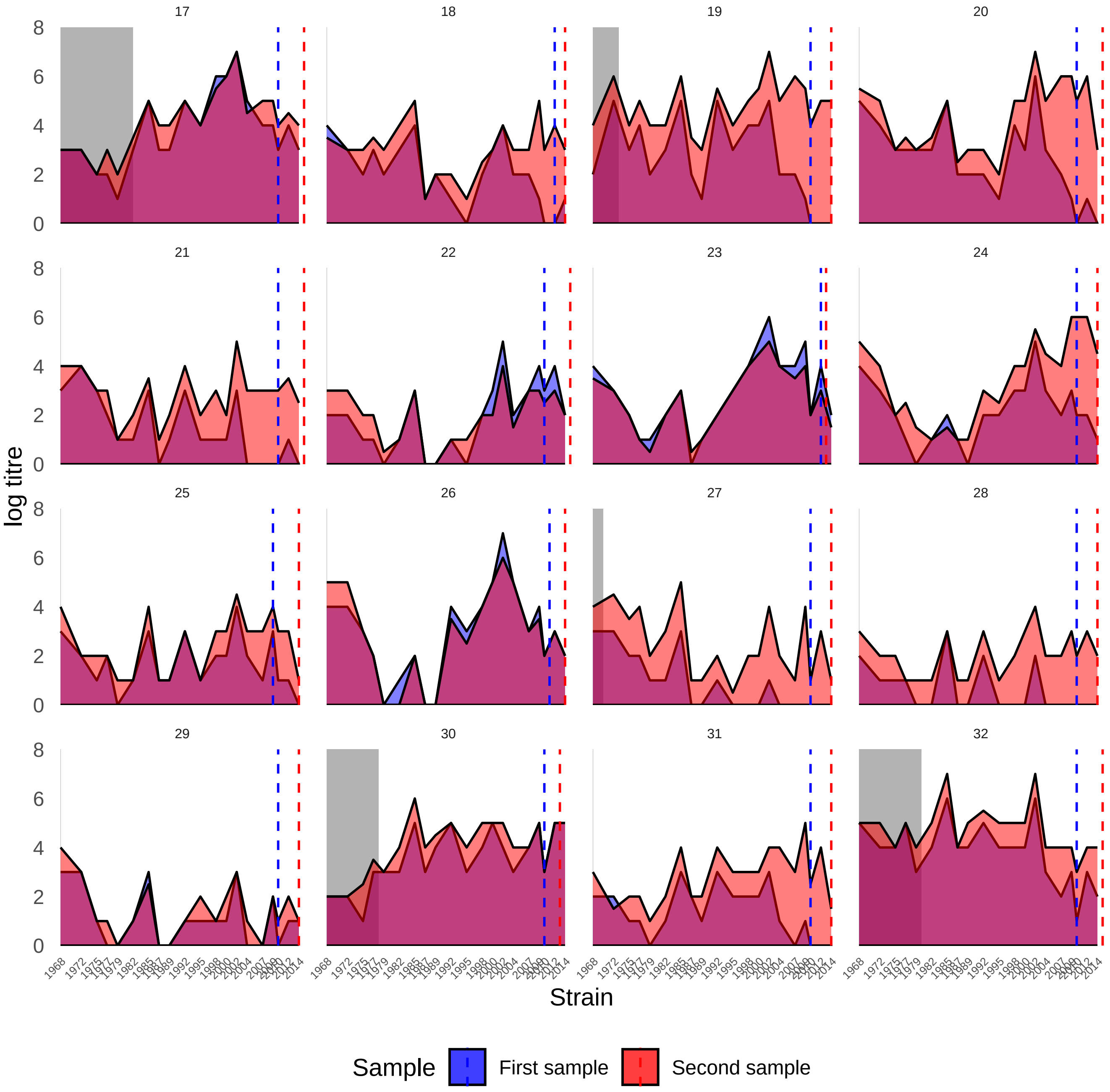

17
18
19
20
21
22
23
24
25
26
27
28
29
30
31
32
log titre
Strain
Sample
First sample
Second sample

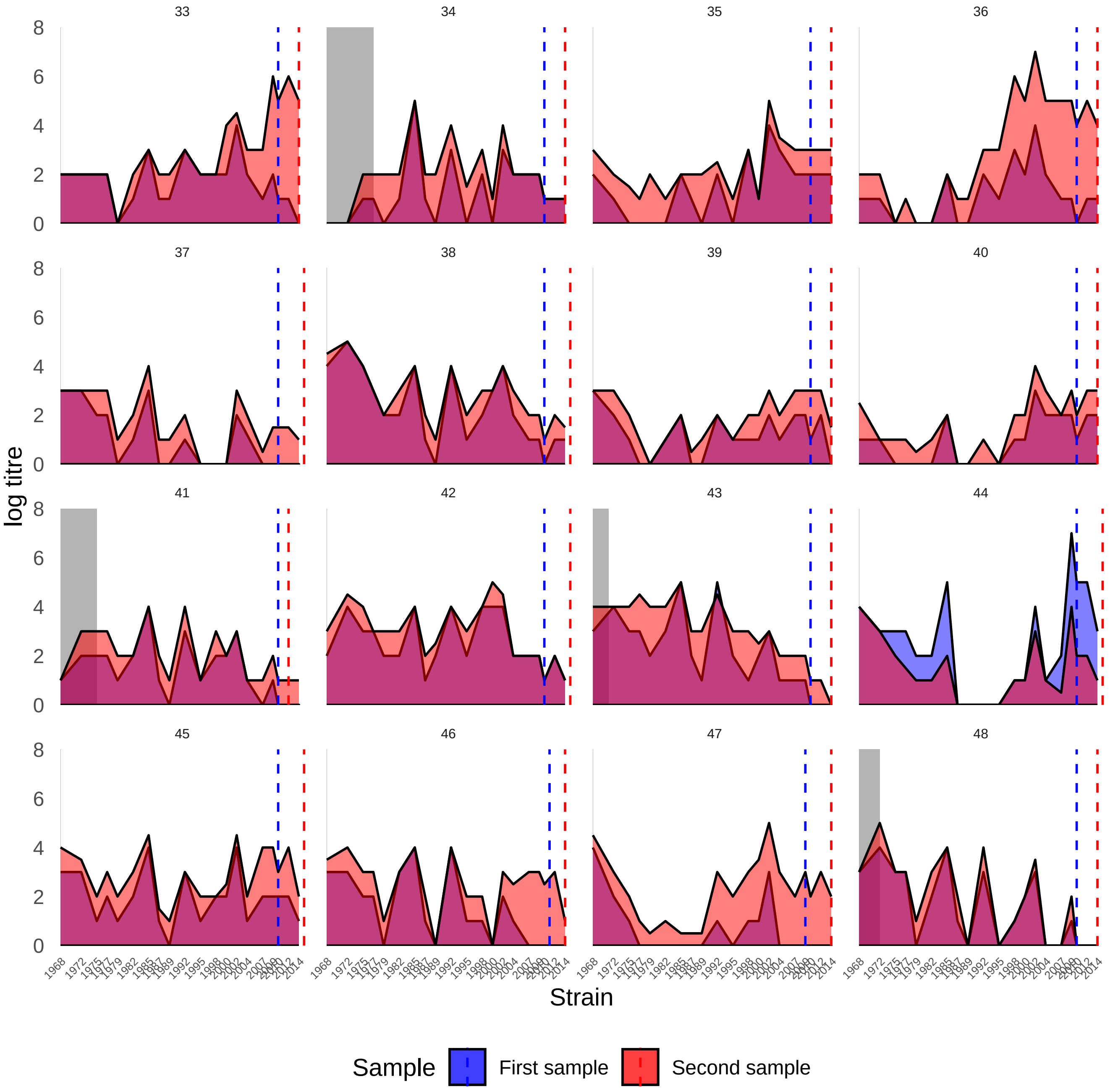
33
34
35
36
37
38
39
40
41
42
43
44
45
46
47
48
log titre
Strain
1968
1972
1975
1977
1979
1982
1985
1987
1989
1992
1995
1998
2000
2002
2004
2007
2009
2010
2012
2014
Sample
First sample
Second sample

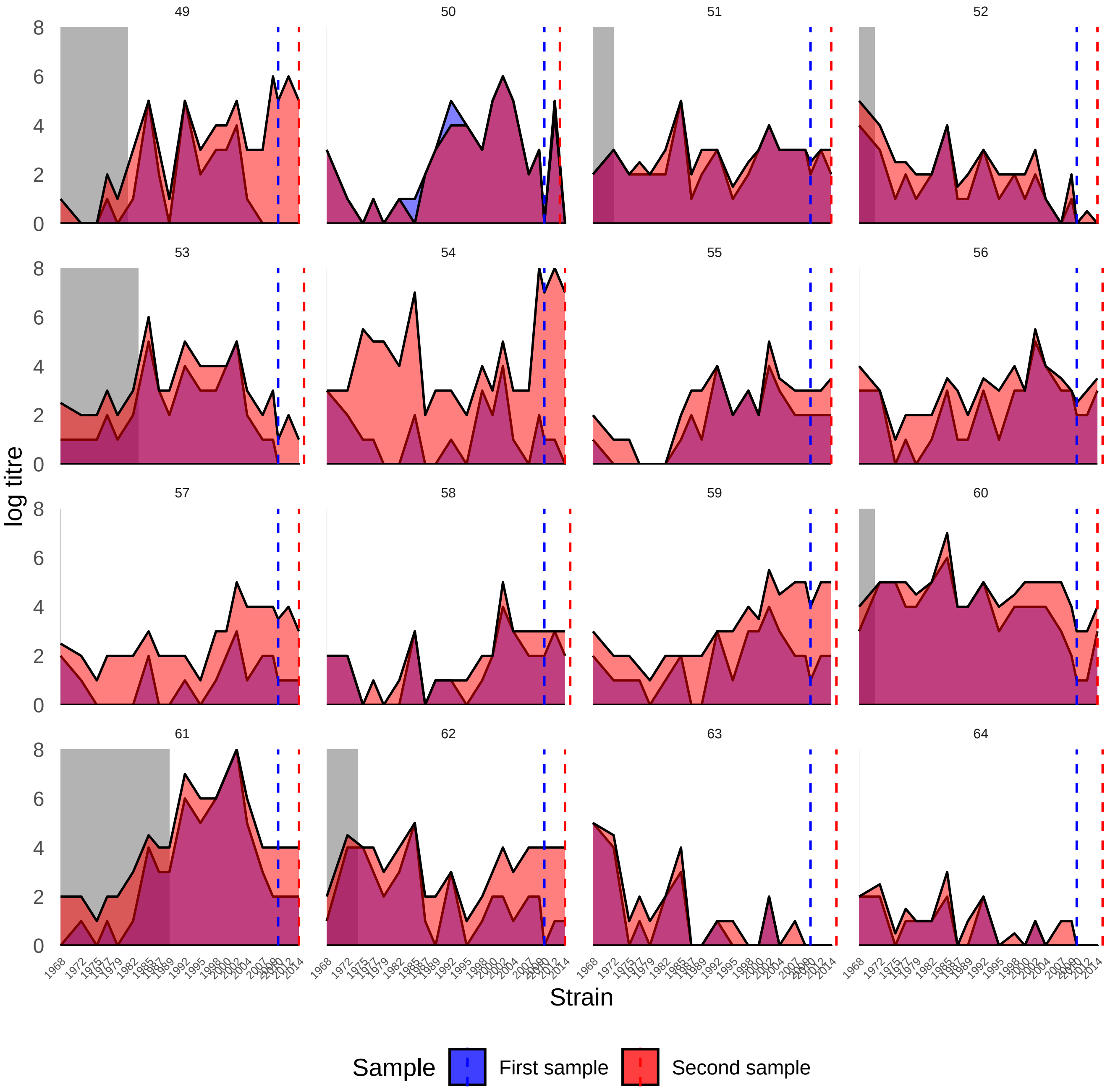

49
50
51
52
53
54
55
56
57
58
59
60
61
62
63
64
log titre
Strain
1968
1972
1975
1977
1979
1982
1985
1987
1989
1992
1995
1998
2000
2002
2004
2007
2009
2010
2012
2014
Sample
First sample
Second sample

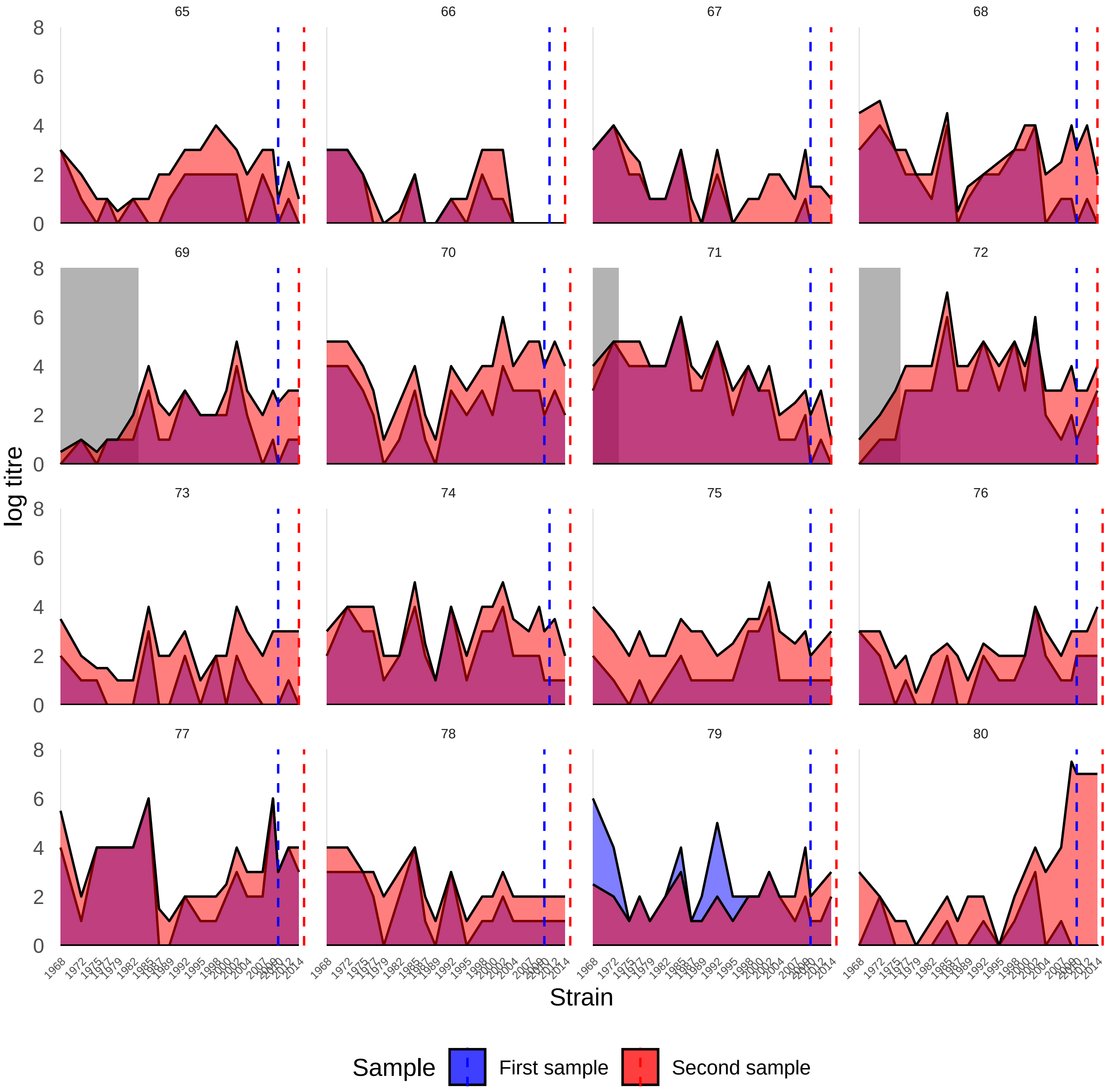

65
66
67
68
69
70
71
72
73
74
75
76
77
78
79
80
log titre
Strain
1968
1972
1975
1977
1979
1982
1985
1987
1989
1992
1995
1998
2000
2002
2004
2007
2009
2010
2012
2014
Sample
First sample
Second sample

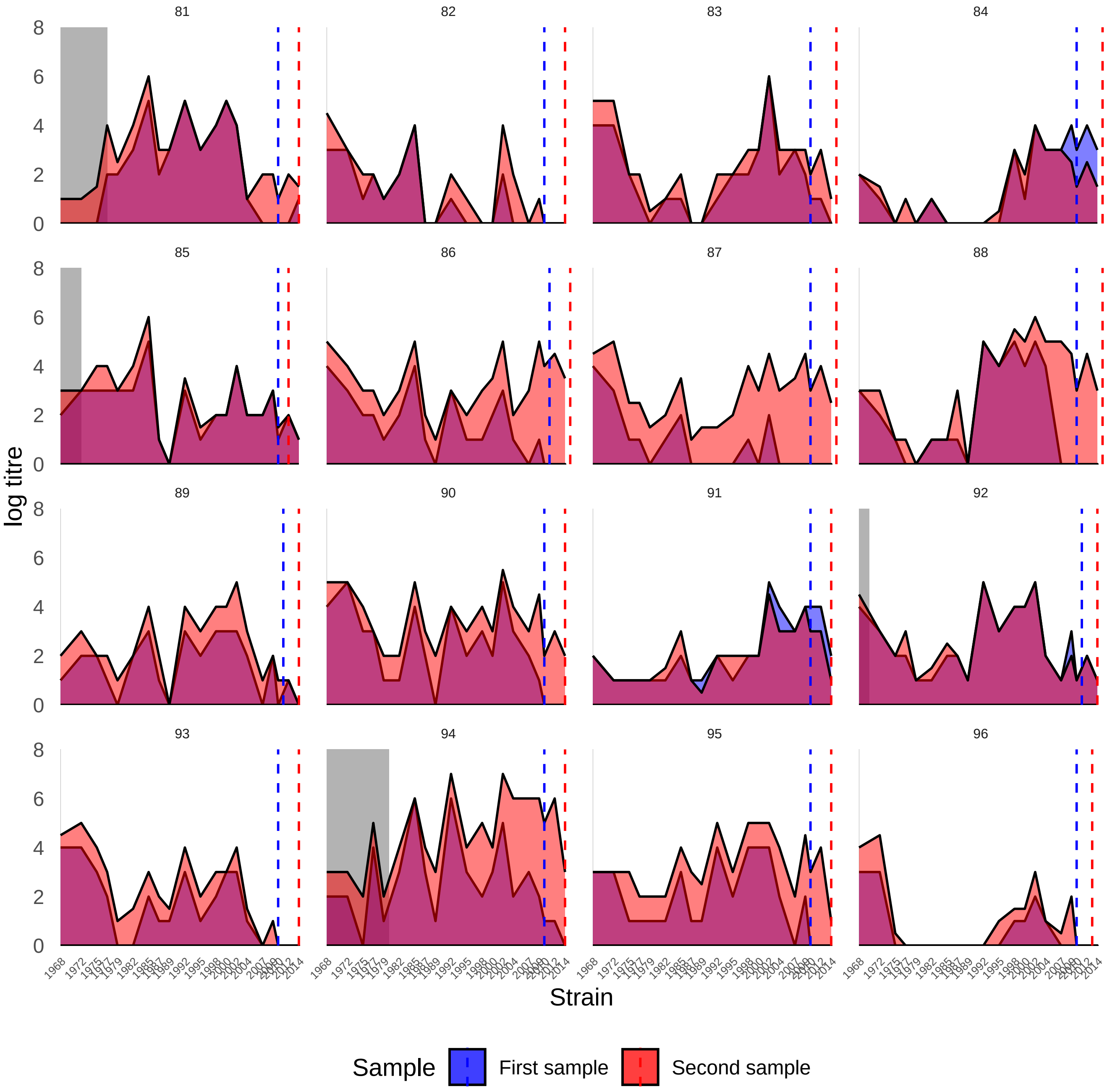

81
82
83
84
85
86
87
88
89
90
91
92
93
94
95
96
log titre
Strain
1968
1972
1975
1977
1979
1982
1985
1987
1989
1992
1995
1998
2000
2002
2004
2007
2009
2010
2012
2014
Sample
First sample
Second sample

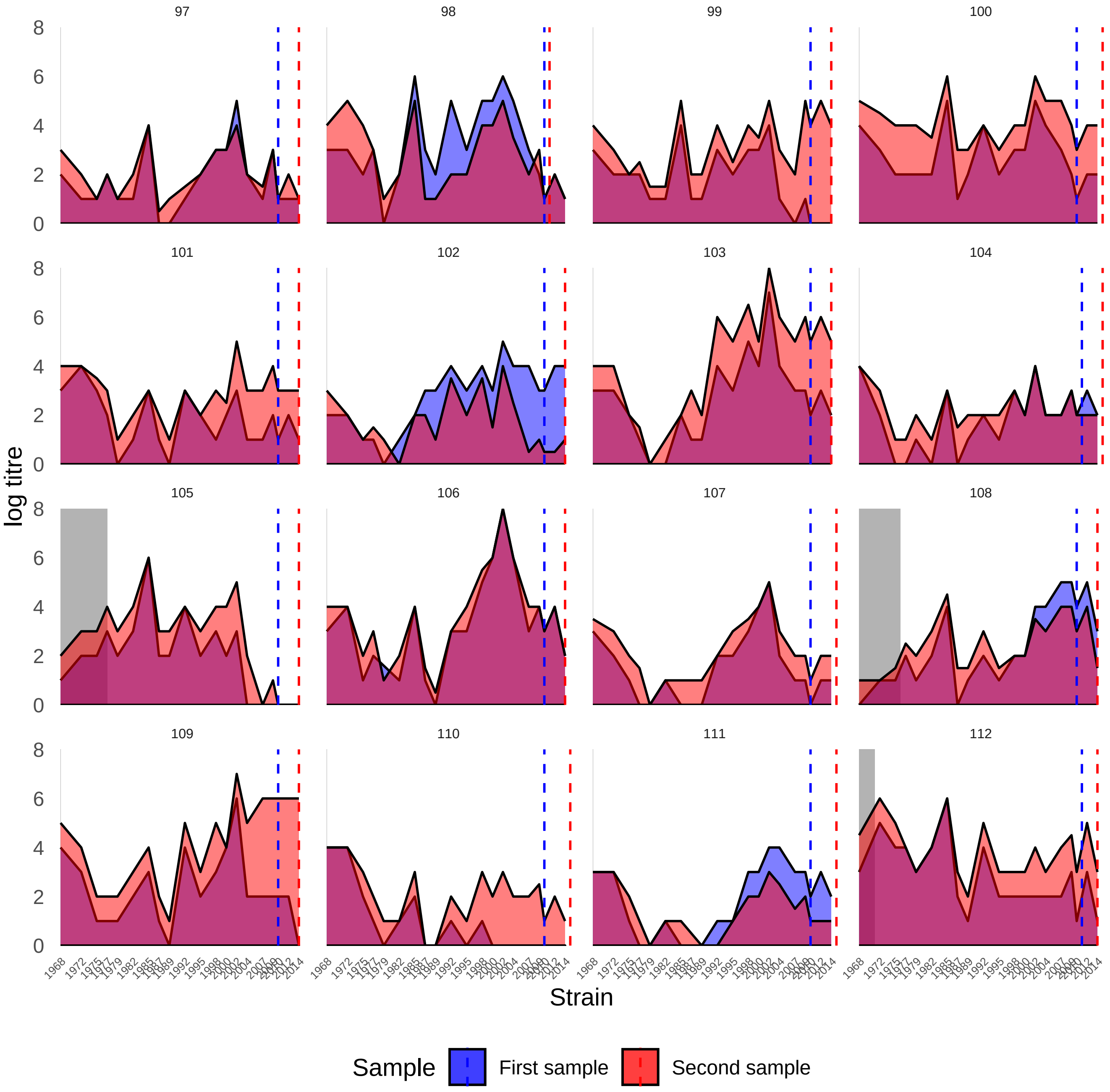

97
98
99
100
101
102
103
104
105
106
107
108
109
110
111
112
log titre
Strain
1968 1972 1975 1977 1979 1982 1985 1987 1989 1992 1995 1998 2000 2002 2004 2007 2009 2010 2012 2014
Sample
First sample
Second sample

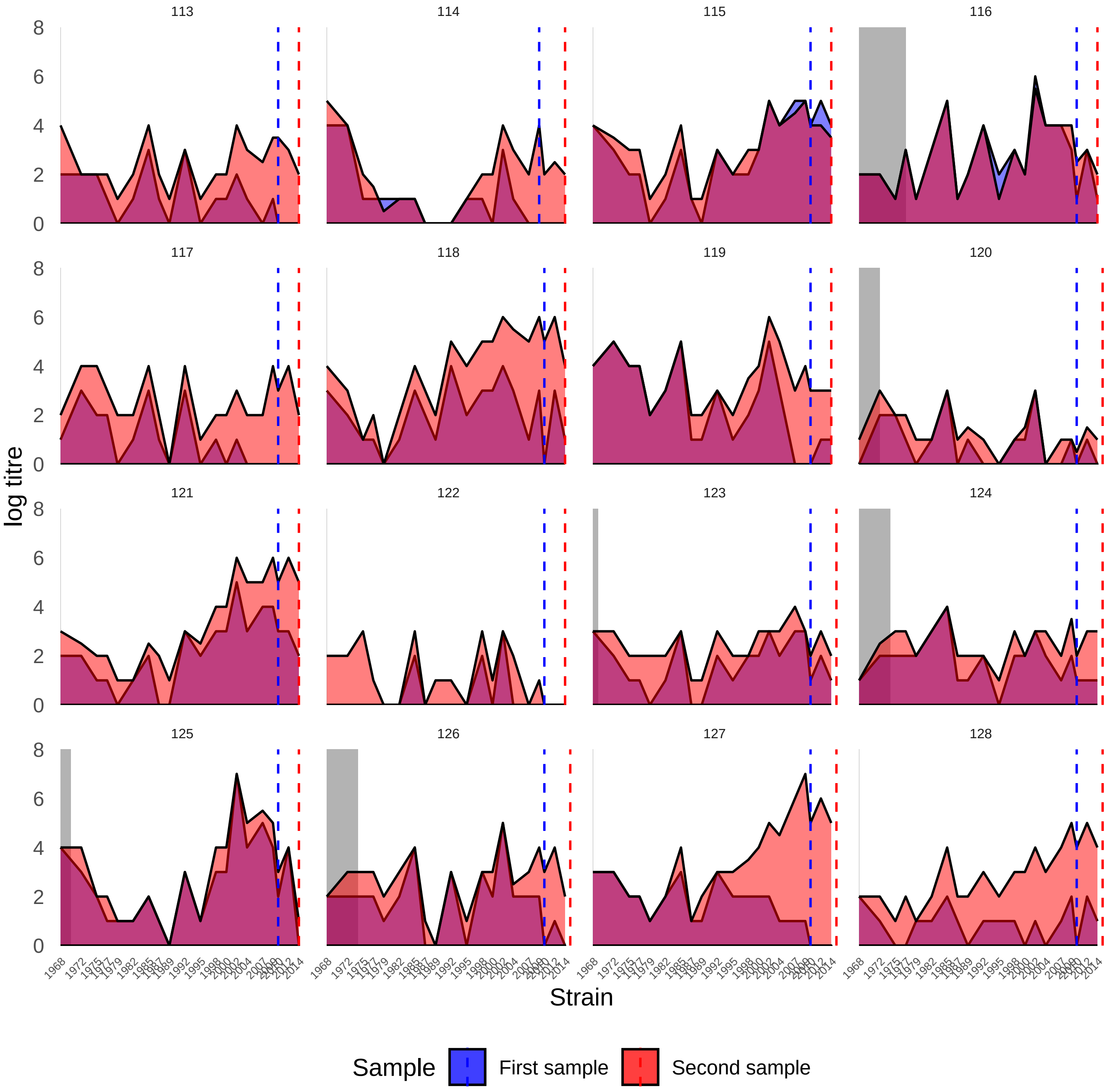

113
114
115
116
117
118
119
120
121
122
123
124
125
126
127
128
log titre
Strain
Sample
First sample
Second sample

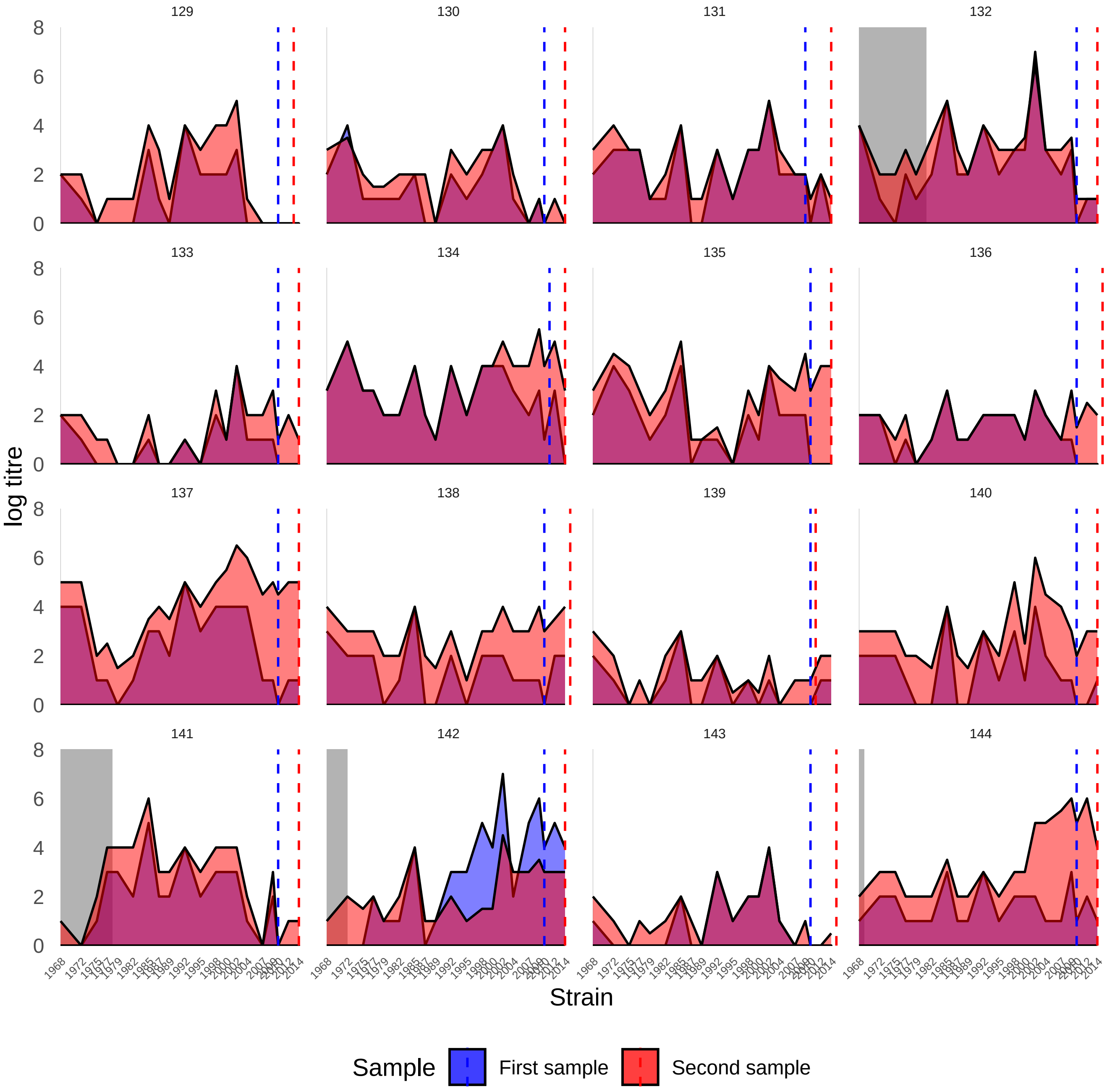
129
130
131
132
133
134
135
136
137
138
139
140
141
142
143
144
log titre
Strain
Sample
First sample
Second sample

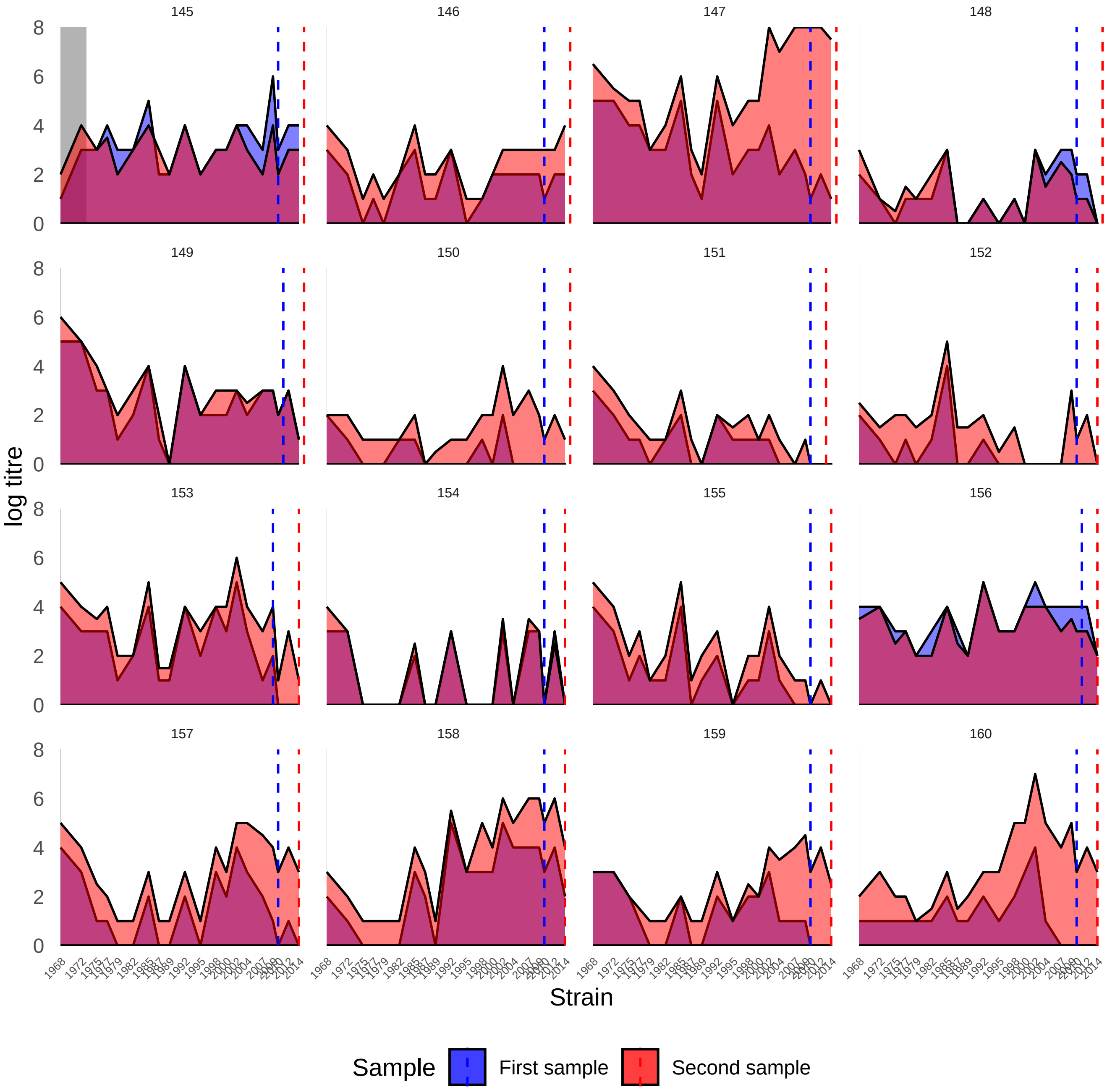

145
146
147
148
149
150
151
152
153
154
155
156
157
158
159
160
log titre
Strain
Sample
First sample
Second sample

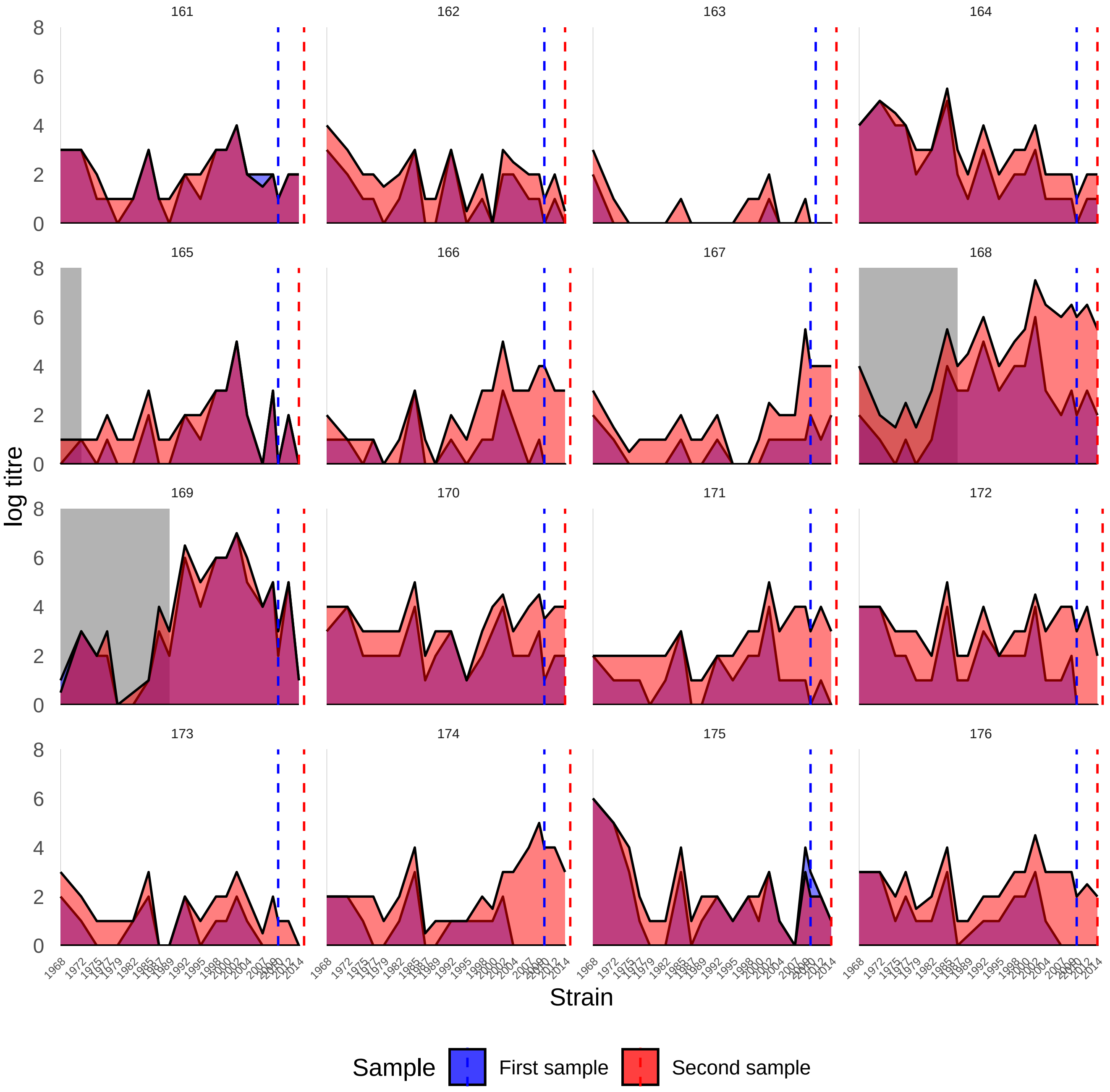
161
162
163
164
165
166
167
168
169
170
171
172
173
174
175
176
log titre
Strain
1968
1972
1975
1977
1979
1982
1985
1987
1989
1992
1995
1998
2000
2002
2004
2007
2009
2010
2012
2014
Sample
First sample
Second sample

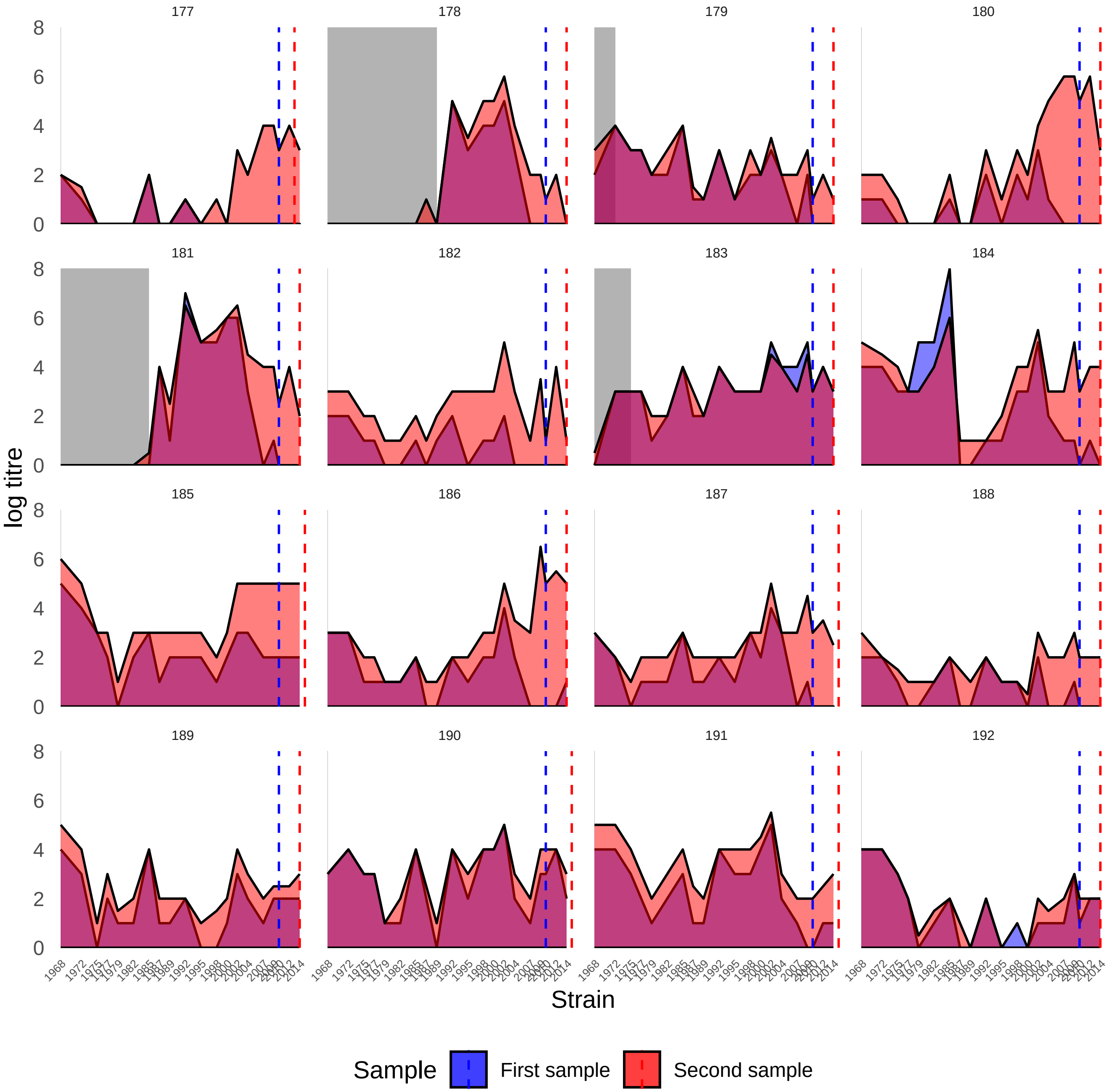
177
178
179
180
181
182
183
184
185
186
187
188
189
190
191
192
log titre
Strain
1968
1972
1975
1977
1979
1982
1985
1987
1989
1992
1995
1998
2000
2002
2004
2007
2009
2012
2014
Sample
First sample
Second sample

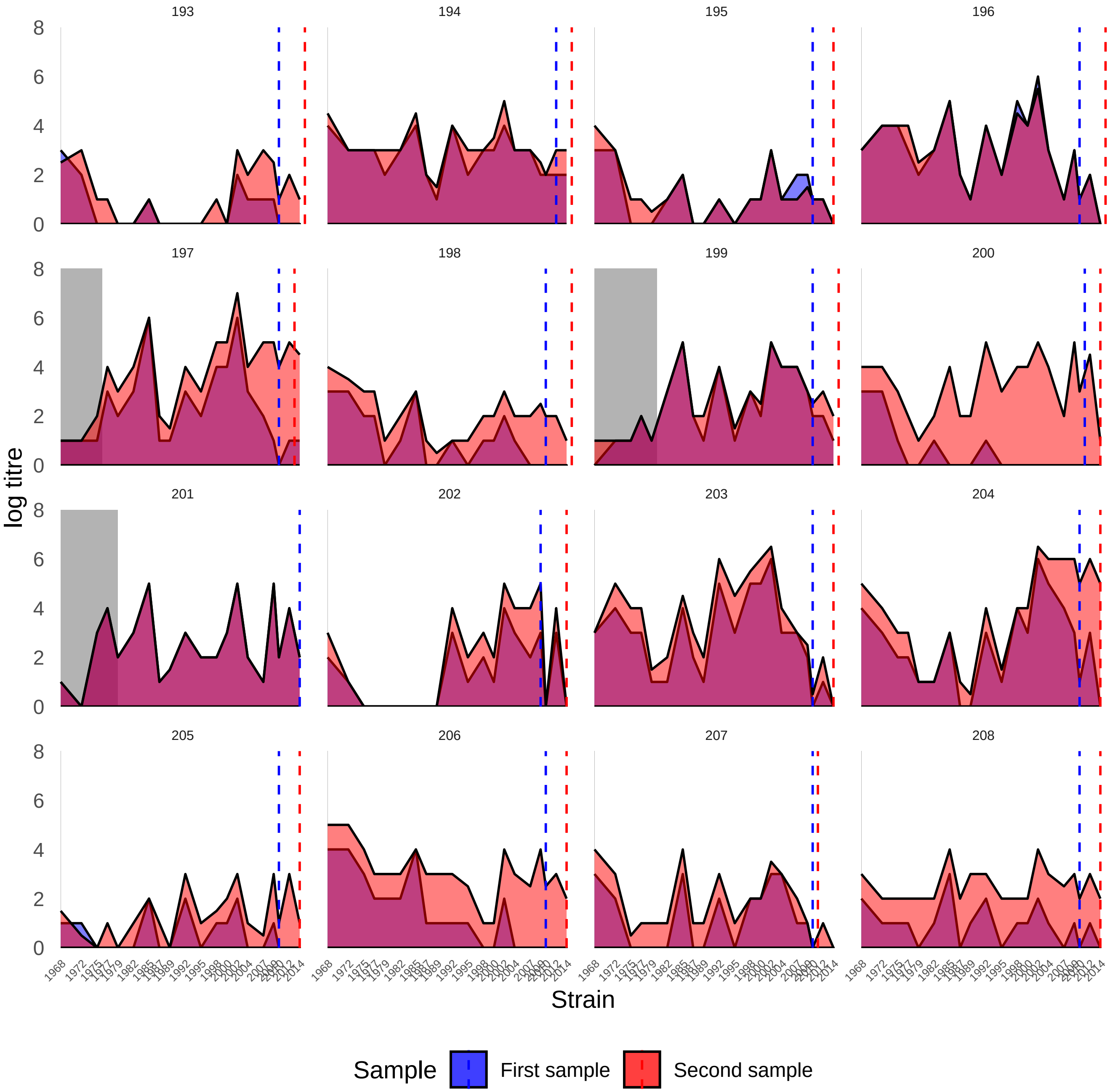

193
194
195
196
197
198
199
200
201
202
203
204
205
206
207
208
log titre
0
2
4
6
8
1968
1972
1975
1977
1979
1982
1985
1987
1989
1992
1995
1998
2000
2002
2004
2007
2009
2010
2012
2014
Strain
Sample
First sample
Second sample

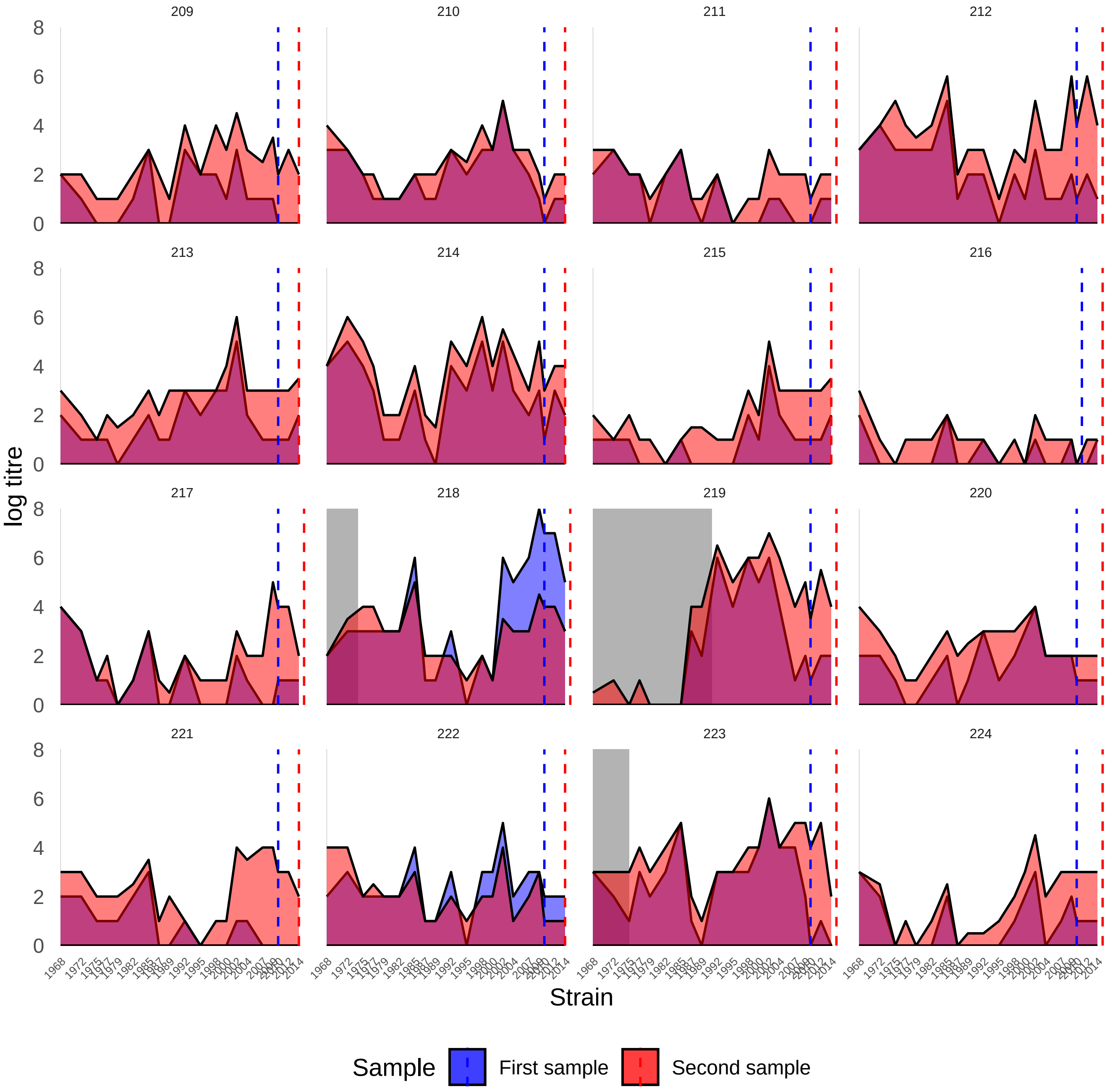
209
210
211
212
213
214
215
216
217
218
219
220
221
222
223
224
log titre
Strain
Sample
First sample
Second sample

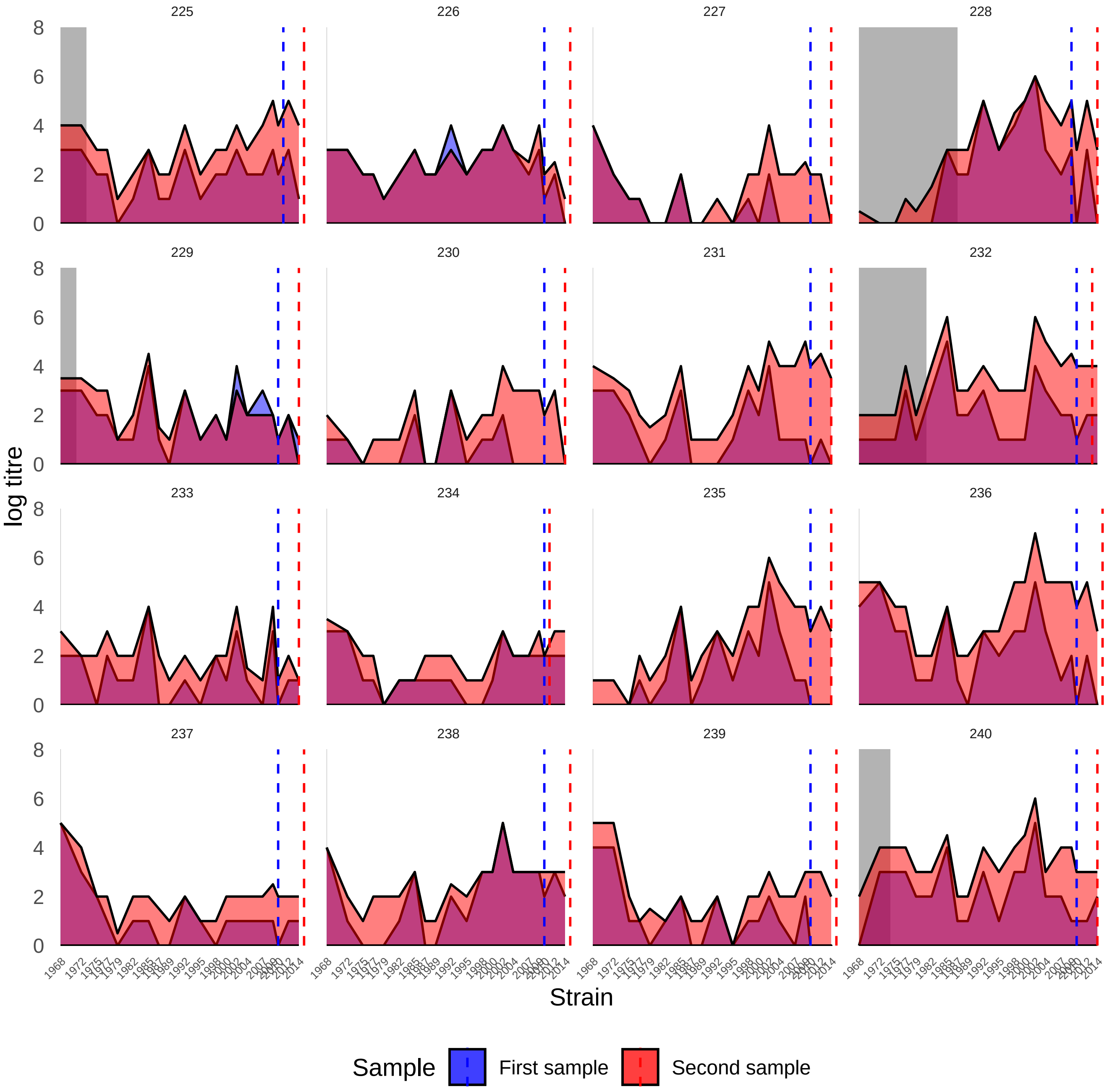

225
226
227
228
229
230
231
232
233
234
235
236
237
238
239
240
log titre
Strain
1968
1972
1975
1977
1979
1982
1985
1987
1989
1992
1995
1998
2000
2002
2004
2007
2009
2010
2012
2014
Sample
First sample
Second sample

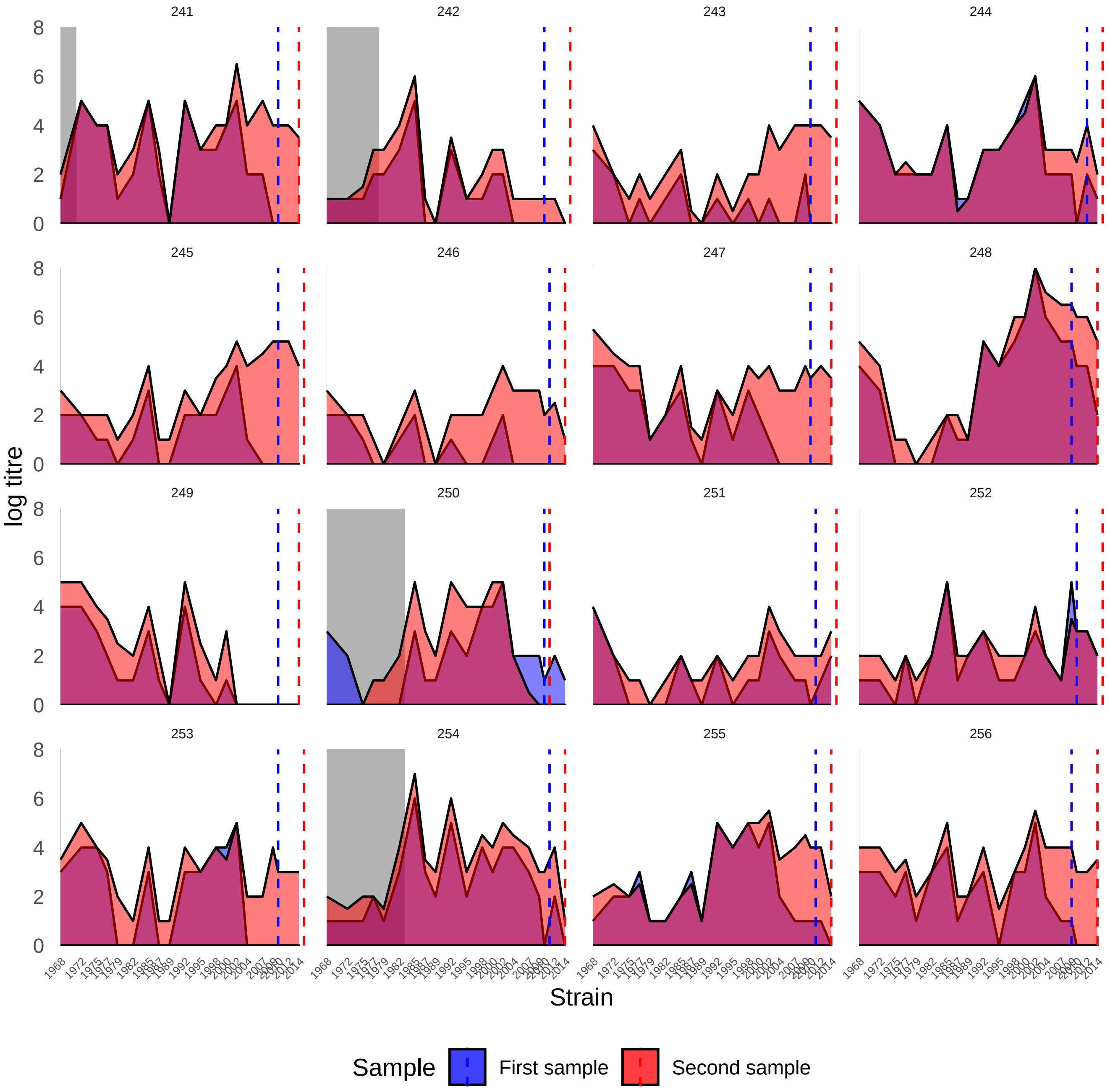

241
242
243
244
245
246
247
248
249
250
251
252
253
254
255
256
log titre
Strain
Sample
First sample
Second sample

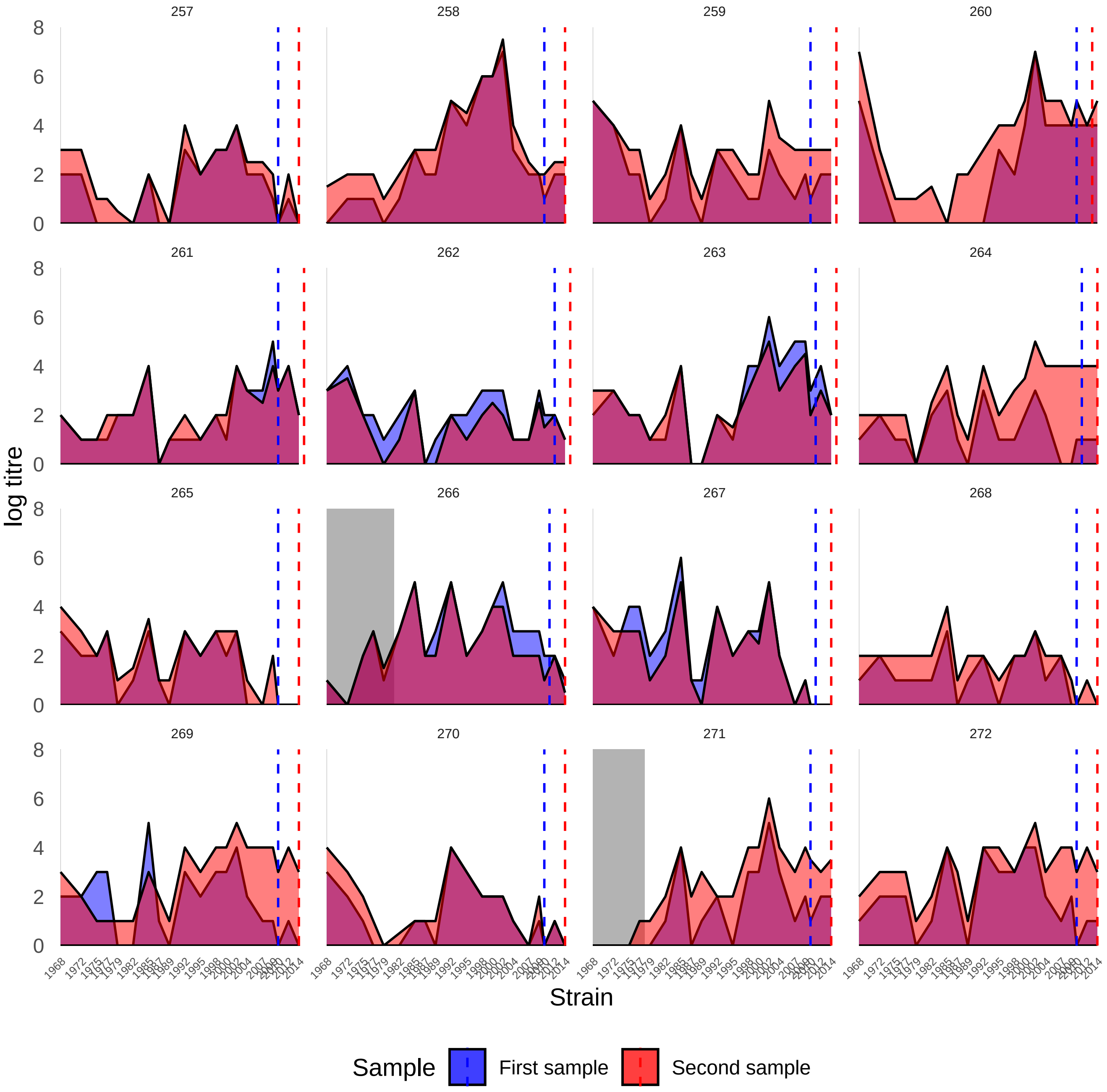

257
258
259
260
261
262
263
264
265
266
267
268
269
270
271
272
log titre
Strain
Sample
First sample
Second sample

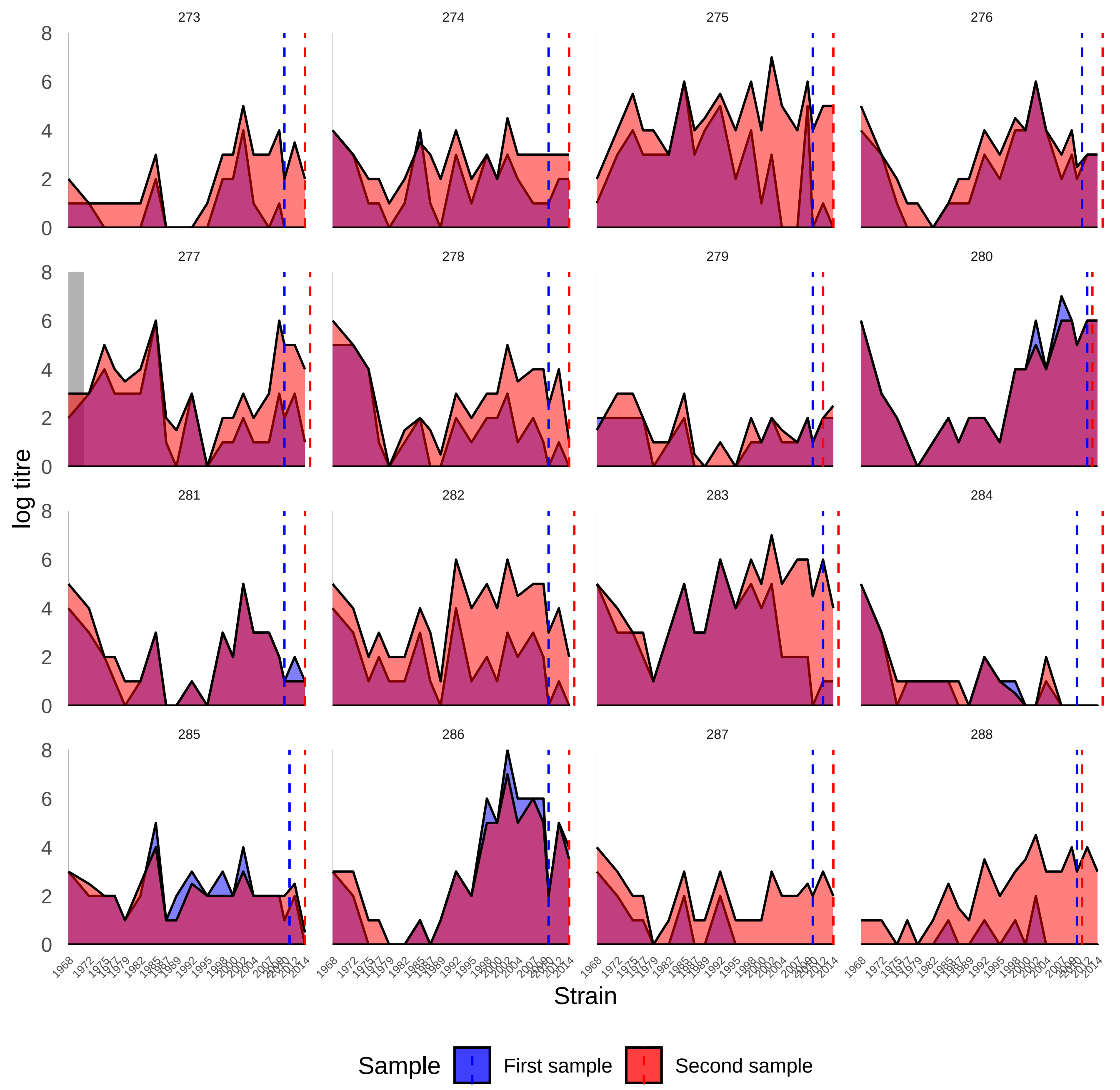

273
274
275
276
277
278
279
280
281
282
283
284
285
286
287
288
log titre
Strain
Sample
First sample
Second sample

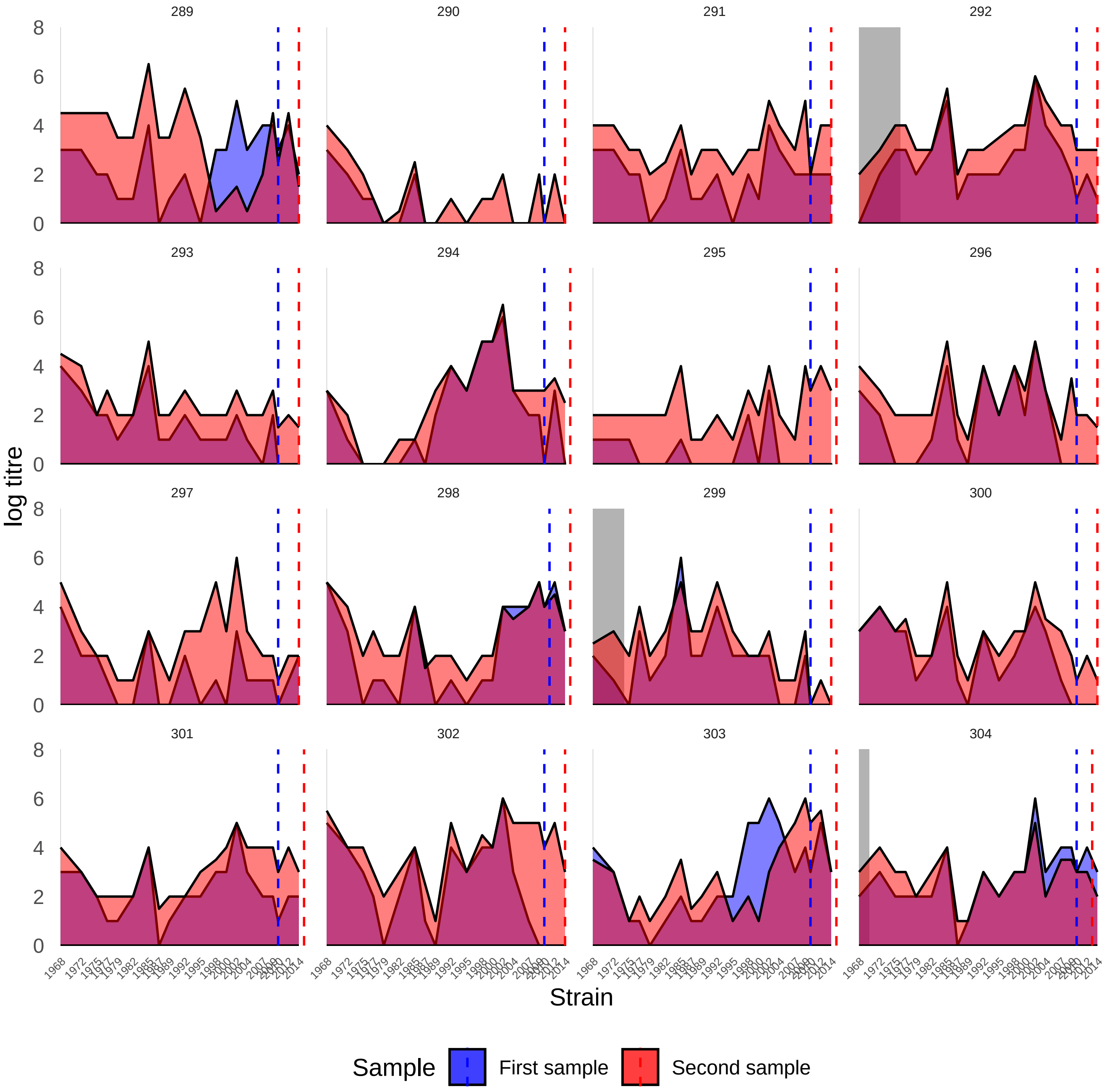
289
290
291
292
293
294
295
296
297
298
299
300
301
302
303
304
log titre
Strain
Sample
First sample
Second sample

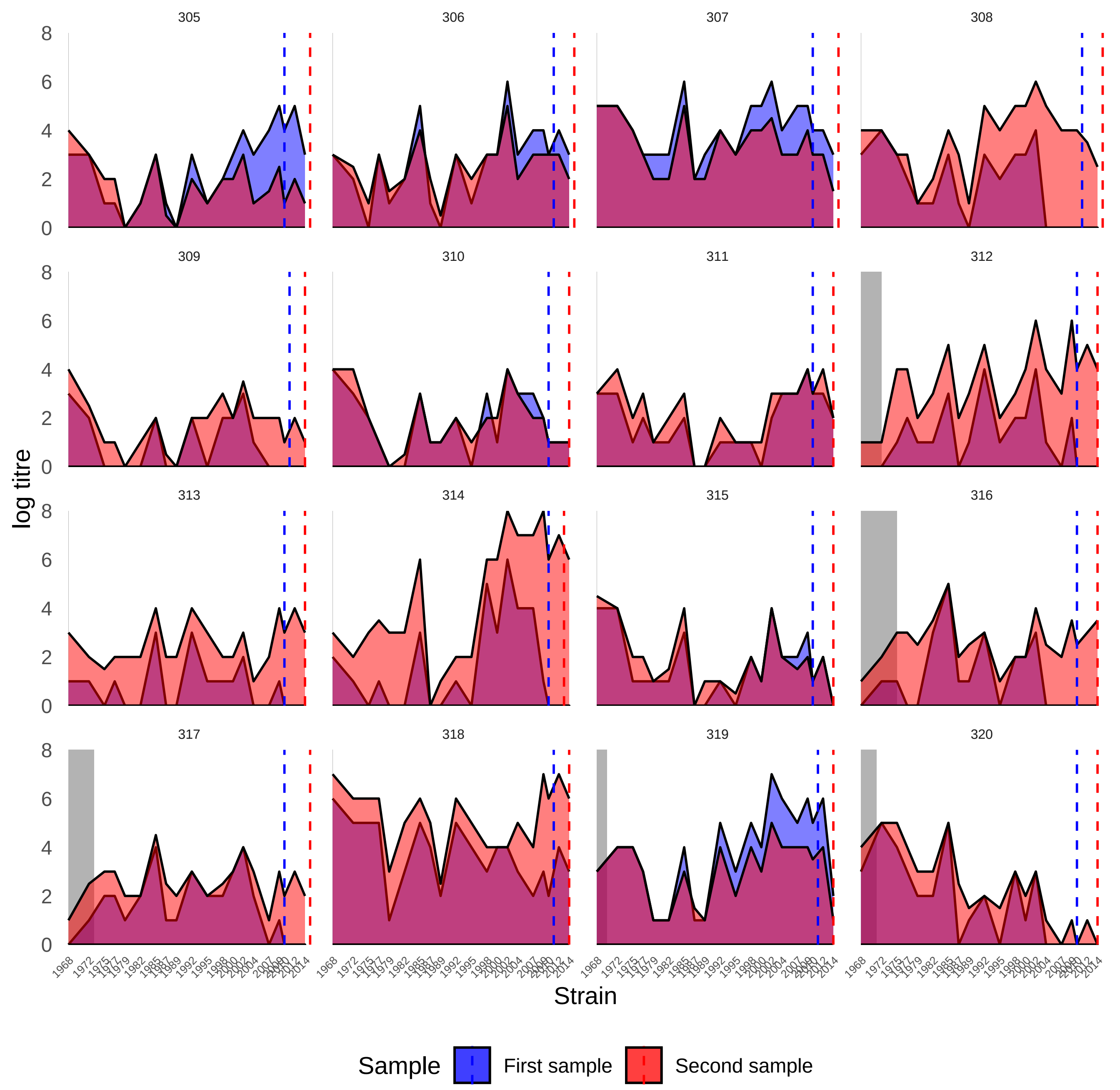
305
306
307
308
309
310
311
312
313
314
315
316
317
318
319
320
log titre
Strain
Sample
First sample
Second sample

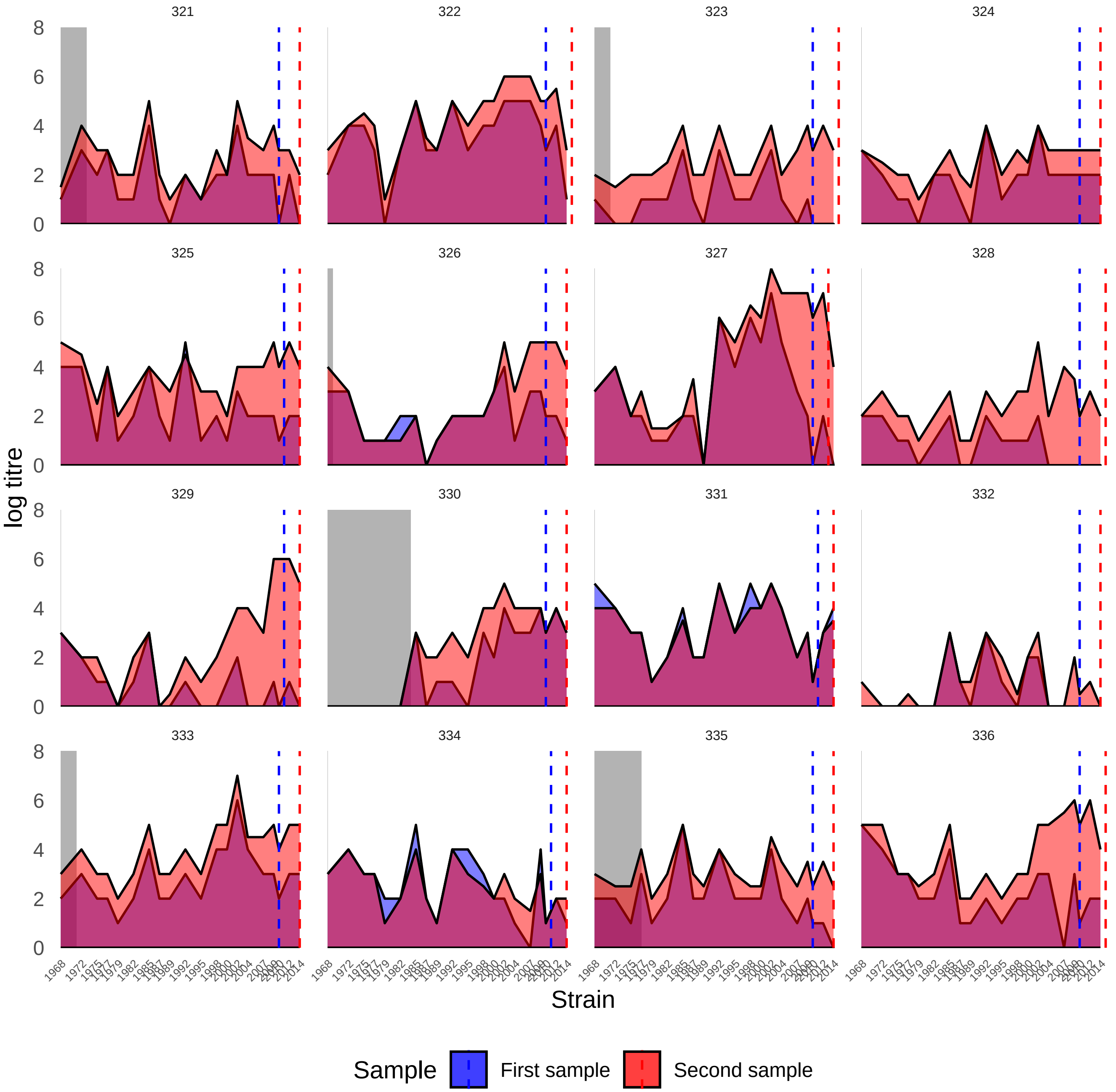

321
322
323
324
325
326
327
328
329
330
331
332
333
334
335
336
log titre
0
2
4
6
8
1968
1972
1975
1977
1979
1982
1985
1987
1989
1992
1995
1998
2000
2002
2004
2007
2009
2010
2012
2014
Strain
Sample
First sample
Second sample

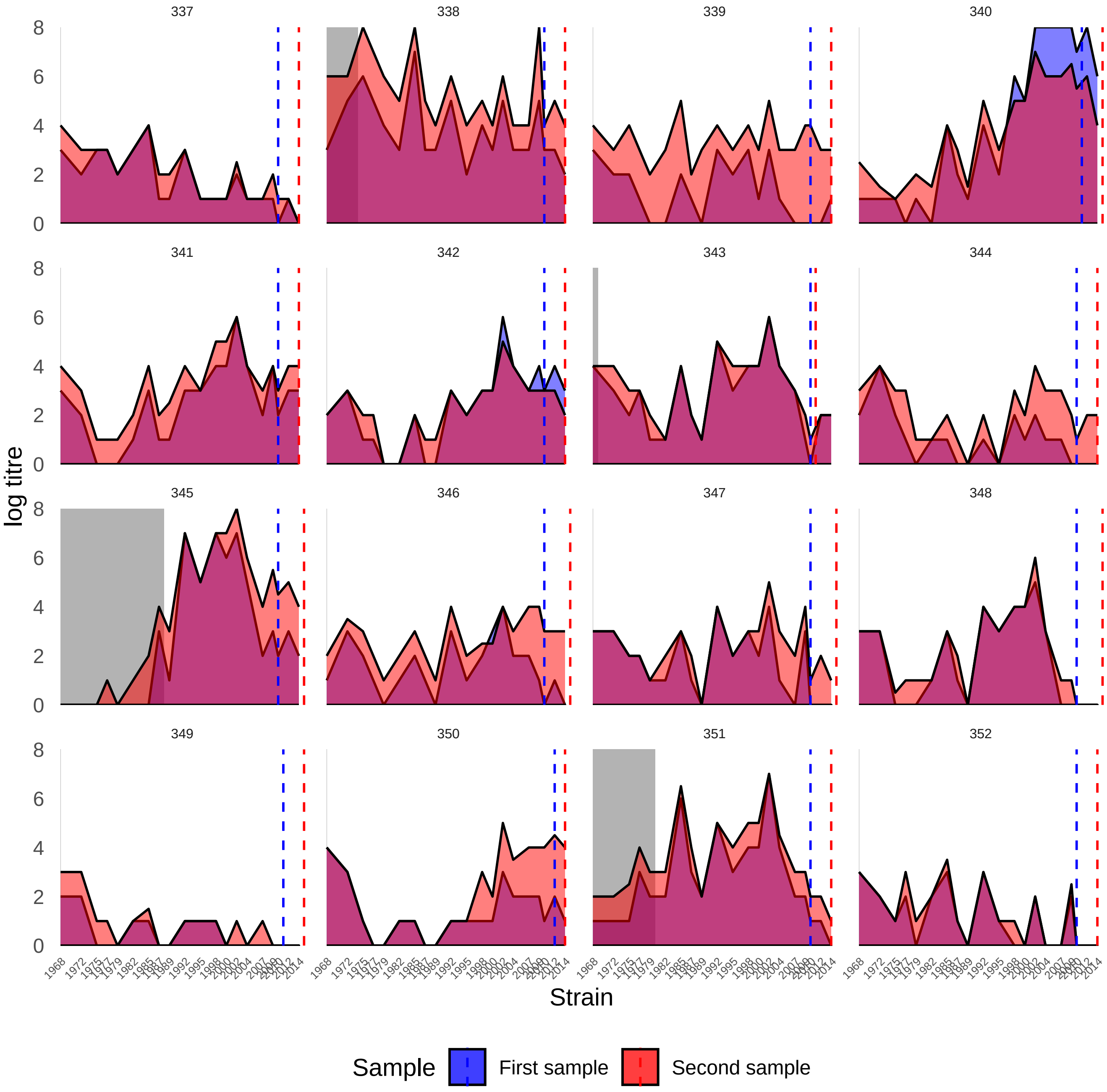

337
338
339
340
341
342
343
344
345
346
347
348
349
350
351
352
0
2
4
6
8
log titre
1968
1972
1975
1977
1979
1982
1985
1987
1989
1992
1995
1998
2000
2002
2004
2007
2009
2010
2012
2014
Strain
Sample
First sample
Second sample

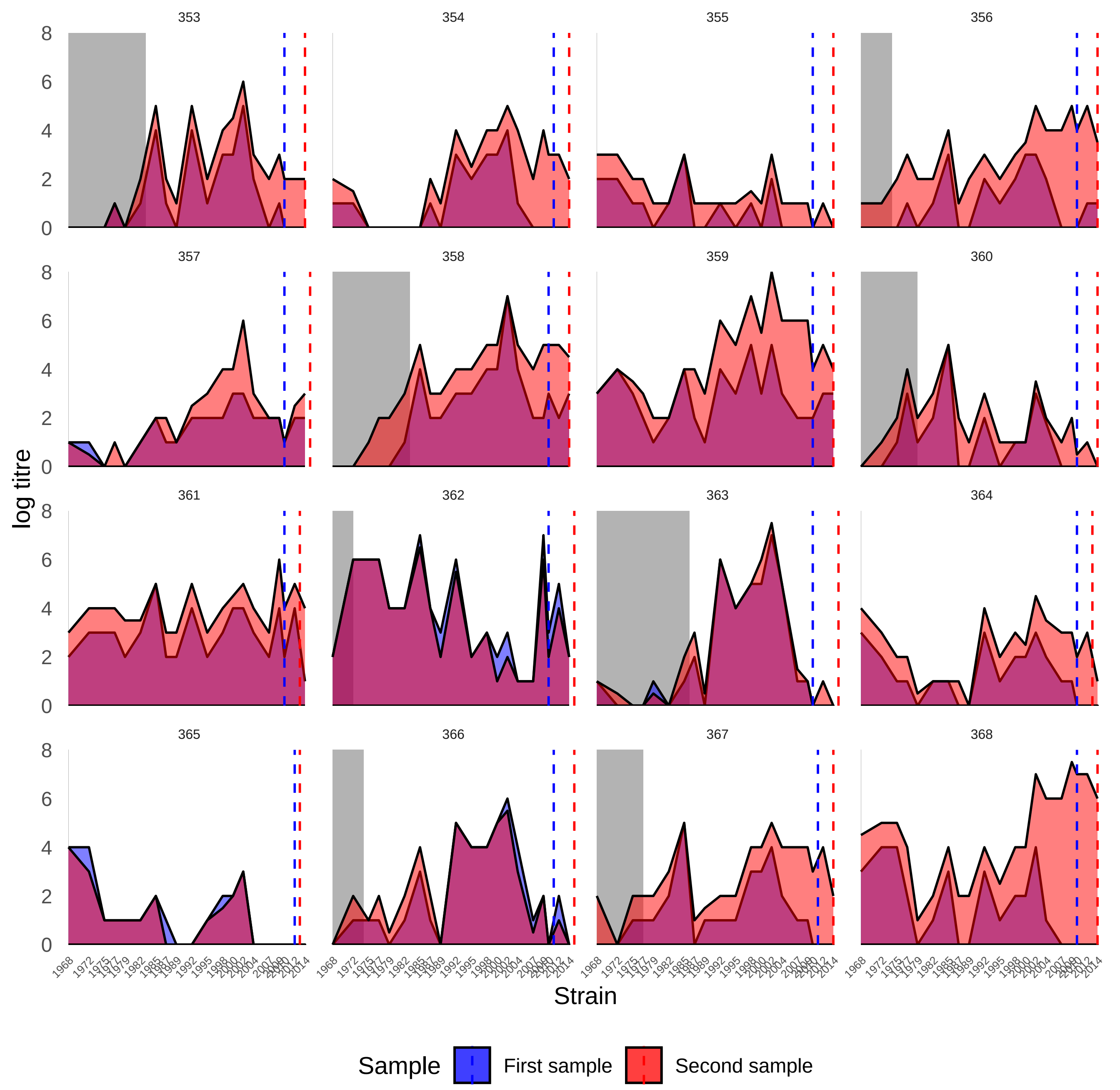
353
354
355
356
357
358
359
360
361
362
363
364
365
366
367
368
log titre
Strain
Sample
First sample
Second sample

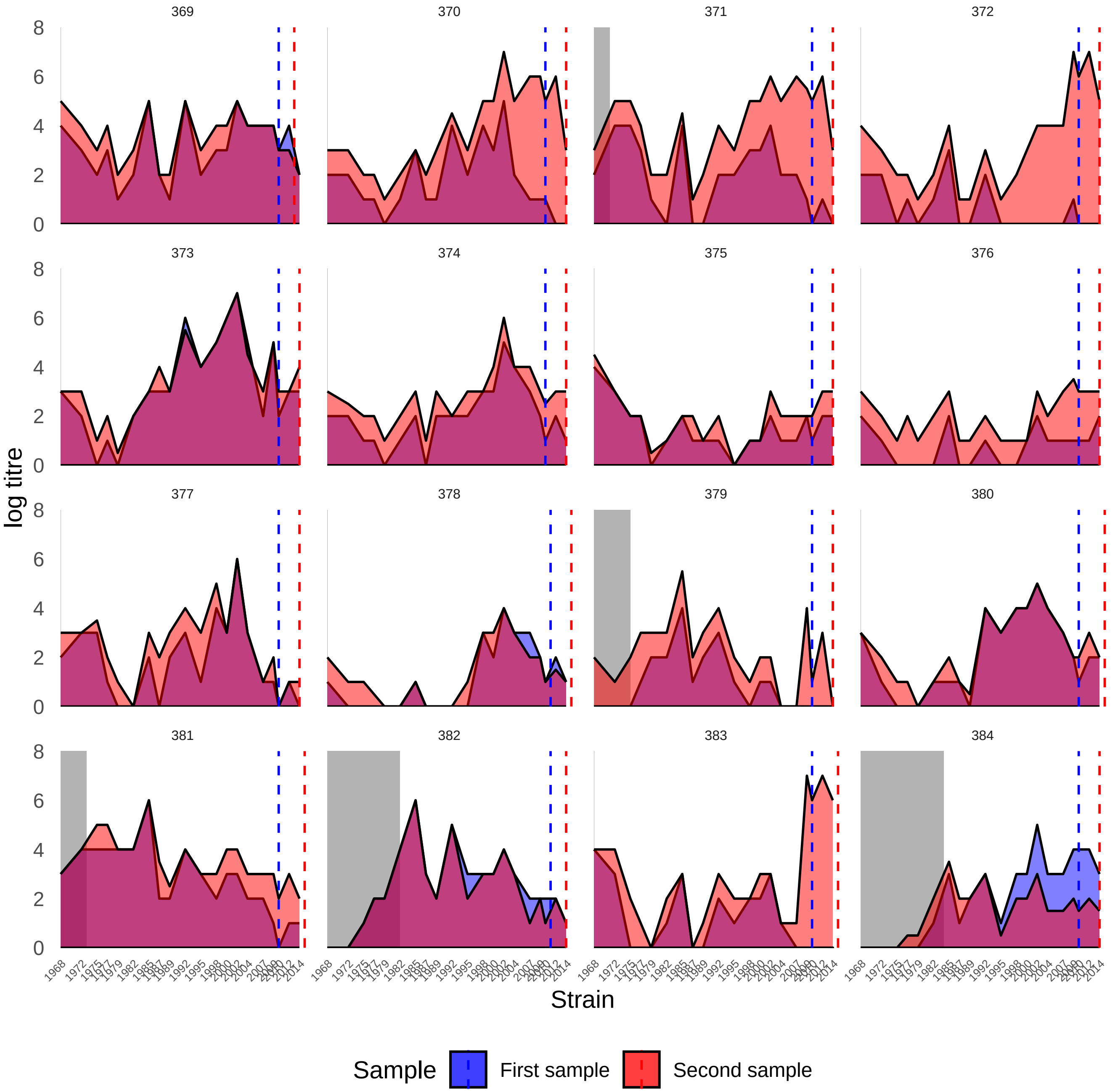

369
370
371
372
373
374
375
376
377
378
379
380
381
382
383
384
log titre
Strain
1968
1972
1975
1977
1979
1982
1985
1987
1989
1992
1995
1998
2000
2002
2004
2007
2009
2010
2012
2014
Sample
First sample
Second sample

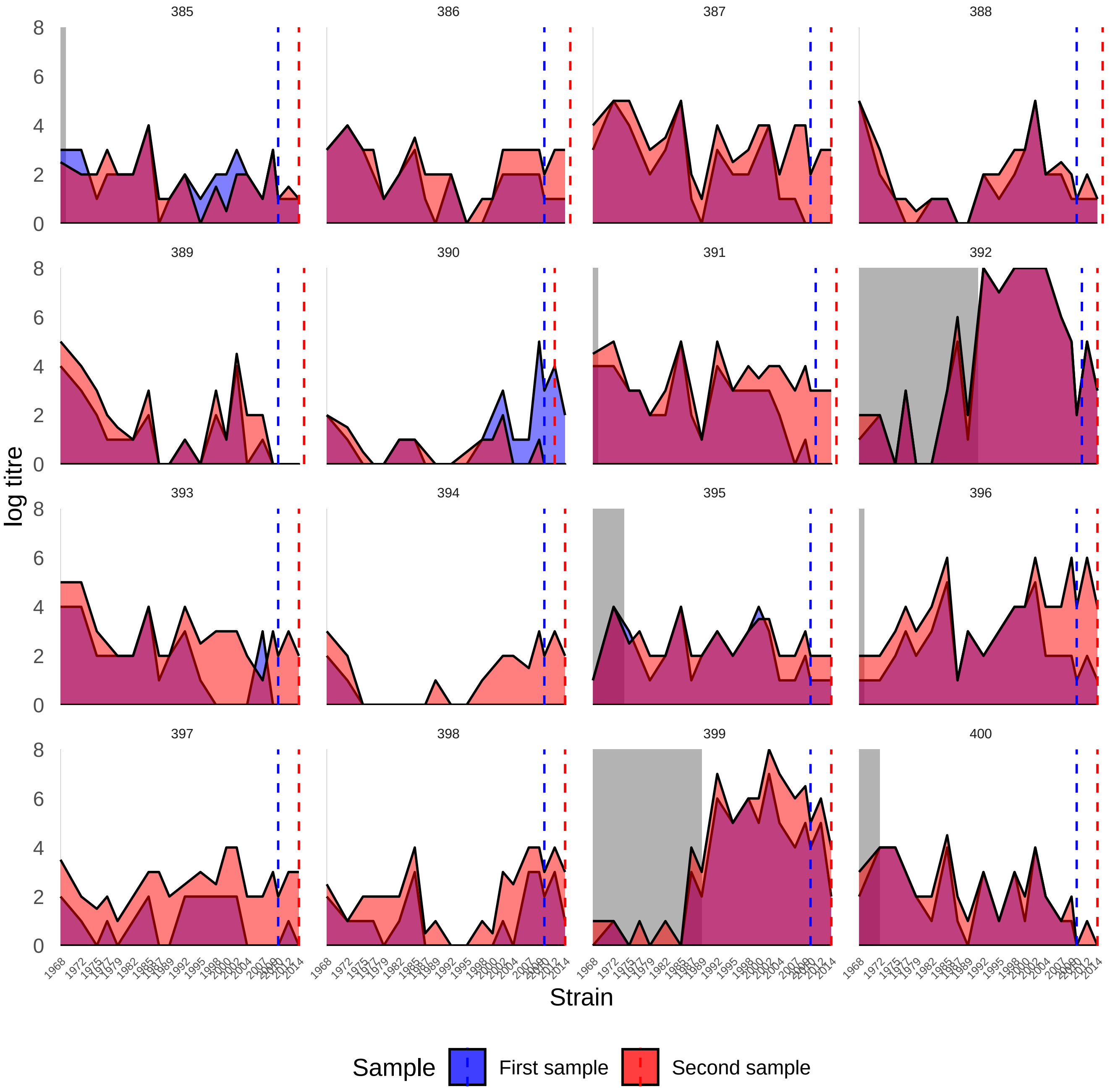

385
386
387
388
389
390
391
392
393
394
395
396
397
398
399
400
log titre
0
2
4
6
8
1968
1972
1975
1977
1979
1982
1985
1987
1989
1992
1995
1998
2000
2002
2004
2007
2009
2010
2012
2014
Strain
Sample
First sample
Second sample

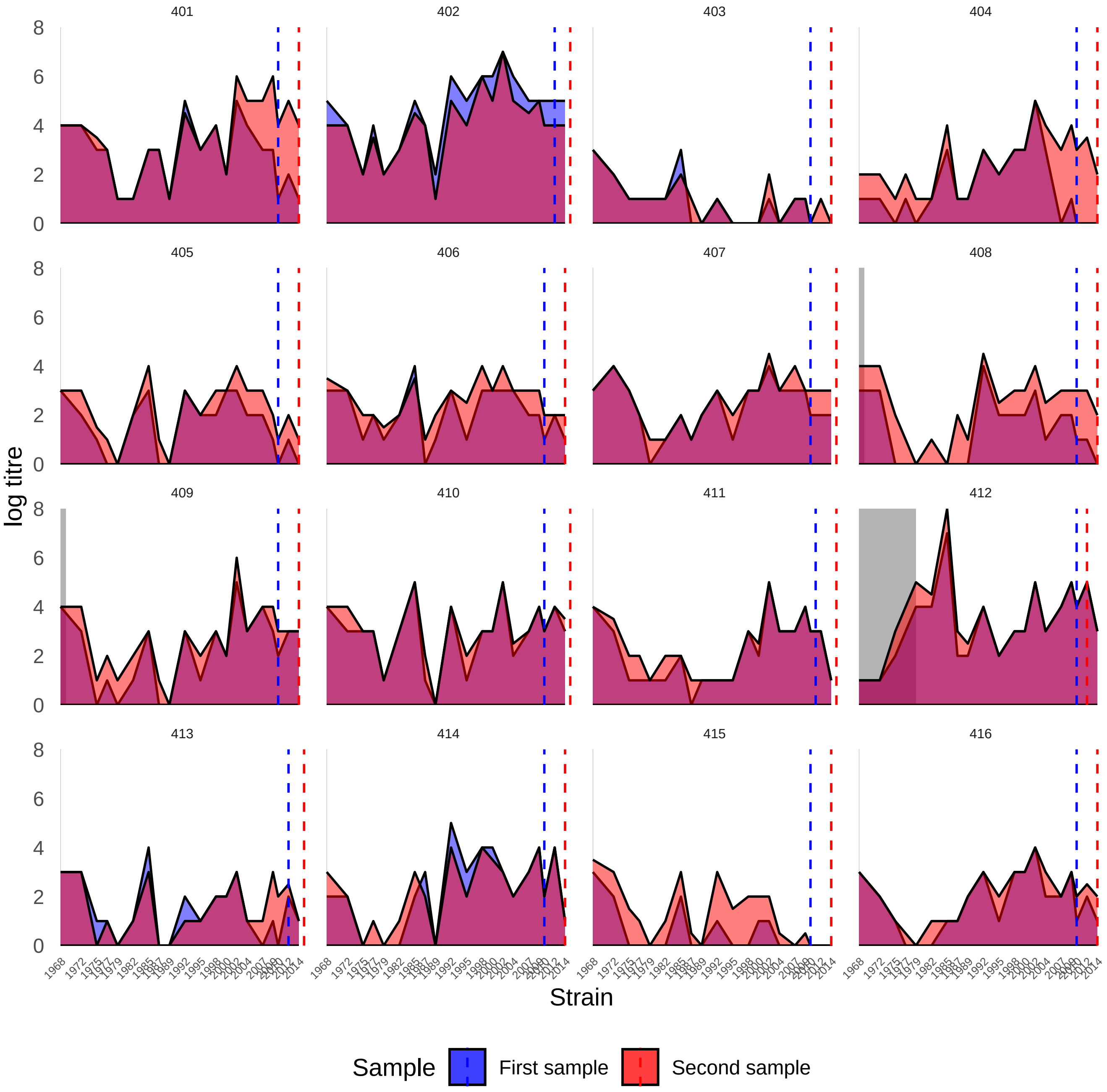
401
402
403
404
405
406
407
408
409
410
411
412
413
414
415
416
log titre
Strain
Sample
First sample
Second sample

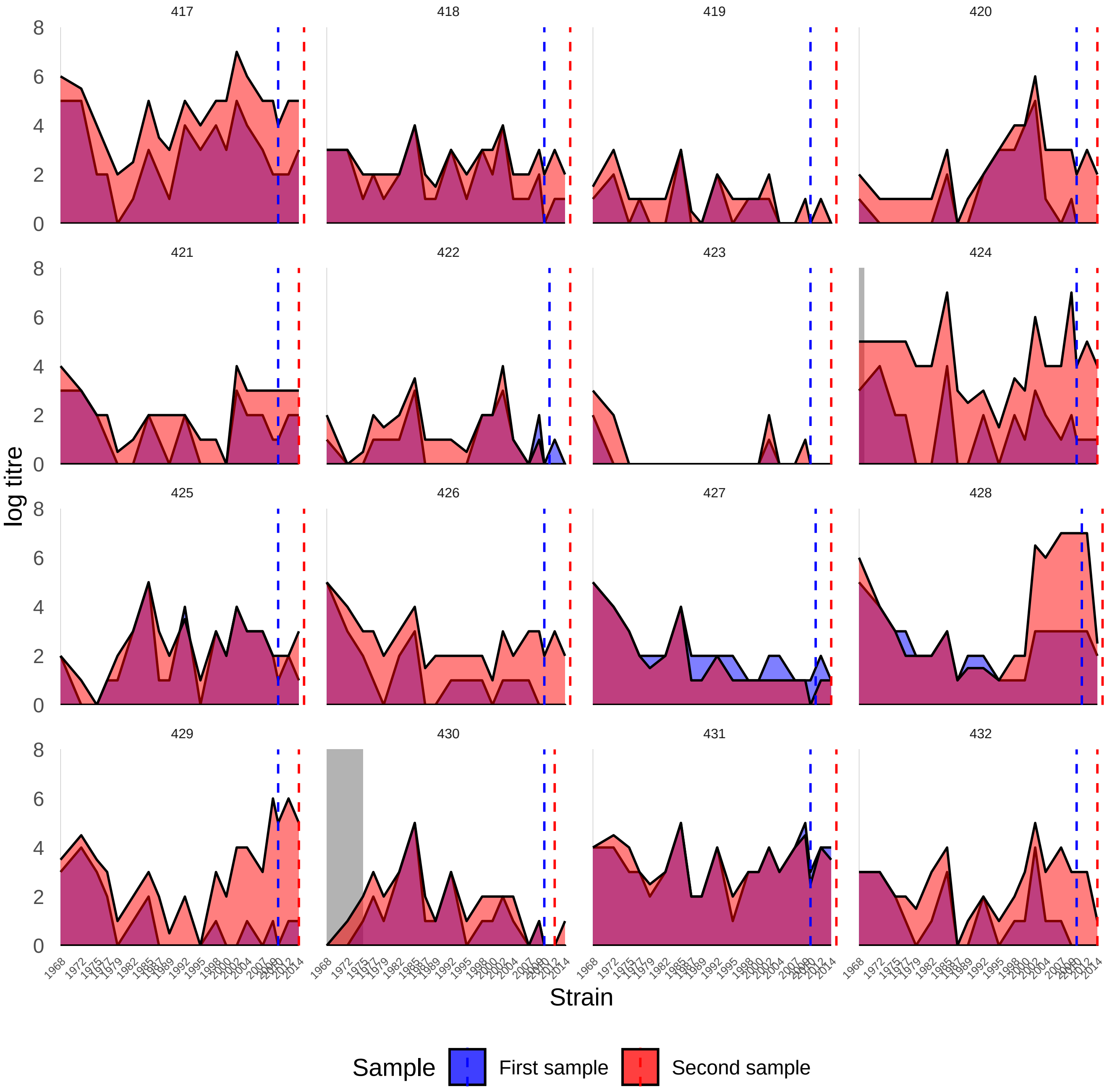

417
418
419
420
421
422
423
424
425
426
427
428
429
430
431
432
0
2
4
6
8
log titre
1968
1972
1975
1977
1979
1982
1985
1987
1989
1992
1995
1998
2000
2002
2004
2007
2009
2010
2012
2014
Strain
Sample
First sample
Second sample

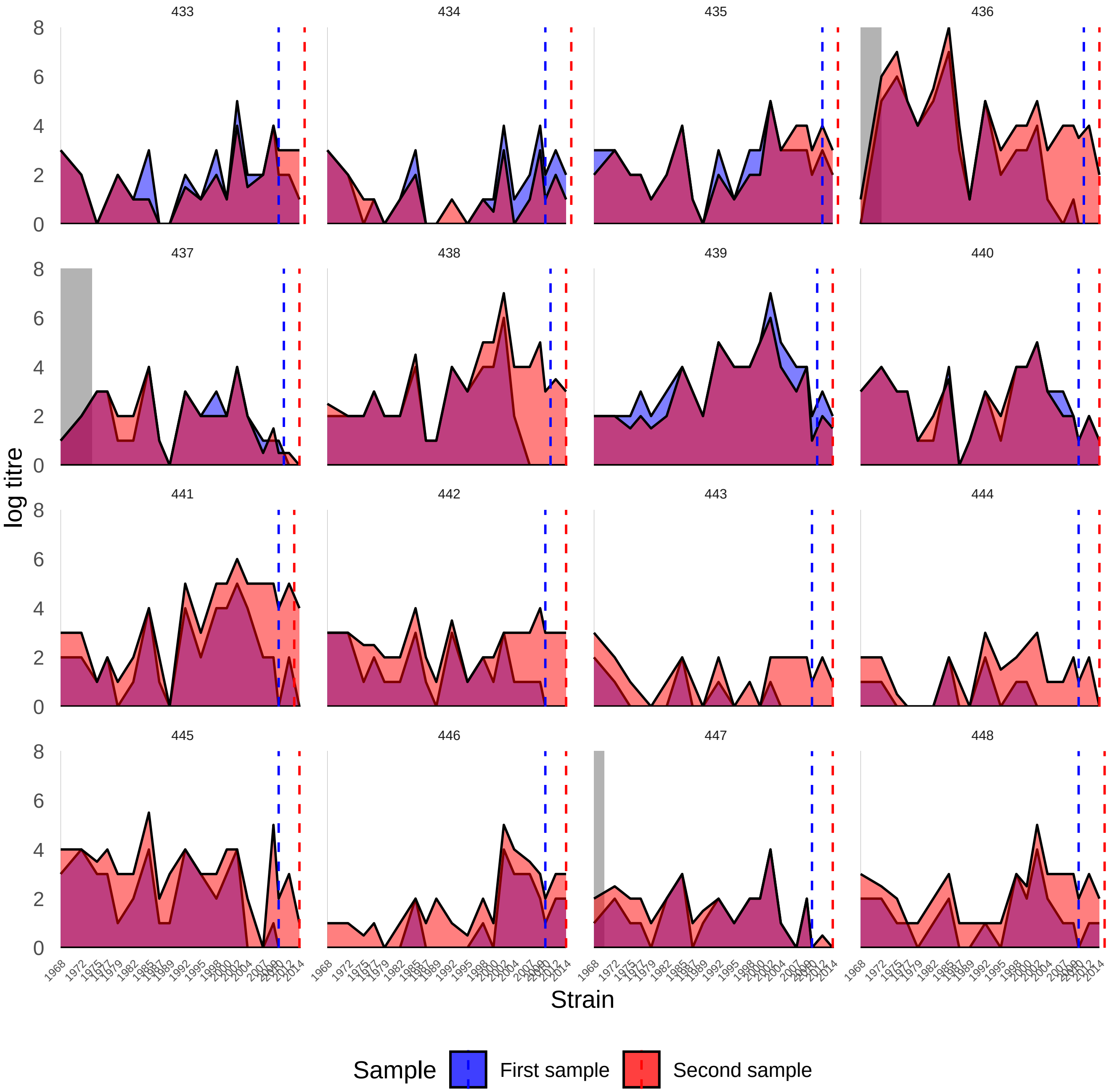
433
434
435
436
437
438
439
440
441
442
443
444
445
446
447
448
log titre
Strain
1968 1972 1975 1977 1979 1982 1985 1987 1989 1992 1995 1998 2000 2002 2004 2007 2009 2010 2012 2014
Sample
First sample
Second sample

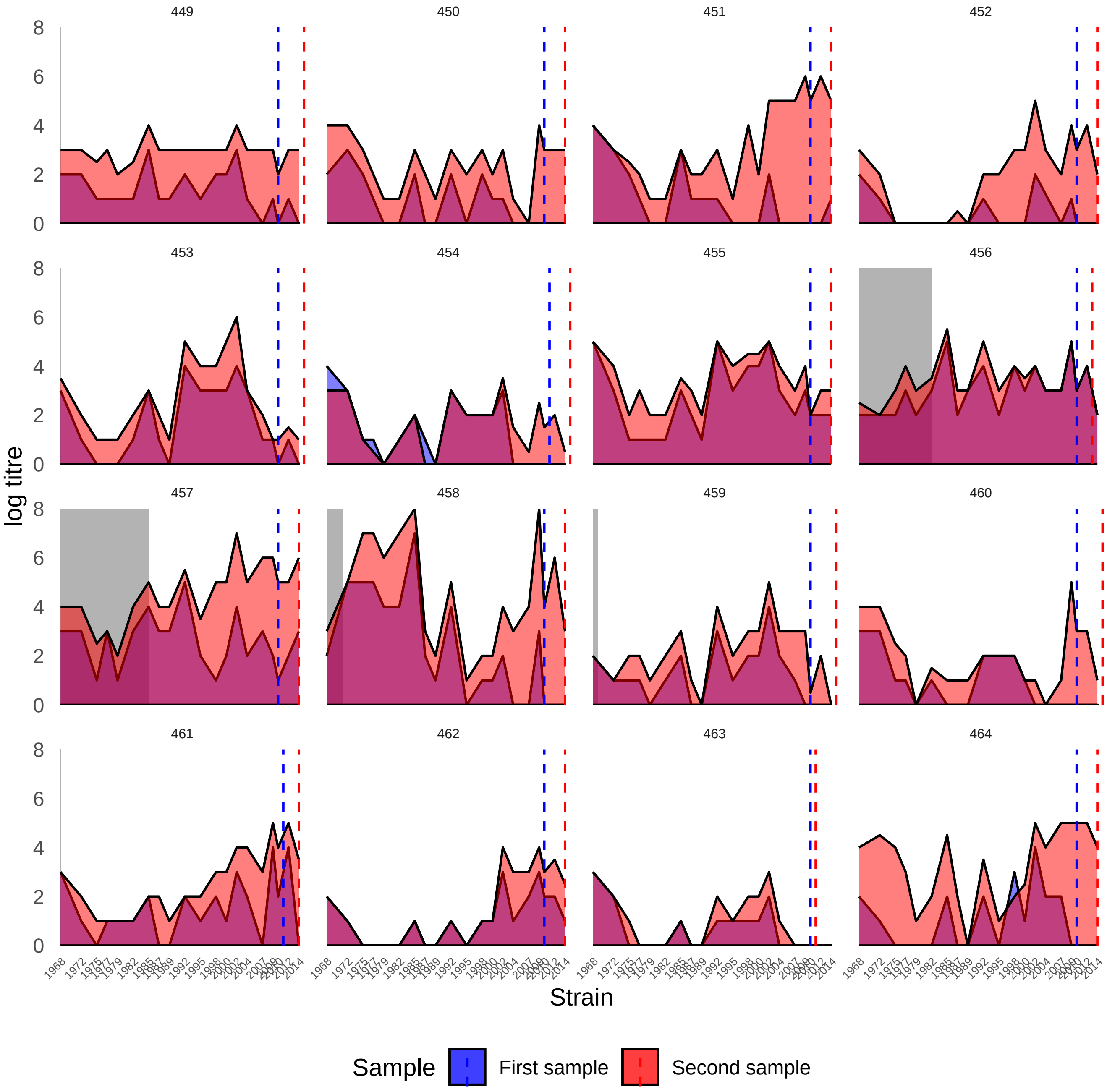
449
450
451
452
453
454
455
456
457
458
459
460
461
462
463
464
log titre
Strain
Sample
First sample
Second sample

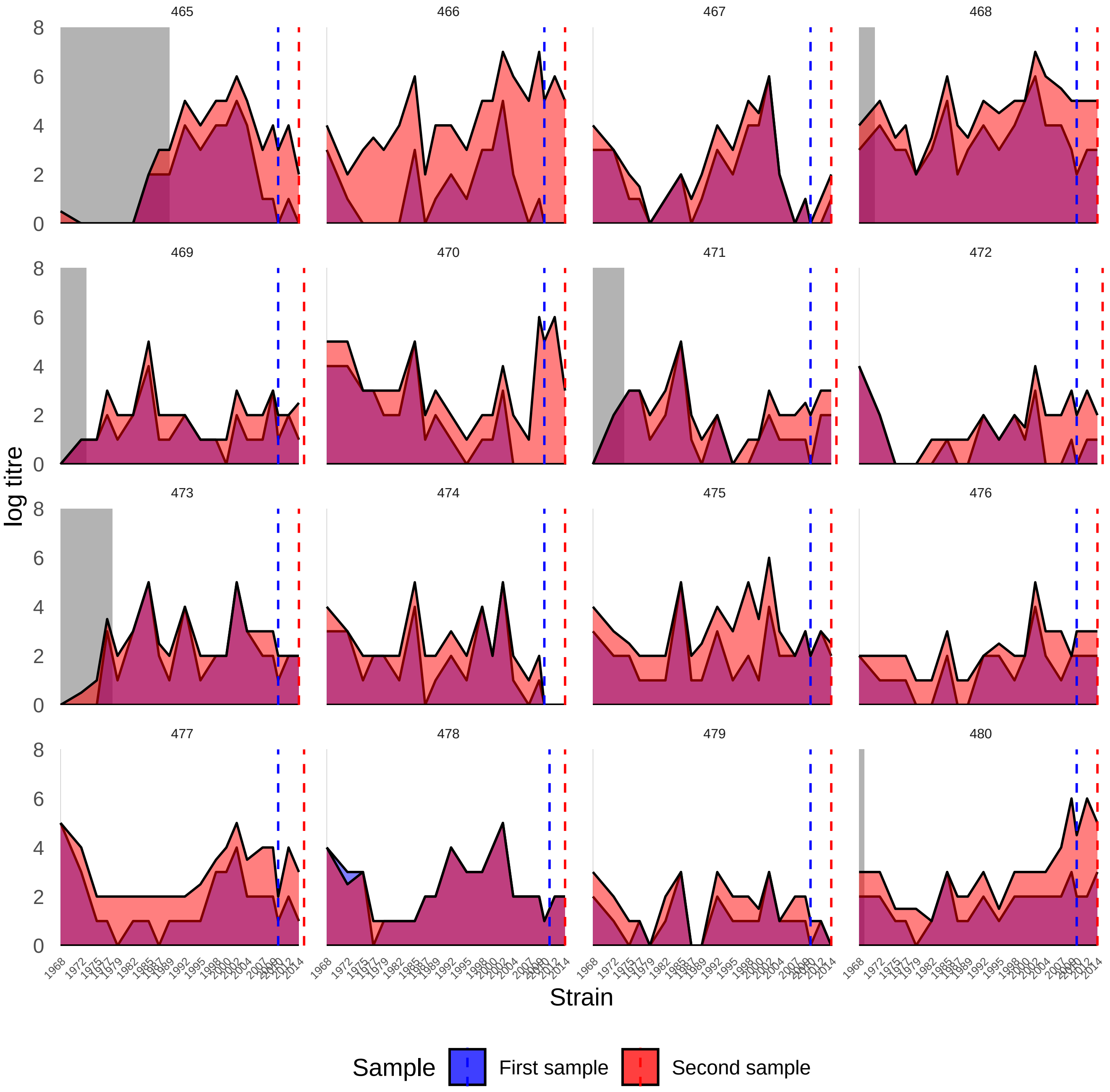

465
466
467
468
469
470
471
472
473
474
475
476
477
478
479
480
log titre
Strain
Sample
First sample
Second sample

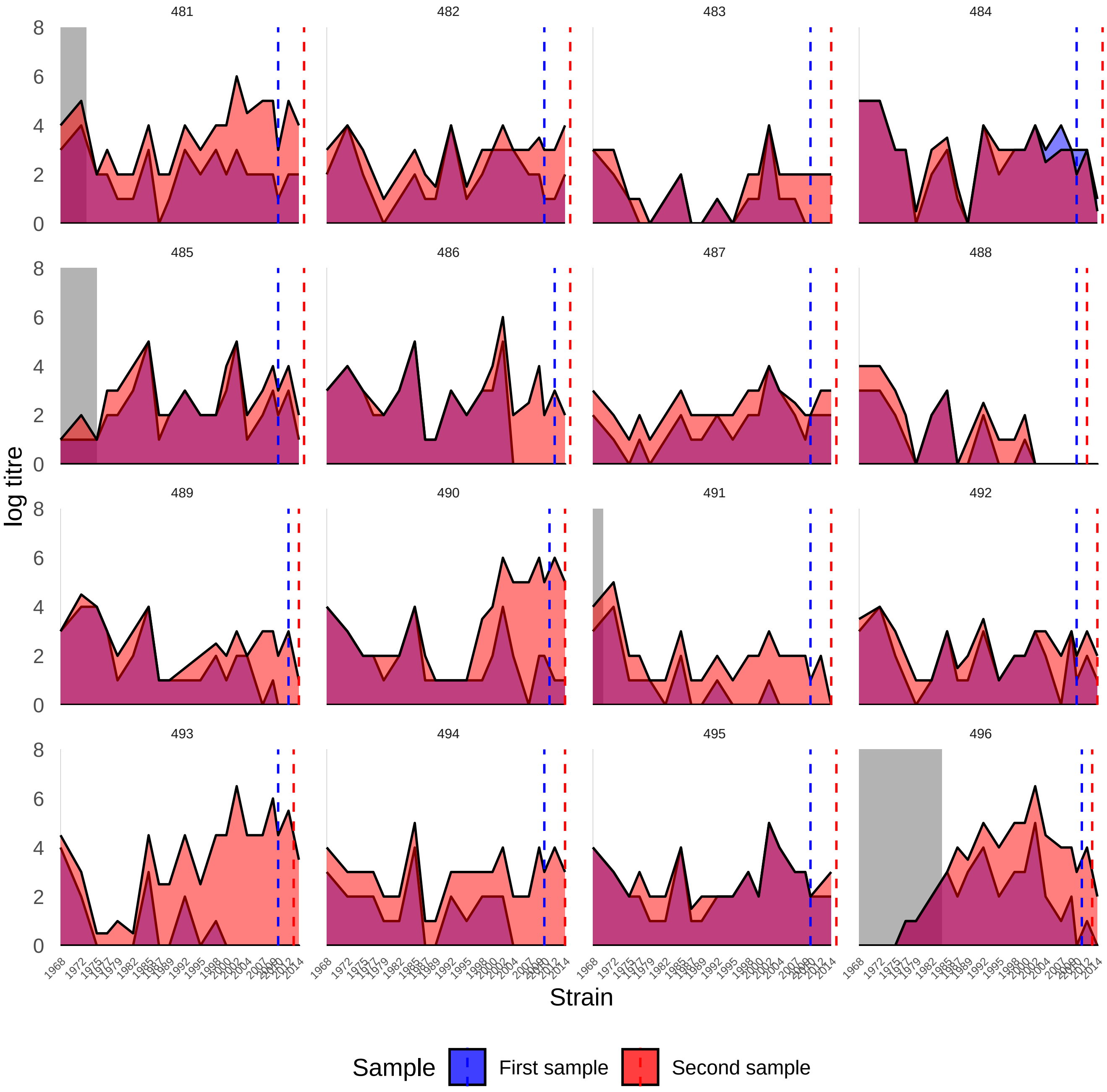
481
482
483
484
485
486
487
488
489
490
491
492
493
494
495
496
log titre
0
2
4
6
8
Strain
1968
1972
1975
1977
1979
1982
1985
1987
1989
1992
1995
1998
2000
2002
2004
2007
2009
2010
2012
2014
Sample
First sample
Second sample

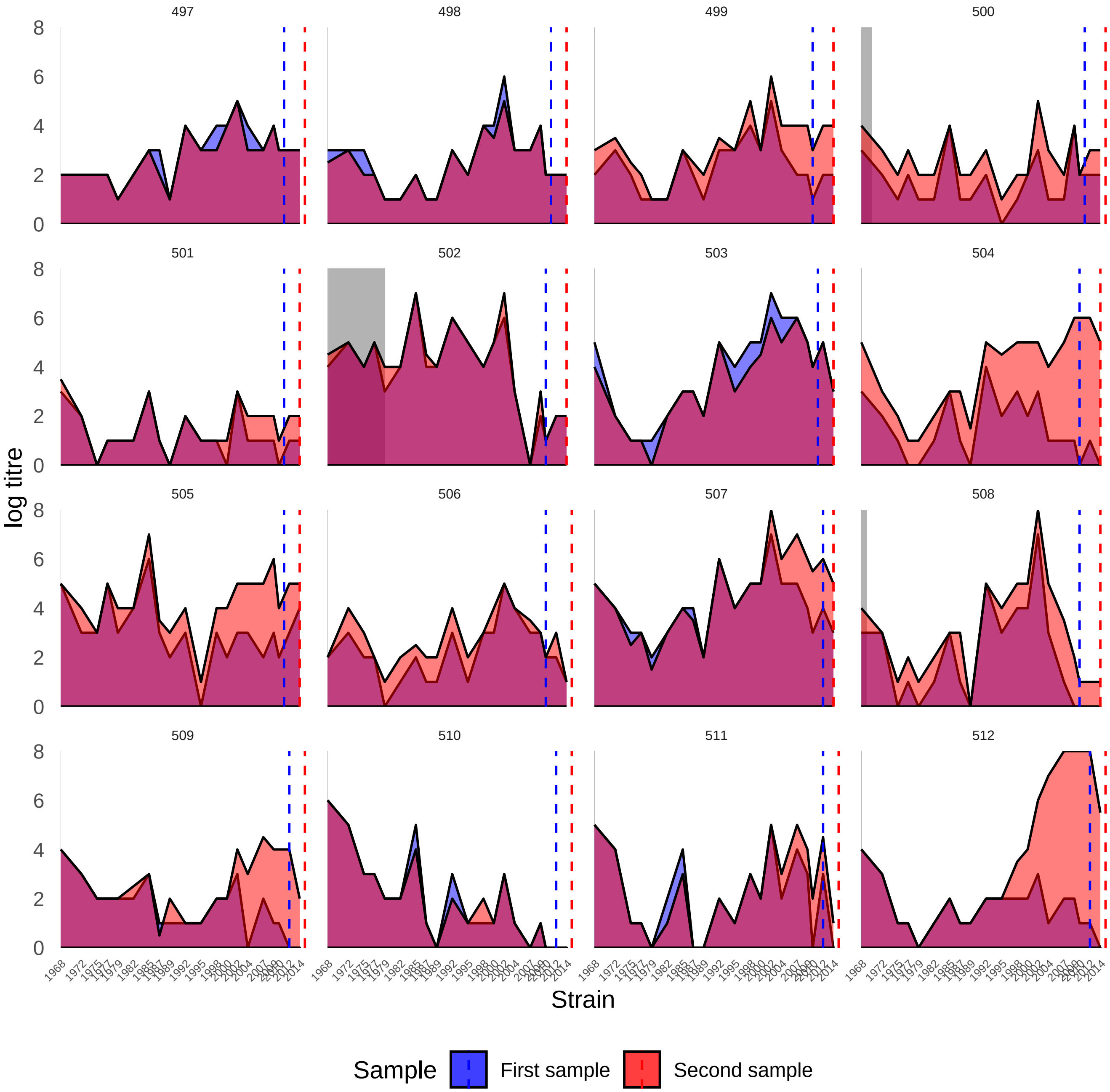
497
498
499
500
501
502
503
504
505
506
507
508
509
510
511
512
log titre
0
2
4
6
8
1968
1972
1975
1977
1979
1982
1985
1987
1989
1992
1995
1998
2000
2002
2004
2007
2009
2010
2012
2014
Strain
Sample
First sample
Second sample

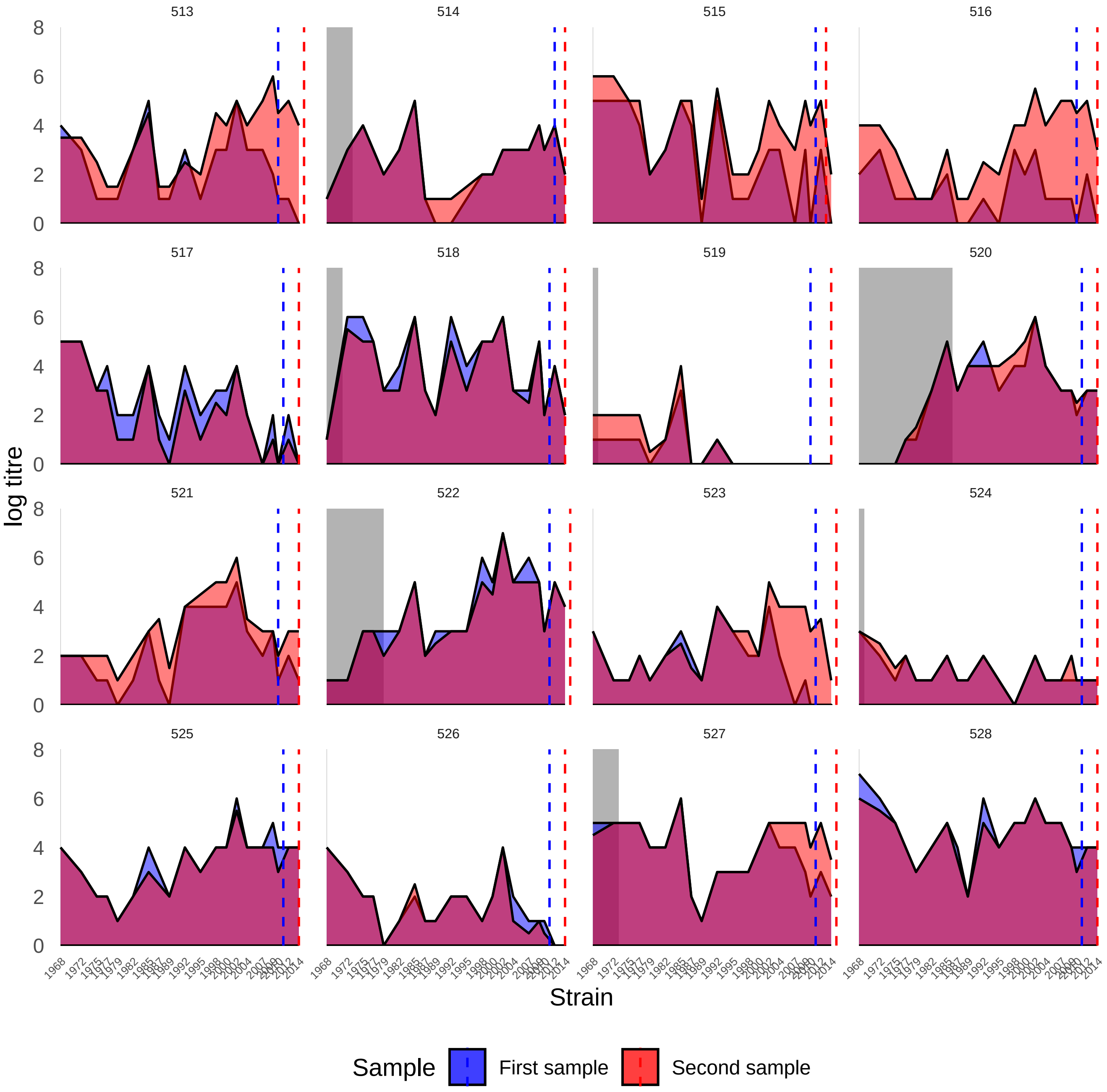

513
514
515
516
517
518
519
520
521
522
523
524
525
526
527
528
log titre
Strain
Sample
First sample
Second sample

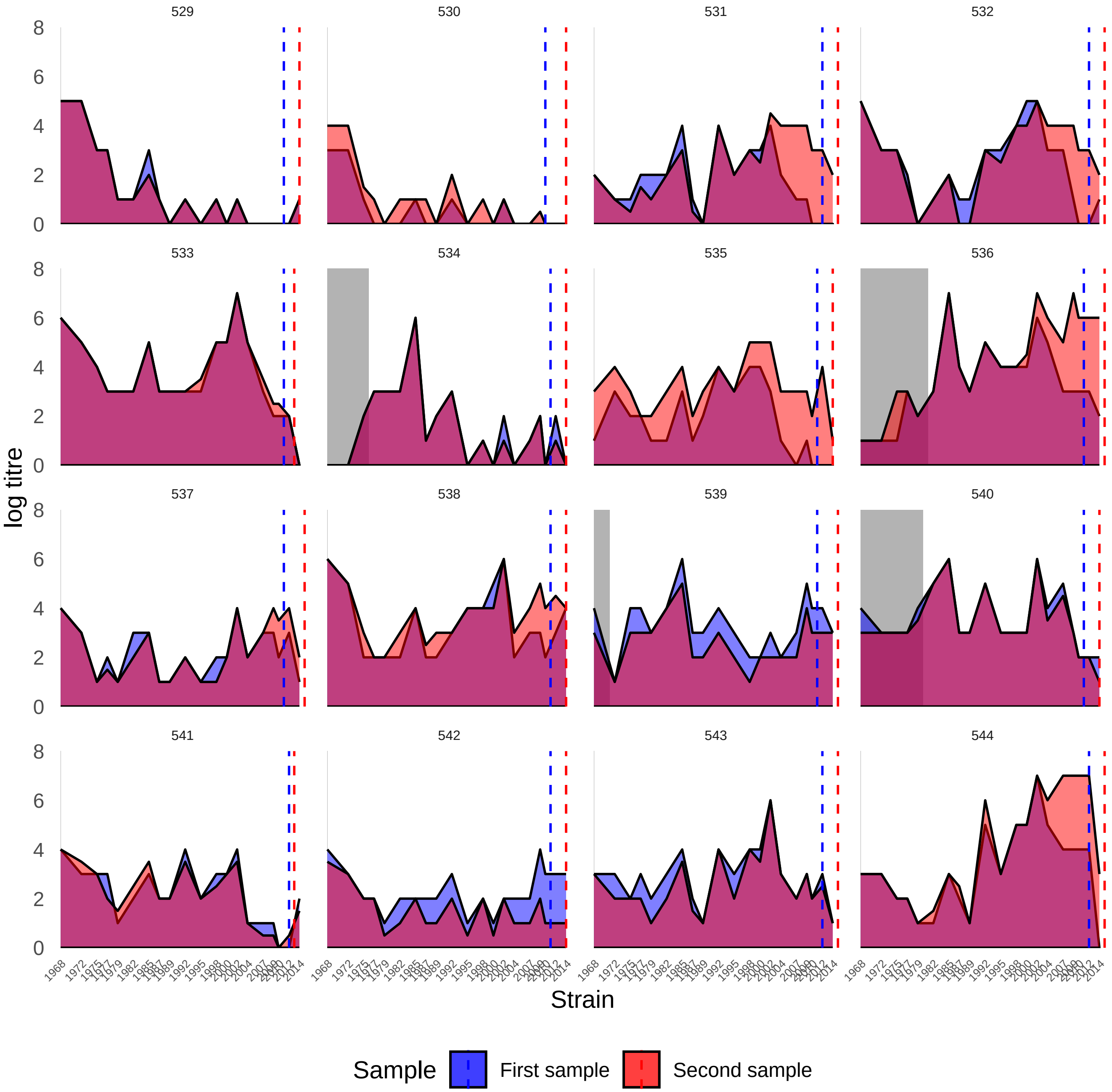
529
530
531
532
533
534
535
536
537
538
539
540
541
542
543
544
log titre
Strain
Sample
First sample
Second sample

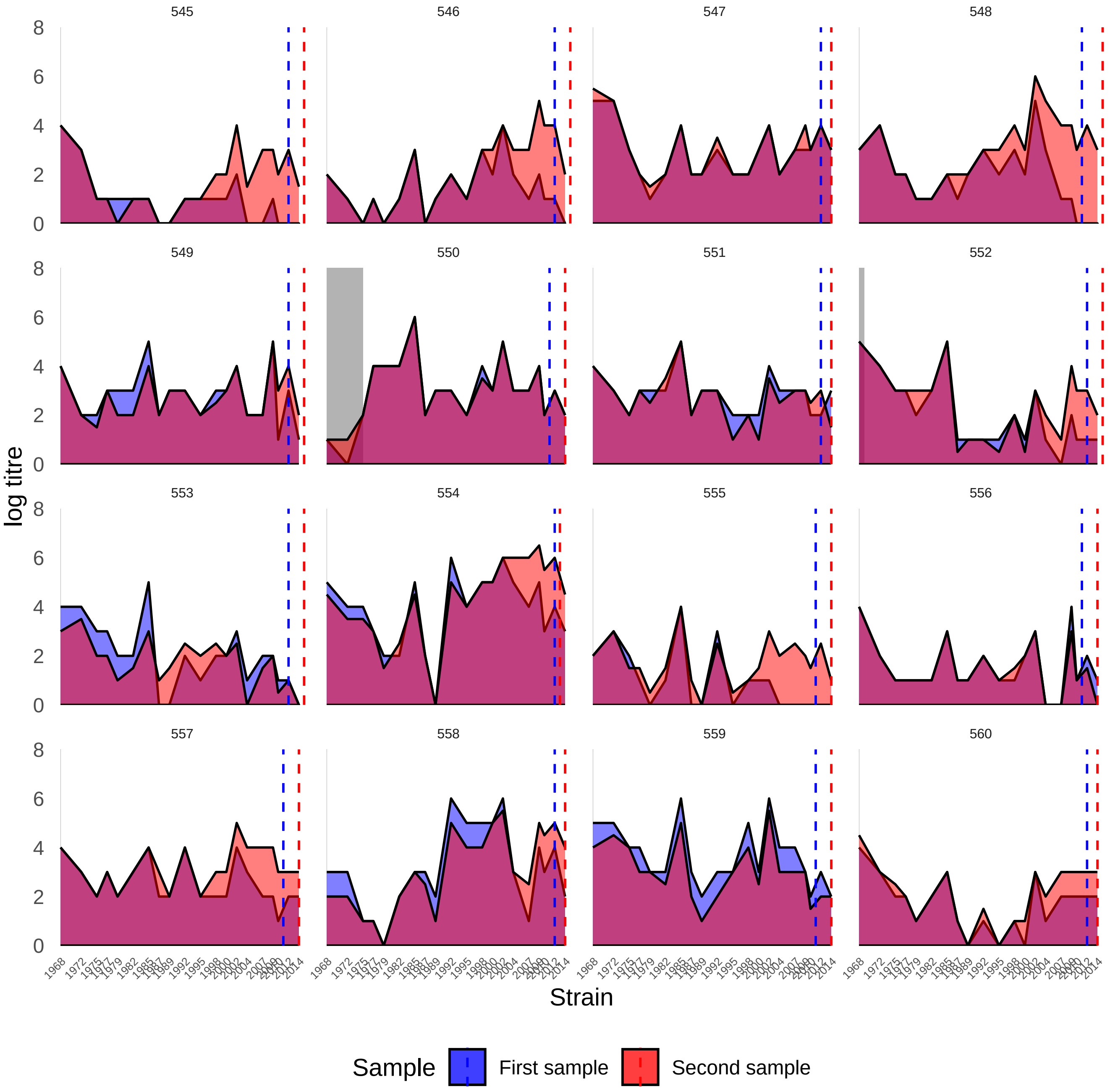
545
546
547
548
549
550
551
552
553
554
555
556
557
558
559
560
log titre
Strain
Sample
First sample
Second sample

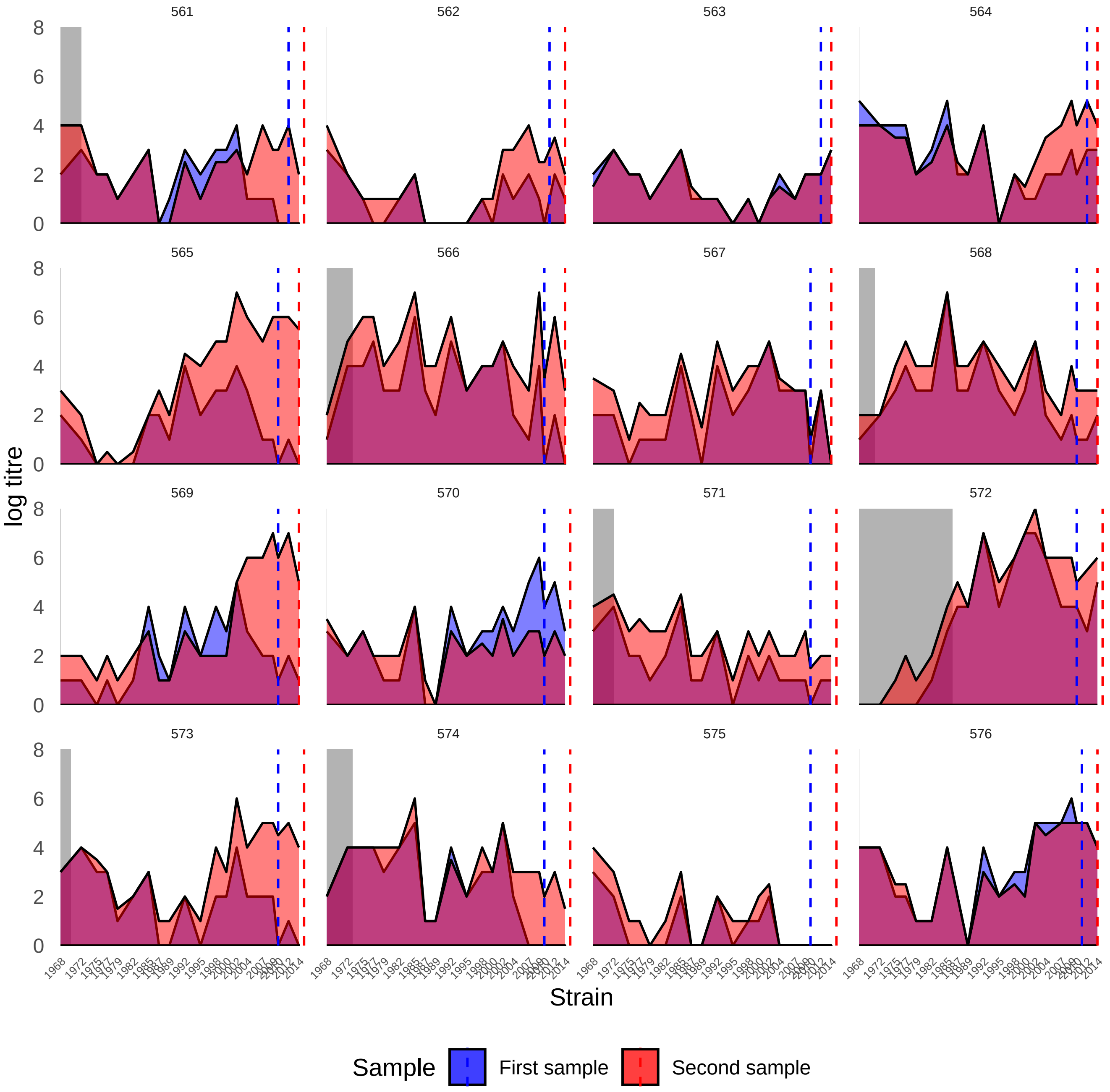

561
562
563
564
565
566
567
568
569
570
571
572
573
574
575
576
log titre
Strain
Sample
First sample
Second sample

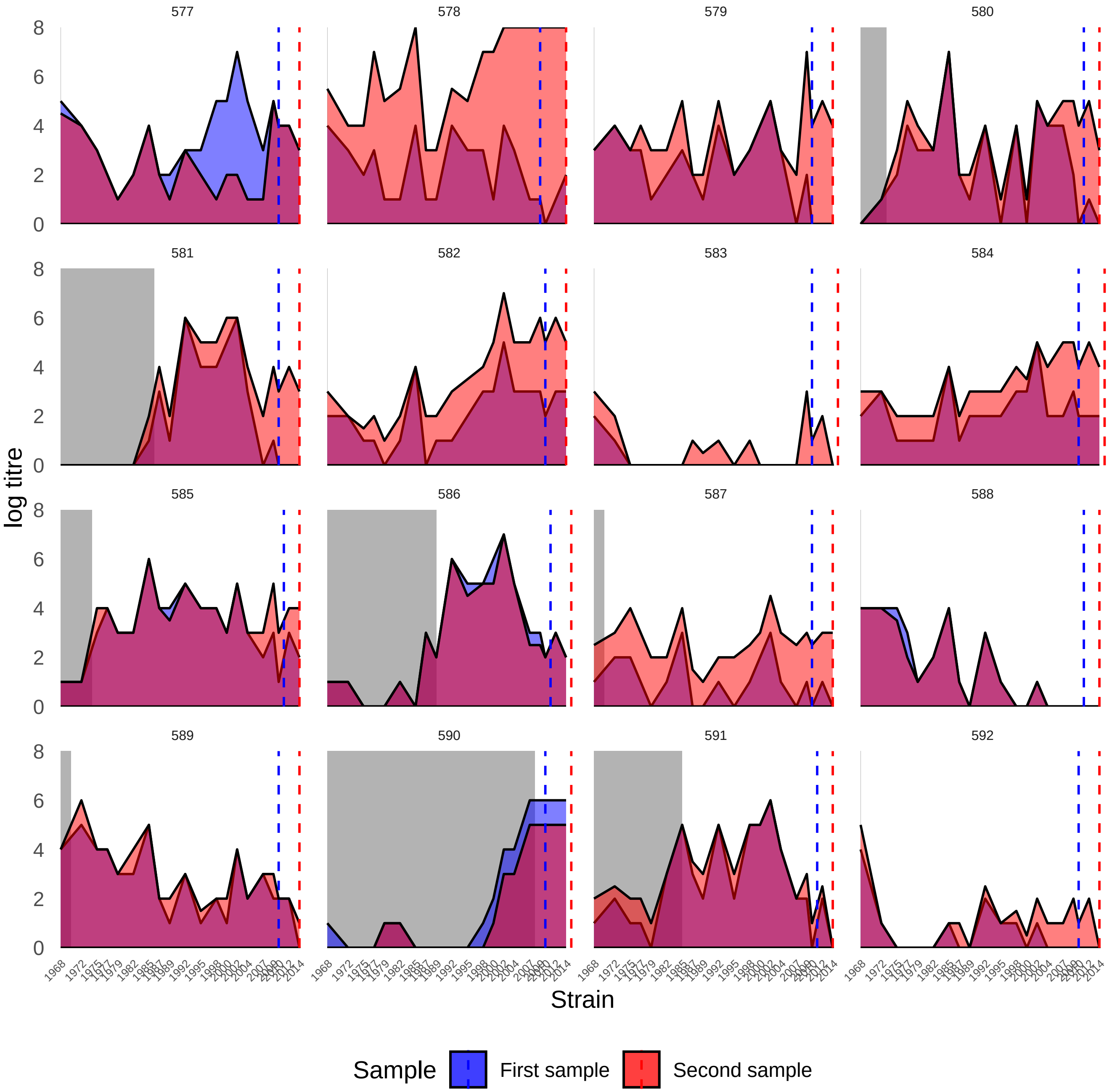

577
578
579
580
581
582
583
584
585
586
587
588
589
590
591
592
log titre
Strain
Sample
First sample
Second sample

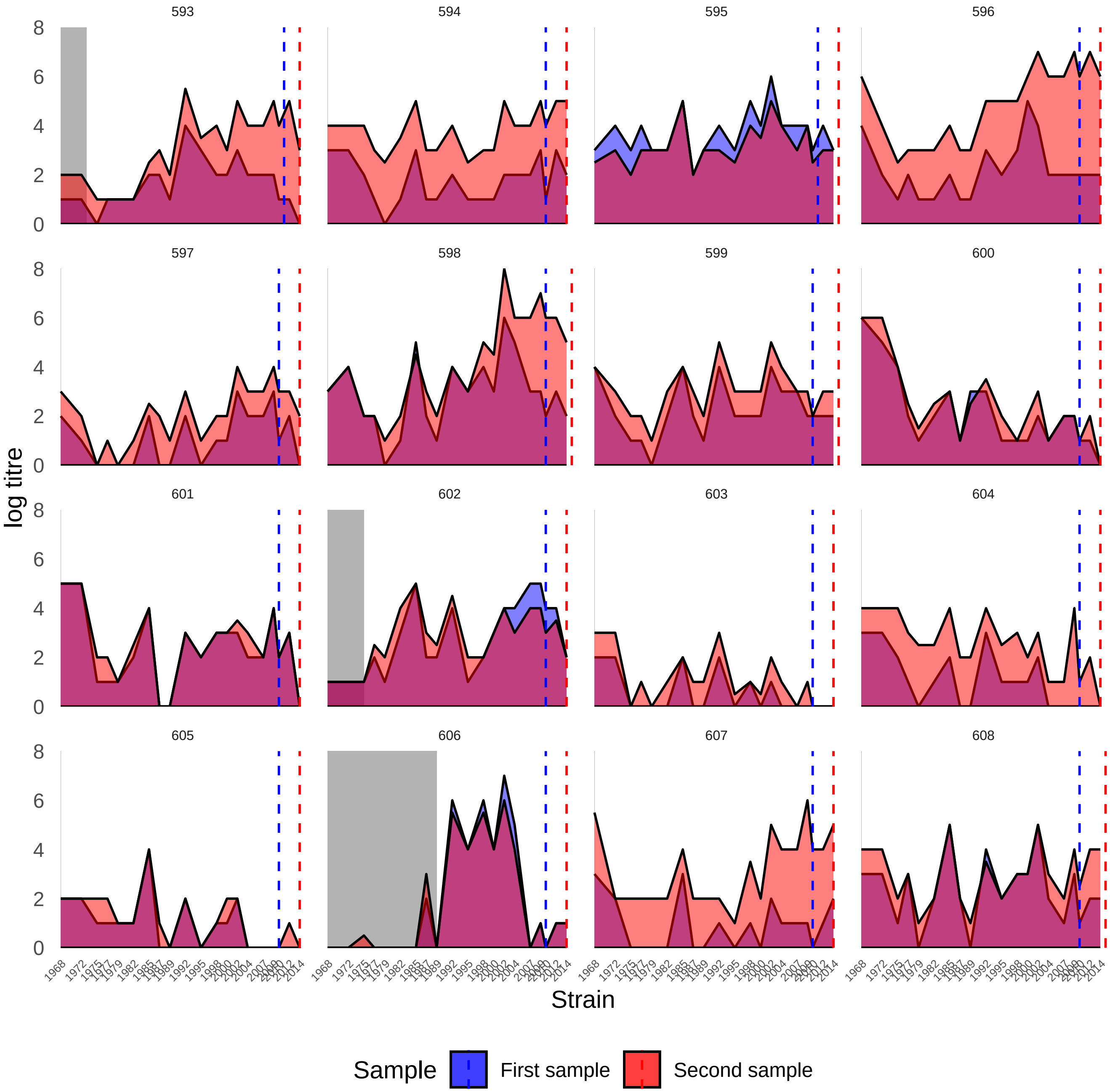
593
594
595
596
597
598
599
600
601
602
603
604
605
606
607
608
log titre
Strain
Sample
First sample
Second sample

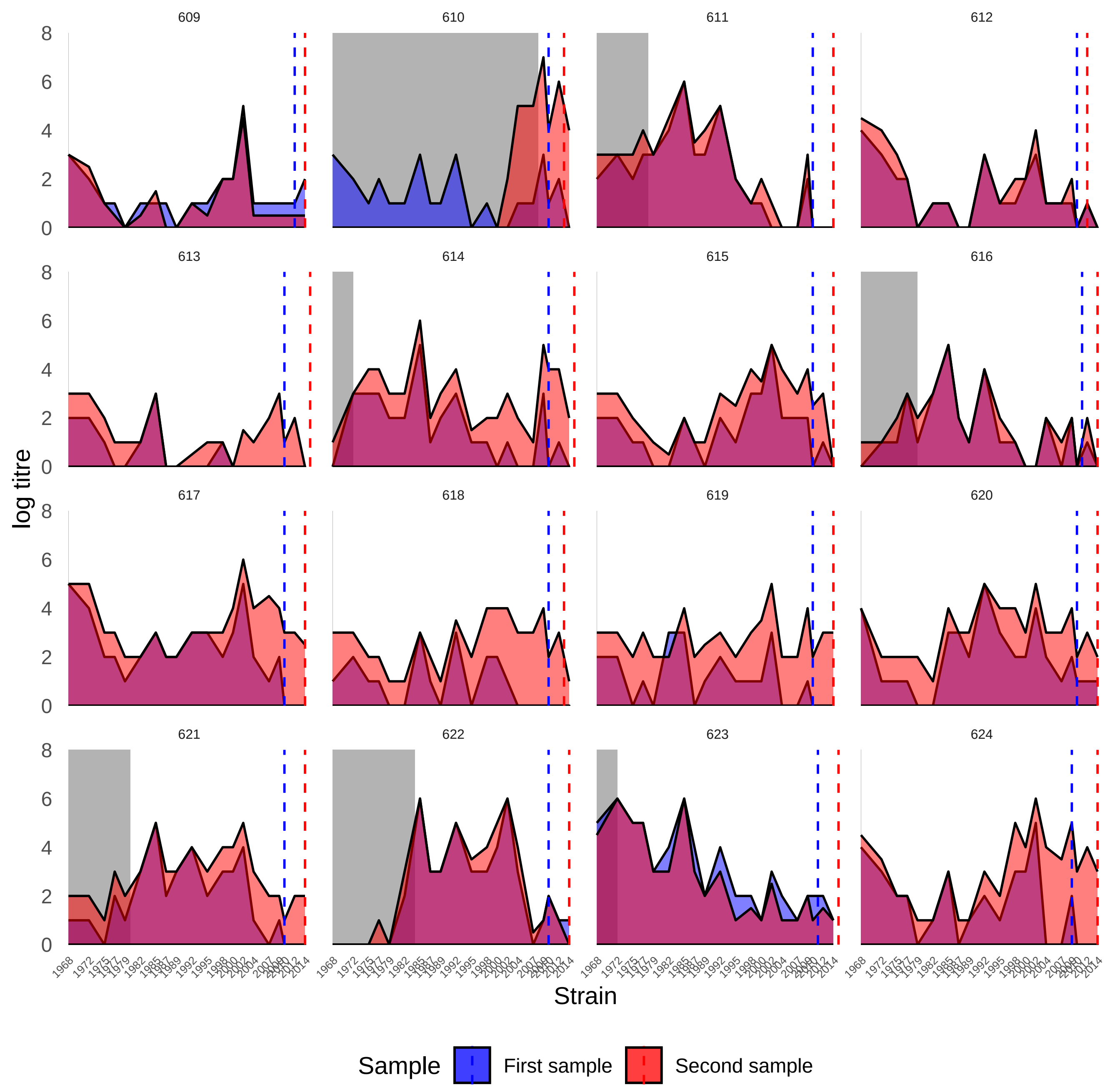

609
610
611
612
613
614
615
616
617
618
619
620
621
622
623
624
log titre
Strain
Sample
First sample
Second sample

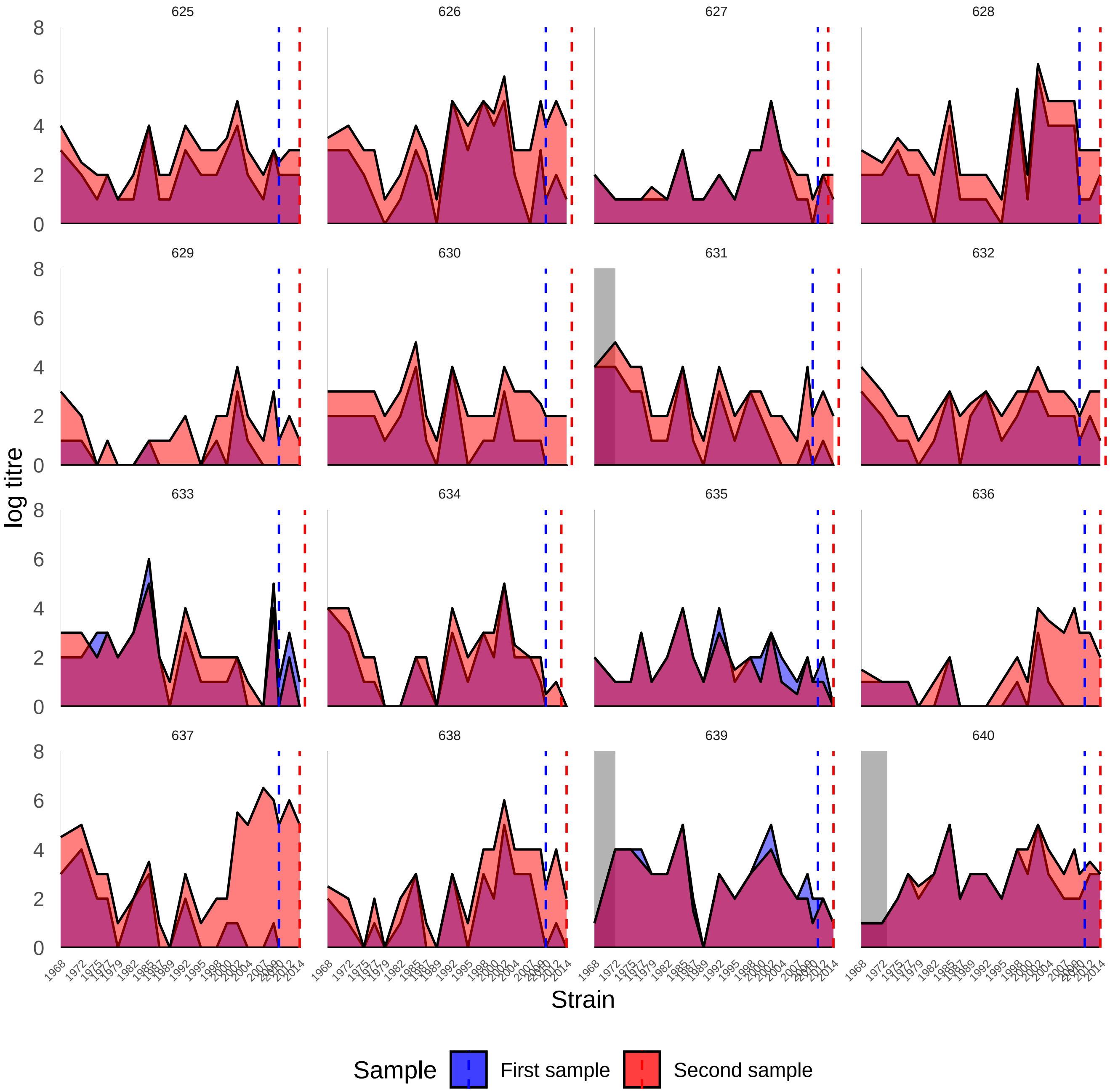
625
626
627
628
629
630
631
632
633
634
635
636
637
638
639
640
log titre
Strain
Sample
First sample
Second sample

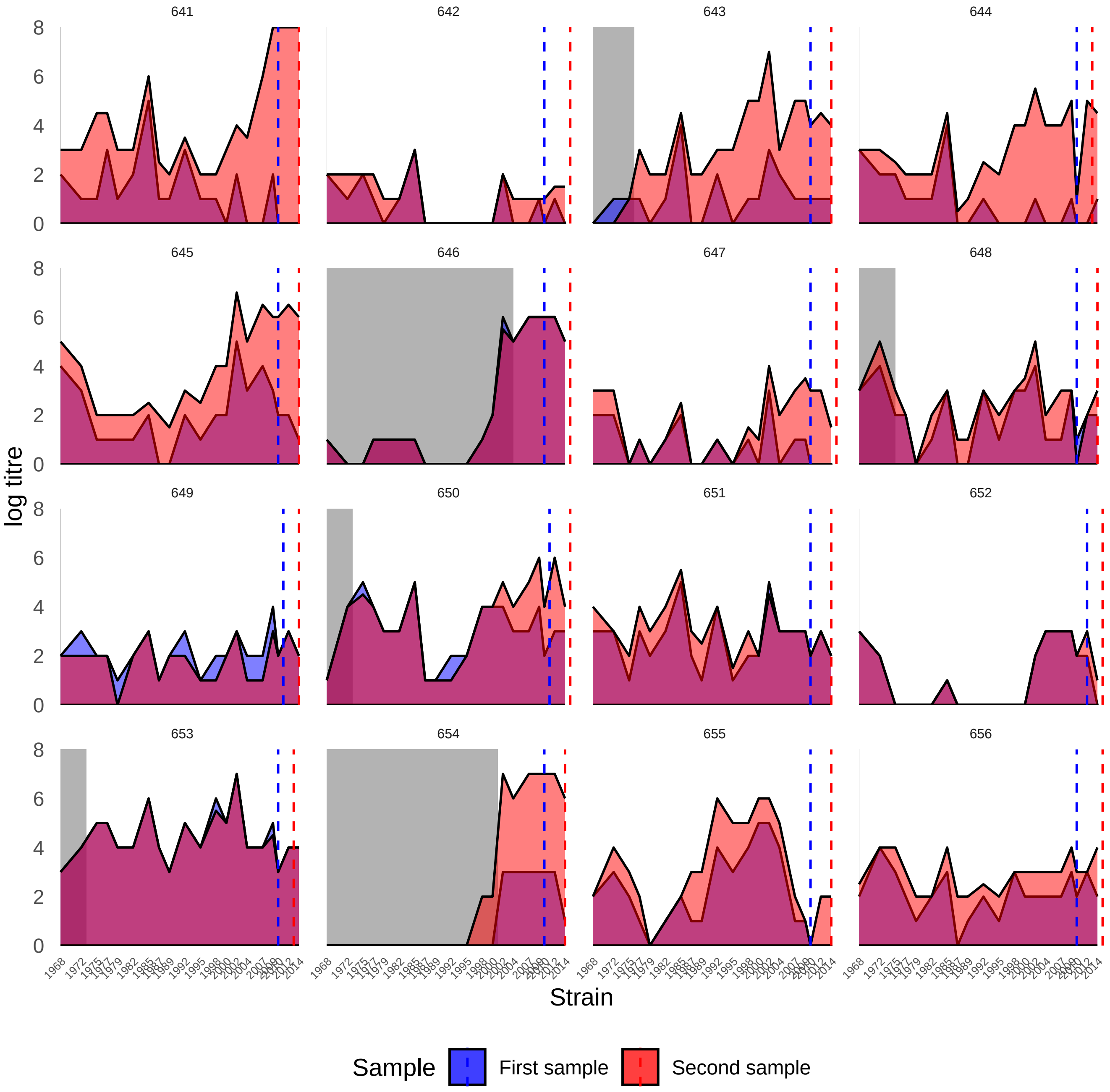

641
642
643
644
645
646
647
648
649
650
651
652
653
654
655
656
log titre
Strain
Sample
First sample
Second sample

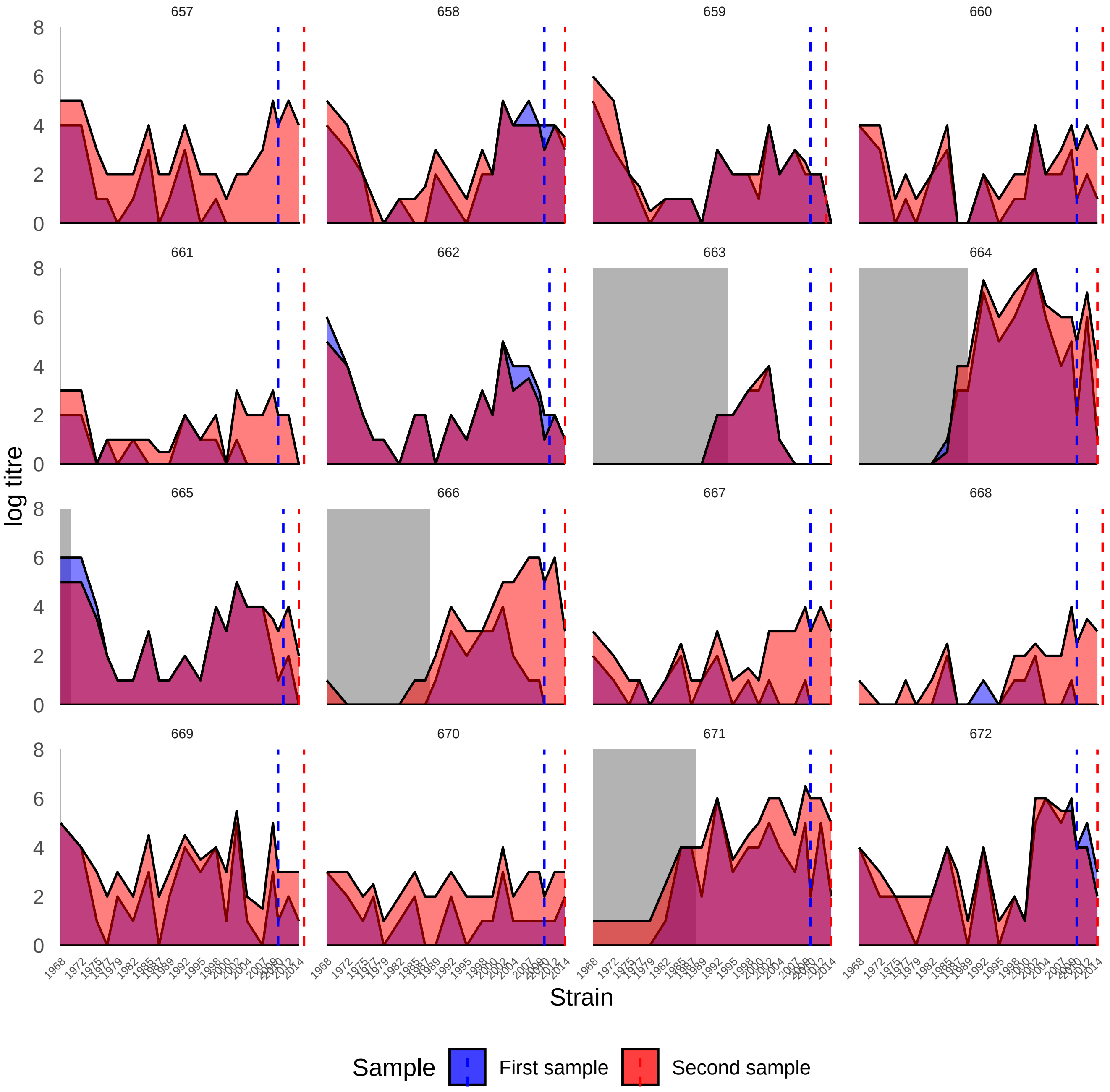
657
658
659
660
661
662
663
664
665
666
667
668
669
670
671
672
log titre
Strain
Sample
First sample
Second sample

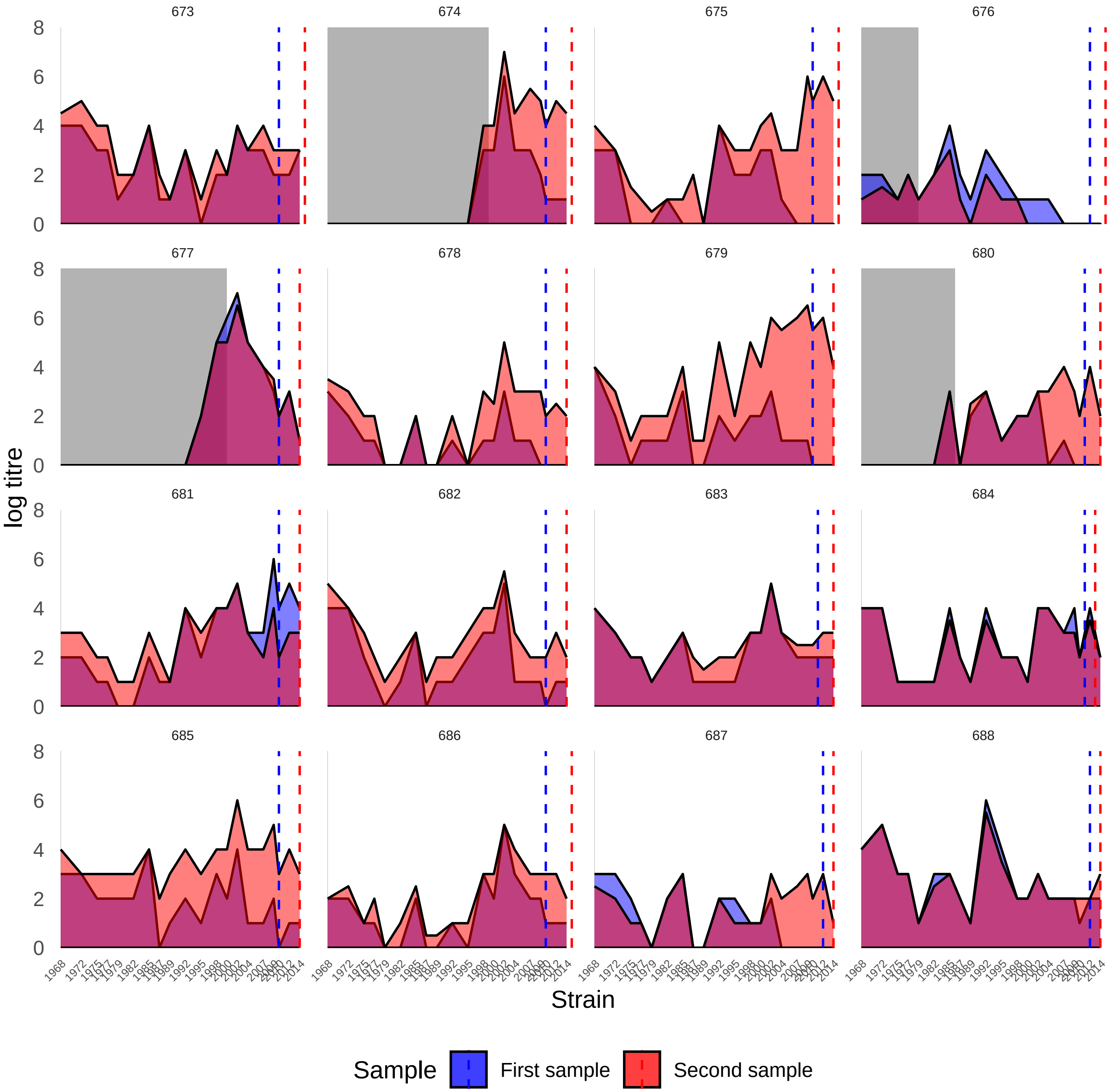
673
674
675
676
677
678
679
680
681
682
683
684
685
686
687
688
log titre
Strain
Sample
First sample
Second sample

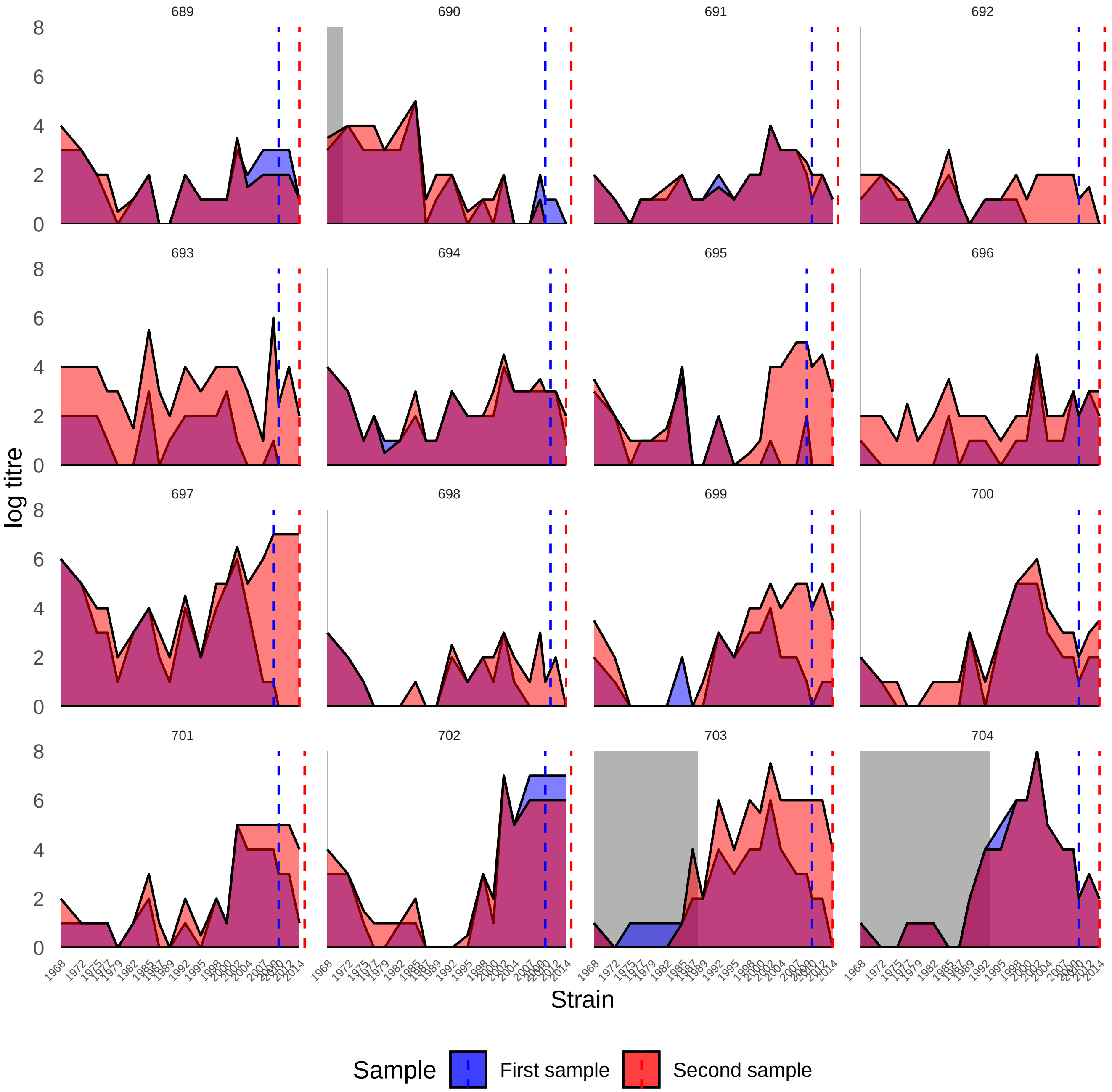

689
690
691
692
693
694
695
696
697
698
699
700
701
702
703
704
log titre
Strain
Sample
First sample
Second sample

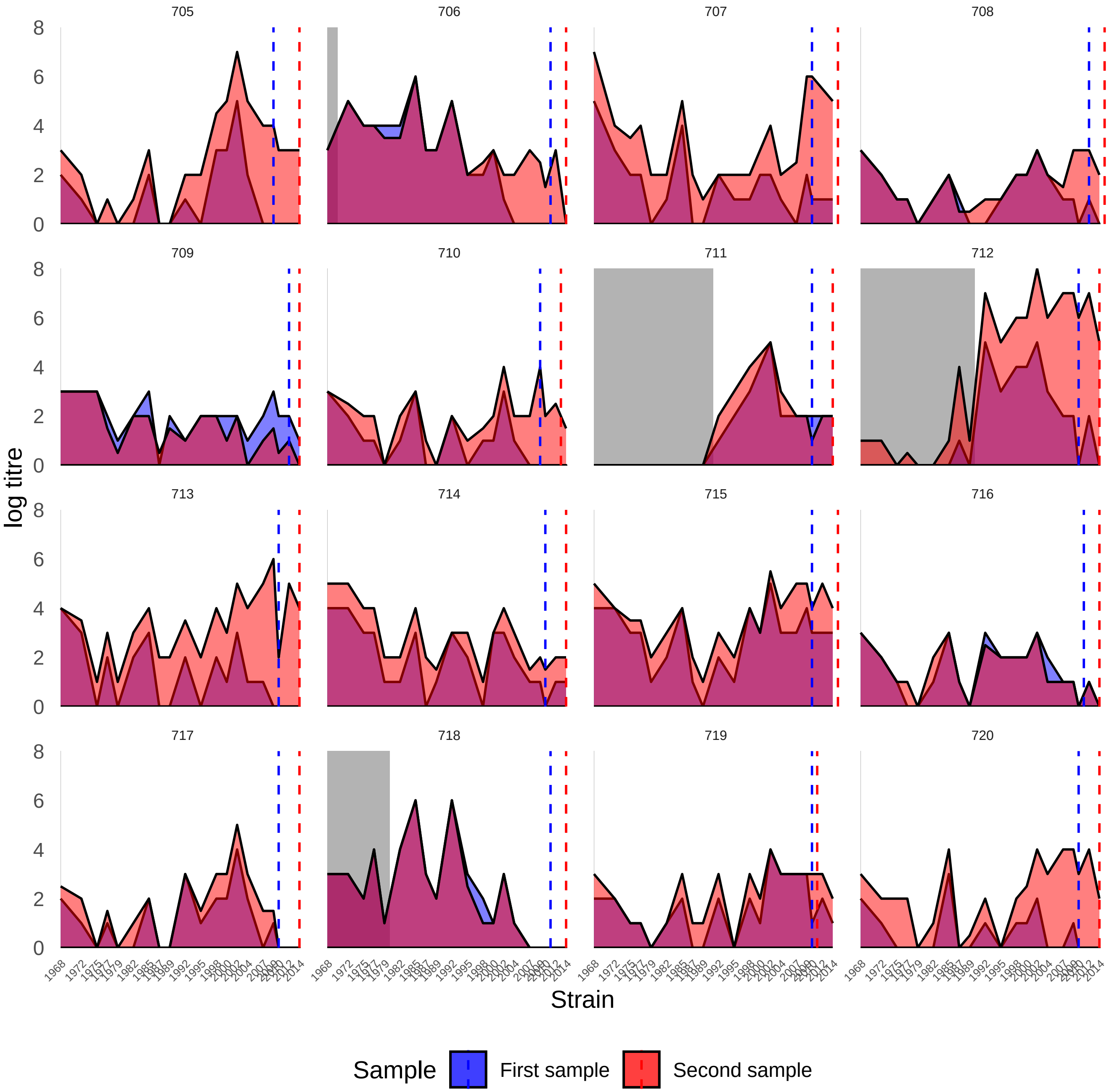
705
706
707
708
709
710
711
712
713
714
715
716
717
718
719
720
log titre
Strain
0
2
4
6
8
1968
1972
1975
1977
1979
1982
1985
1987
1989
1992
1995
1998
2000
2002
2004
2007
2009
2010
2012
2014
Sample
First sample
Second sample

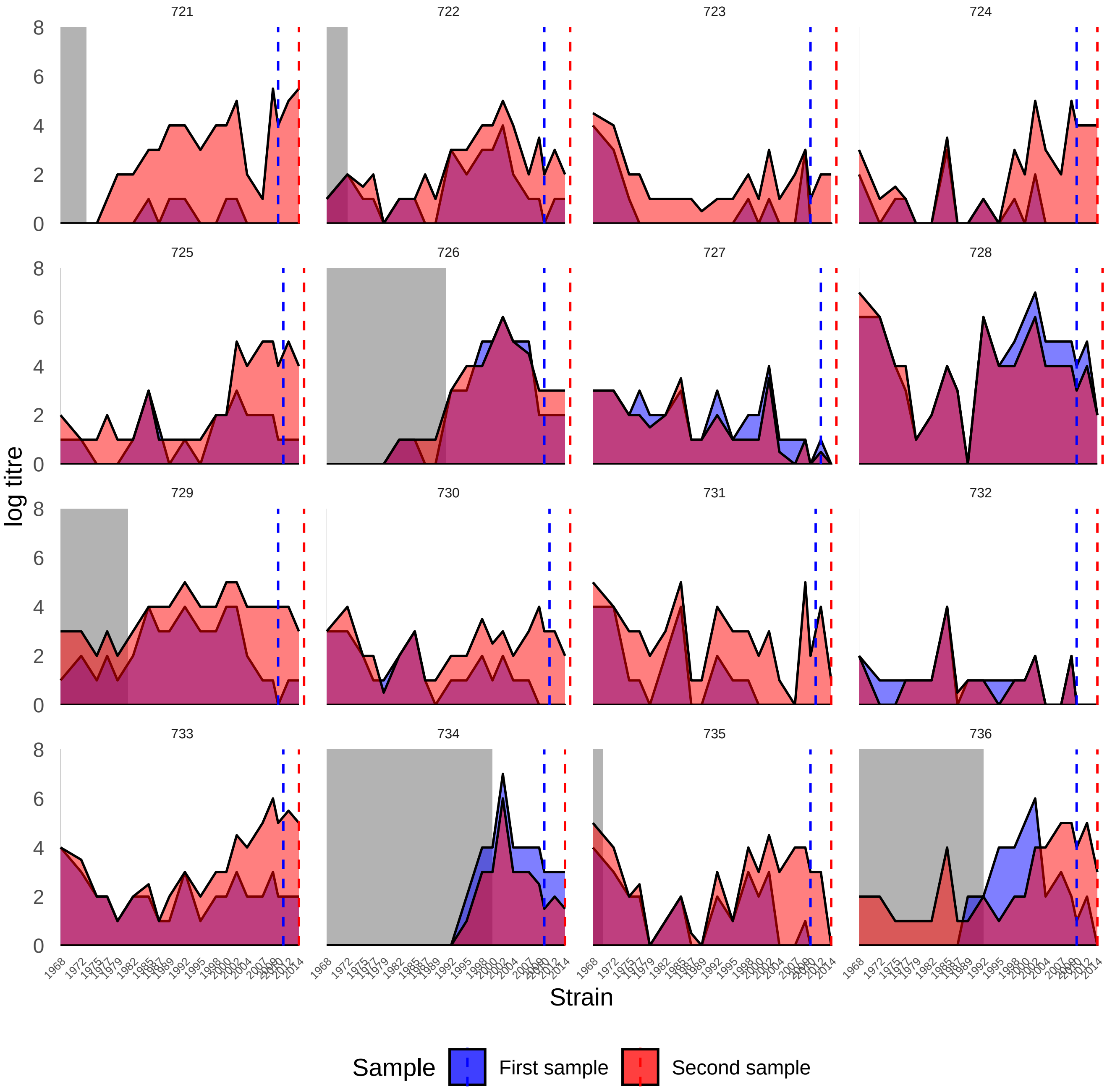

721
722
723
724
725
726
727
728
729
730
731
732
733
734
735
736
log titre
Strain
Sample
First sample
Second sample

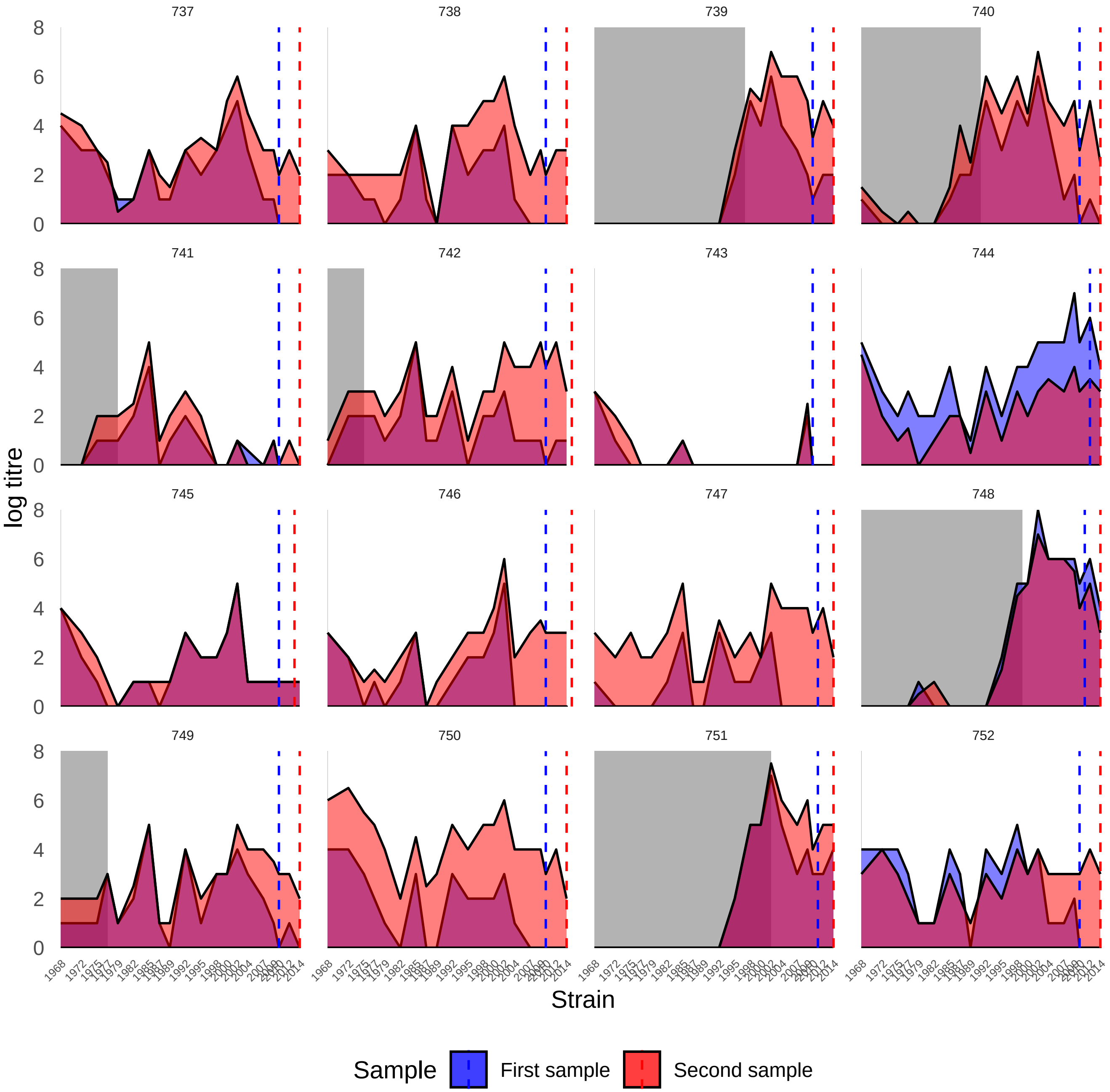
737
738
739
740
741
742
743
744
745
746
747
748
749
750
751
752
log titre
Strain
Sample
First sample
Second sample

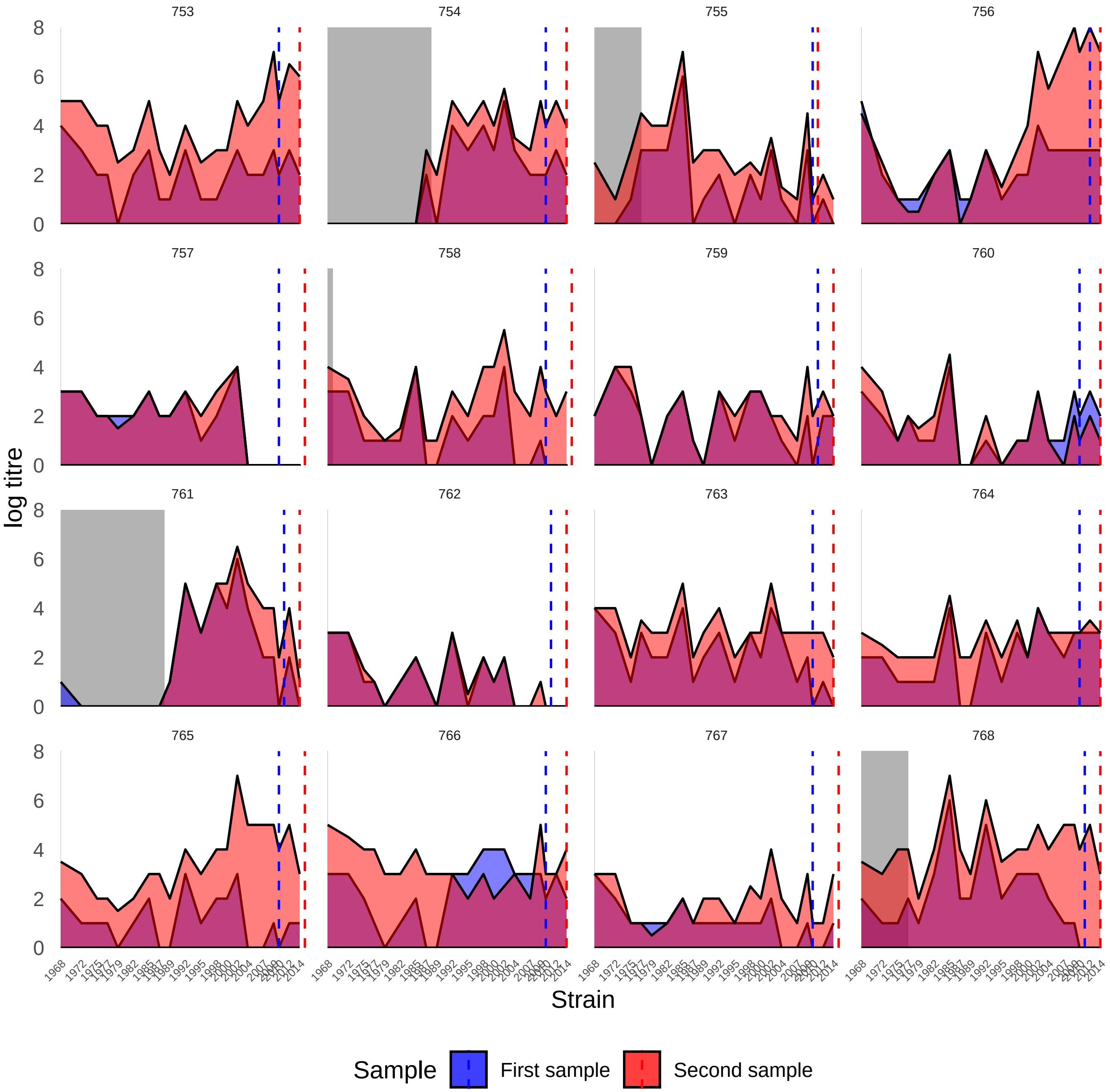

753
754
755
756
757
758
759
760
761
762
763
764
765
766
767
768
log titre
Strain
Sample
First sample
Second sample

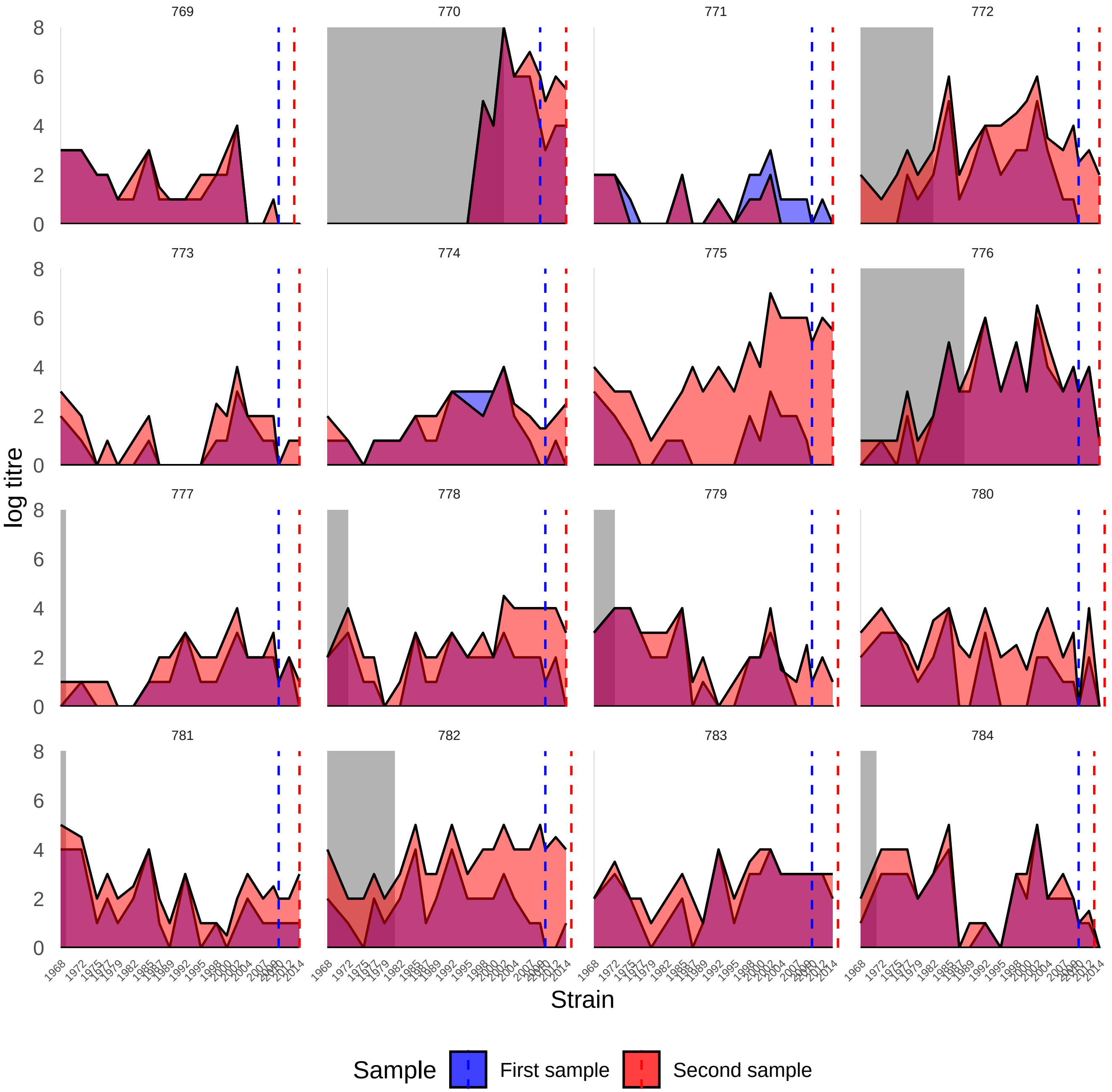

769
770
771
772
773
774
775
776
777
778
779
780
781
782
783
784
log titre
Strain
Sample
First sample
Second sample

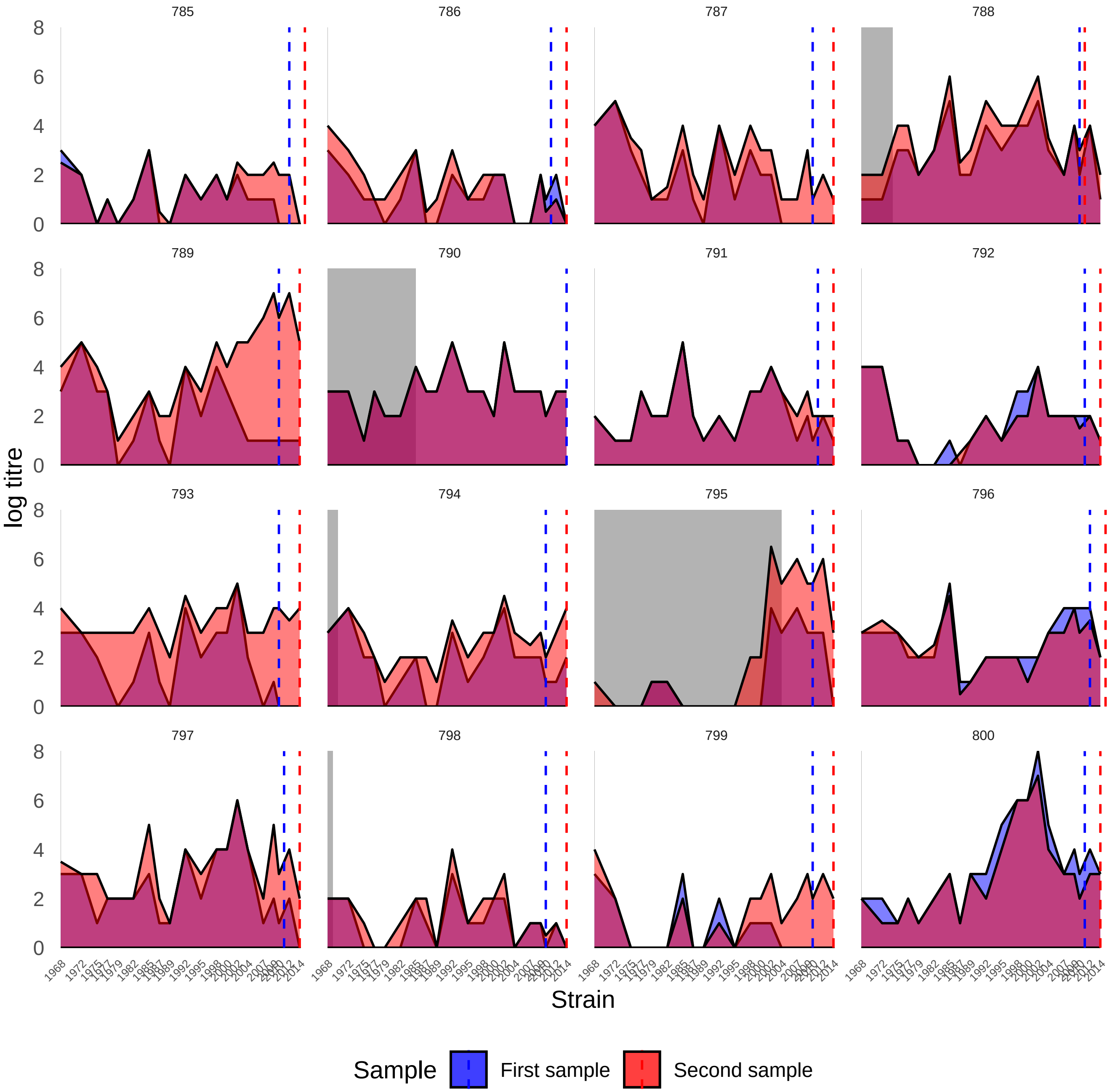

785
786
787
788
789
790
791
792
793
794
795
796
797
798
799
800
log titre
Strain
Sample
First sample
Second sample

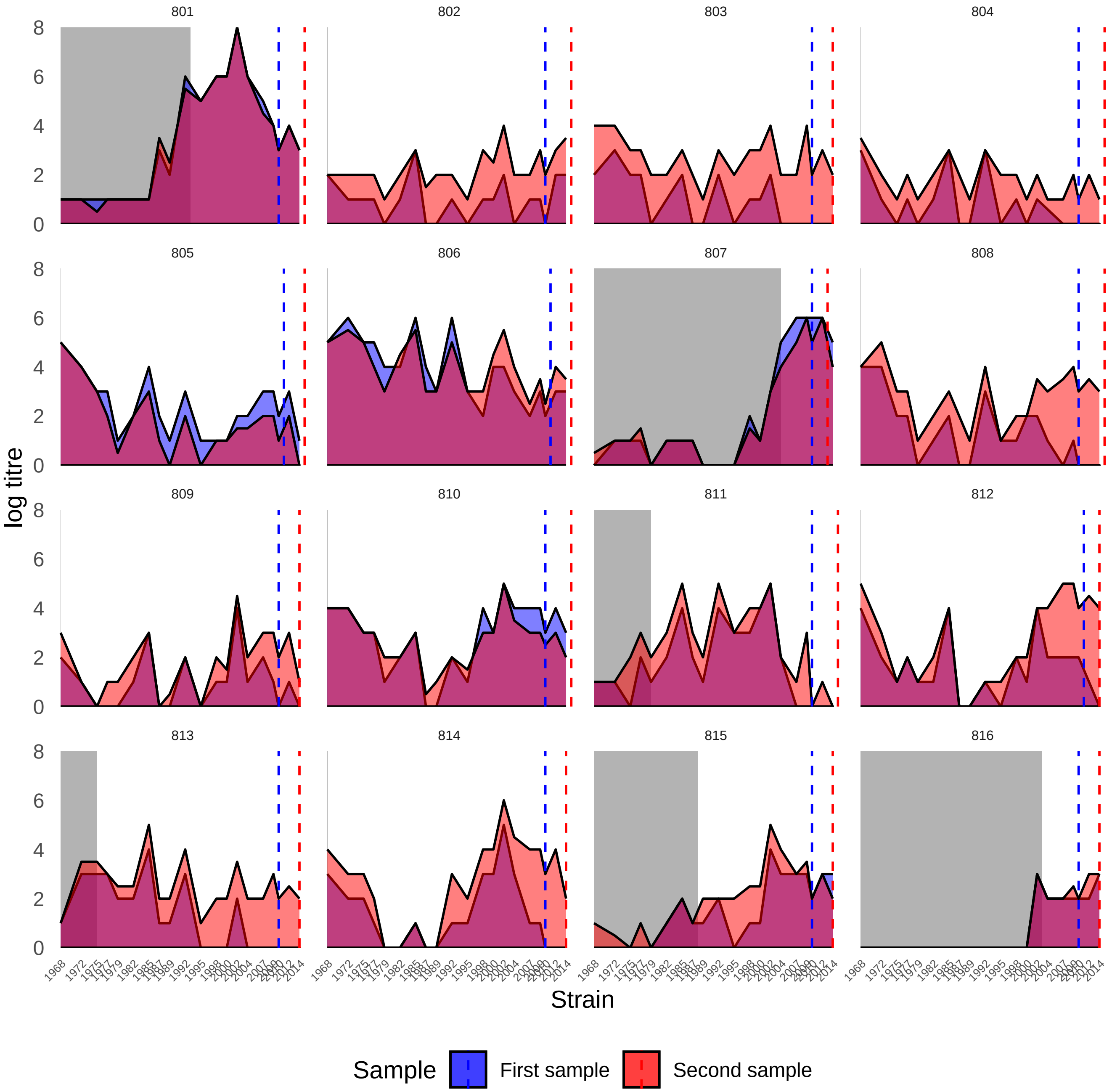

801
802
803
804
805
806
807
808
809
810
811
812
813
814
815
816
log titre
Strain
0
2
4
6
8
1968
1972
1975
1977
1979
1982
1985
1987
1989
1992
1995
1998
2000
2002
2004
2007
2009
2010
2012
2014
Sample
First sample
Second sample

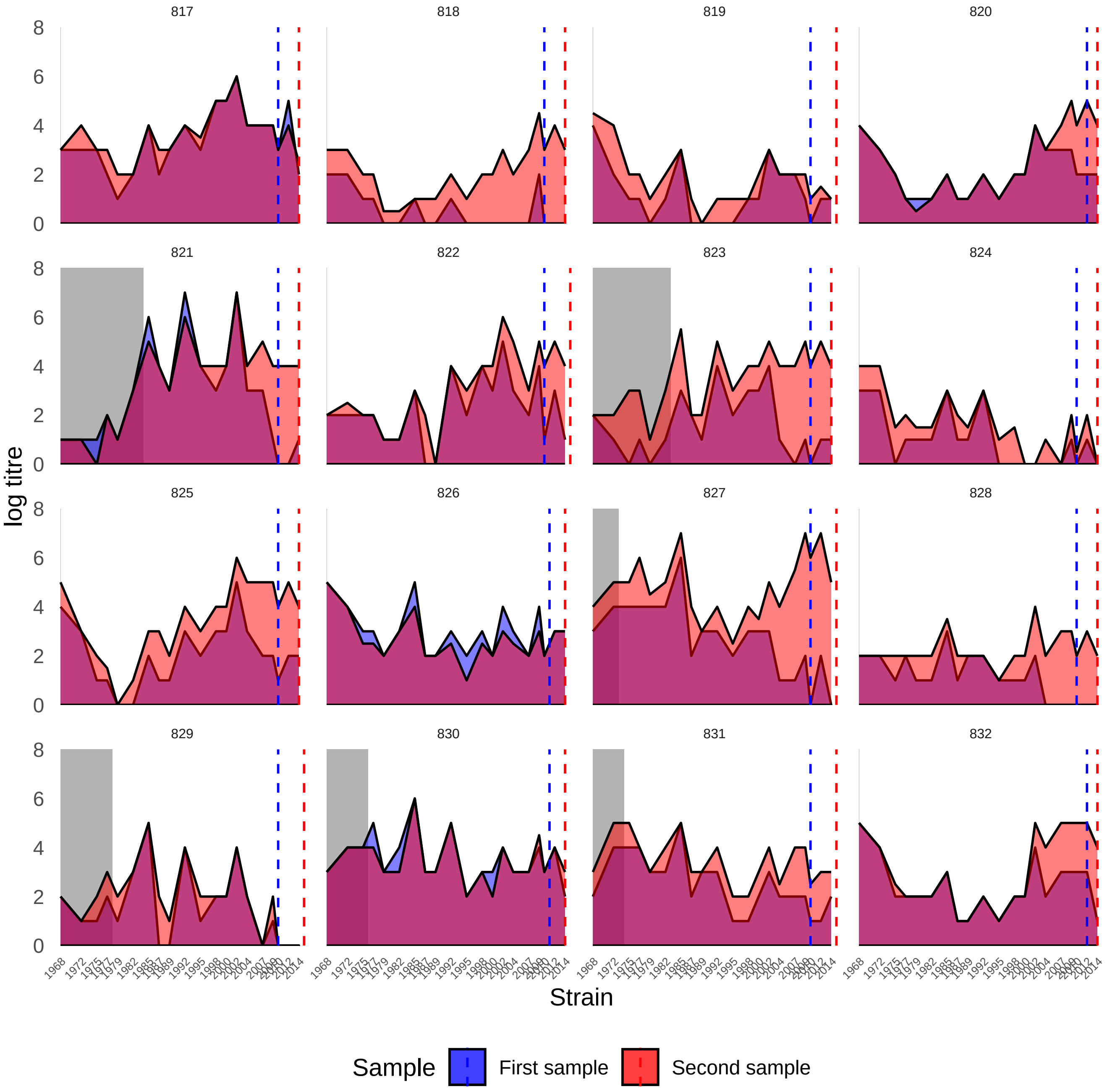

817
818
819
820
821
822
823
824
825
826
827
828
829
830
831
832
log titre
Strain
Sample
First sample
Second sample

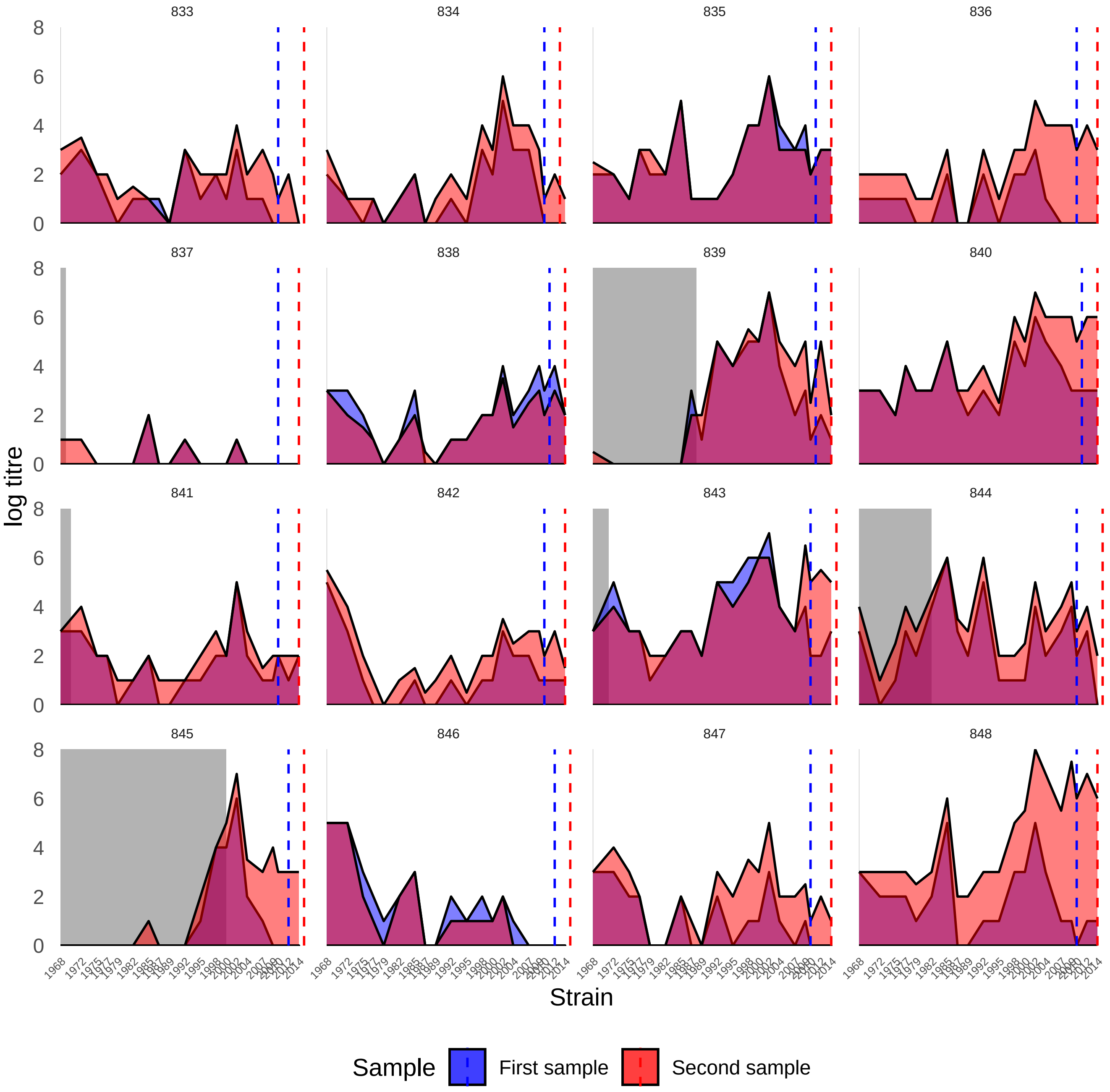

833
834
835
836
837
838
839
840
841
842
843
844
845
846
847
848
log titre
Strain
Sample
First sample
Second sample

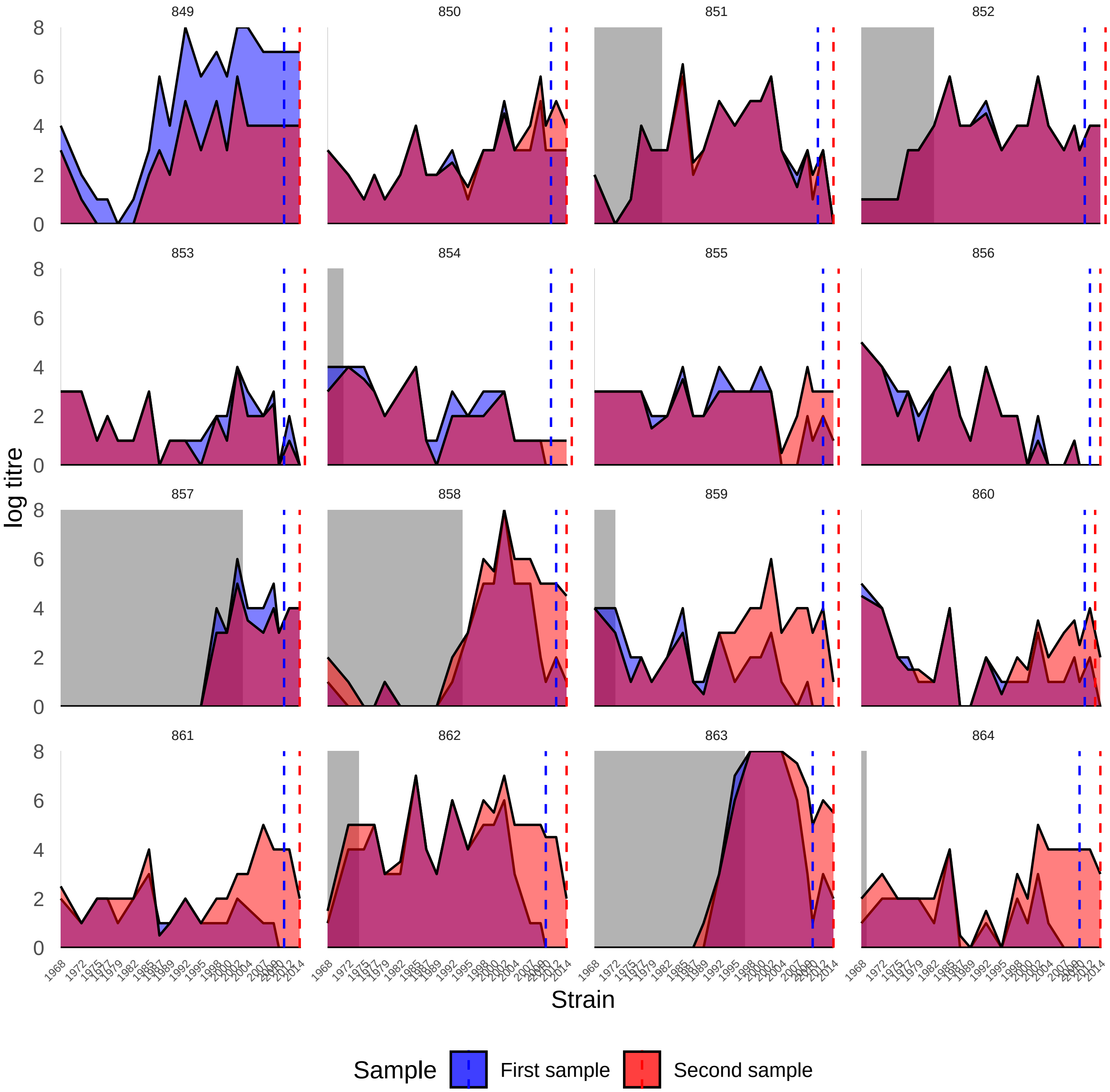

849
850
851
852
853
854
855
856
857
858
859
860
861
862
863
864
log titre
Strain
Sample
First sample
Second sample

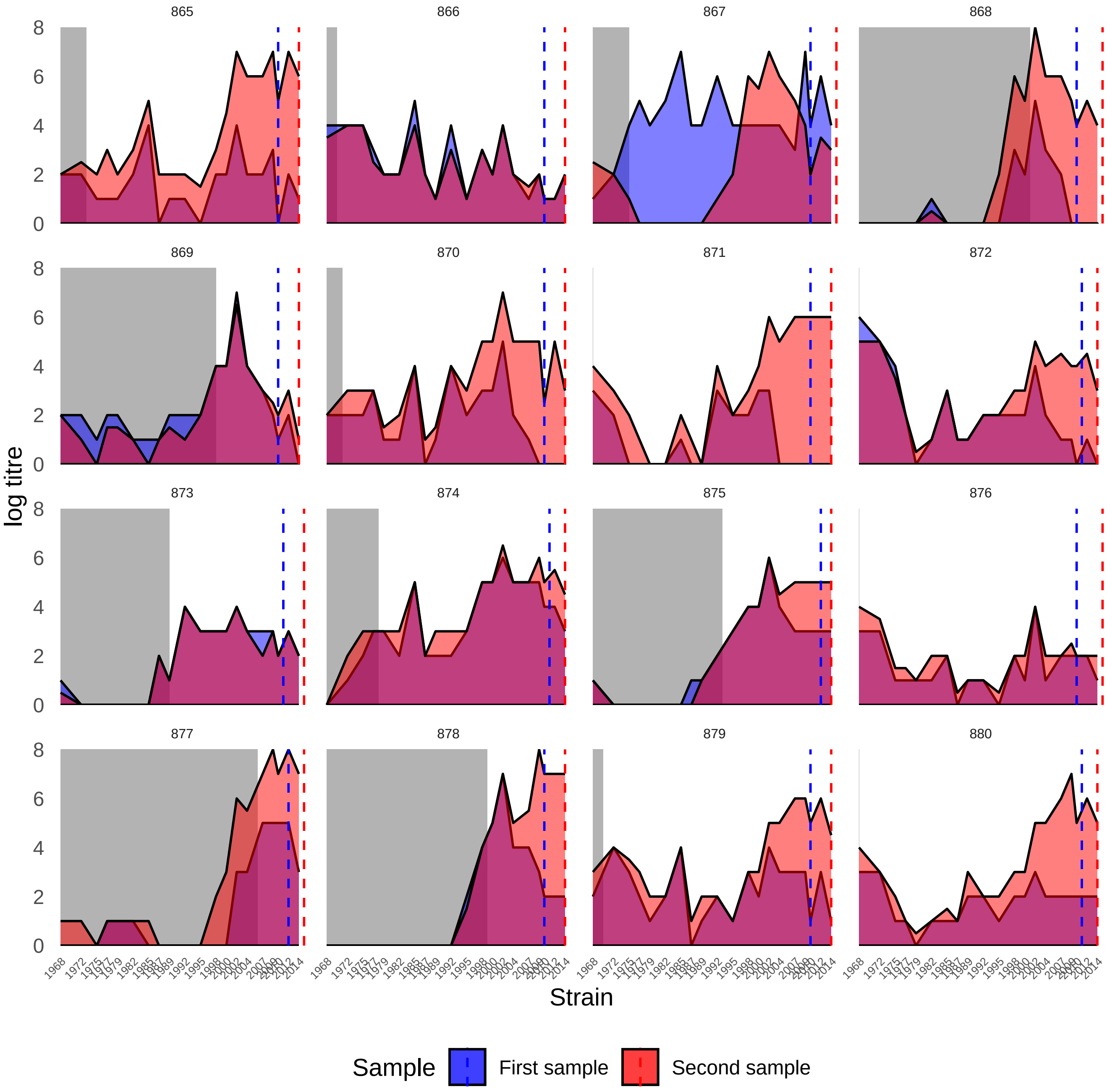

865
866
867
868
869
870
871
872
873
874
875
876
877
878
879
880
log titre
0
2
4
6
8
Strain
1968
1972
1975
1977
1979
1982
1985
1987
1989
1992
1995
1998
2000
2002
2004
2007
2009
2010
2012
2014
Sample
First sample
Second sample

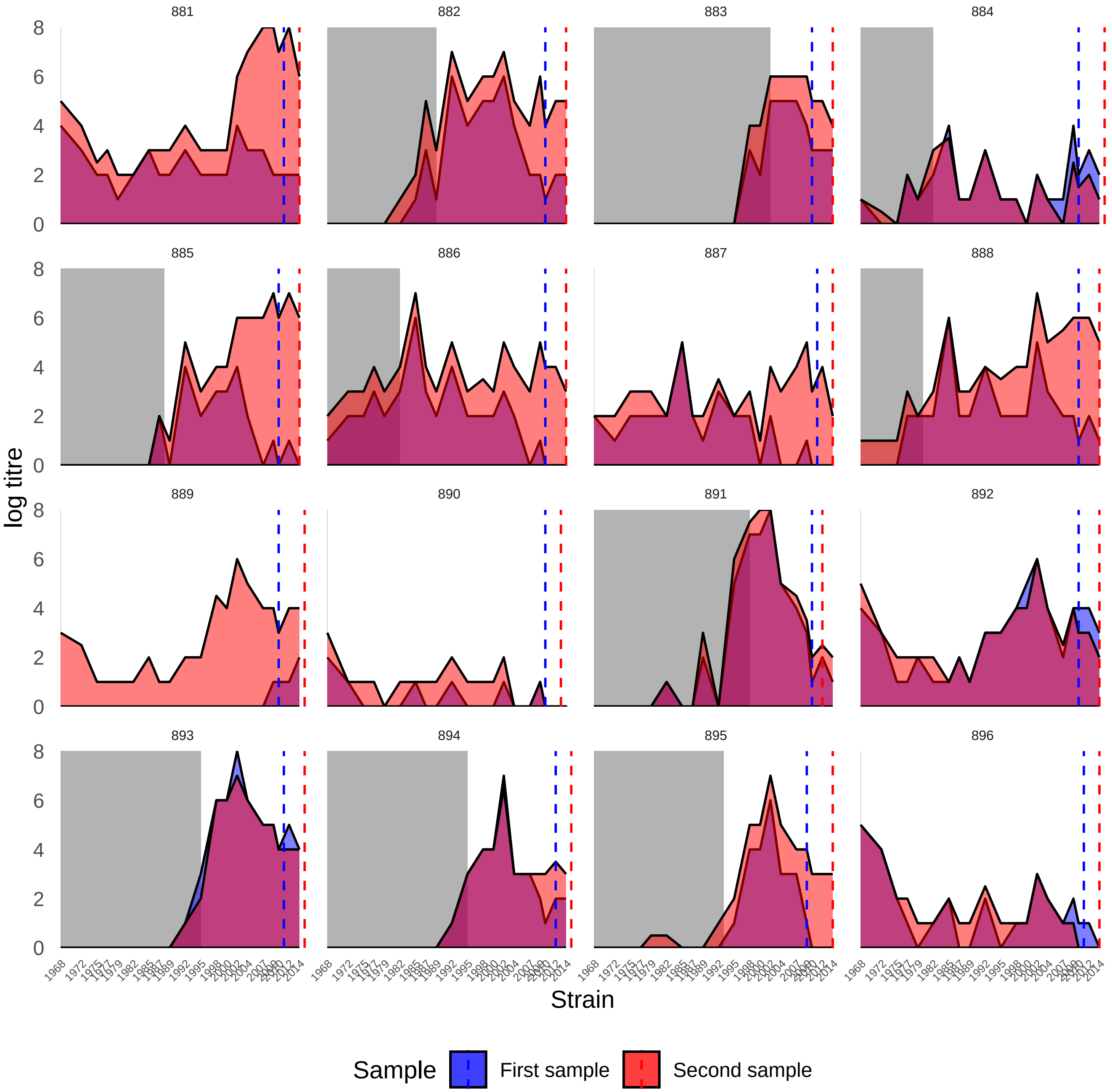
881
882
883
884
885
886
887
888
889
890
891
892
893
894
895
896
log titre
Strain
Sample
First sample
Second sample

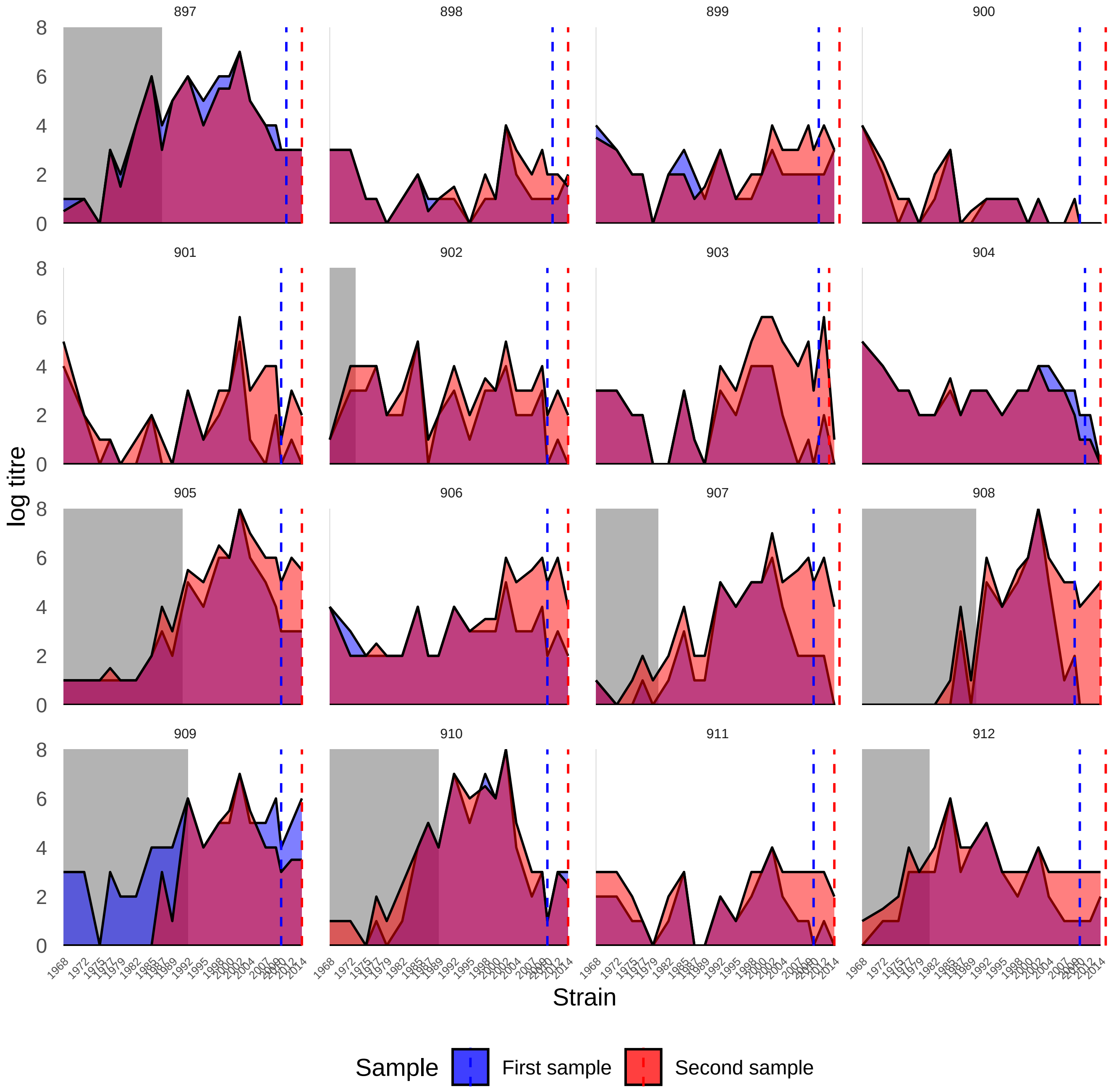

897
898
899
900
901
902
903
904
905
906
907
908
909
910
911
912
log titre
Strain
Sample
First sample
Second sample

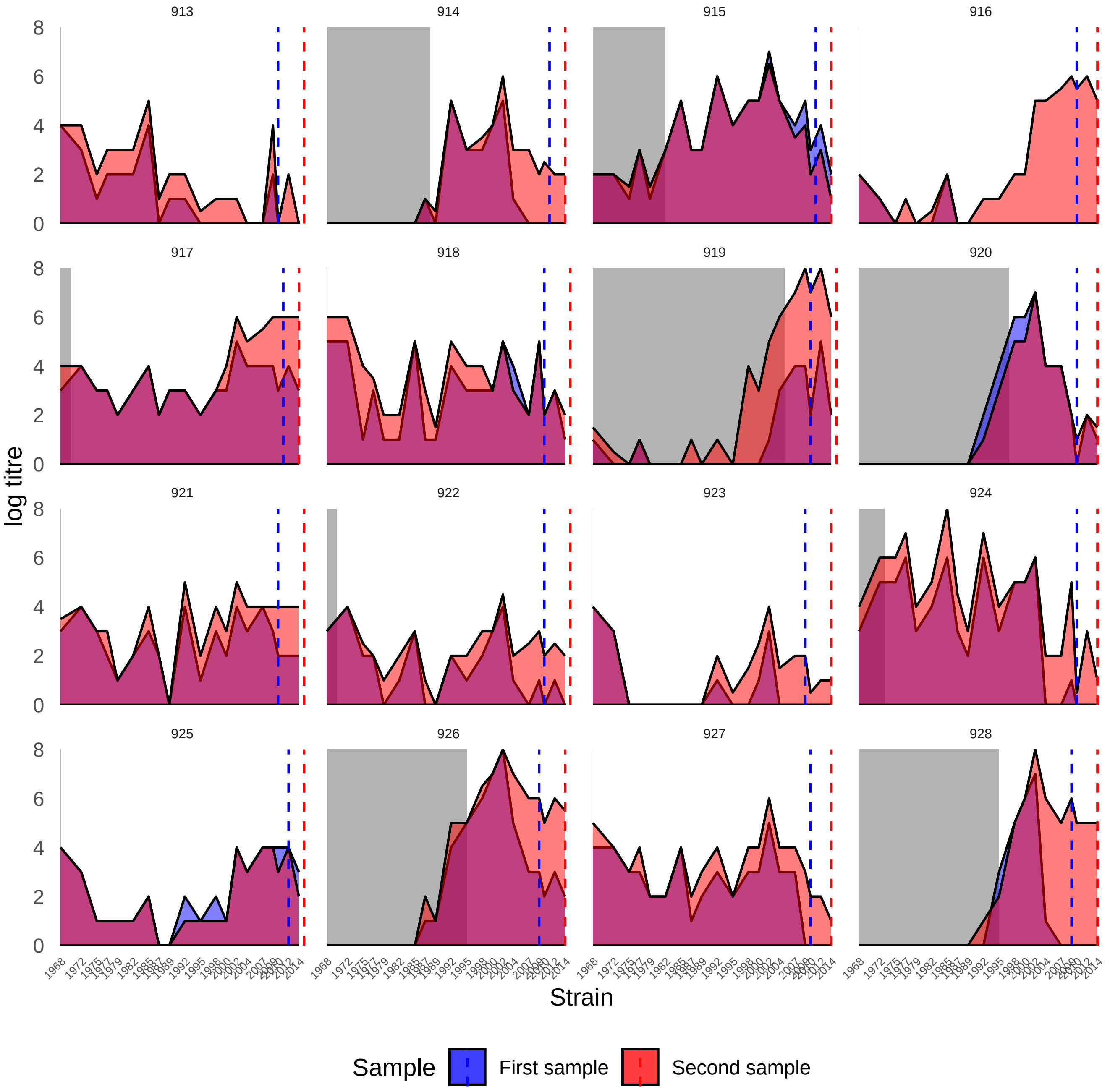

913
914
915
916
917
918
919
920
921
922
923
924
925
926
927
928
log titre
0
2
4
6
8
1968
1972
1975
1977
1979
1982
1985
1987
1989
1992
1995
1998
2000
2002
2004
2007
2009
2010
2012
2014
Strain
Sample
First sample
Second sample

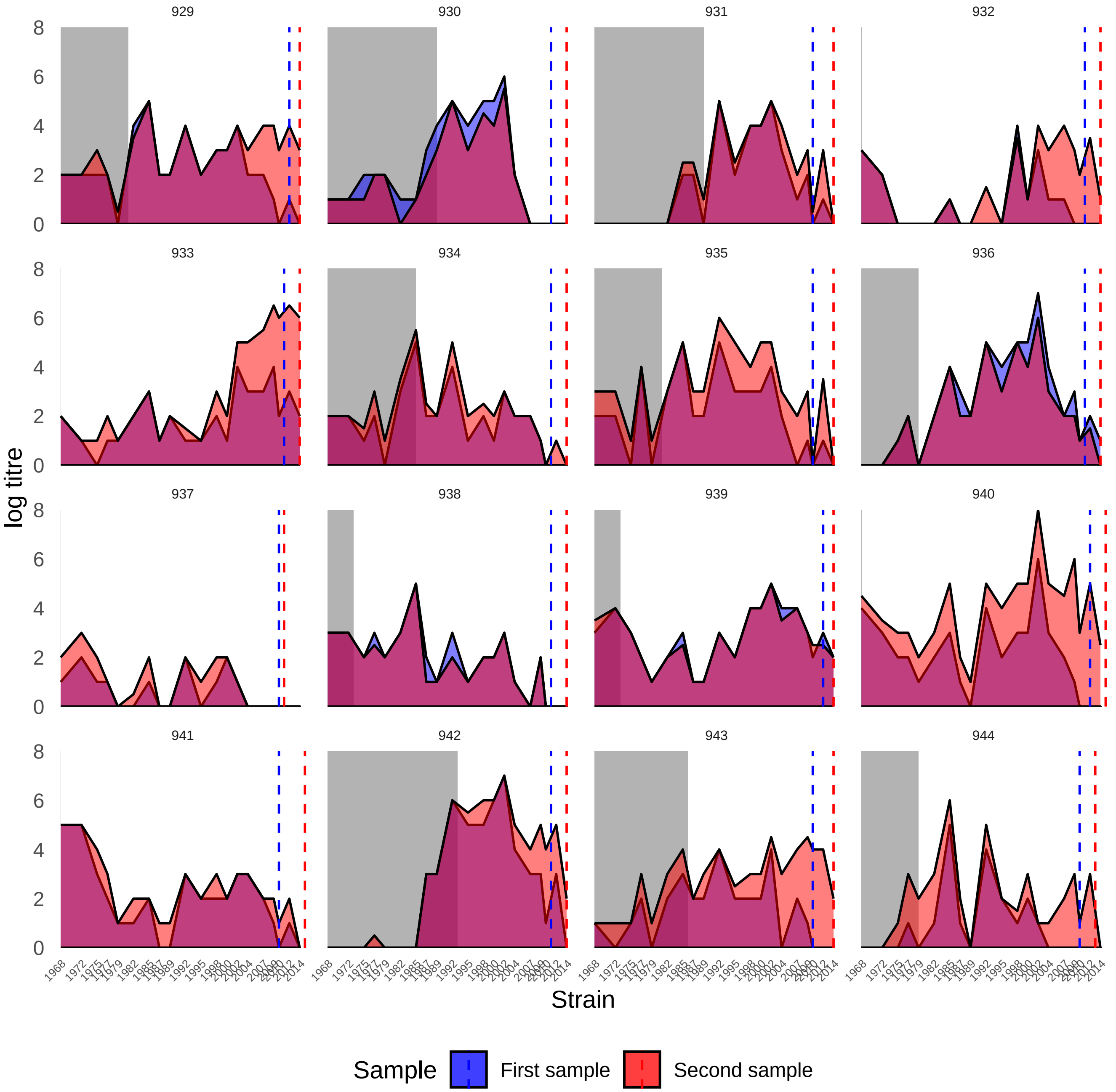
929
930
931
932
933
934
935
936
937
938
939
940
941
942
943
944
log titre
Strain
0
2
4
6
8
1968
1972
1975
1977
1979
1982
1985
1987
1989
1992
1995
1998
2000
2002
2004
2007
2009
2010
2012
2014
Sample
First sample
Second sample

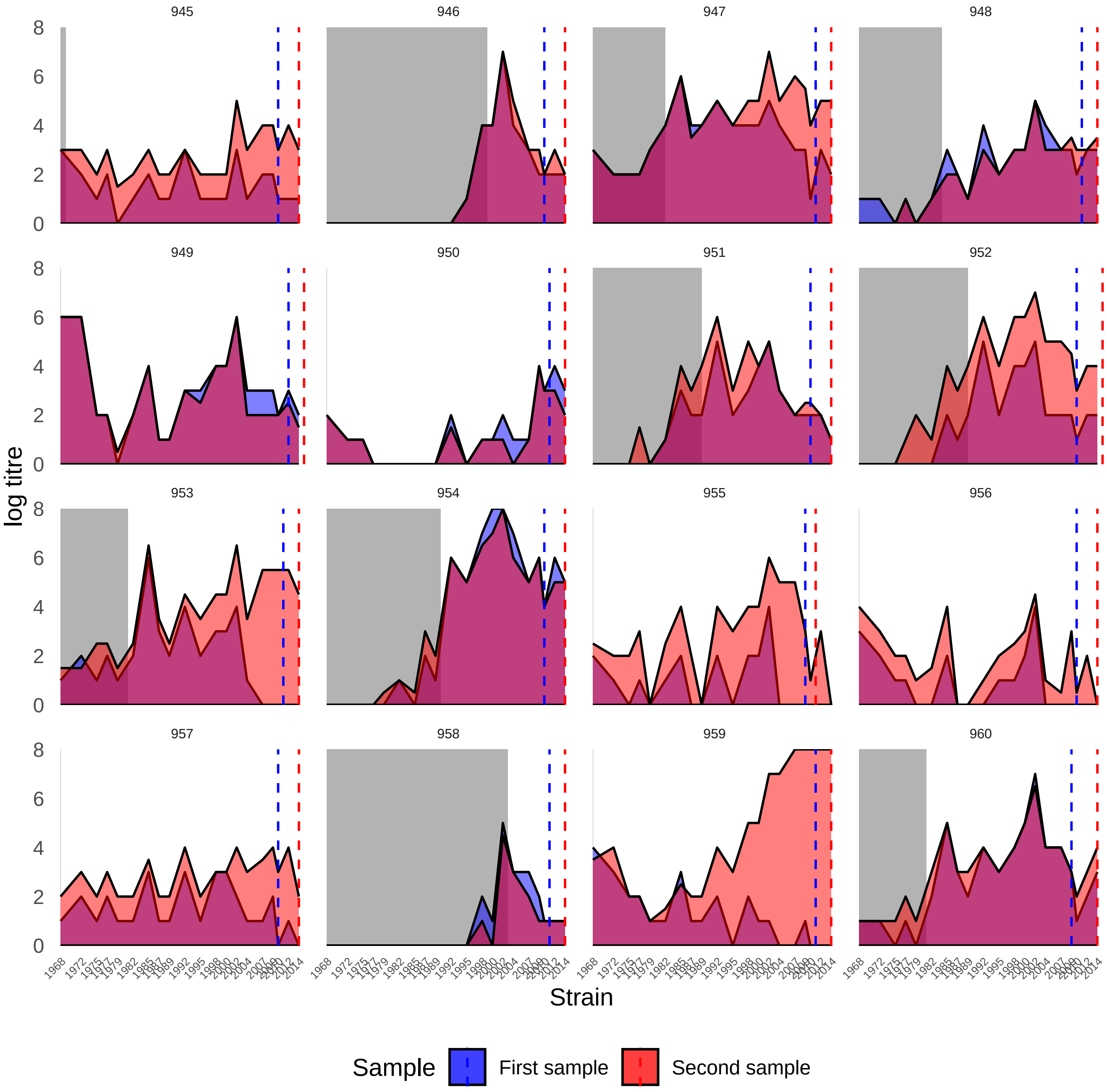
945
946
947
948
949
950
951
952
953
954
955
956
957
958
959
960
log titre
Strain
Sample
First sample
Second sample

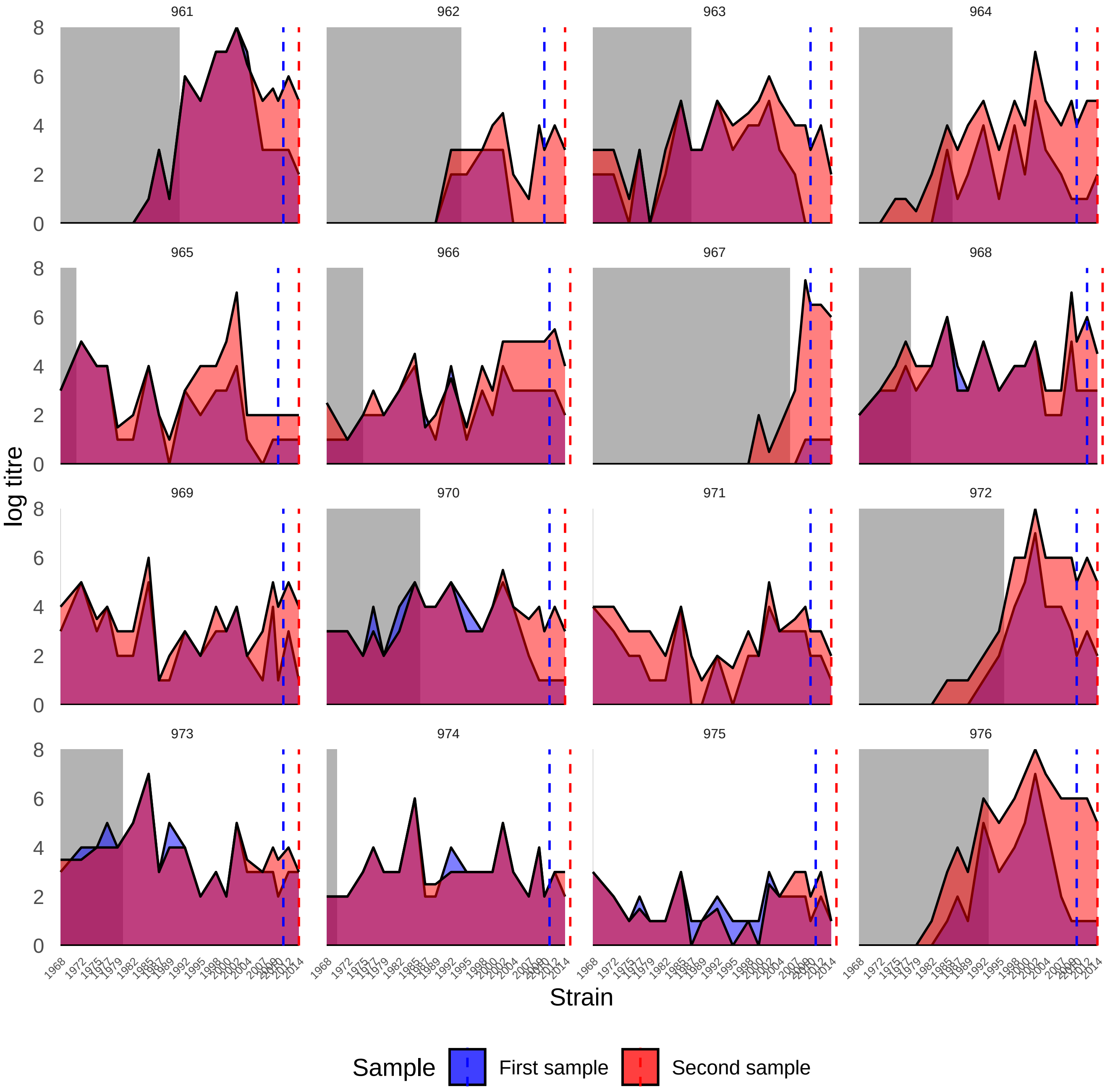
961
962
963
964
965
966
967
968
969
970
971
972
973
974
975
976
log titre
Strain
Sample
First sample
Second sample

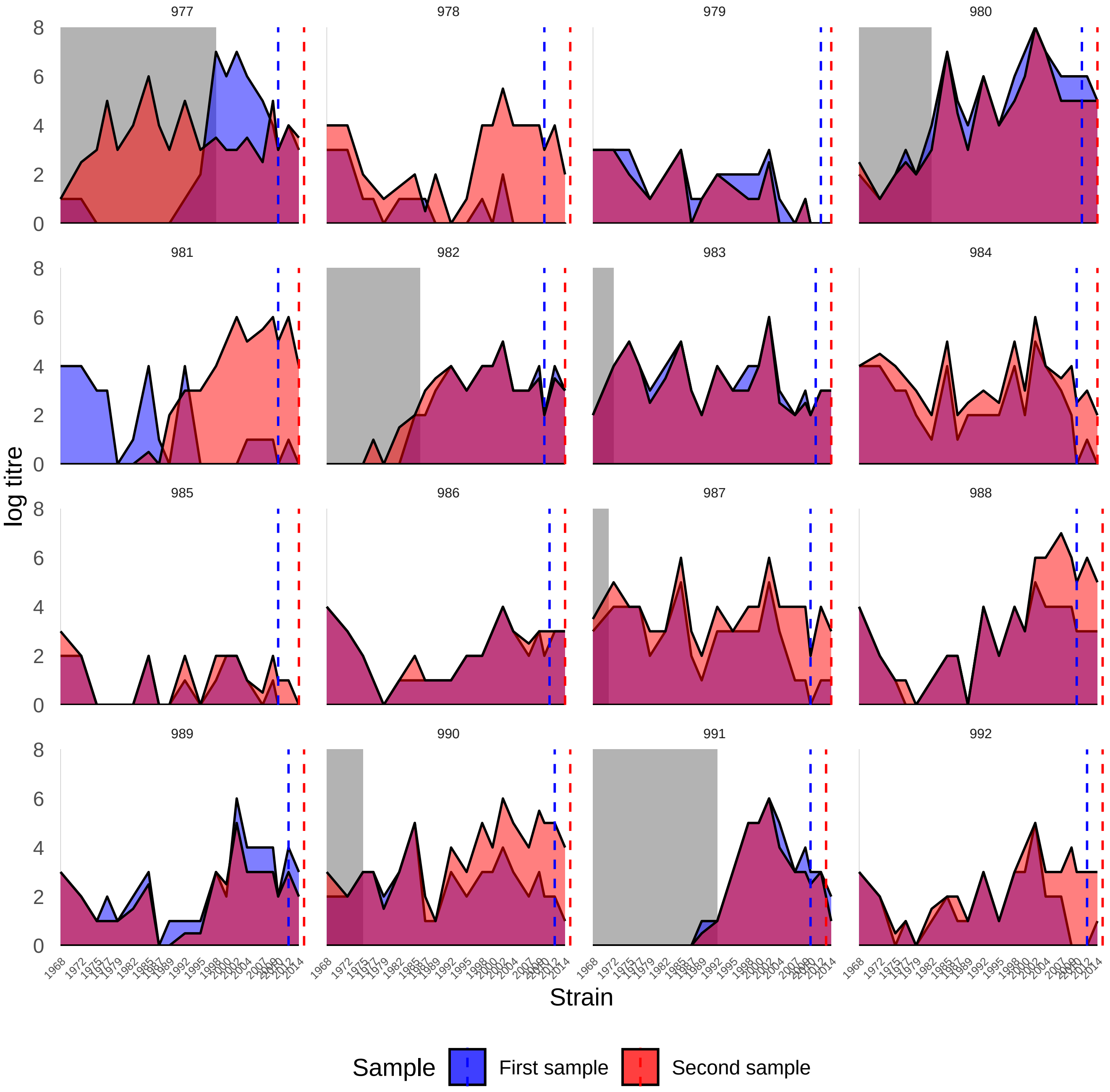
977
978
979
980
981
982
983
984
985
986
987
988
989
990
991
992
log titre
0
2
4
6
8
Strain
1968
1972
1975
1977
1979
1982
1985
1987
1989
1992
1995
1998
2000
2002
2004
2007
2009
2010
2012
2014
Sample
First sample
Second sample

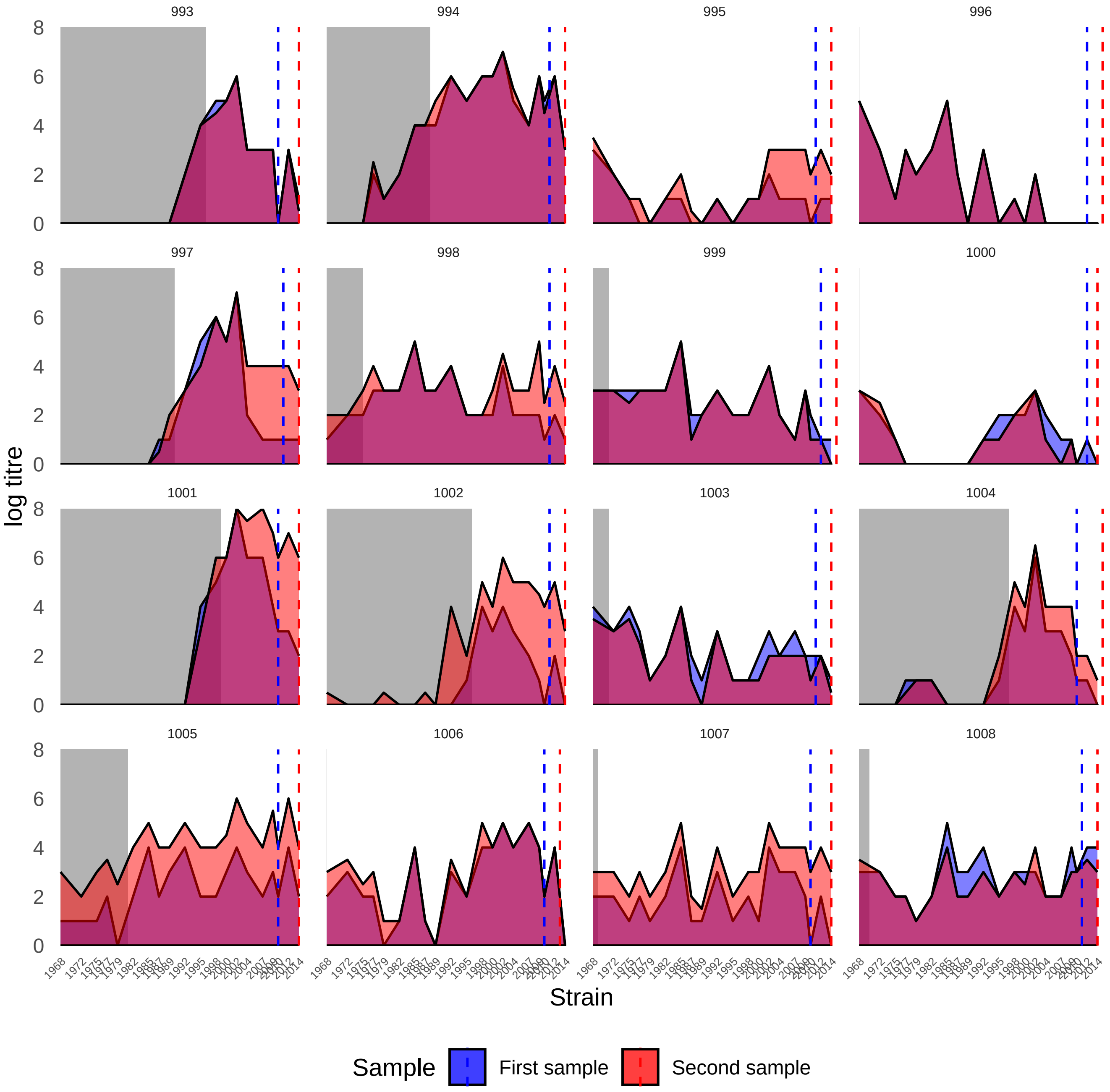
993
994
995
996
997
998
999
1000
1001
1002
1003
1004
1005
1006
1007
1008
log titre
Strain
Sample
First sample
Second sample

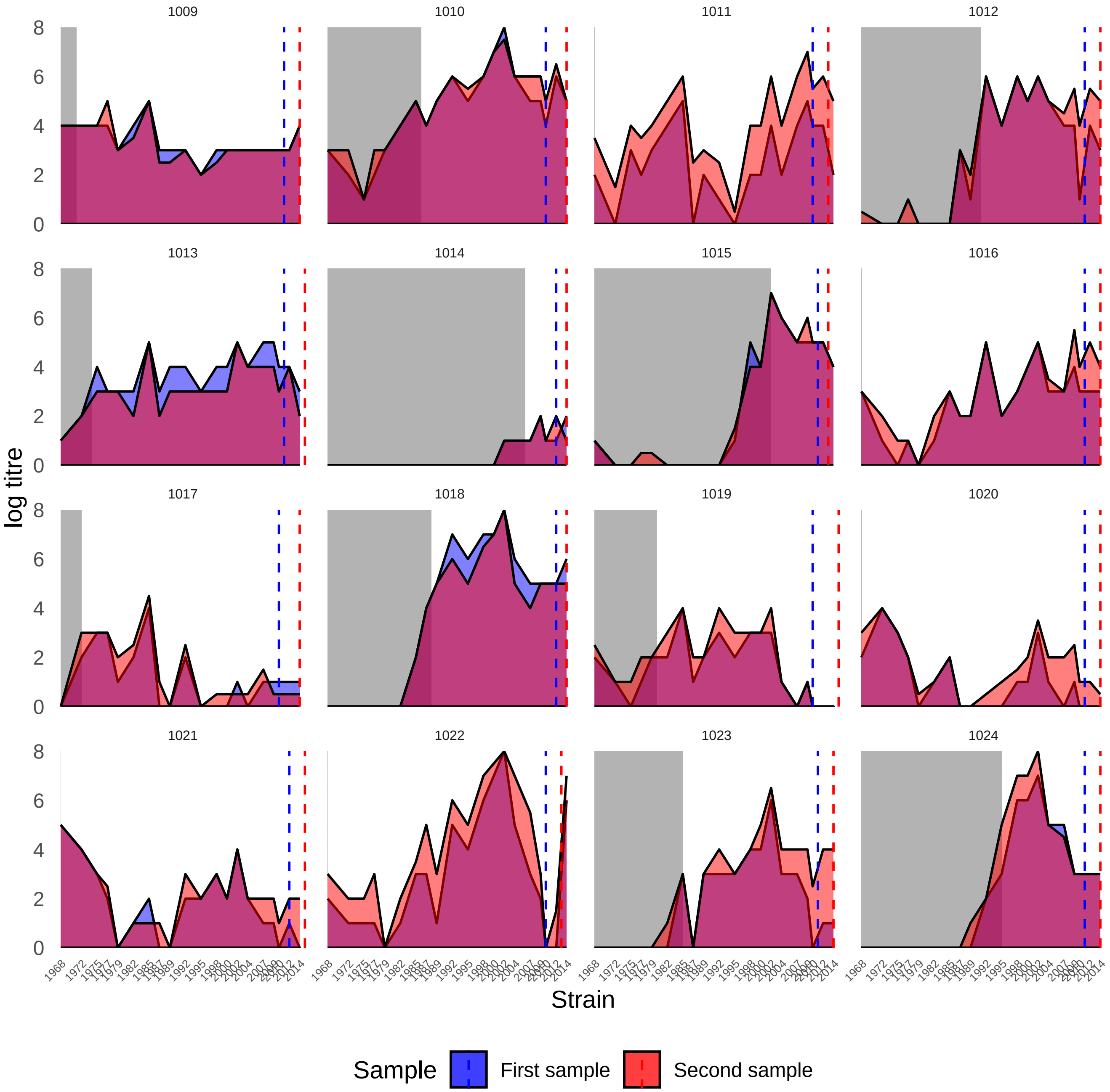

1009
1010
1011
1012
1013
1014
1015
1016
1017
1018
1019
1020
1021
1022
1023
1024
log titre
Strain
Sample
First sample
Second sample

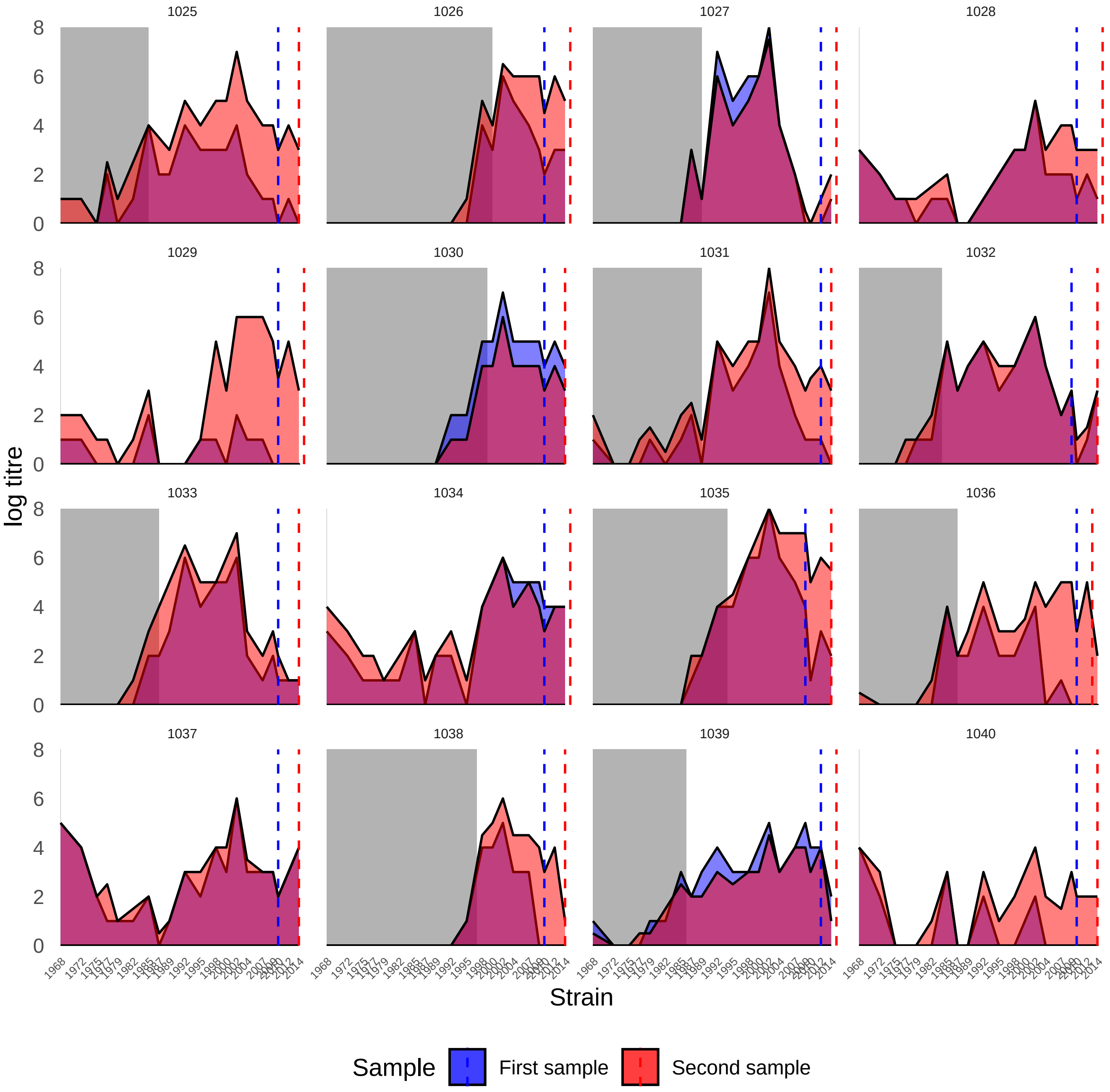
1025
1026
1027
1028
1029
1030
1031
1032
1033
1034
1035
1036
1037
1038
1039
1040
log titre
0
2
4
6
8
1968
1972
1975
1977
1979
1982
1985
1987
1989
1992
1995
1998
2000
2002
2004
2007
2009
2010
2012
2014
Strain
Sample
First sample
Second sample

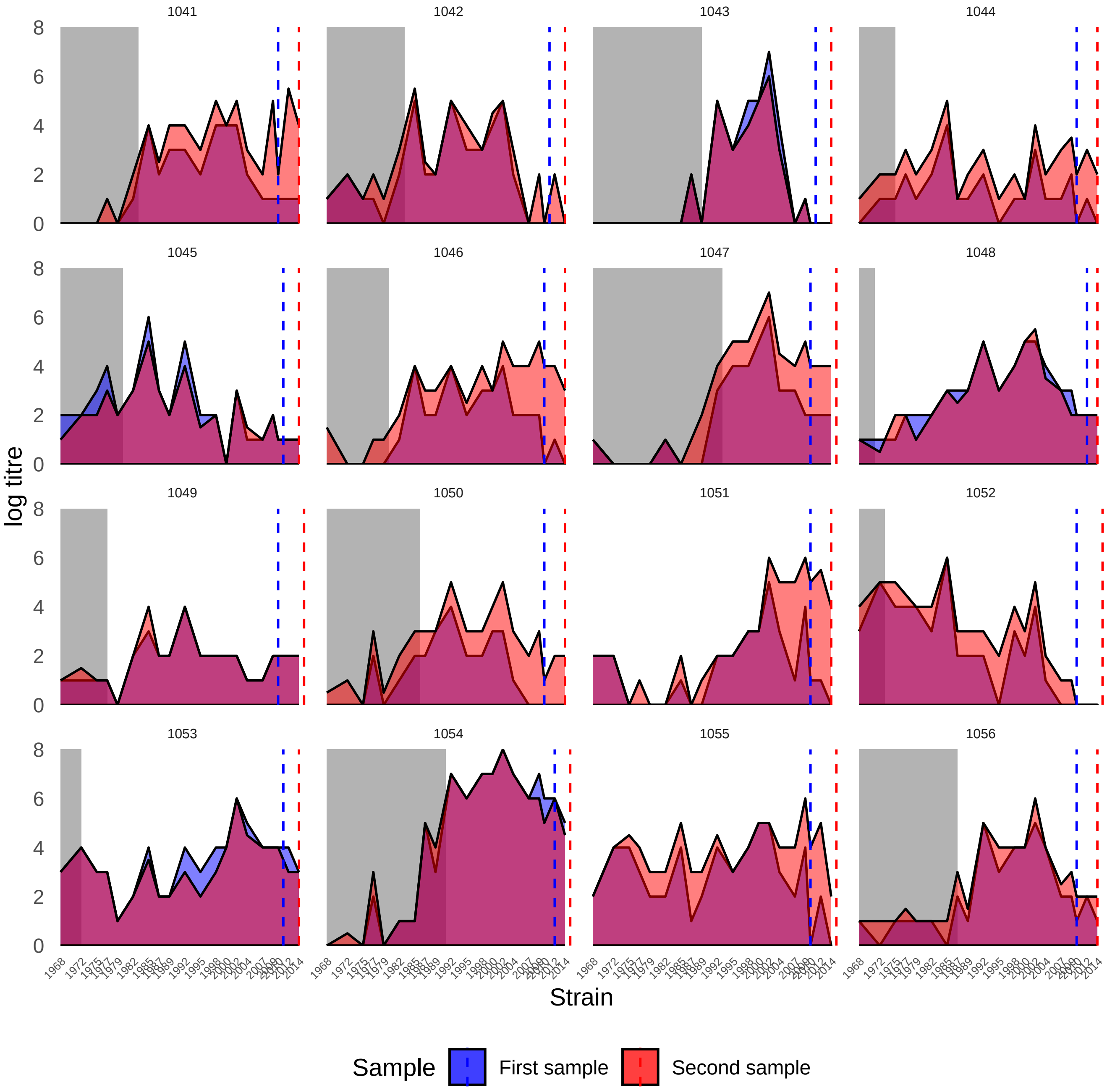

1041
1042
1043
1044
1045
1046
1047
1048
1049
1050
1051
1052
1053
1054
1055
1056
log titre
Strain
Sample
First sample
Second sample

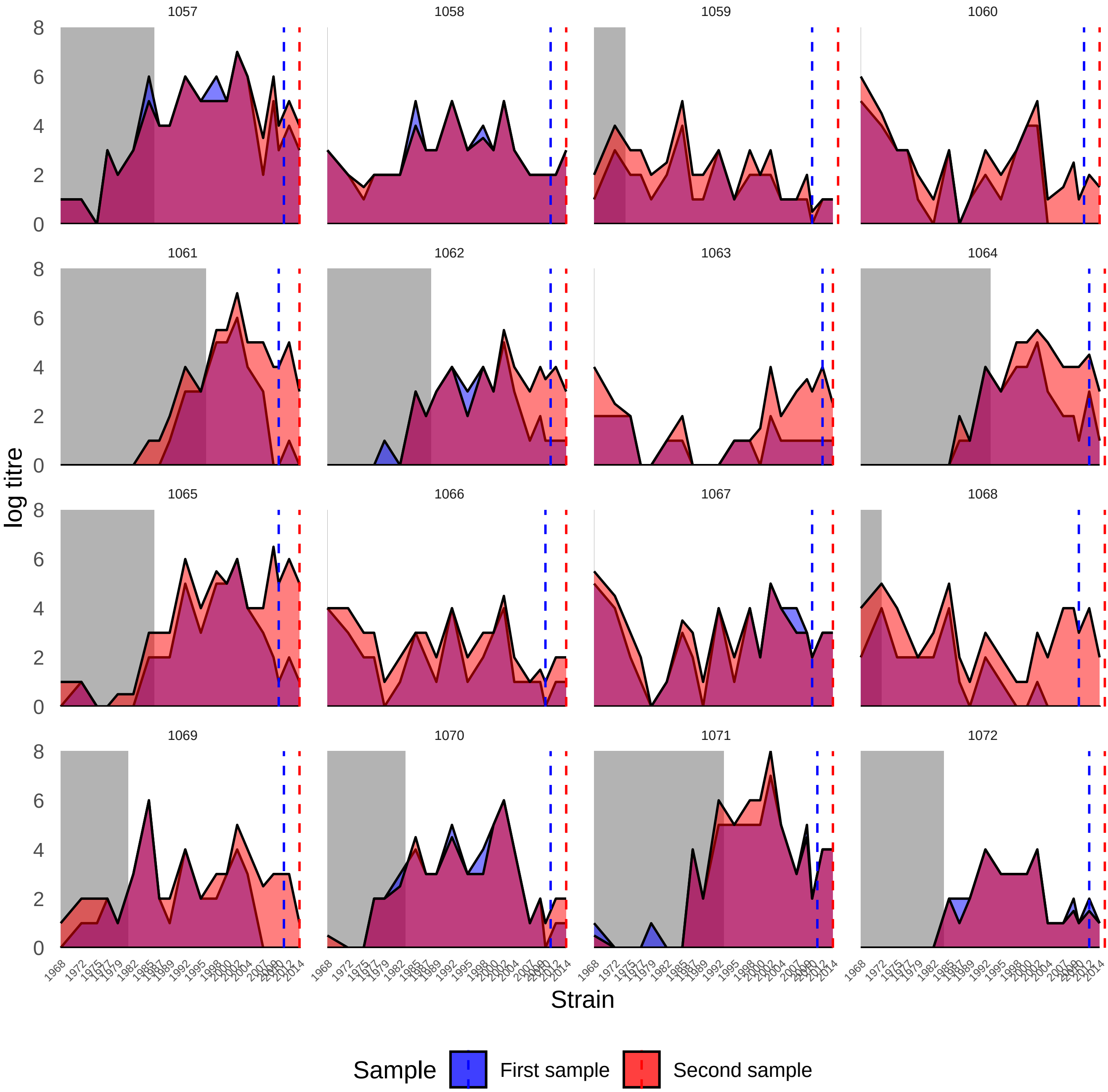
1057
1058
1059
1060
1061
1062
1063
1064
1065
1066
1067
1068
1069
1070
1071
1072
log titre
Strain
Sample
First sample
Second sample

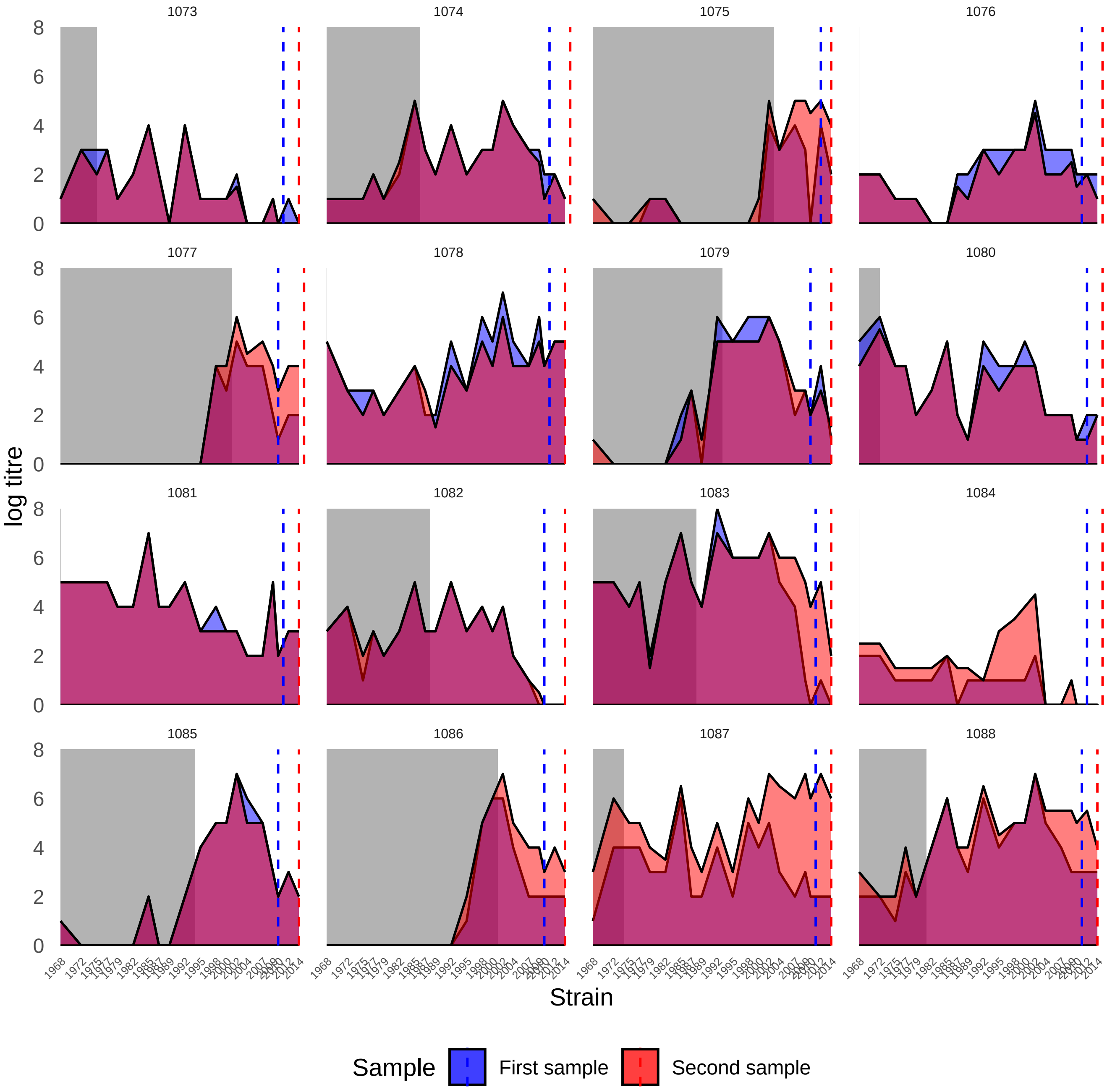

1073
1074
1075
1076
1077
1078
1079
1080
1081
1082
1083
1084
1085
1086
1087
1088
log titre
Strain
Sample
First sample
Second sample

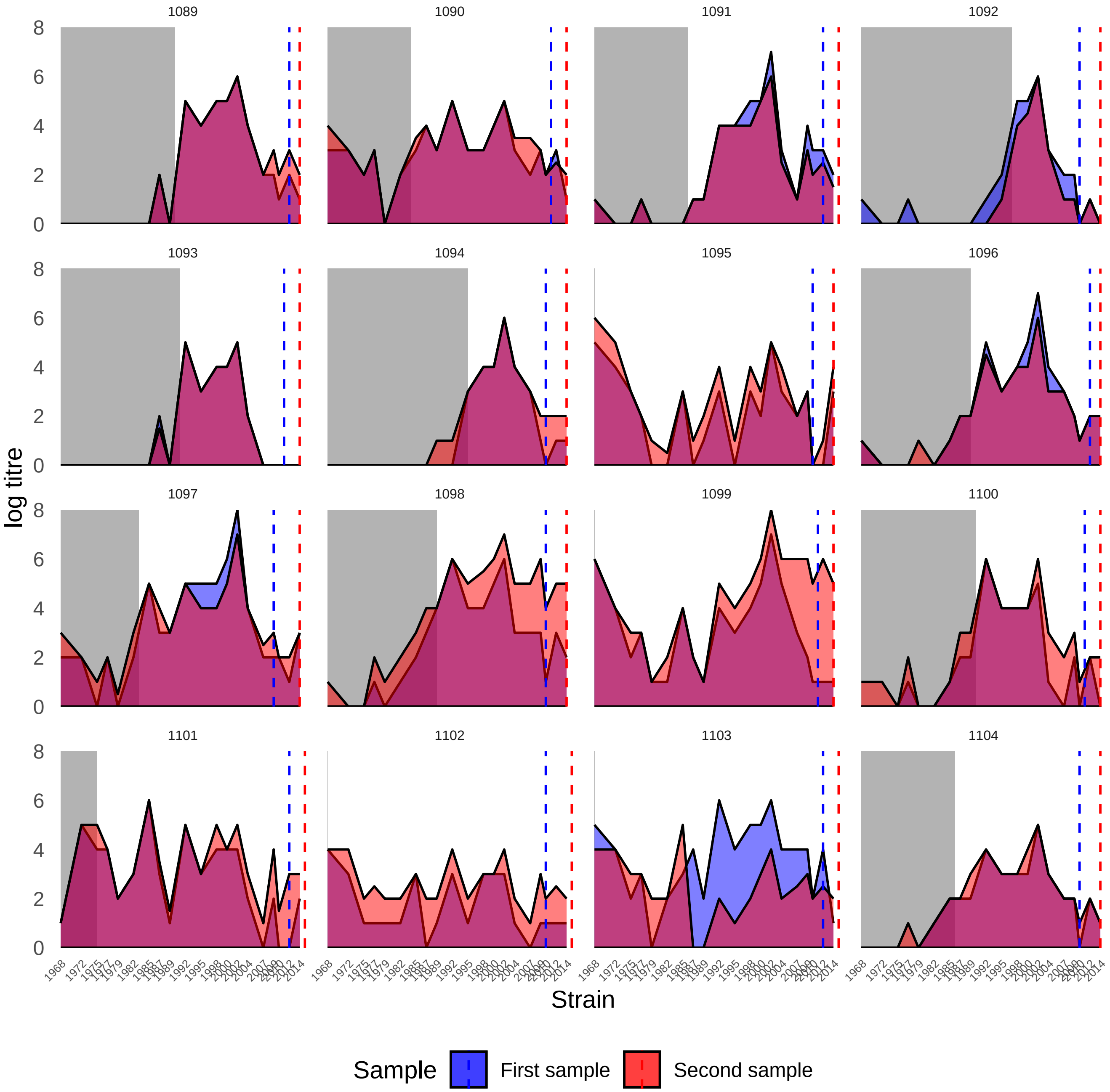

1089
1090
1091
1092
1093
1094
1095
1096
1097
1098
1099
1100
1101
1102
1103
1104
log titre
0
2
4
6
8
1968
1972
1975
1977
1979
1982
1985
1987
1989
1992
1995
1998
2000
2002
2004
2007
2009
2010
2012
2014
Strain
Sample
First sample
Second sample

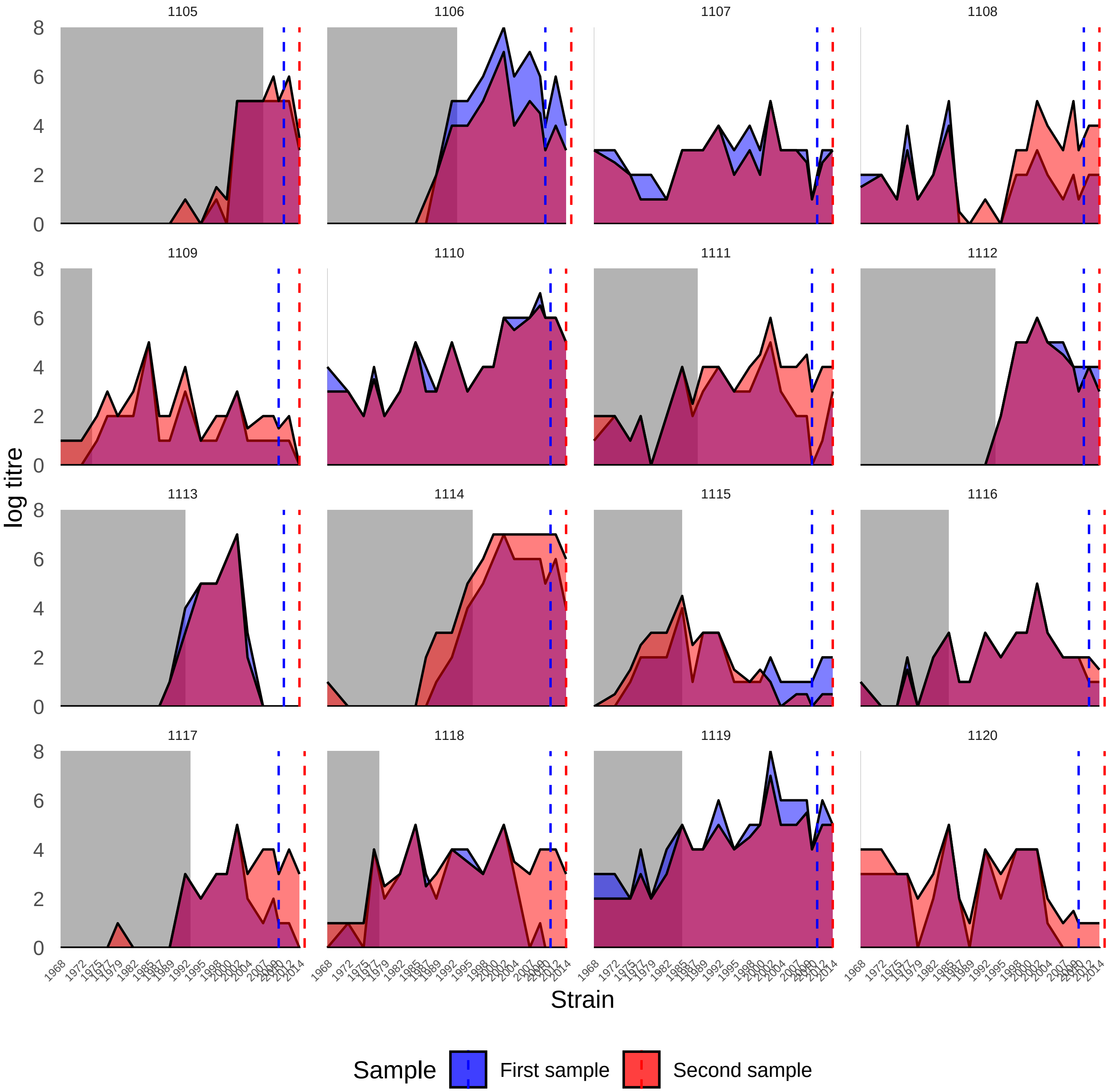
1105
1106
1107
1108
1109
1110
1111
1112
1113
1114
1115
1116
1117
1118
1119
1120
log titre
Strain
Sample
First sample
Second sample

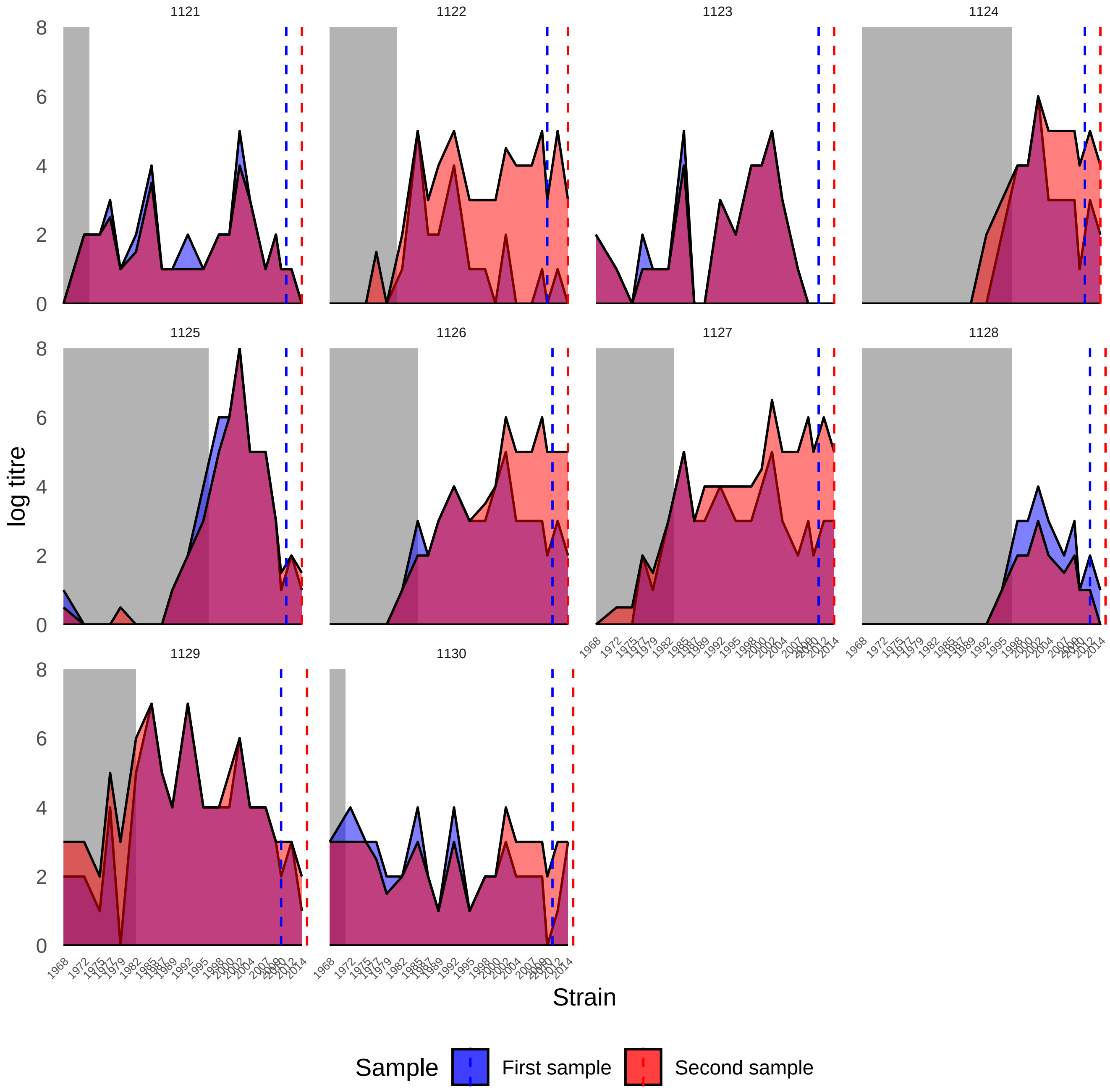
1121
1122
1123
1124
1125
1126
1127
1128
1129
1130
log titre
Strain
0
2
4
6
8
1968
1972
1975
1977
1979
1982
1985
1987
1989
1992
1995
1998
2000
2002
2004
2007
2009
2010
2012
2014
Sample
First sample
Second sample

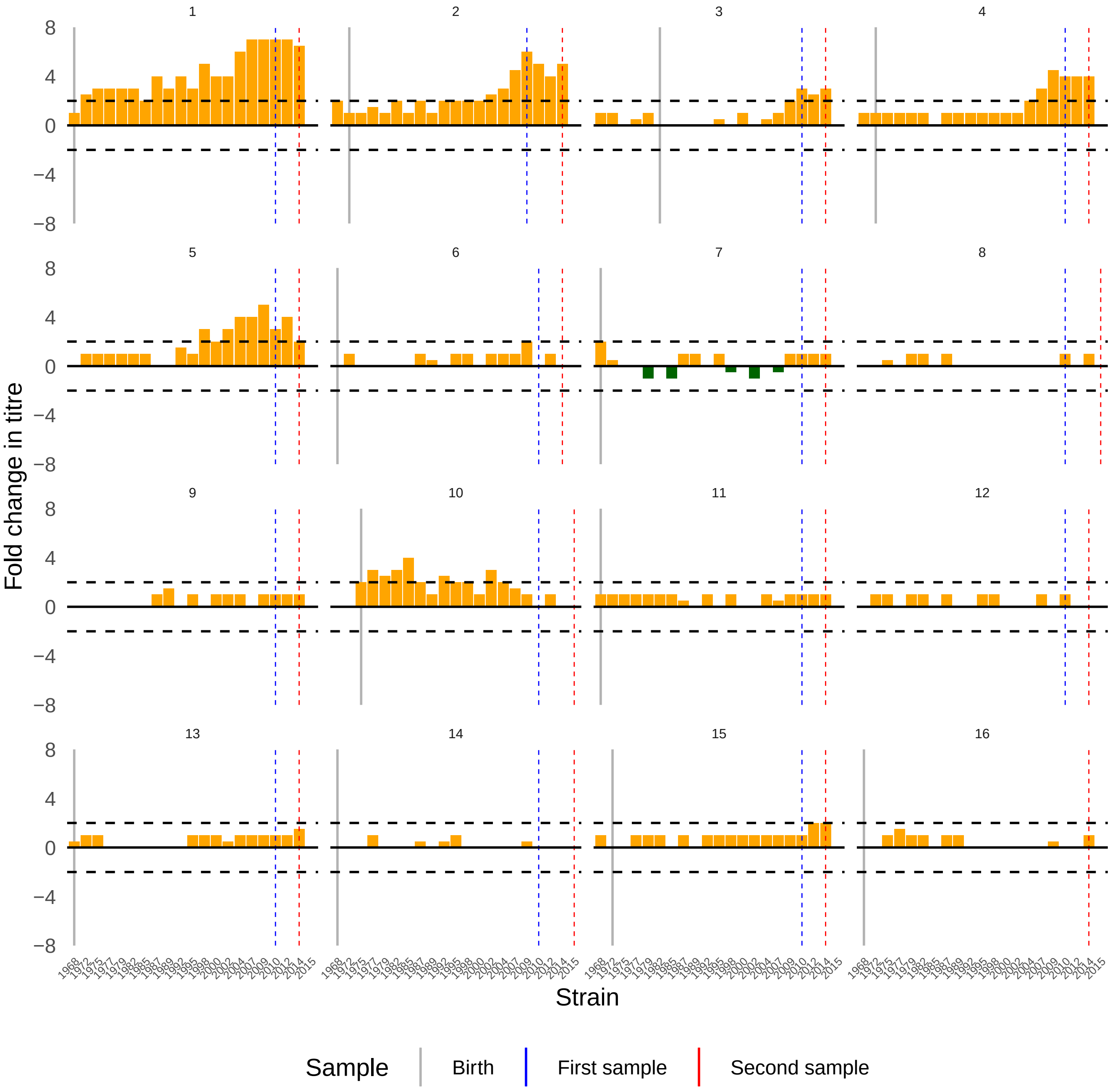

1
2
3
4
5
6
7
8
9
10
11
12
13
14
15
16
8
4
0
−4
−8
Fold change in titre
1968
1972
1975
1977
1979
1982
1985
1987
1989
1992
1995
1998
2000
2002
2004
2007
2009
2010
2012
2014
2015
Strain
Sample
Birth
First sample
Second sample

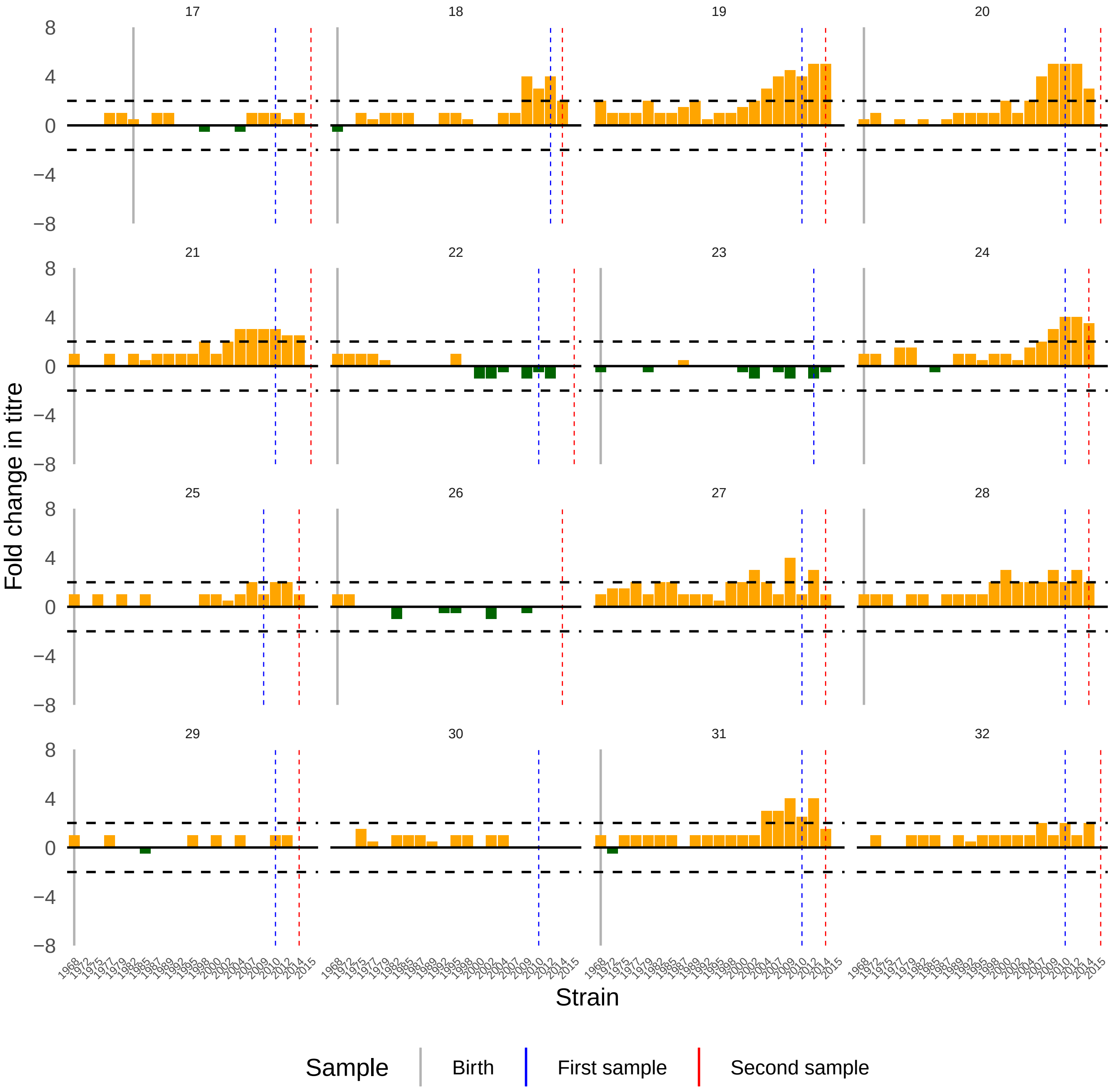
17
18
19
20
21
22
23
24
25
26
27
28
29
30
31
32
Fold change in titre
8
4
0
−4
−8
1968 1972 1975 1977 1979 1982 1985 1987 1989 1992 1995 1998 2000 2002 2004 2007 2009 2010 2012 2014 2015
Strain
Sample
Birth
First sample
Second sample

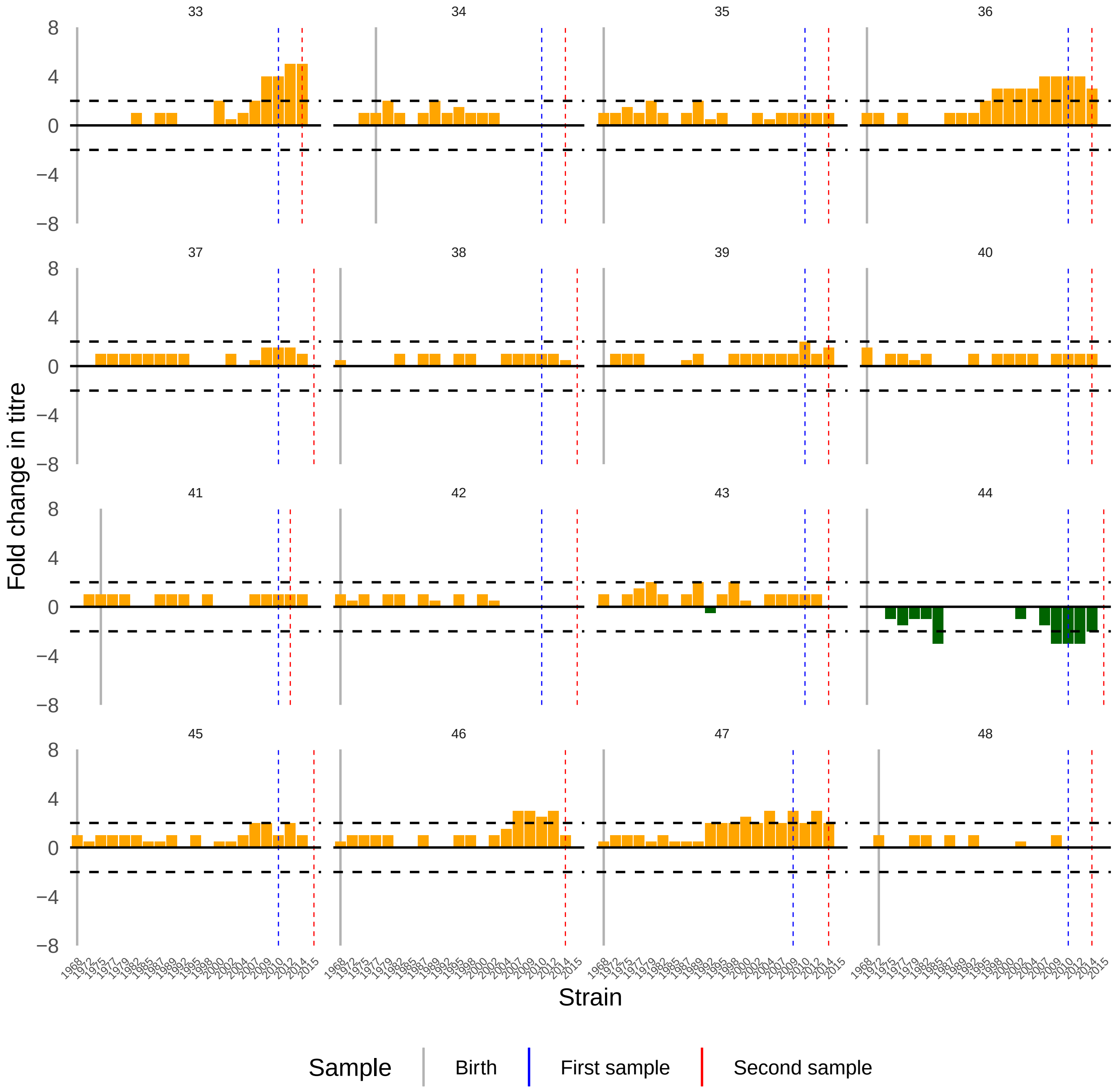

33
34
35
36
37
38
39
40
41
42
43
44
45
46
47
48
Fold change in titre
8
4
0
−4
−8
1968 1972 1975 1977 1979 1982 1985 1987 1989 1992 1995 1998 2000 2002 2004 2007 2009 2010 2012 2014 2015
Strain
Sample
Birth
First sample
Second sample

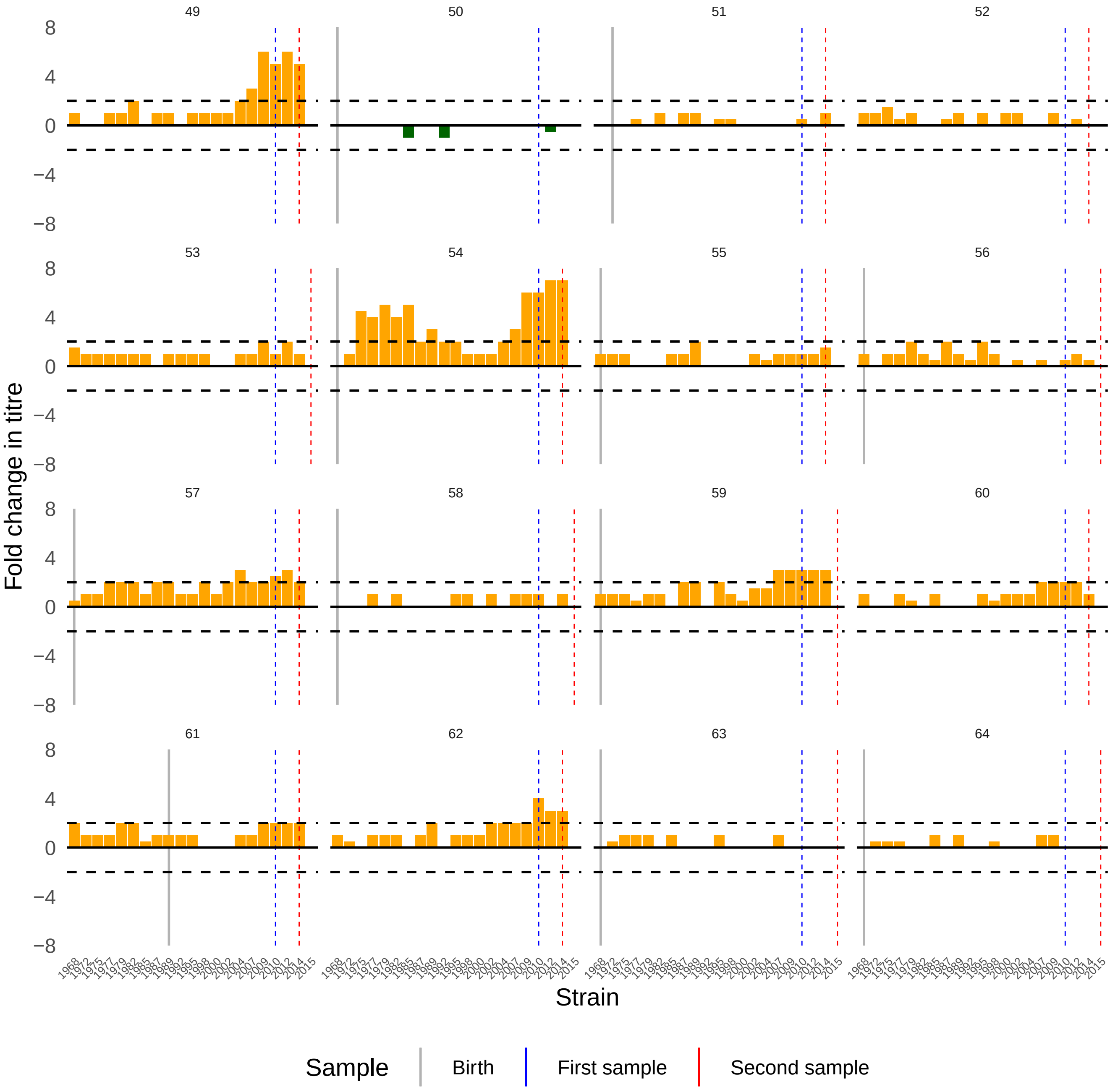

49
50
51
52
53
54
55
56
57
58
59
60
61
62
63
64
8
4
0
−4
−8
Fold change in titre
1968
1972
1975
1977
1979
1982
1985
1987
1989
1992
1995
1998
2000
2002
2004
2007
2009
2010
2012
2014
2015
Strain
Sample
Birth
First sample
Second sample

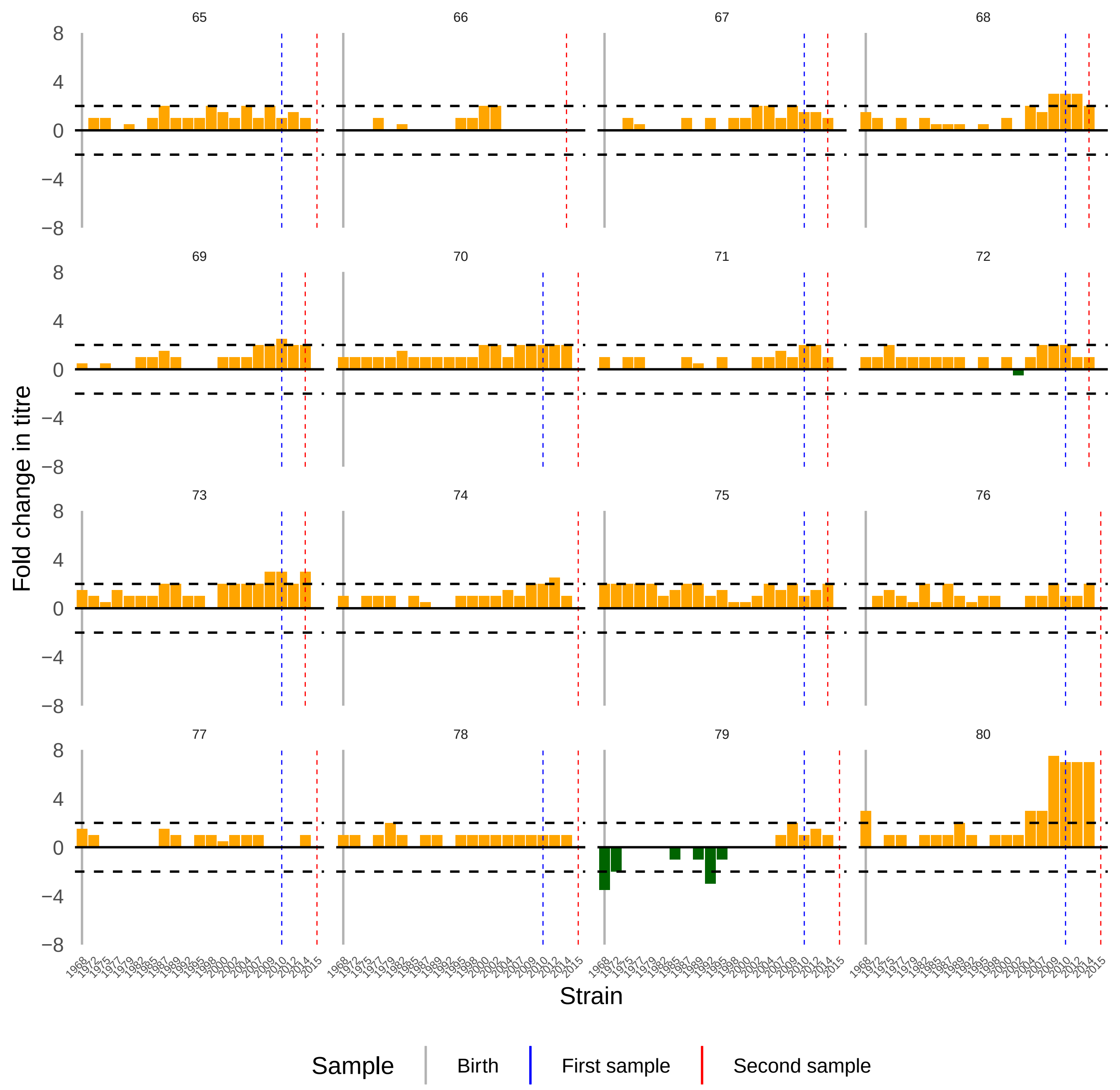

65
66
67
68
69
70
71
72
73
74
75
76
77
78
79
80
8
4
0
−4
−8
Fold change in titre
1968 1972 1975 1977 1979 1982 1985 1987 1989 1992 1995 1998 2000 2002 2004 2007 2009 2010 2012 2014 2015
Strain
Sample
Birth
First sample
Second sample

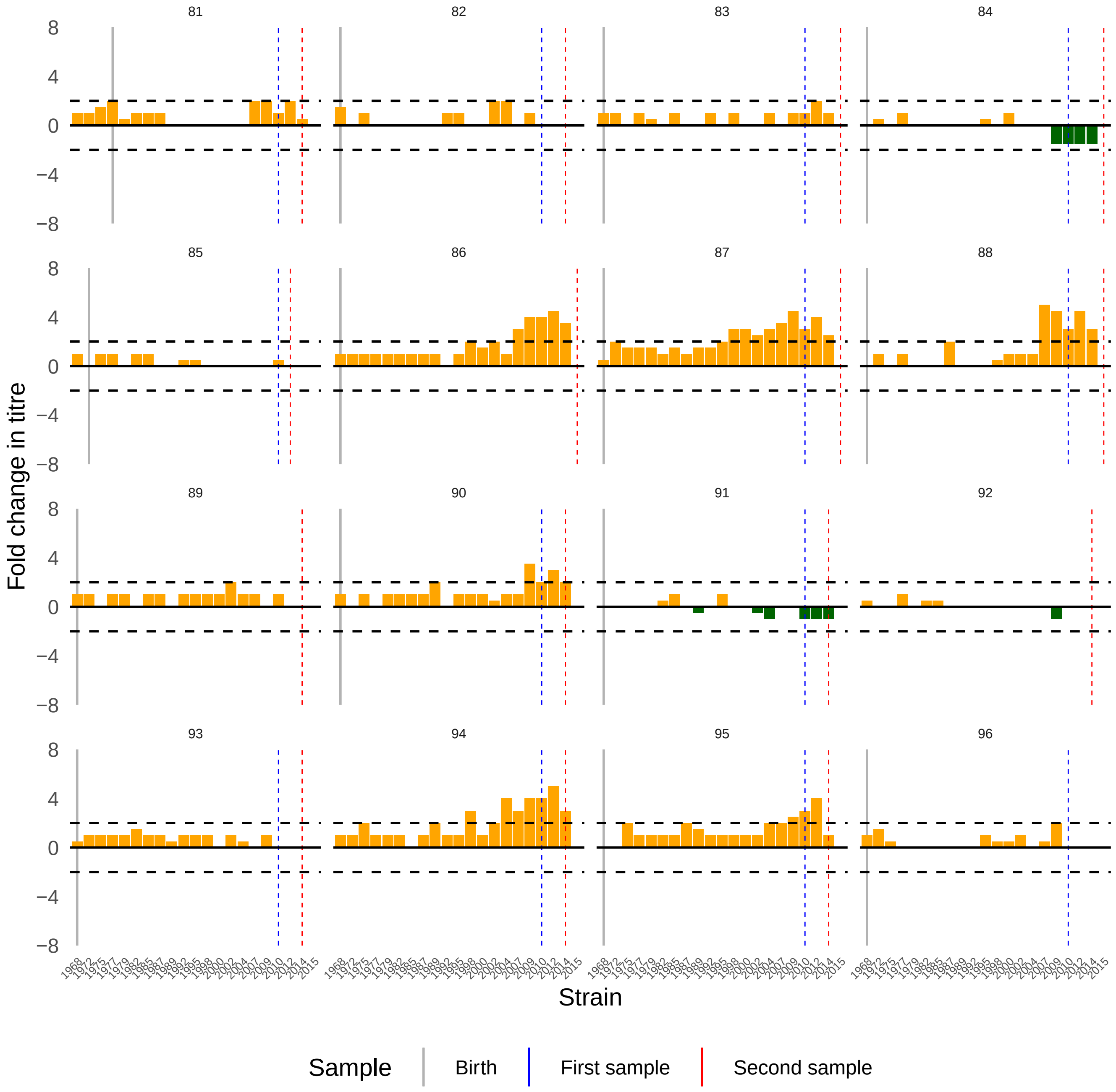

81
82
83
84
85
86
87
88
89
90
91
92
93
94
95
96
Fold change in titre
Strain
Sample
Birth
First sample
Second sample

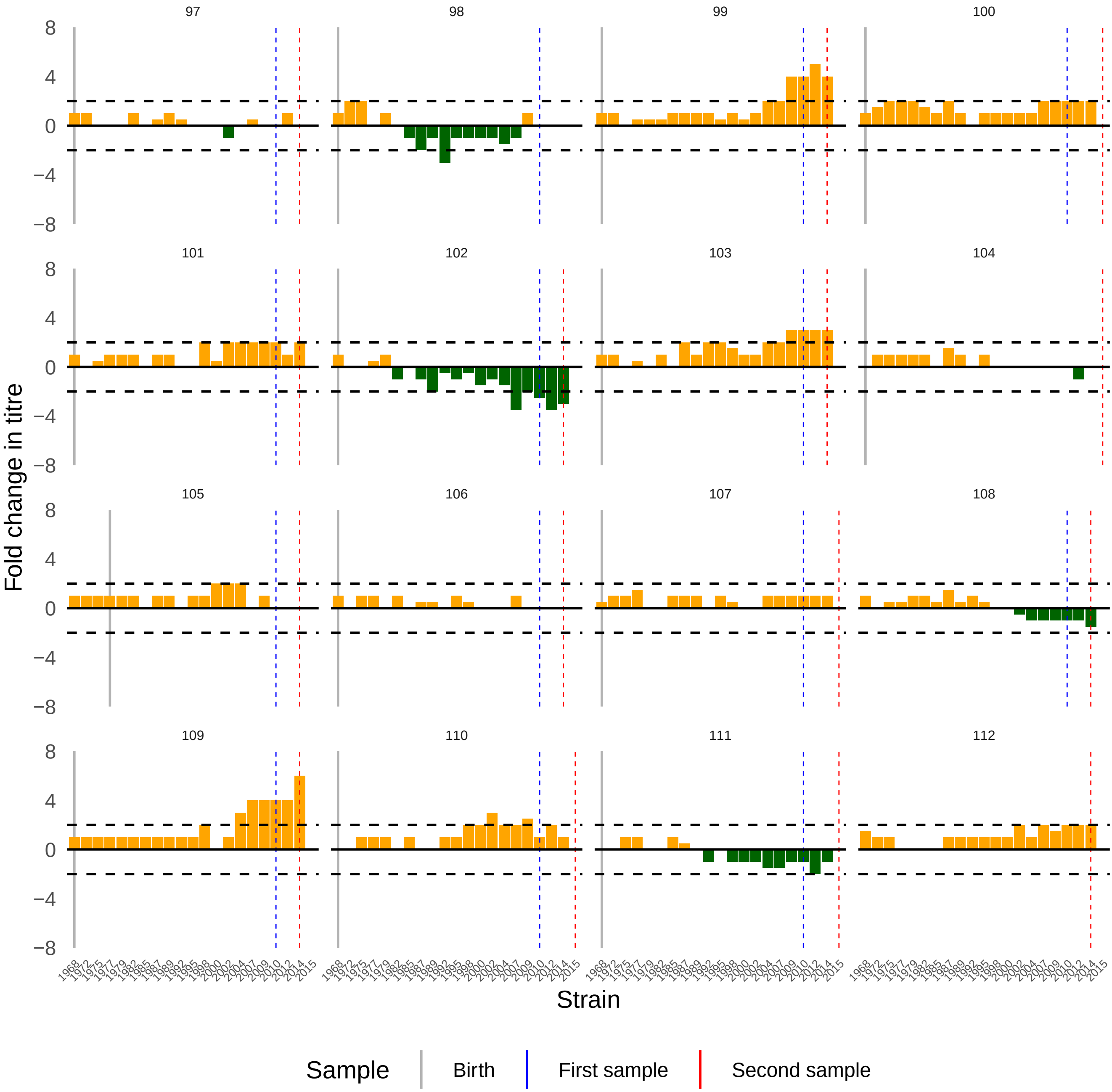

97
98
99
100
101
102
103
104
105
106
107
108
109
110
111
112
8
4
0
−4
−8
Fold change in titre
1968 1972 1975 1977 1979 1982 1985 1987 1989 1992 1995 1998 2000 2002 2004 2007 2009 2010 2012 2014 2015
Strain
Sample
Birth
First sample
Second sample

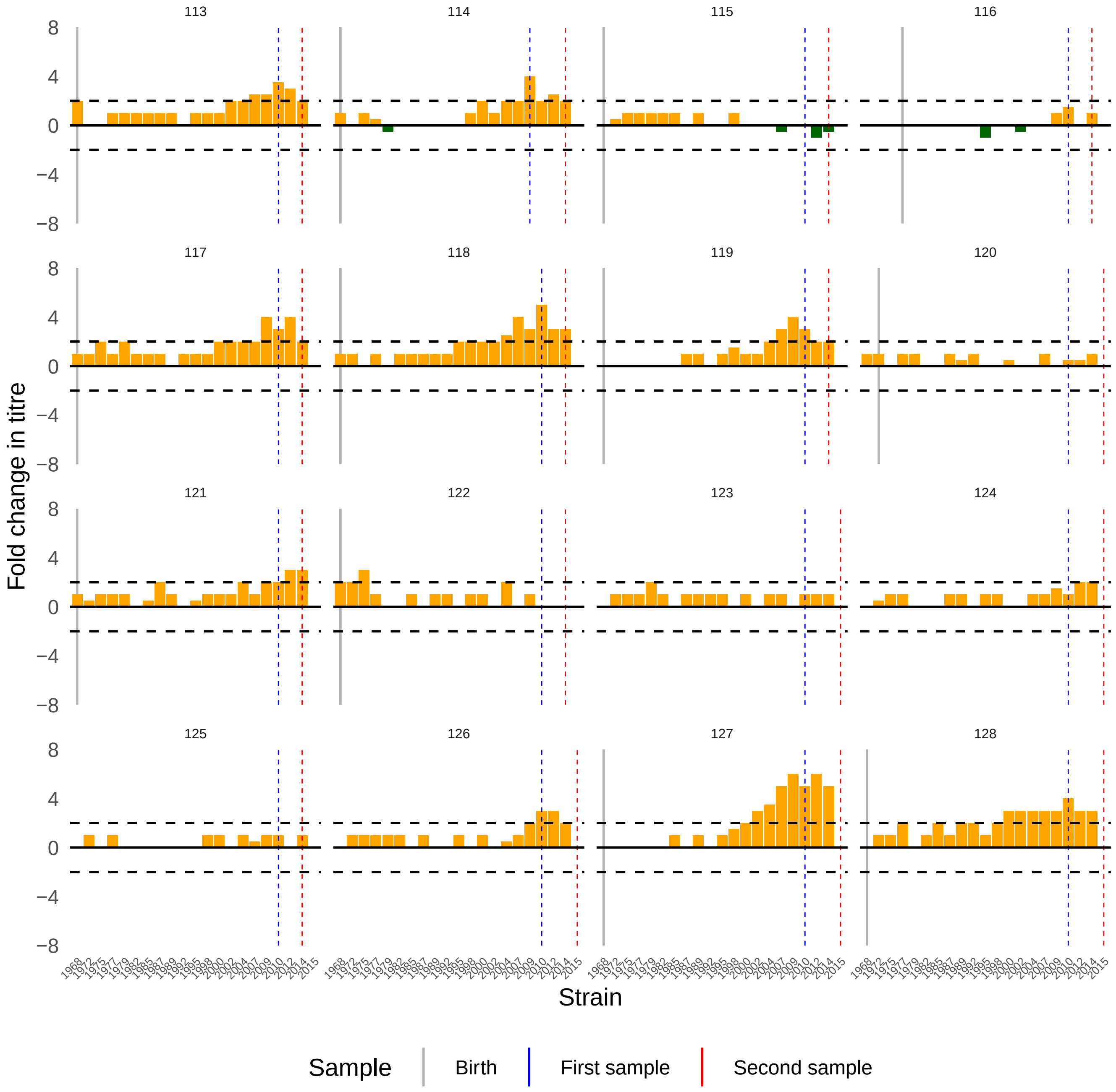

113
114
115
116
117
118
119
120
121
122
123
124
125
126
127
128
8
4
0
−4
−8
Fold change in titre
Strain
Sample
Birth
First sample
Second sample

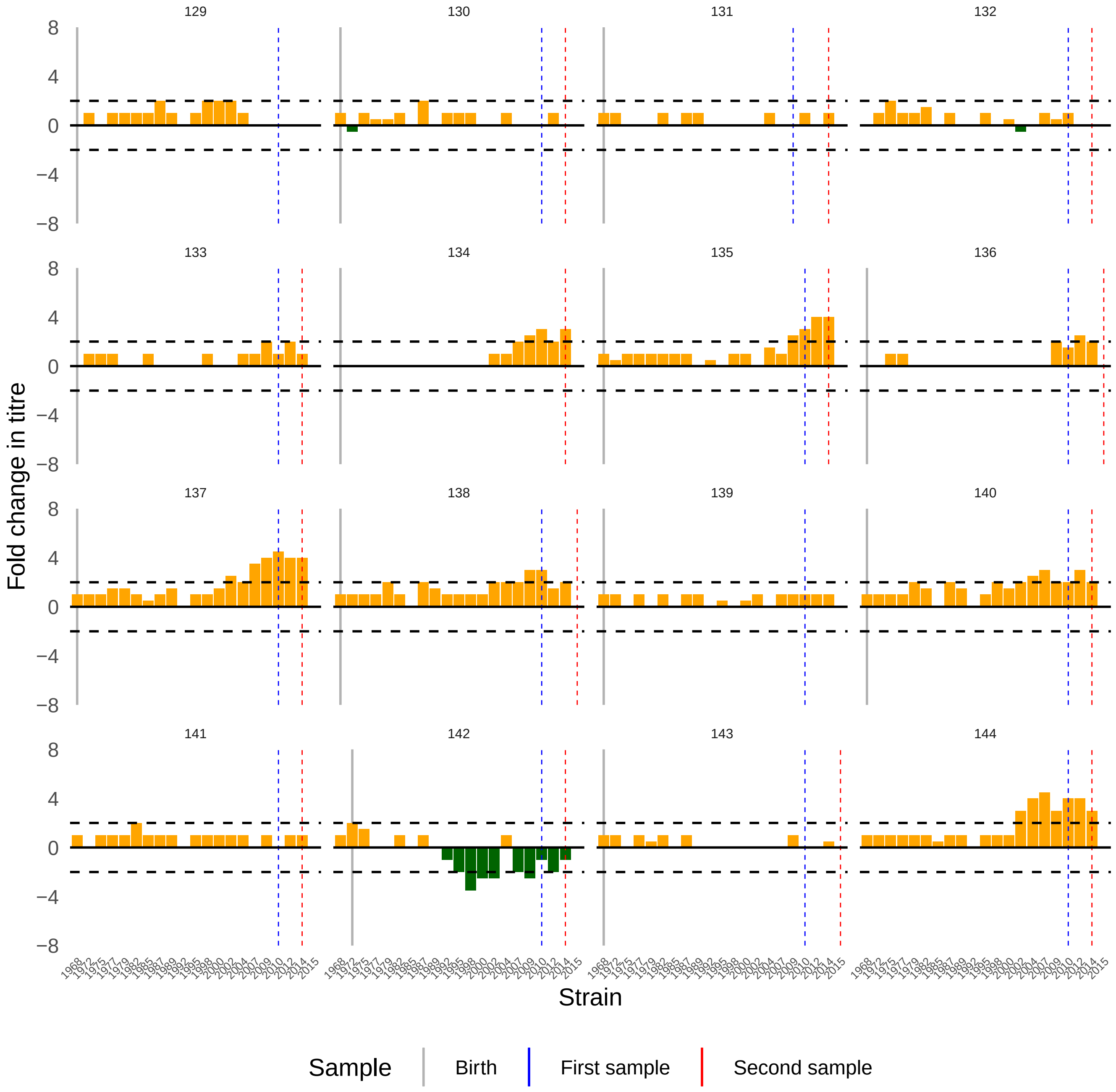

129
130
131
132
133
134
135
136
137
138
139
140
141
142
143
144
8
4
0
−4
−8
Fold change in titre
1968 1972 1975 1977 1979 1982 1985 1987 1989 1992 1995 1998 2000 2002 2004 2007 2009 2010 2012 2014 2015
Strain
Sample
Birth
First sample
Second sample

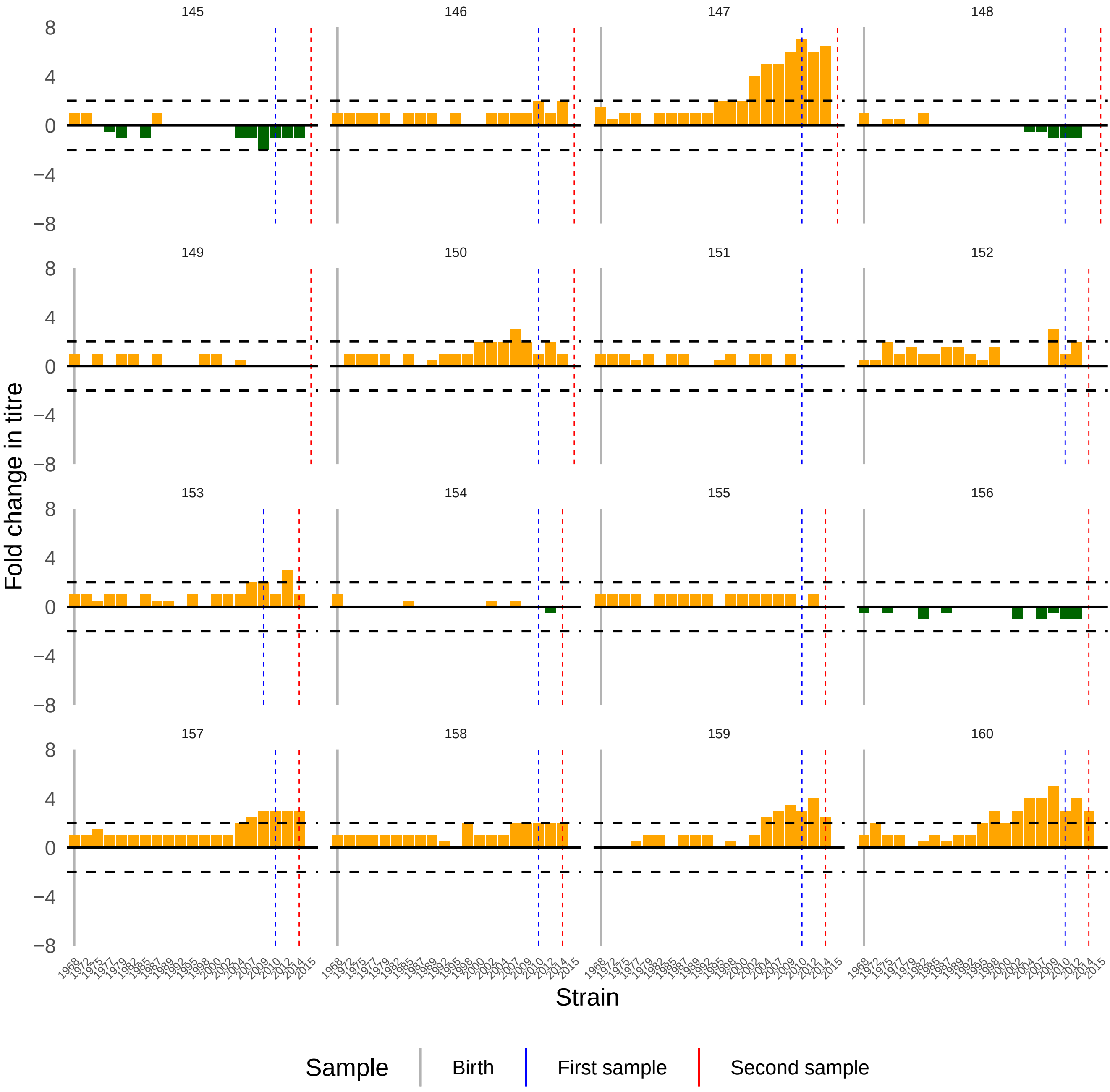

145
146
147
148
149
150
151
152
153
154
155
156
157
158
159
160
Fold change in titre
Strain
Sample
Birth
First sample
Second sample

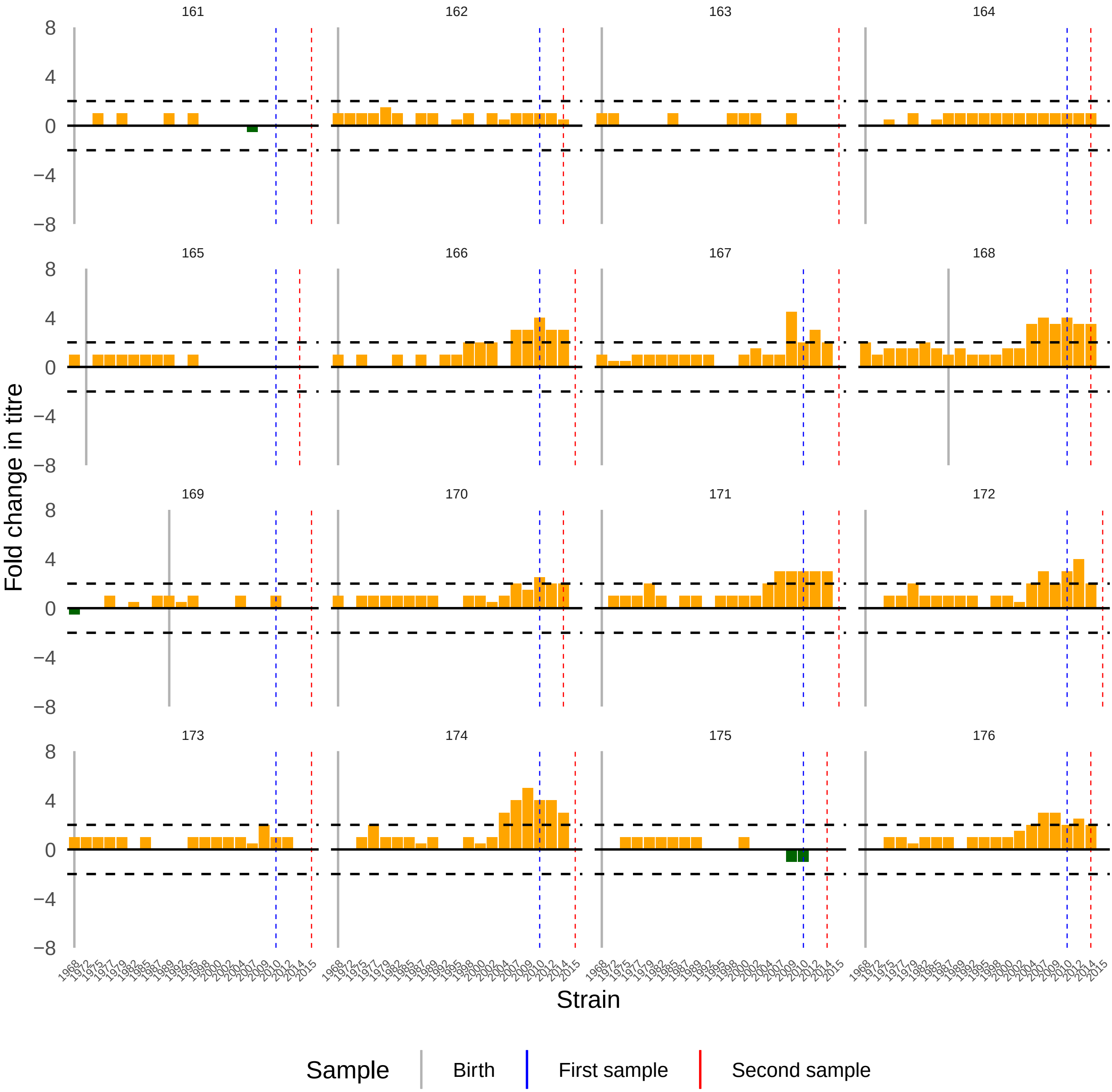
161
162
163
164
165
166
167
168
169
170
171
172
173
174
175
176
Fold change in titre
8
4
0
−4
−8
1968 1972 1975 1977 1979 1982 1985 1987 1989 1992 1995 1998 2000 2002 2004 2007 2009 2010 2012 2014 2015
Strain
Sample
Birth
First sample
Second sample

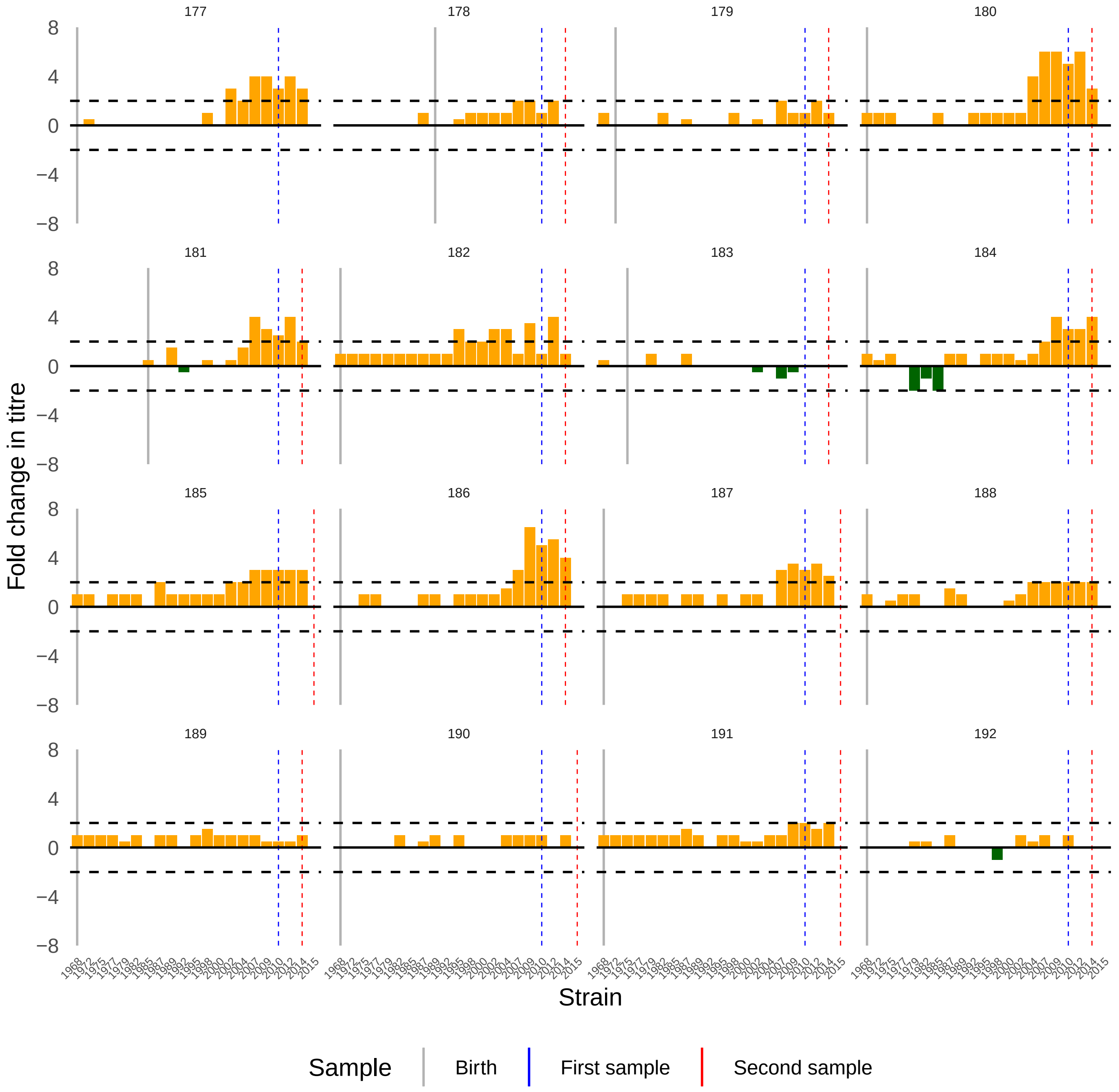

177
178
179
180
181
182
183
184
185
186
187
188
189
190
191
192
Fold change in titre
Strain
Sample
Birth
First sample
Second sample

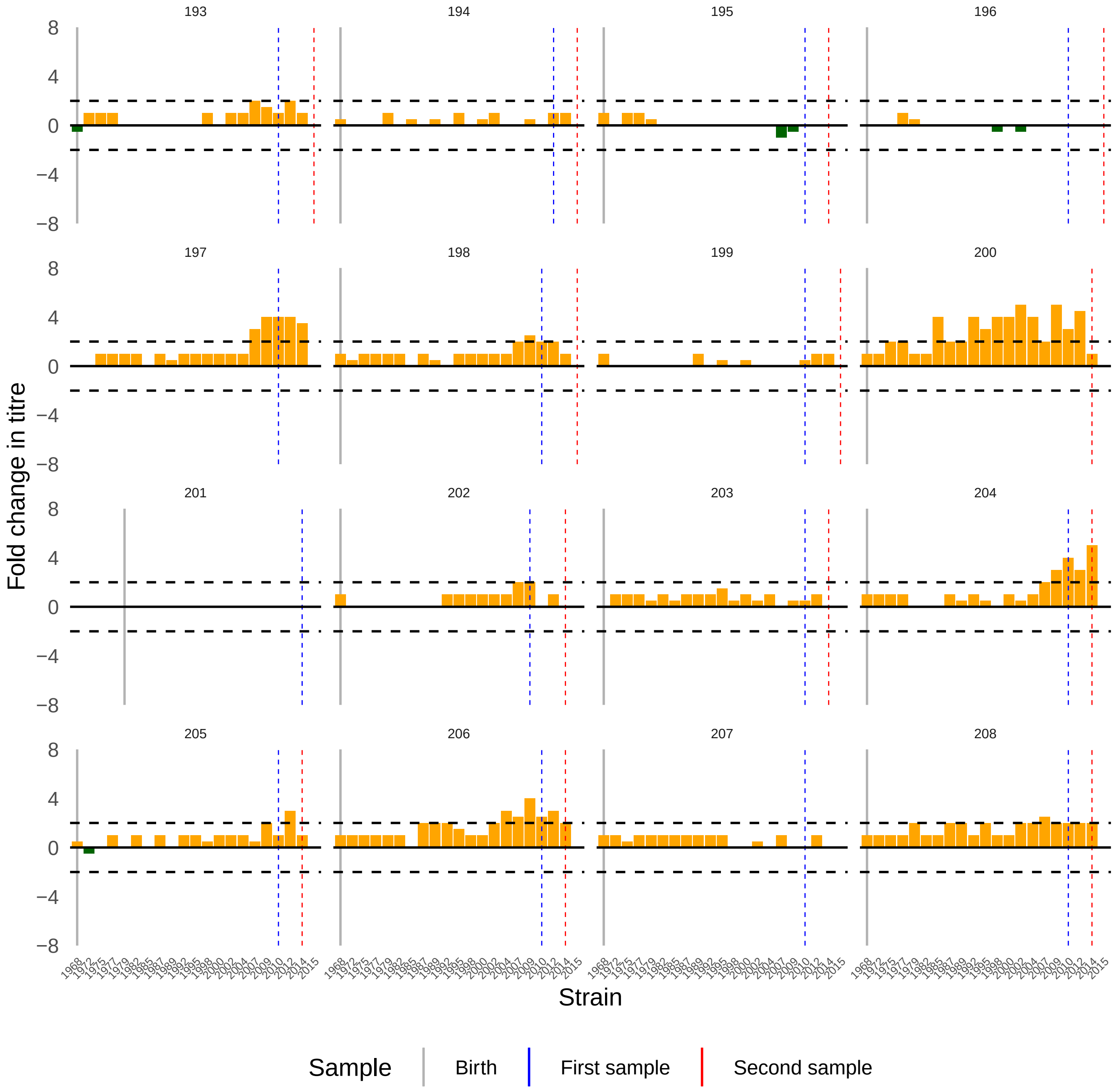
193
194
195
196
197
198
199
200
201
202
203
204
205
206
207
208
Fold change in titre
Strain
1968
1972
1975
1977
1979
1982
1985
1987
1989
1992
1995
1998
2000
2002
2004
2007
2009
2010
2012
2014
2015
Sample
Birth
First sample
Second sample

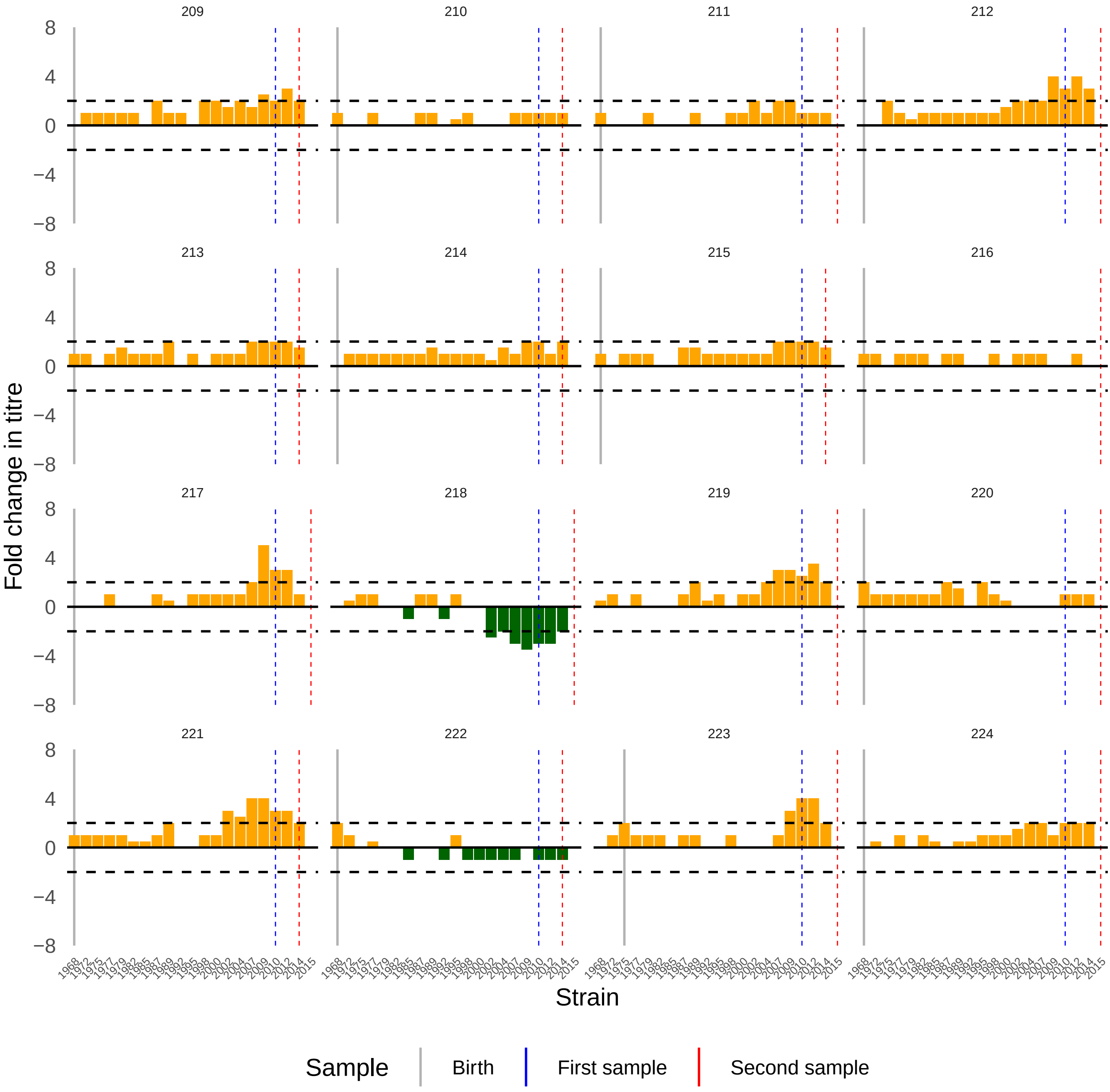
209
210
211
212
213
214
215
216
217
218
219
220
221
222
223
224
8
4
0
−4
−8
Fold change in titre
1968 1972 1975 1977 1979 1982 1985 1987 1989 1992 1995 1998 2000 2002 2004 2007 2009 2010 2012 2014 2015
Strain
Sample
Birth
First sample
Second sample

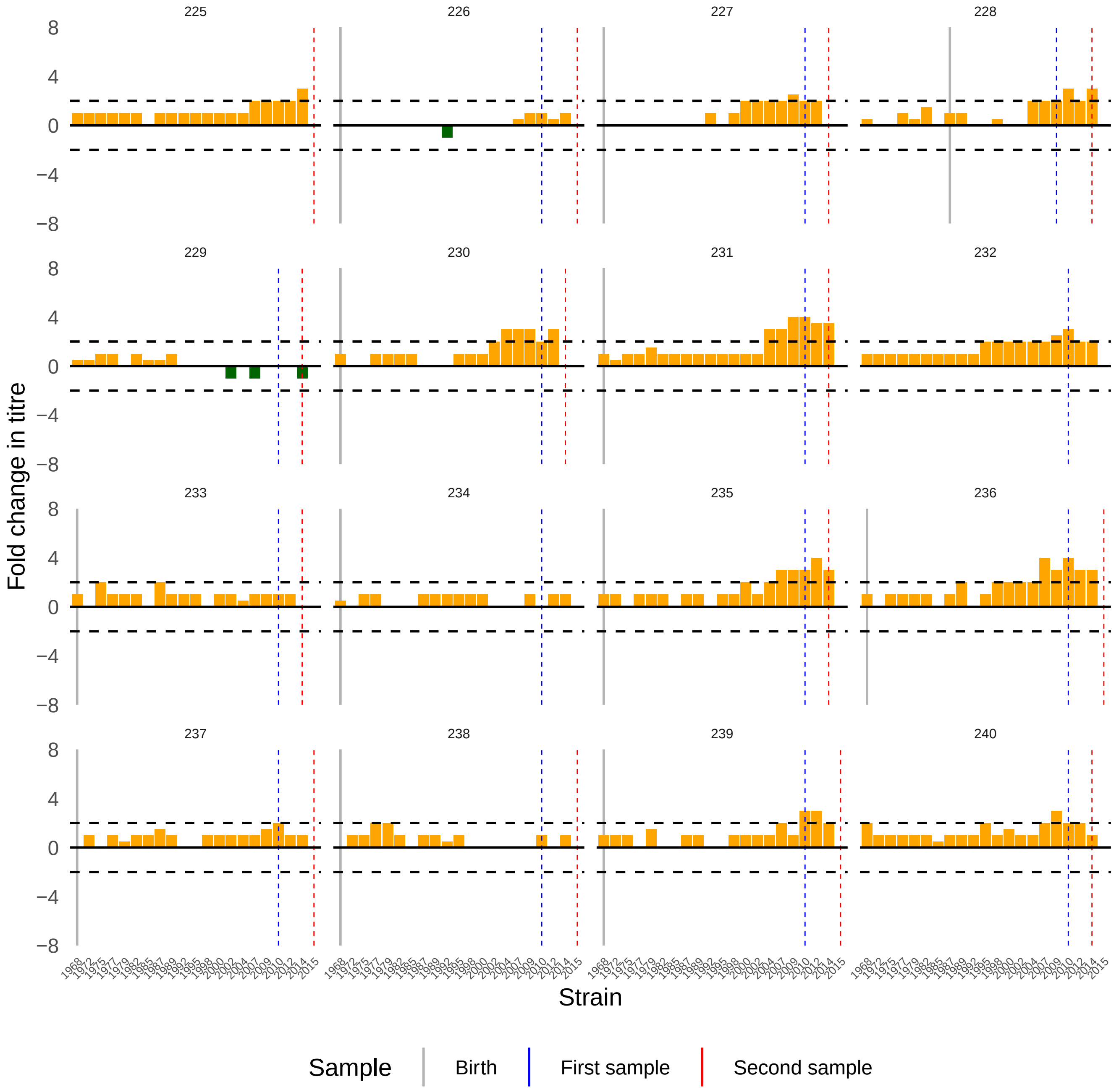

225
226
227
228
229
230
231
232
233
234
235
236
237
238
239
240
Fold change in titre
Strain
Sample
Birth
First sample
Second sample

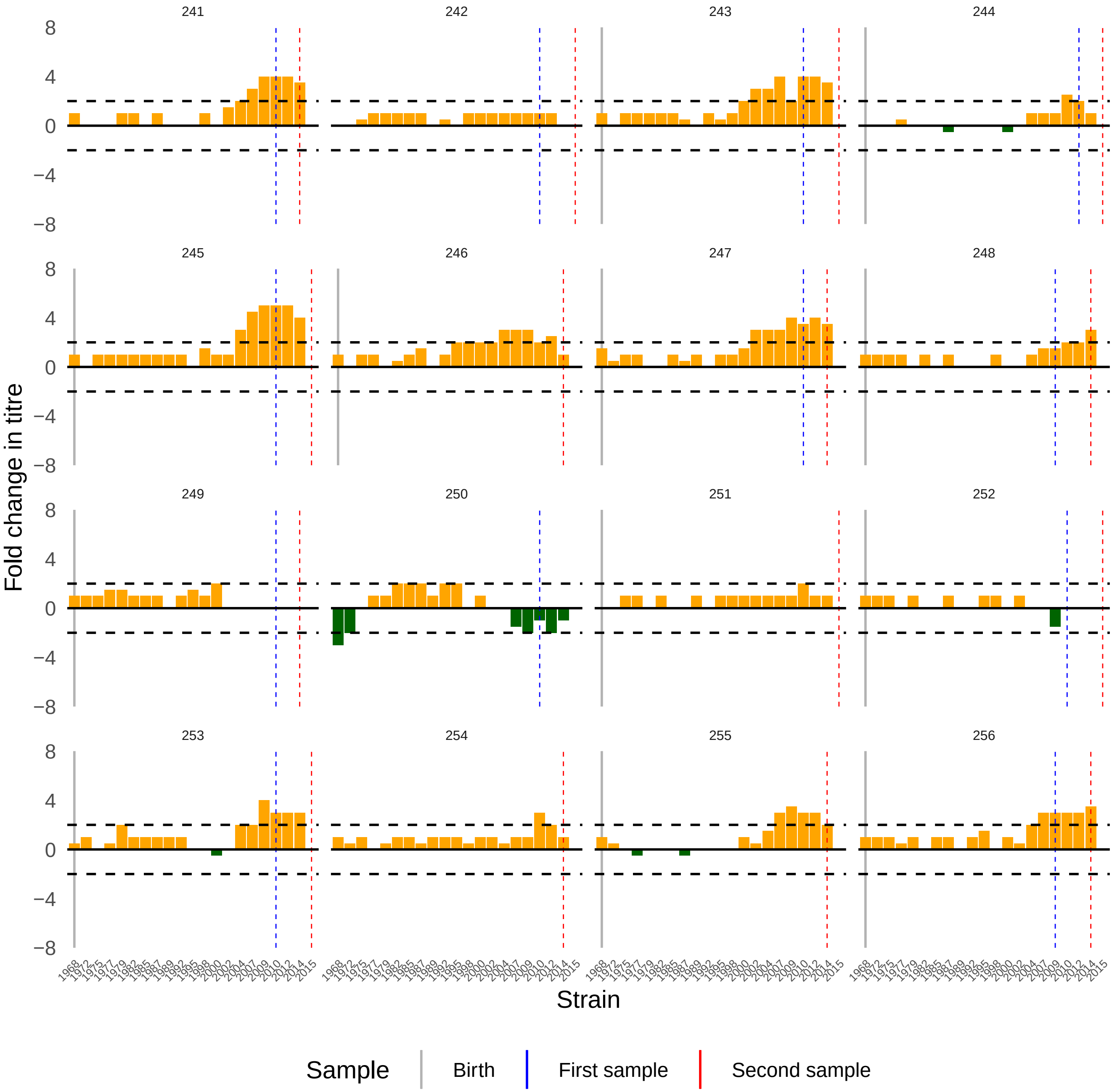

241
242
243
244
245
246
247
248
249
250
251
252
253
254
255
256
8
4
0
−4
−8
Fold change in titre
1968 1972 1975 1977 1979 1982 1985 1987 1989 1992 1995 1998 2000 2002 2004 2007 2009 2010 2012 2014 2015
Strain
Sample
Birth
First sample
Second sample

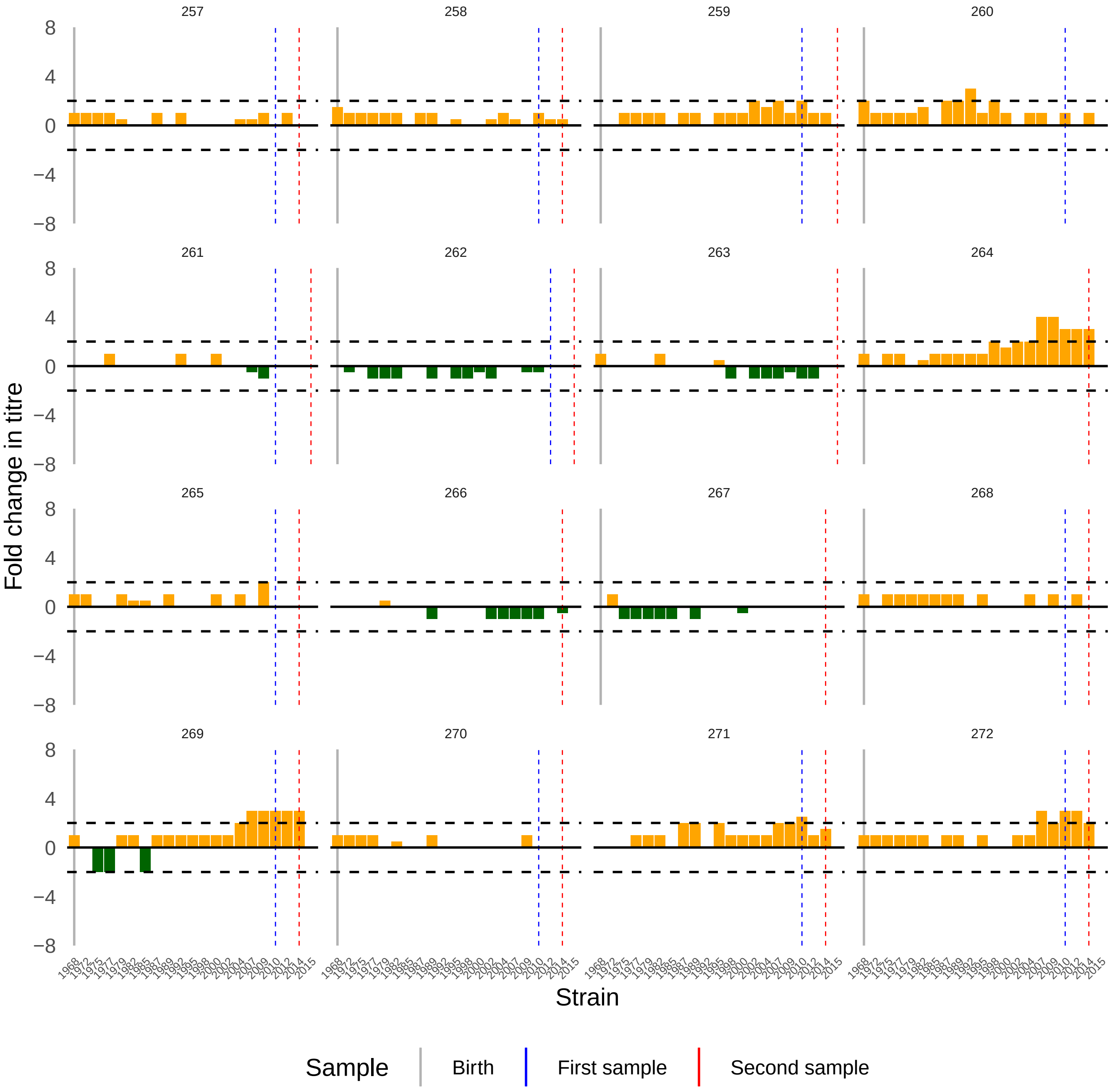

257
258
259
260
261
262
263
264
265
266
267
268
269
270
271
272
Fold change in titre
Strain
1968 1972 1975 1977 1979 1982 1985 1987 1989 1992 1995 1998 2000 2002 2004 2007 2009 2010 2012 2014 2015
Sample
Birth
First sample
Second sample

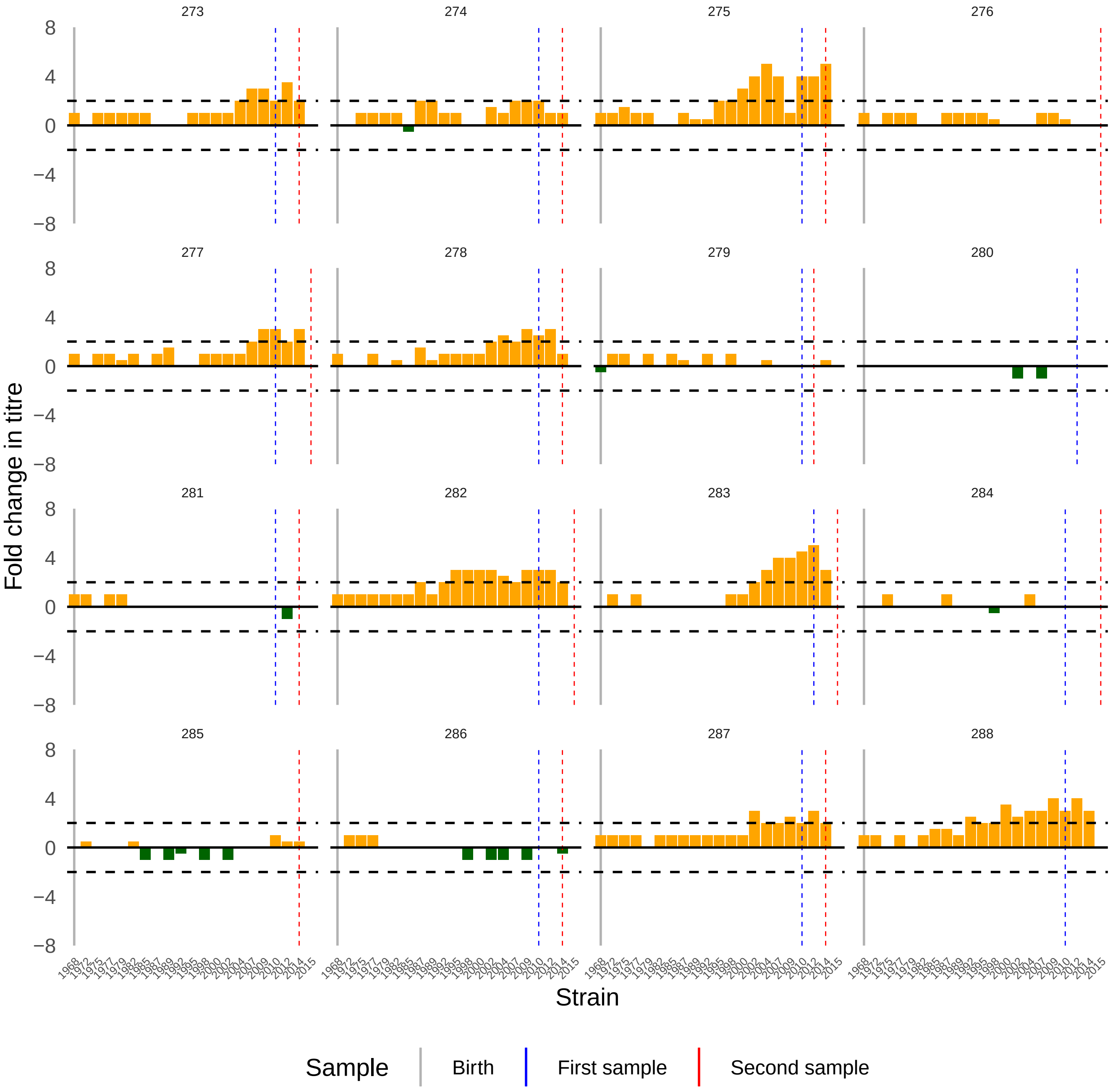

273
274
275
276
277
278
279
280
281
282
283
284
285
286
287
288
8
4
0
−4
−8
Fold change in titre
1968
1972
1975
1977
1979
1982
1985
1987
1989
1992
1995
1998
2000
2002
2004
2007
2009
2010
2012
2014
2015
Strain
Sample
Birth
First sample
Second sample

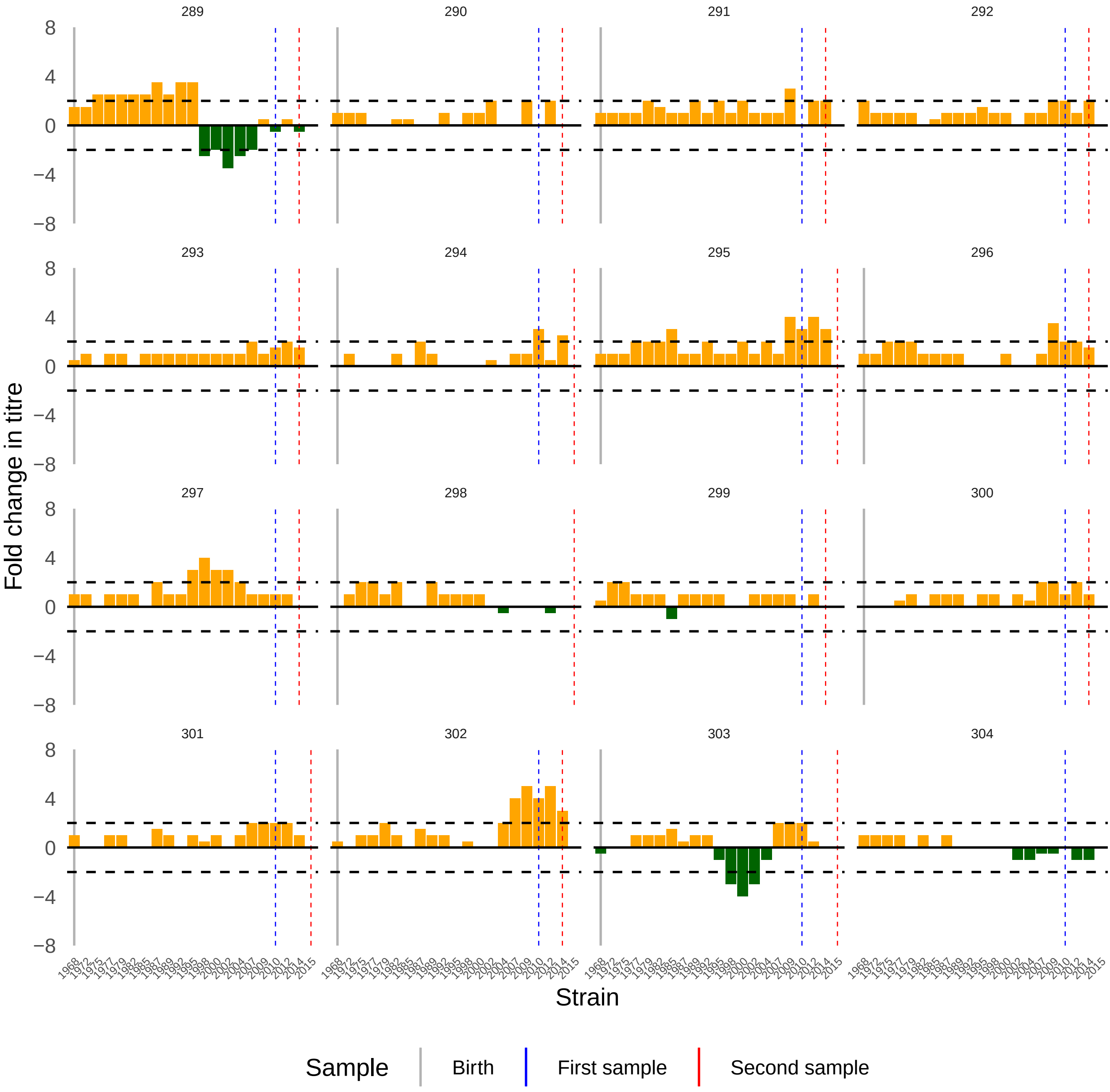
289
290
291
292
293
294
295
296
297
298
299
300
301
302
303
304
8
4
0
−4
−8
Fold change in titre
1968 1972 1975 1977 1979 1982 1985 1987 1989 1992 1995 1998 2000 2002 2004 2007 2009 2010 2012 2014 2015
Strain
Sample
Birth
First sample
Second sample

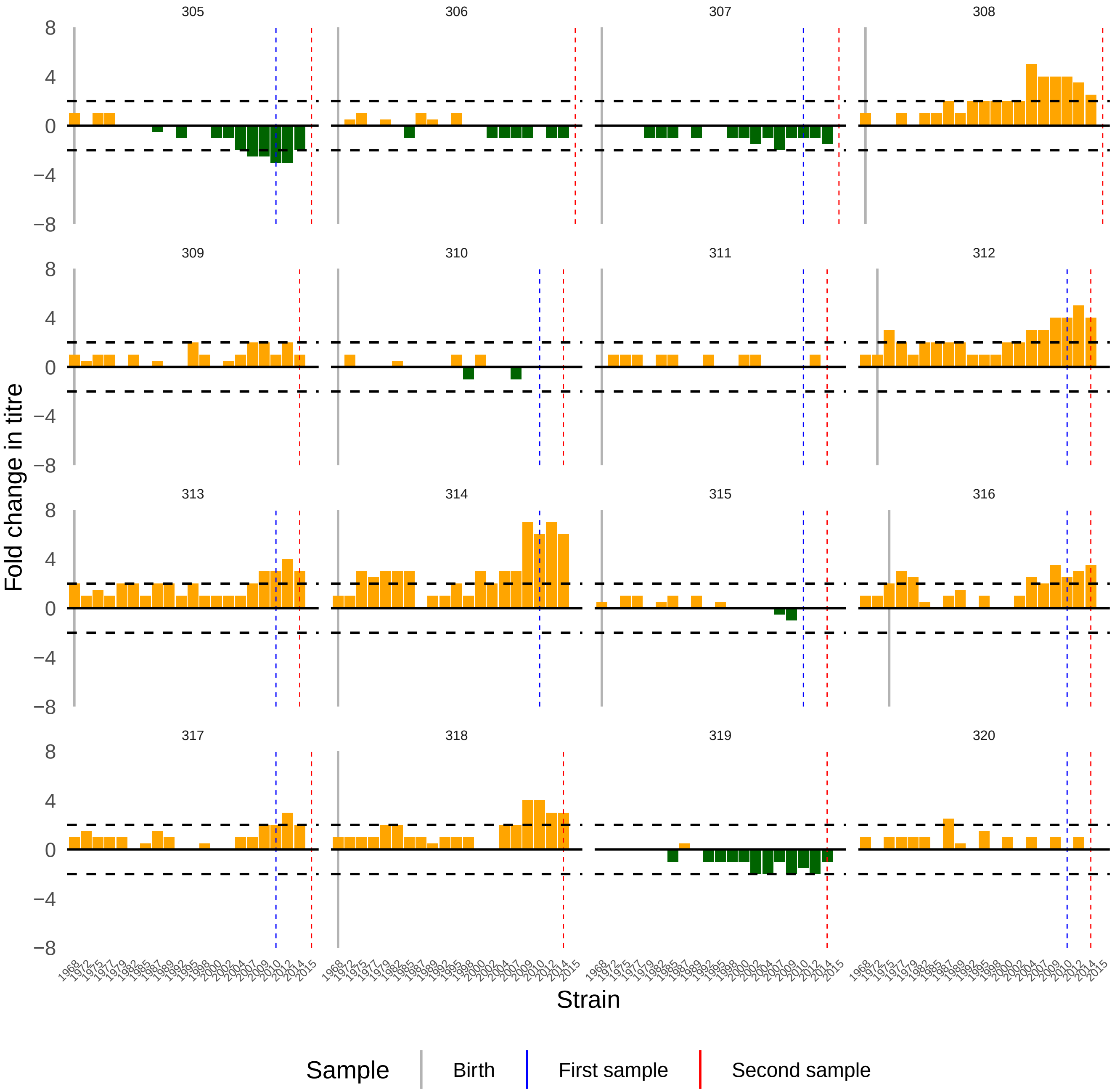
305
306
307
308
309
310
311
312
313
314
315
316
317
318
319
320
8
4
0
−4
−8
Fold change in titre
1968
1972
1975
1977
1979
1982
1985
1987
1989
1992
1995
1998
2000
2002
2004
2007
2009
2010
2012
2014
2015
Strain
Sample
Birth
First sample
Second sample

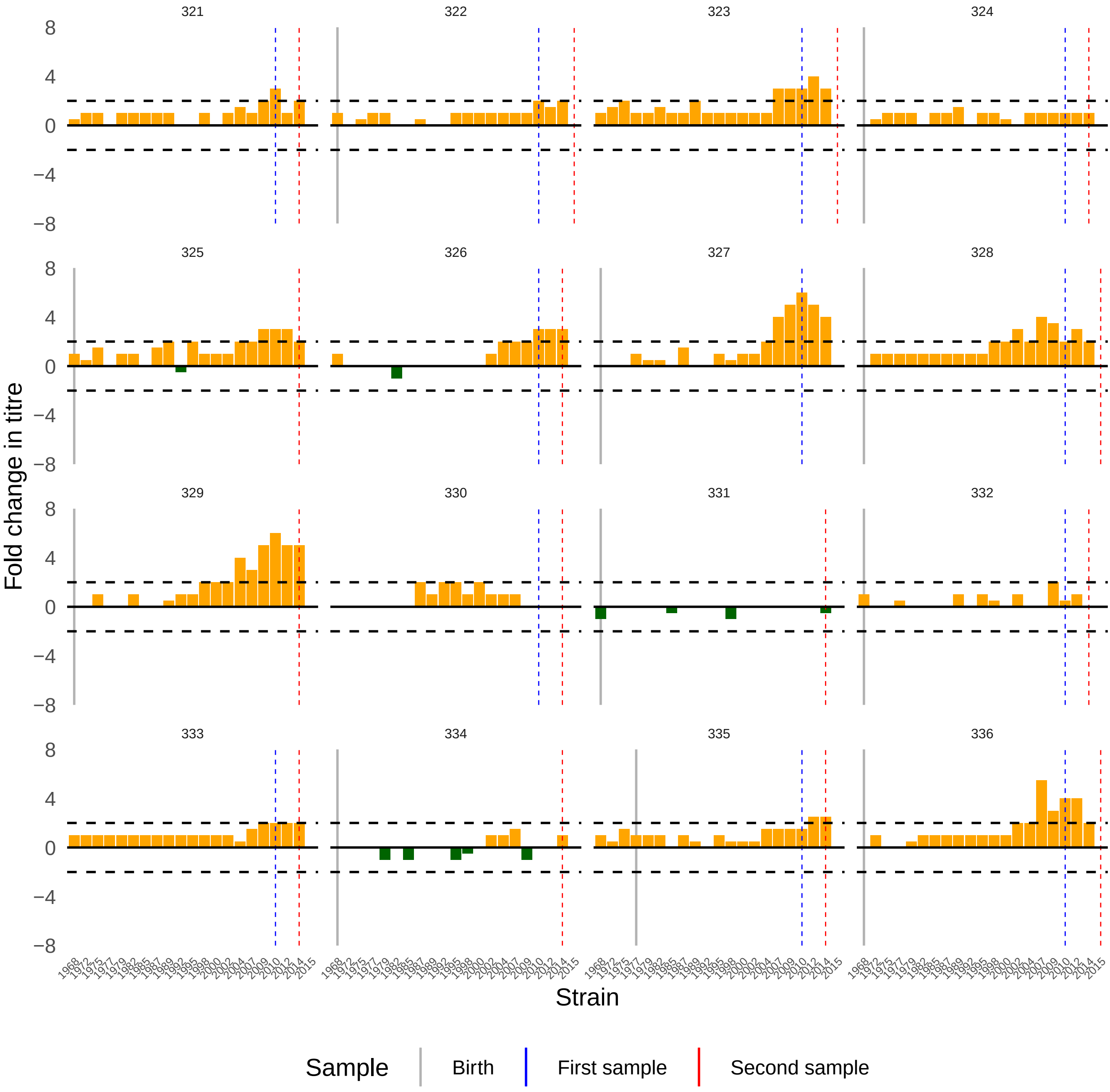
321
322
323
324
325
326
327
328
329
330
331
332
333
334
335
336
8
4
0
−4
−8
Fold change in titre
1968 1972 1975 1977 1979 1982 1985 1987 1989 1992 1995 1998 2000 2002 2004 2007 2009 2010 2012 2014 2015
Strain
Sample
Birth
First sample
Second sample

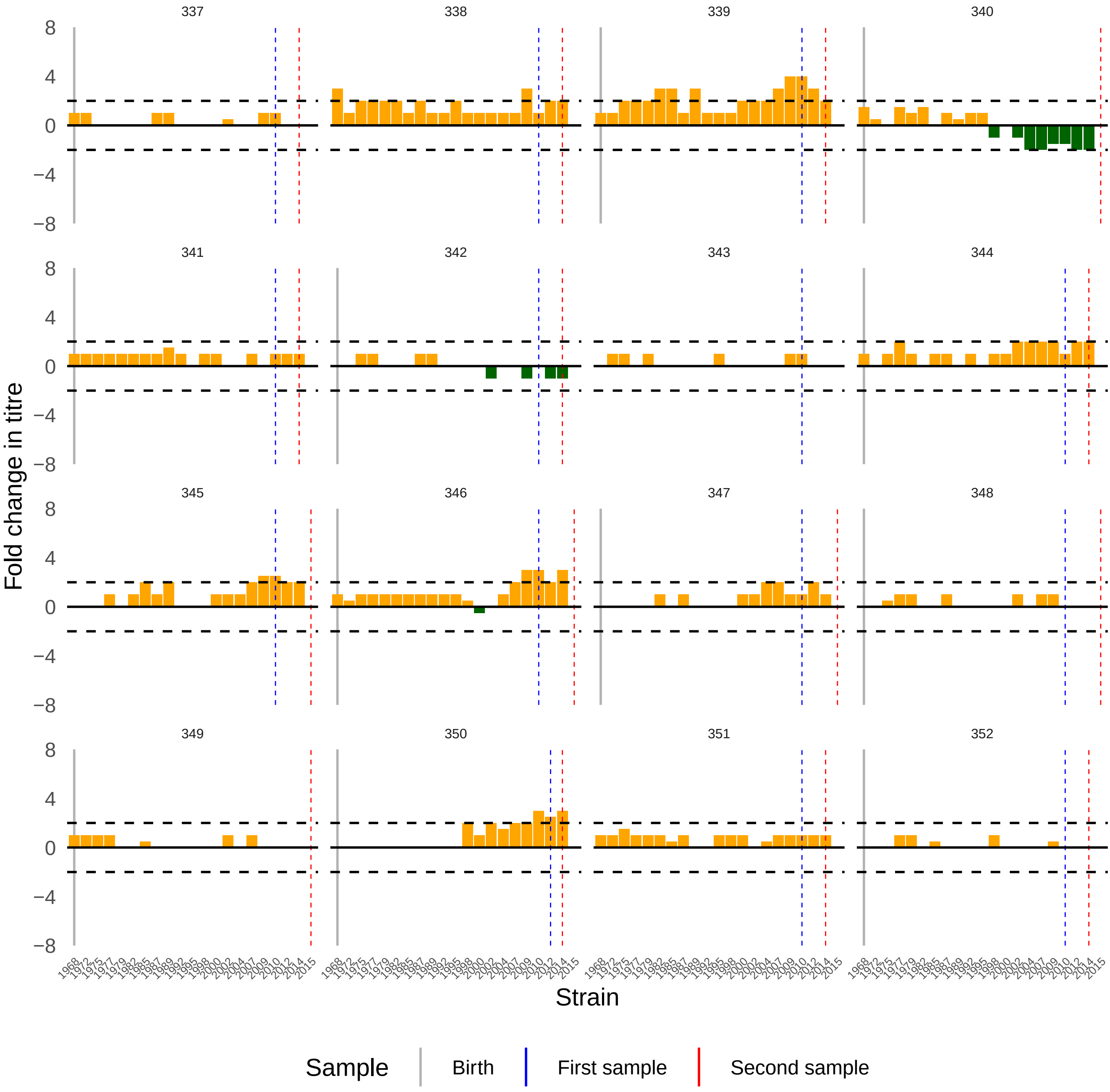
337
338
339
340
341
342
343
344
345
346
347
348
349
350
351
352
8
4
0
−4
−8
Fold change in titre
1968 1972 1975 1977 1979 1982 1985 1987 1989 1992 1995 1998 2000 2002 2004 2007 2009 2010 2012 2014 2015
Strain
Sample
Birth
First sample
Second sample

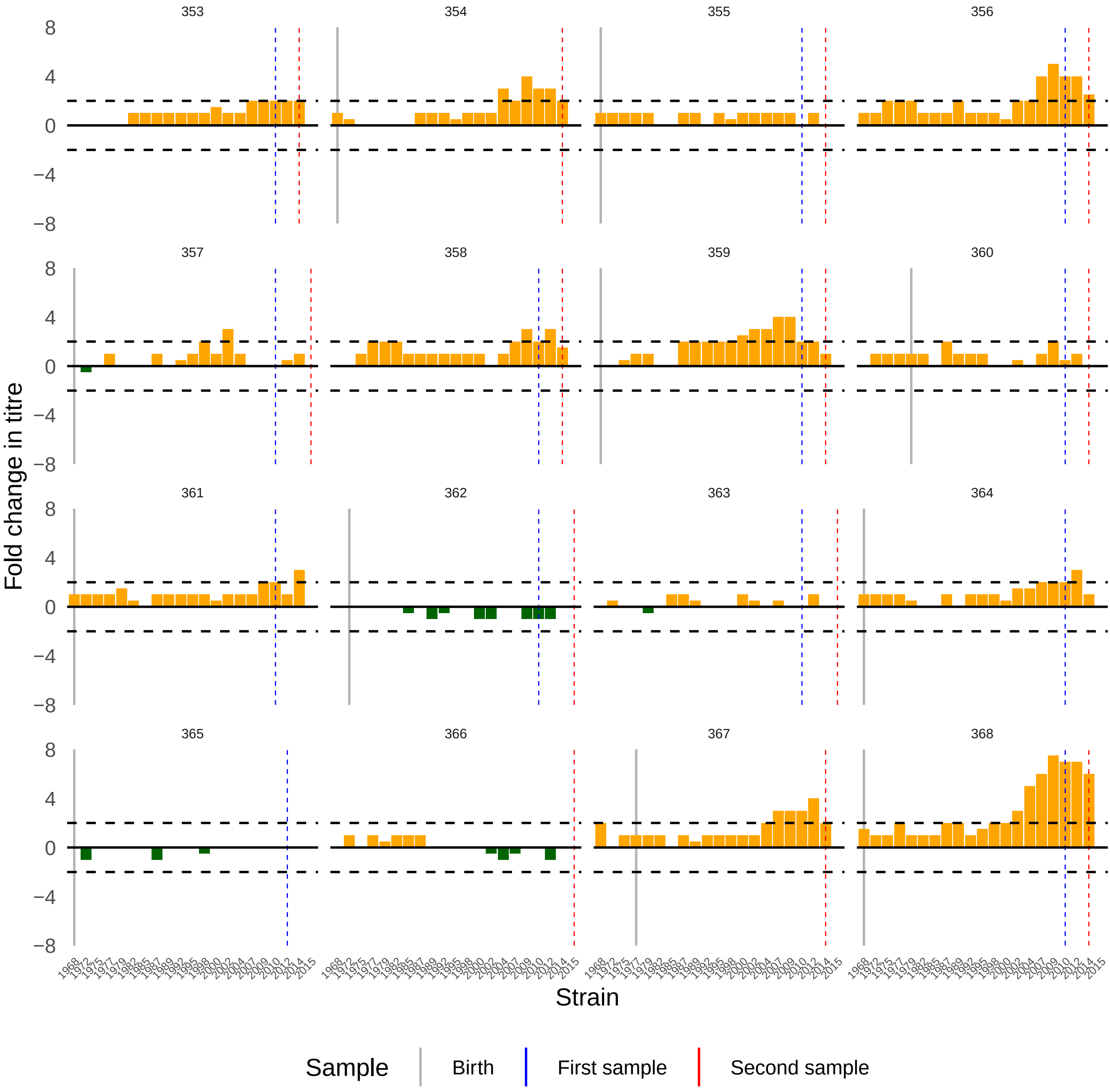
353
354
355
356
357
358
359
360
361
362
363
364
365
366
367
368
8
4
0
−4
−8
Fold change in titre
1968
1972
1975
1977
1979
1982
1985
1987
1989
1992
1995
1998
2000
2002
2004
2007
2009
2010
2012
2014
2015
Strain
Sample
Birth
First sample
Second sample

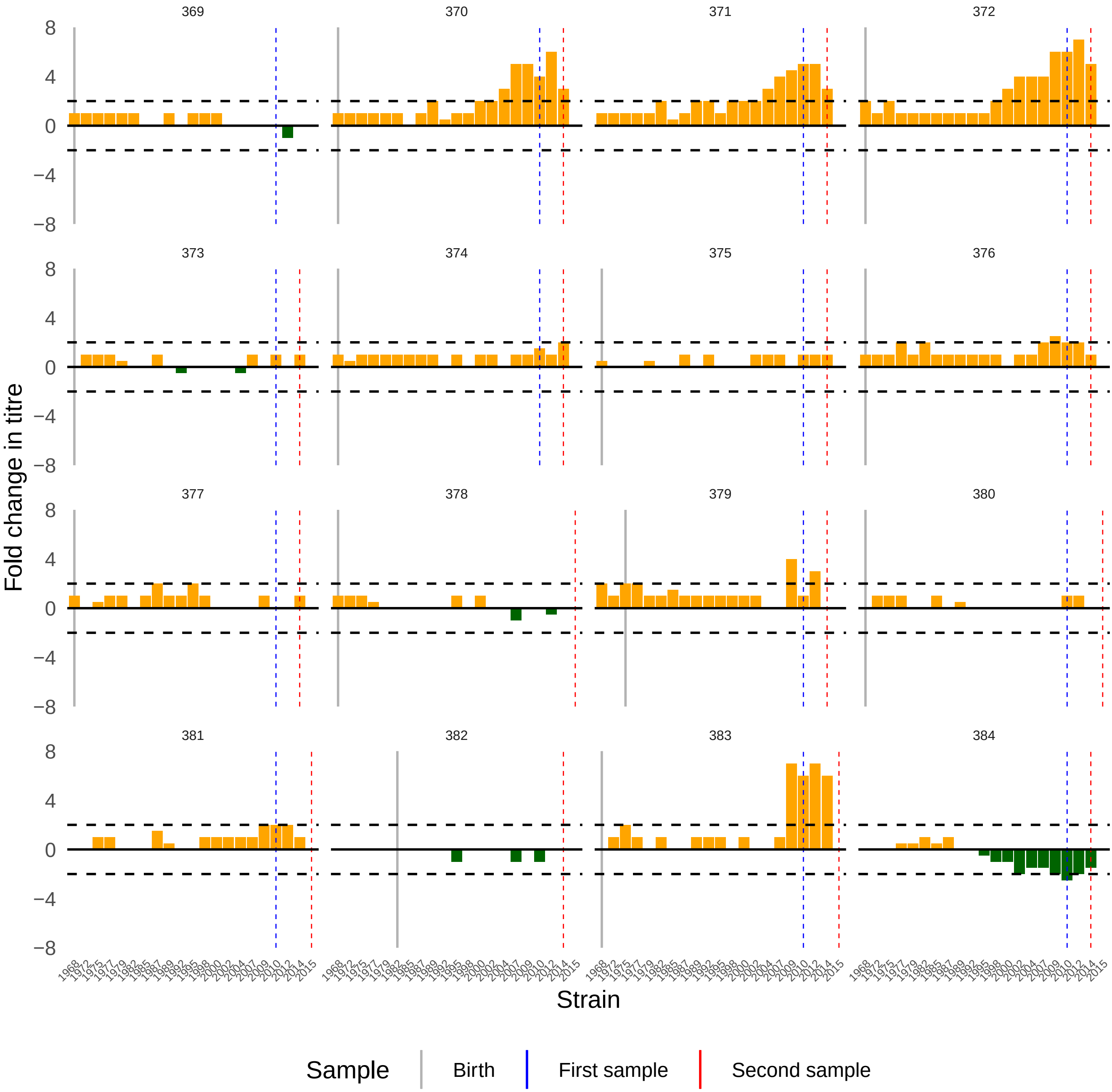
369
370
371
372
373
374
375
376
377
378
379
380
381
382
383
384
8
4
0
−4
−8
Fold change in titre
1968
1972
1975
1977
1979
1982
1985
1987
1989
1992
1995
1998
2000
2002
2004
2007
2009
2010
2012
2014
2015
Strain
Sample
Birth
First sample
Second sample

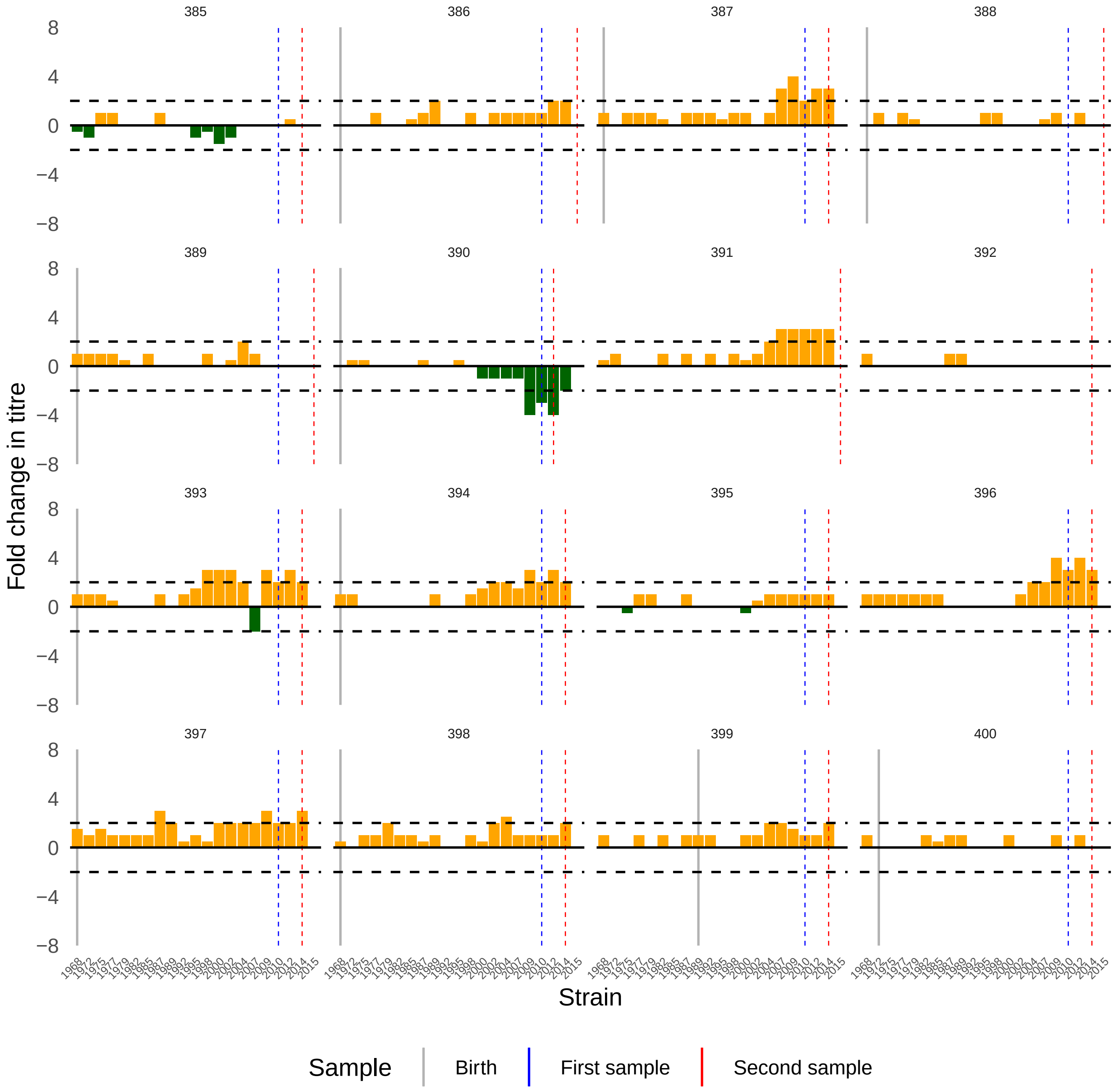
385
386
387
388
389
390
391
392
393
394
395
396
397
398
399
400
Fold change in titre
Strain
Sample
Birth
First sample
Second sample

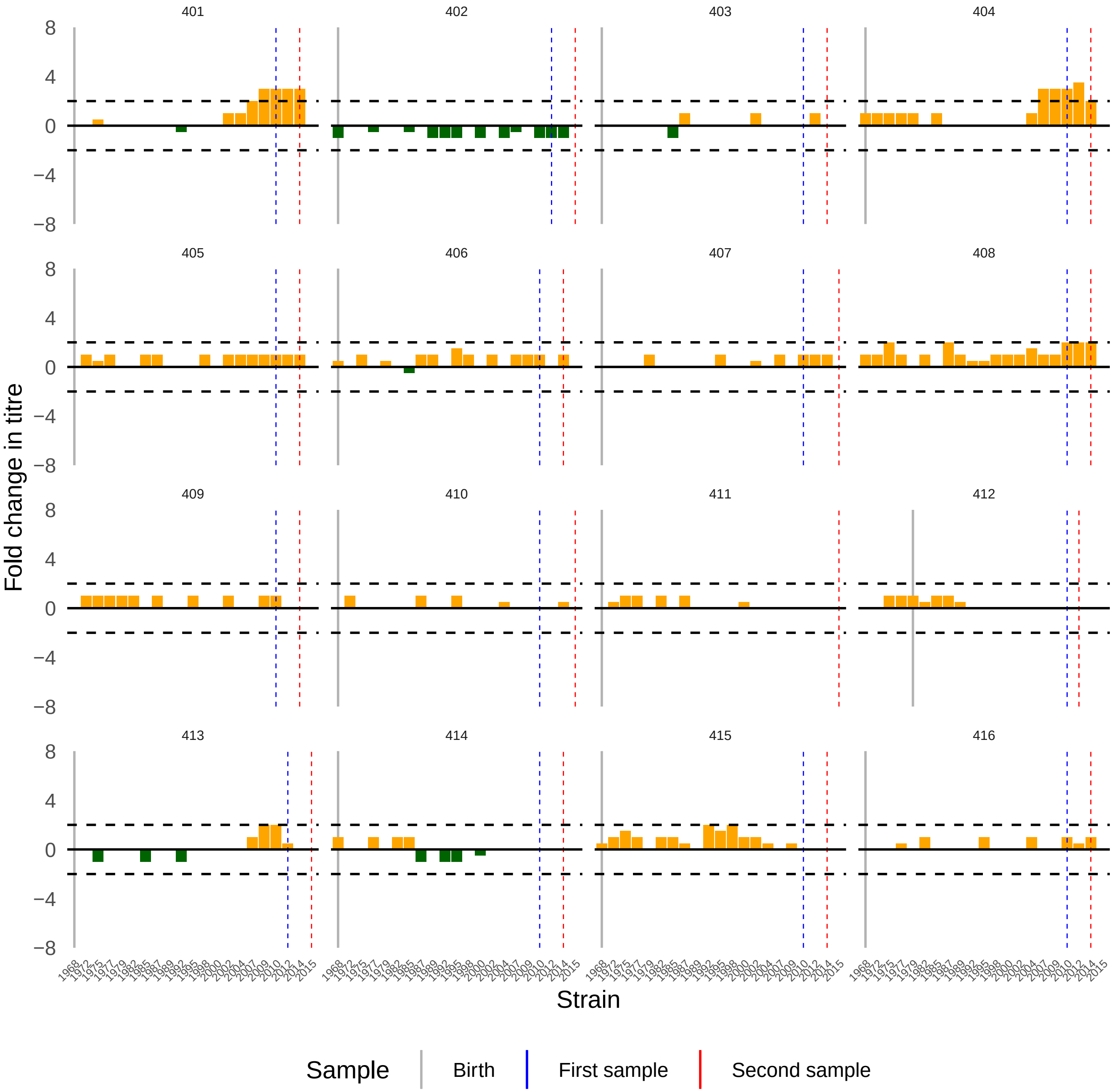

401
402
403
404
405
406
407
408
409
410
411
412
413
414
415
416
Fold change in titre
8
4
0
−4
−8
1968 1972 1975 1977 1979 1982 1985 1987 1989 1992 1995 1998 2000 2002 2004 2007 2009 2010 2012 2014 2015
Strain
Sample
Birth
First sample
Second sample

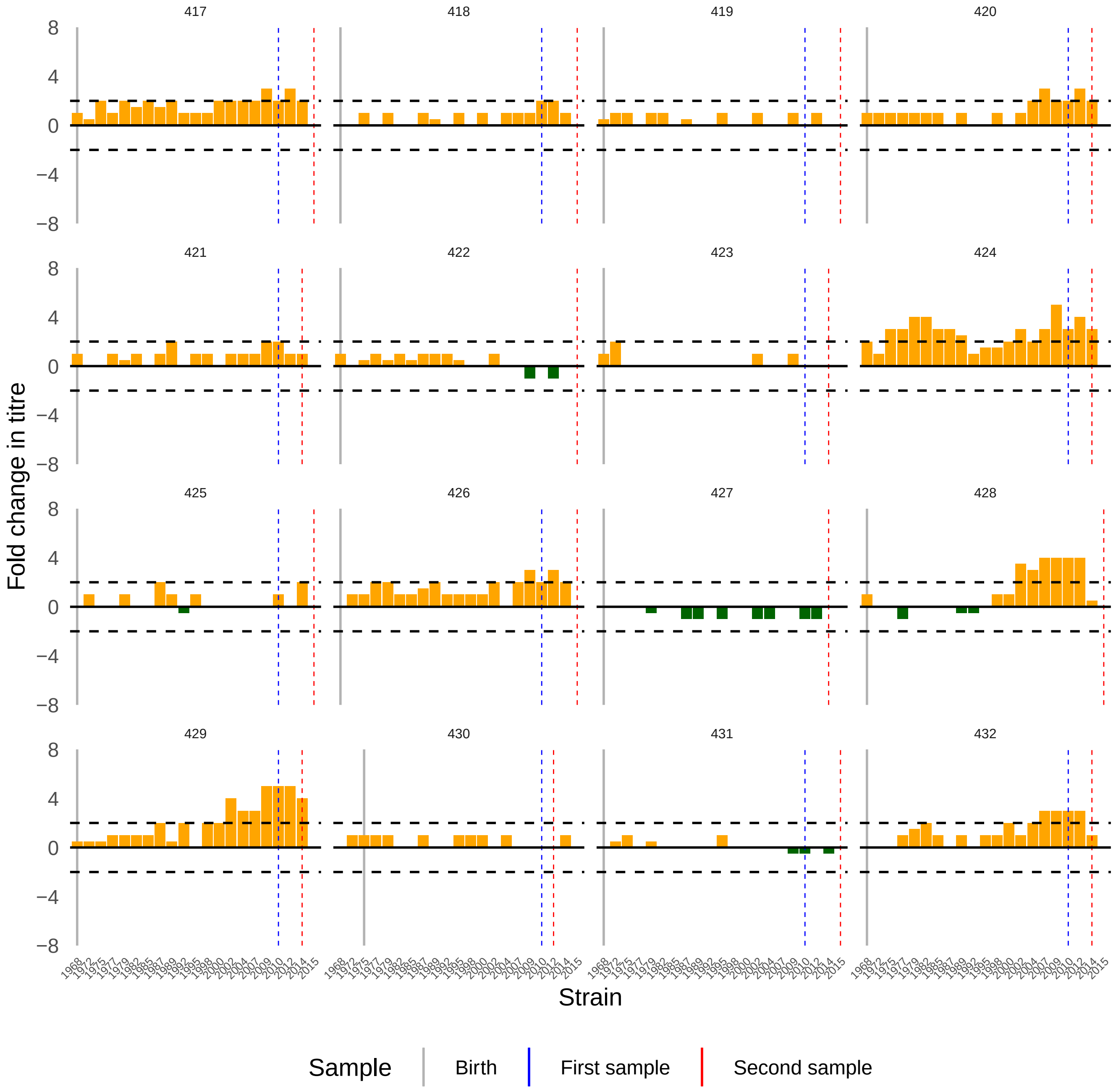

417
418
419
420
421
422
423
424
425
426
427
428
429
430
431
432
Fold change in titre
Strain
Sample
Birth
First sample
Second sample

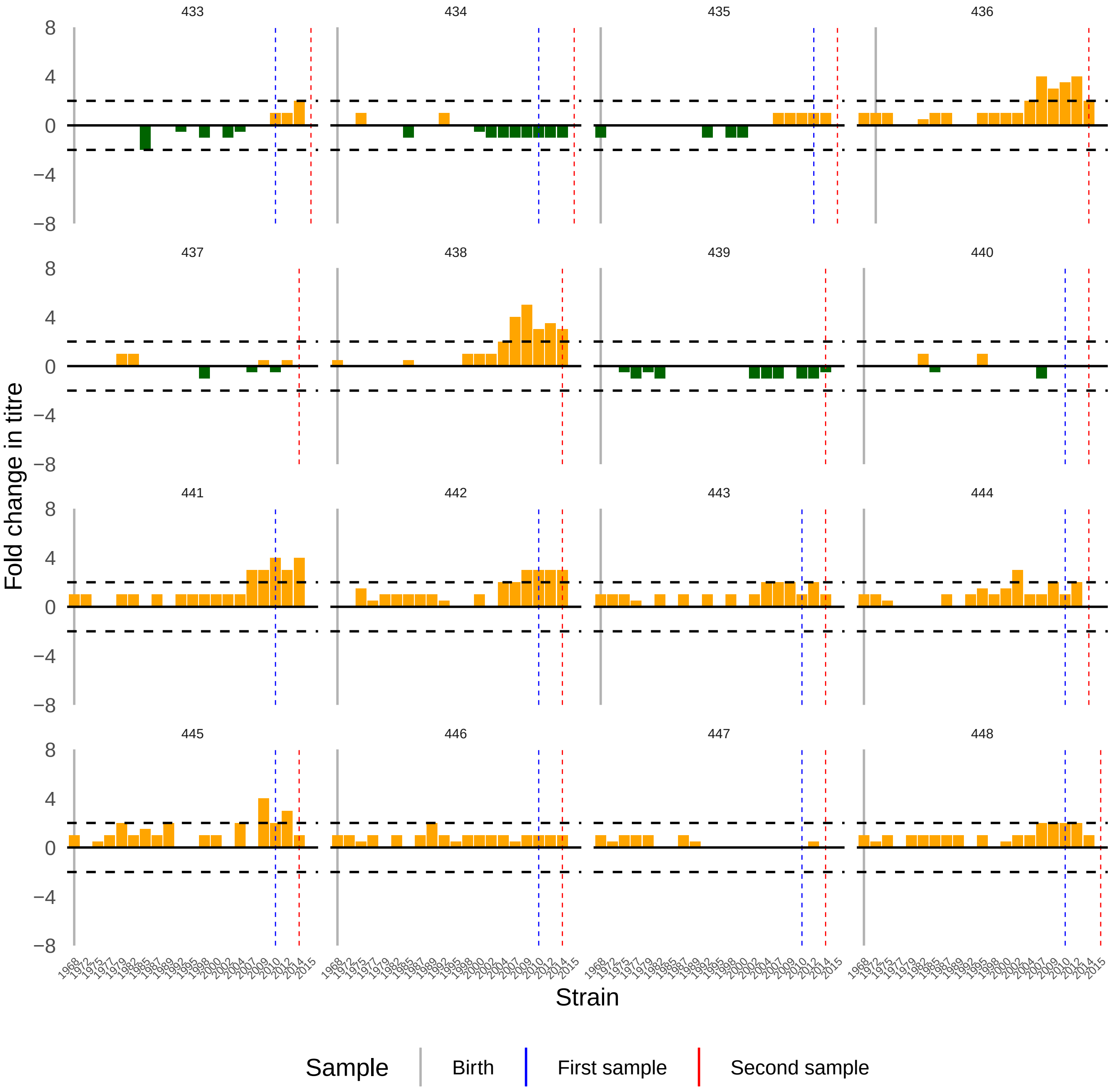
433
434
435
436
437
438
439
440
441
442
443
444
445
446
447
448
Fold change in titre
Strain
Sample
Birth
First sample
Second sample

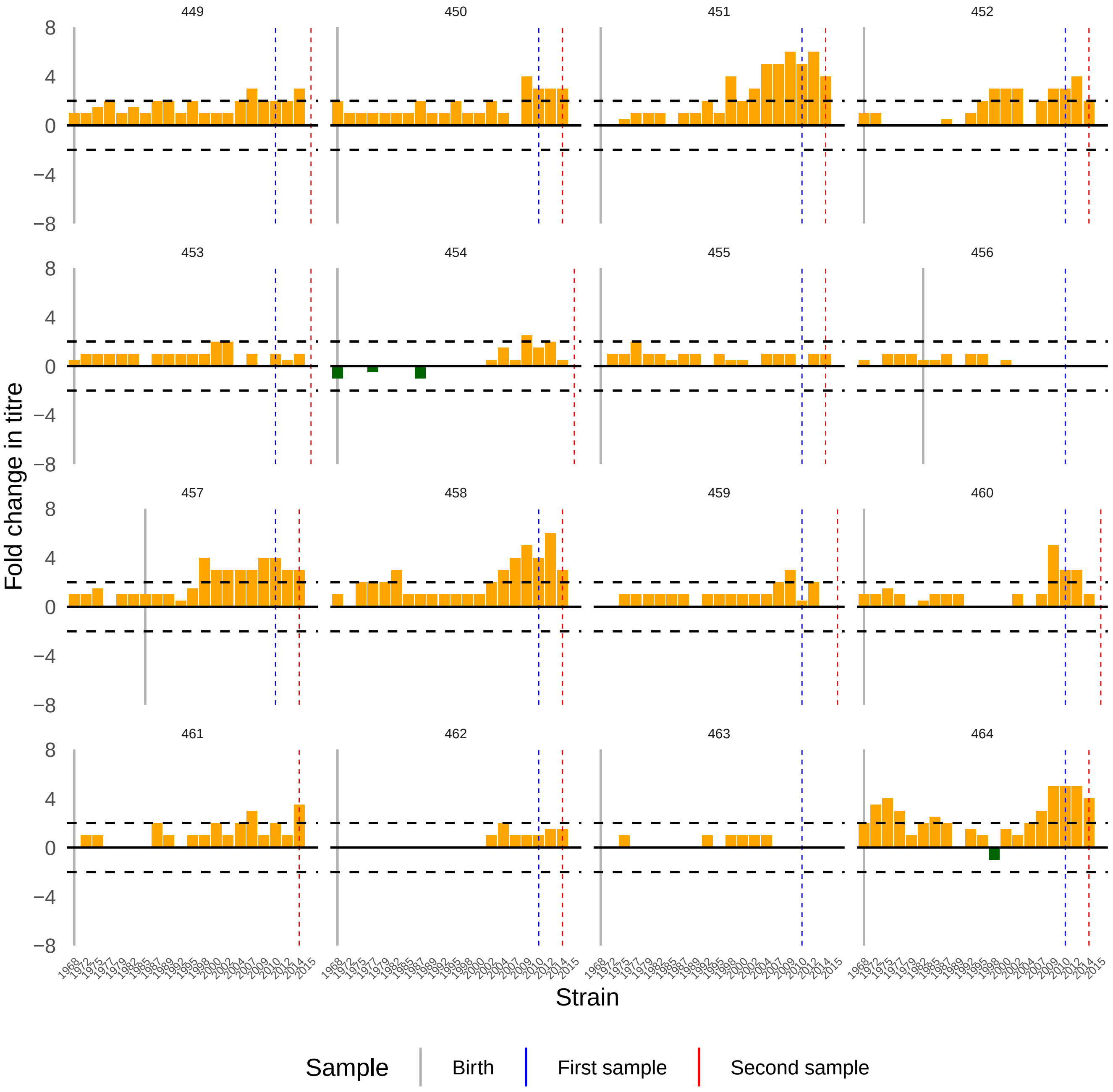
449
450
451
452
453
454
455
456
457
458
459
460
461
462
463
464
Fold change in titre
8
4
0
−4
−8
1968 1972 1975 1977 1979 1982 1985 1987 1989 1992 1995 1998 2000 2002 2004 2007 2009 2010 2012 2014 2015
Strain
Sample
Birth
First sample
Second sample

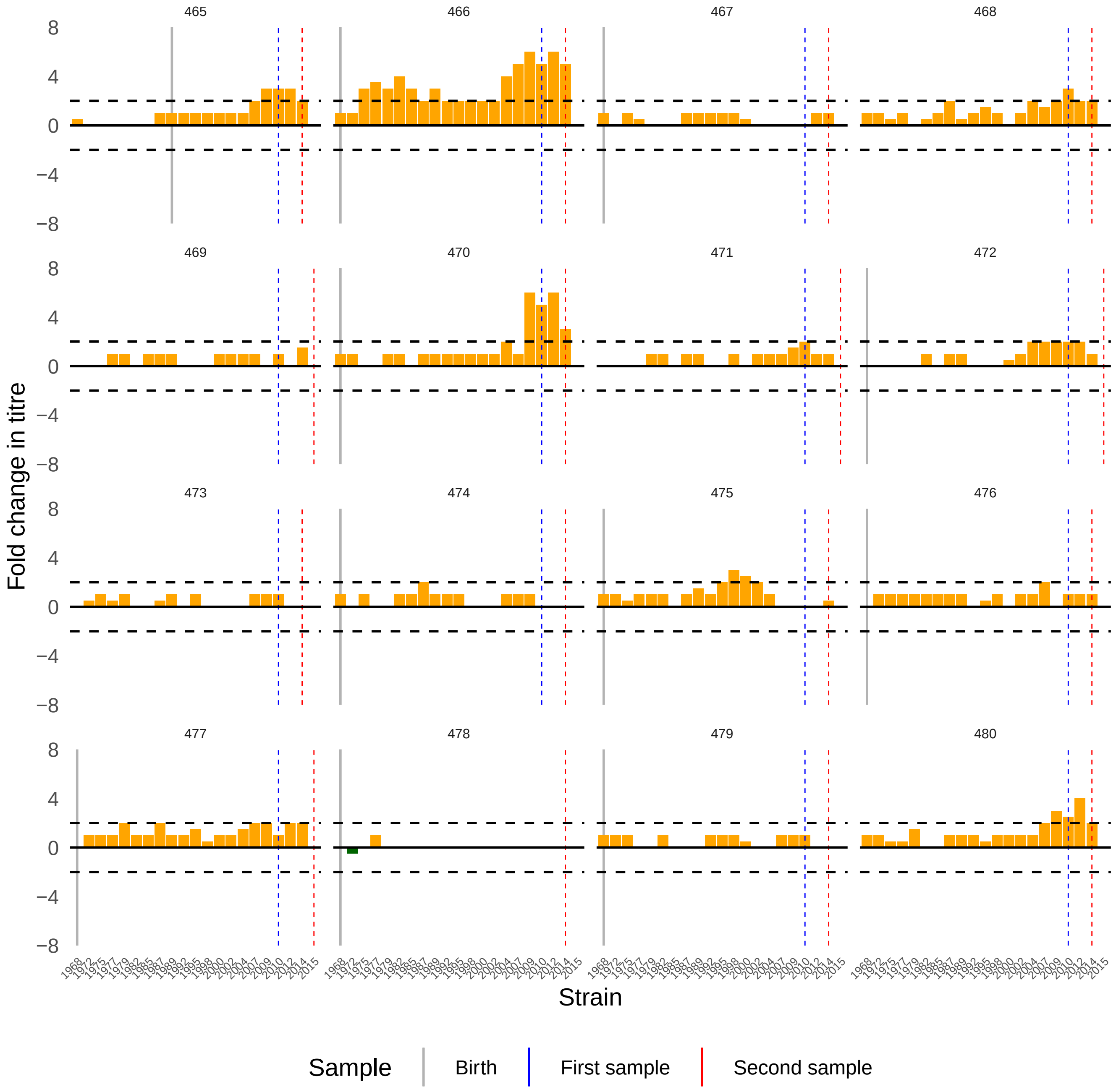
465
466
467
468
469
470
471
472
473
474
475
476
477
478
479
480
8
4
0
−4
−8
Fold change in titre
1968
1972
1975
1977
1979
1982
1985
1987
1989
1992
1995
1998
2000
2002
2004
2007
2009
2010
2012
2014
2015
Strain
Sample
Birth
First sample
Second sample

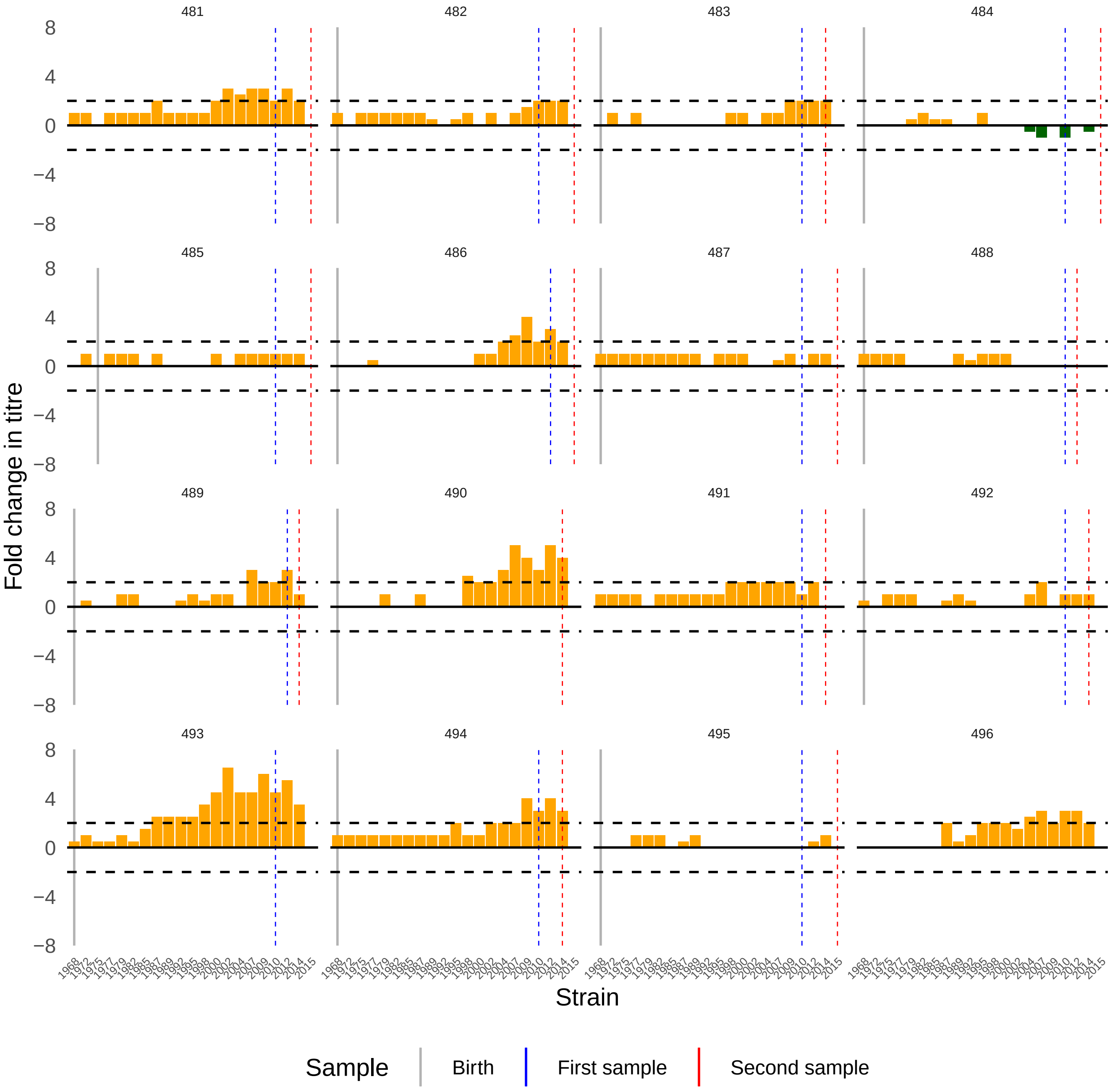

481
482
483
484
485
486
487
488
489
490
491
492
493
494
495
496
Fold change in titre
Strain
Sample
Birth
First sample
Second sample

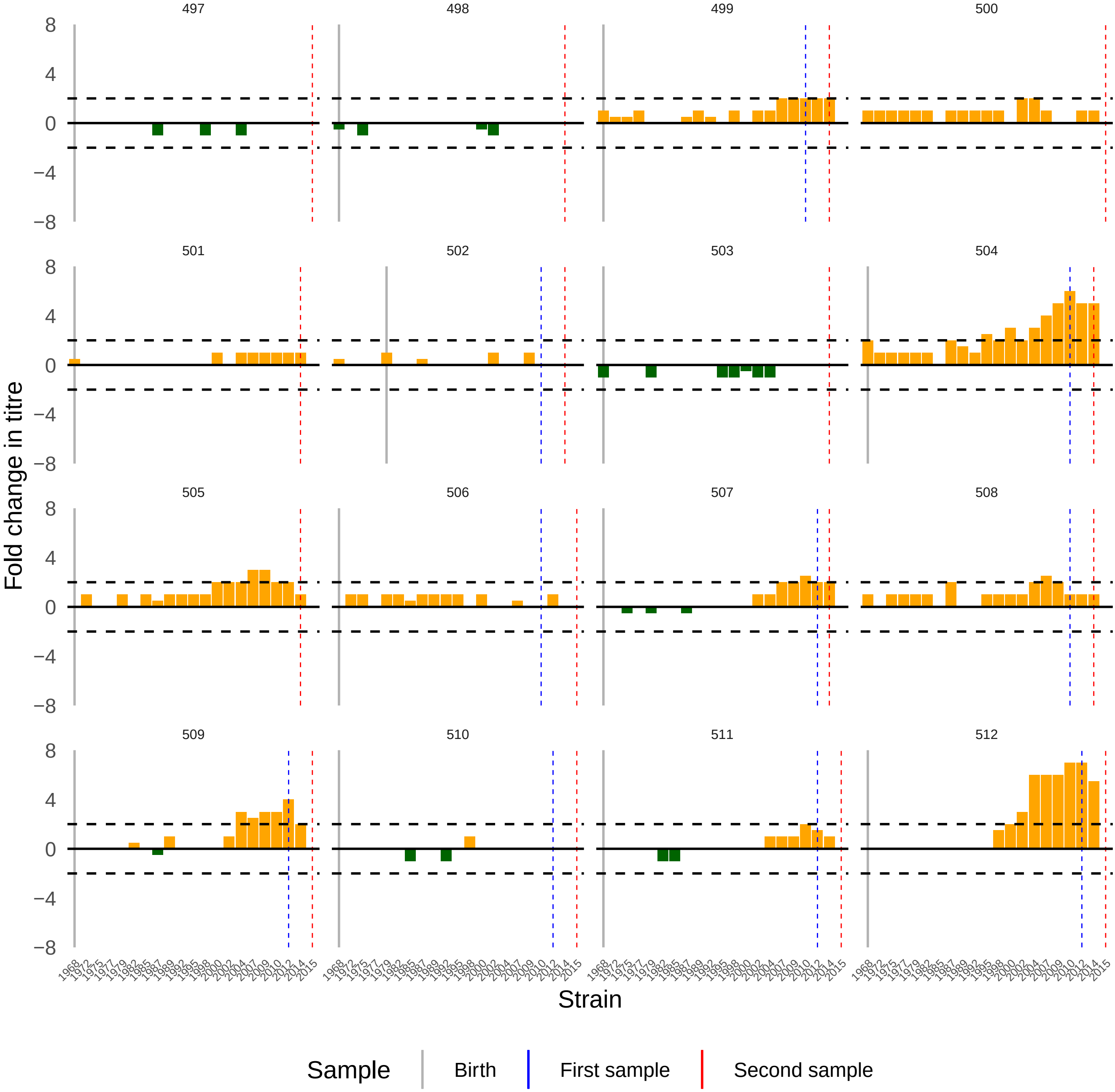

497
498
499
500
501
502
503
504
505
506
507
508
509
510
511
512
8
4
0
−4
−8
1968 1972 1975 1977 1979 1982 1985 1987 1989 1992 1995 1998 2000 2002 2004 2007 2009 2010 2012 2014 2015
Fold change in titre
Strain
Sample
Birth
First sample
Second sample

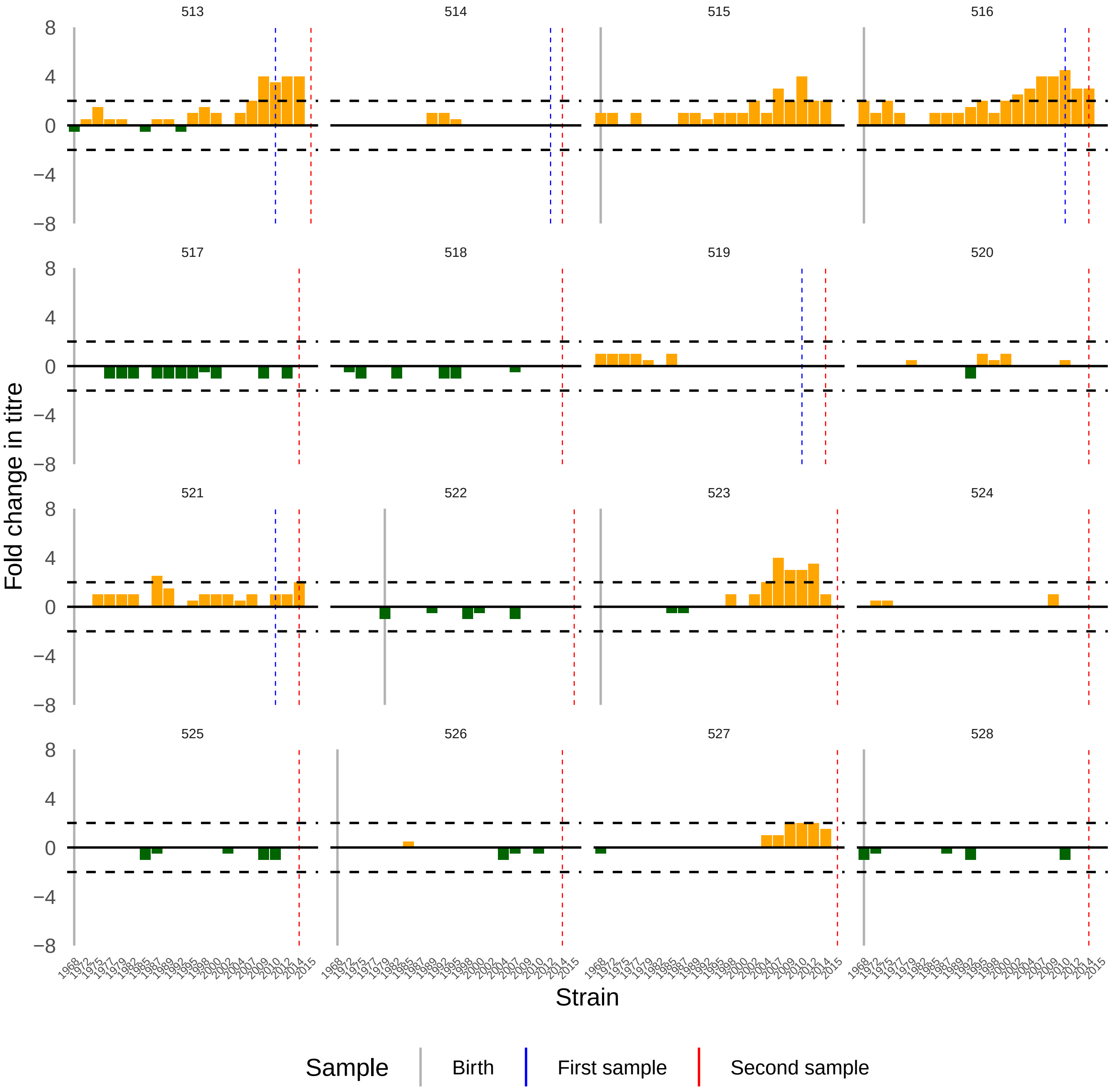

513
514
515
516
517
518
519
520
521
522
523
524
525
526
527
528
Fold change in titre
8
4
0
−4
−8
1968 1972 1975 1977 1979 1982 1985 1987 1989 1992 1995 1998 2000 2002 2004 2007 2009 2010 2012 2014 2015
Strain
Sample
Birth
First sample
Second sample

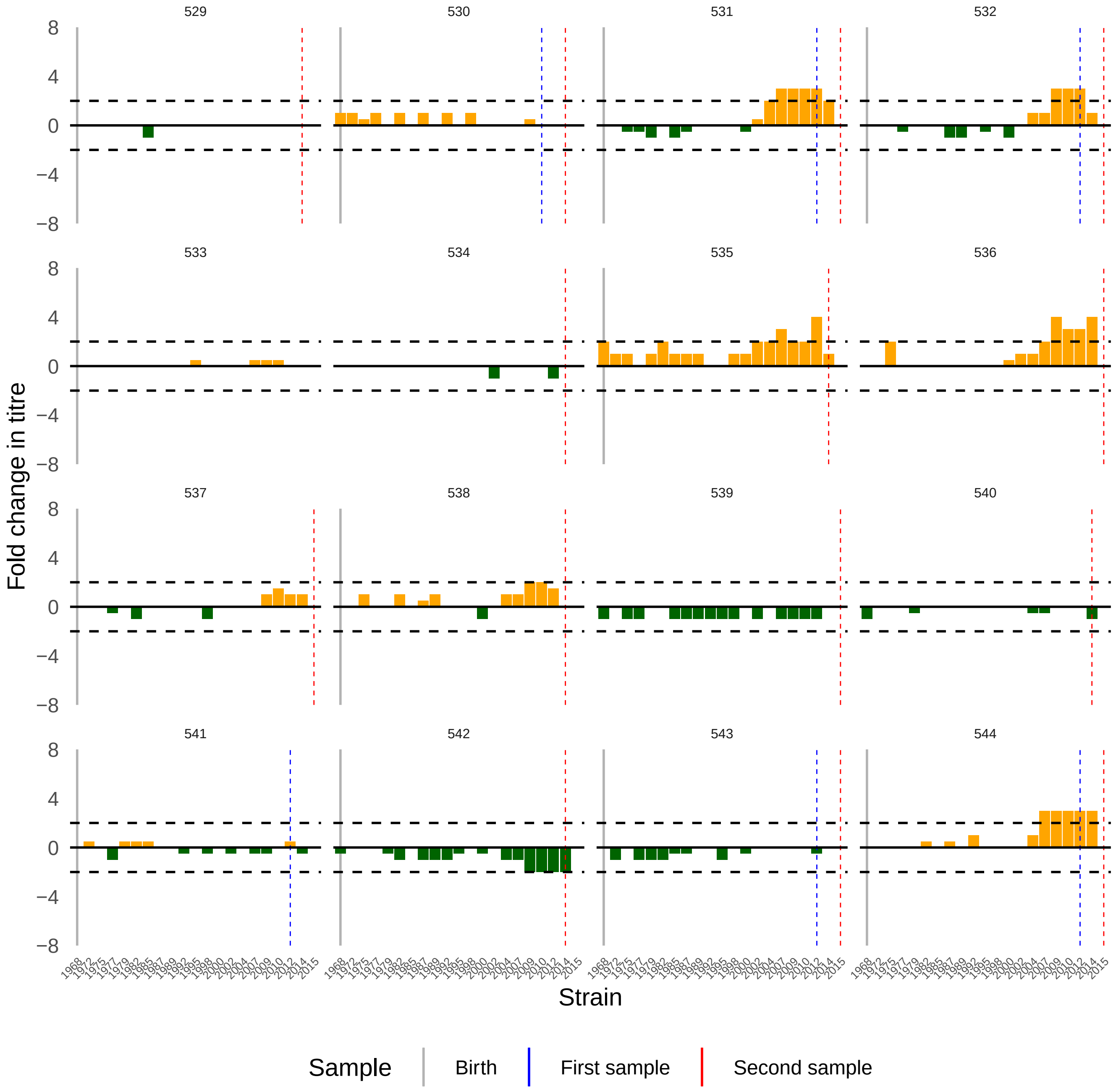

529
530
531
532
533
534
535
536
537
538
539
540
541
542
543
544
8
4
0
−4
−8
Fold change in titre
1968 1972 1975 1977 1979 1982 1985 1987 1989 1992 1995 1998 2000 2002 2004 2007 2009 2010 2012 2014 2015
Strain
Sample
Birth
First sample
Second sample

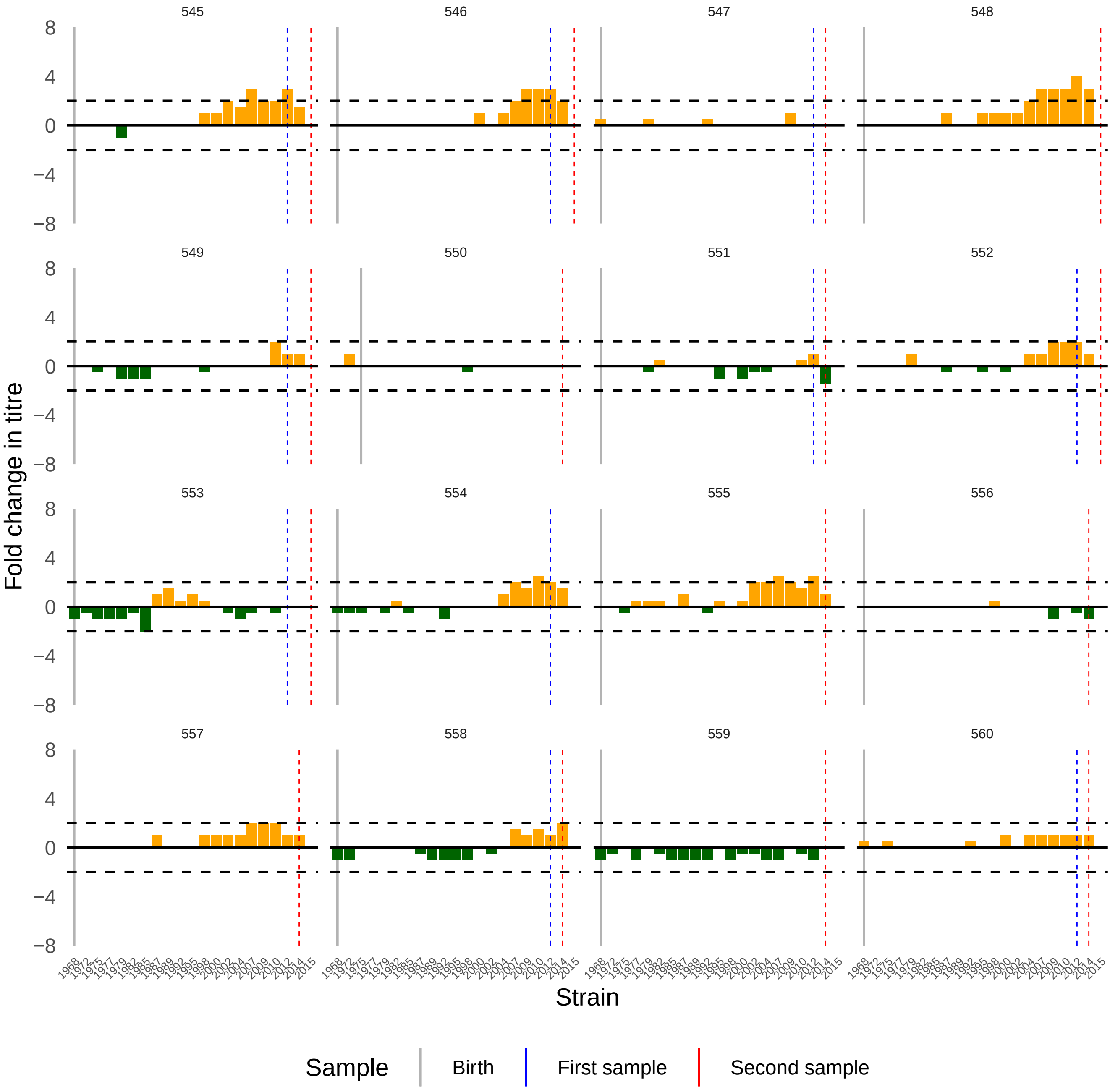

545
546
547
548
549
550
551
552
553
554
555
556
557
558
559
560
8
4
0
−4
−8
Fold change in titre
1968 1972 1975 1977 1979 1982 1985 1987 1989 1992 1995 1998 2000 2002 2004 2007 2009 2010 2012 2014 2015
Strain
Sample
Birth
First sample
Second sample

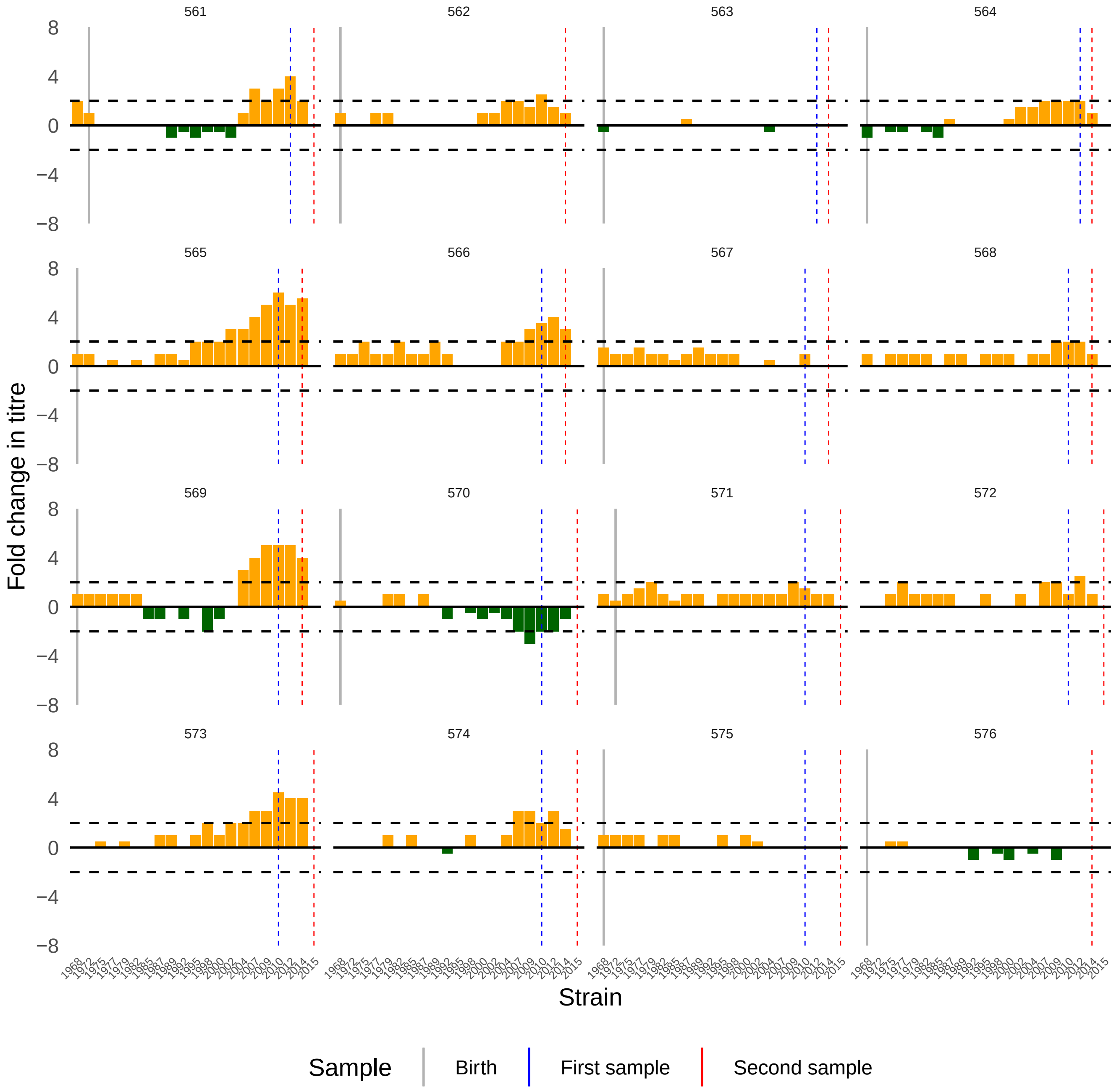
561
562
563
564
565
566
567
568
569
570
571
572
573
574
575
576
Fold change in titre
Strain
Sample
Birth
First sample
Second sample

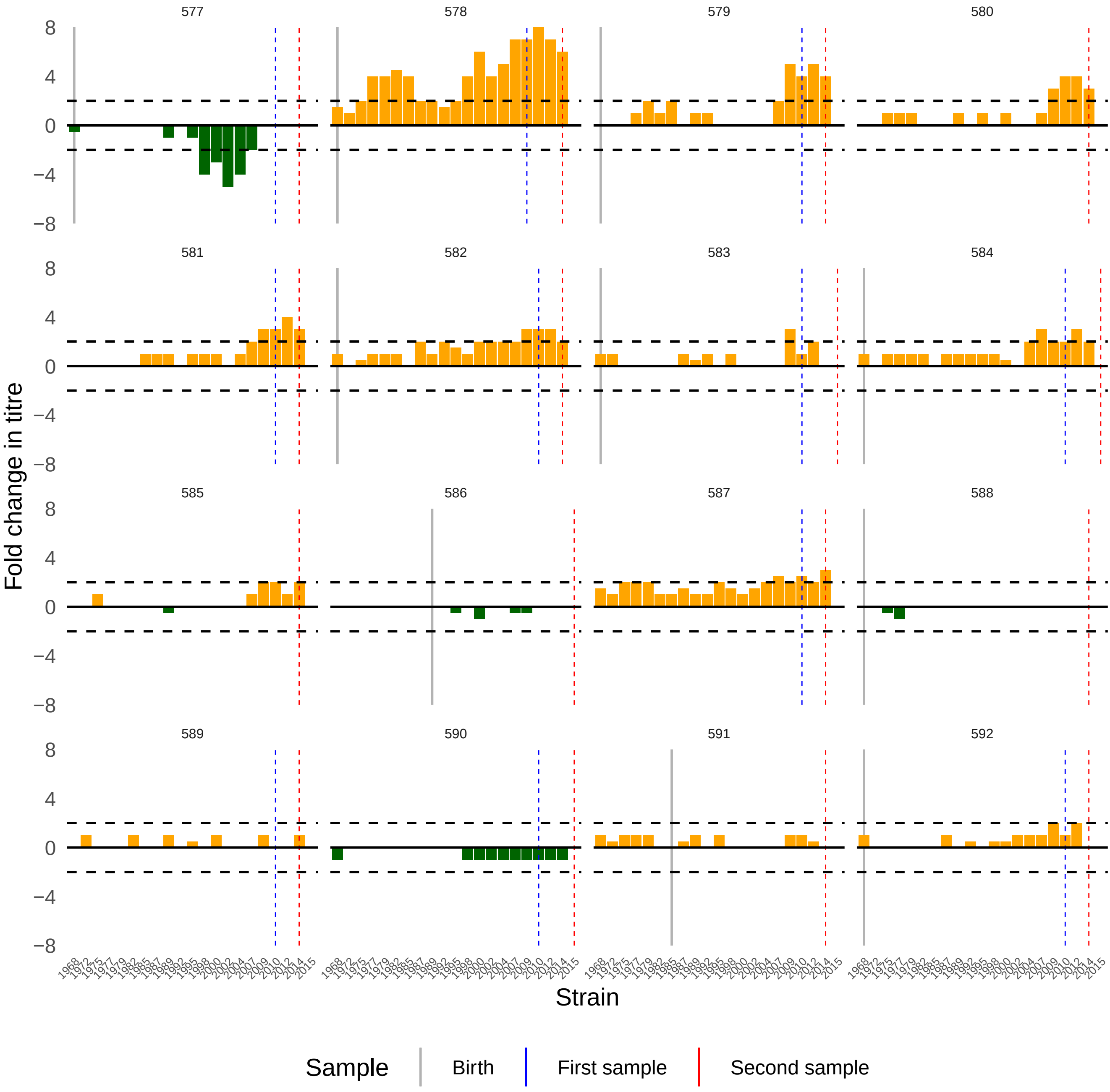
577
578
579
580
581
582
583
584
585
586
587
588
589
590
591
592
Fold change in titre
8
4
0
−4
−8
1968 1972 1975 1977 1979 1982 1985 1987 1989 1992 1995 1998 2000 2002 2004 2007 2009 2010 2012 2014 2015
Strain
Sample
Birth
First sample
Second sample

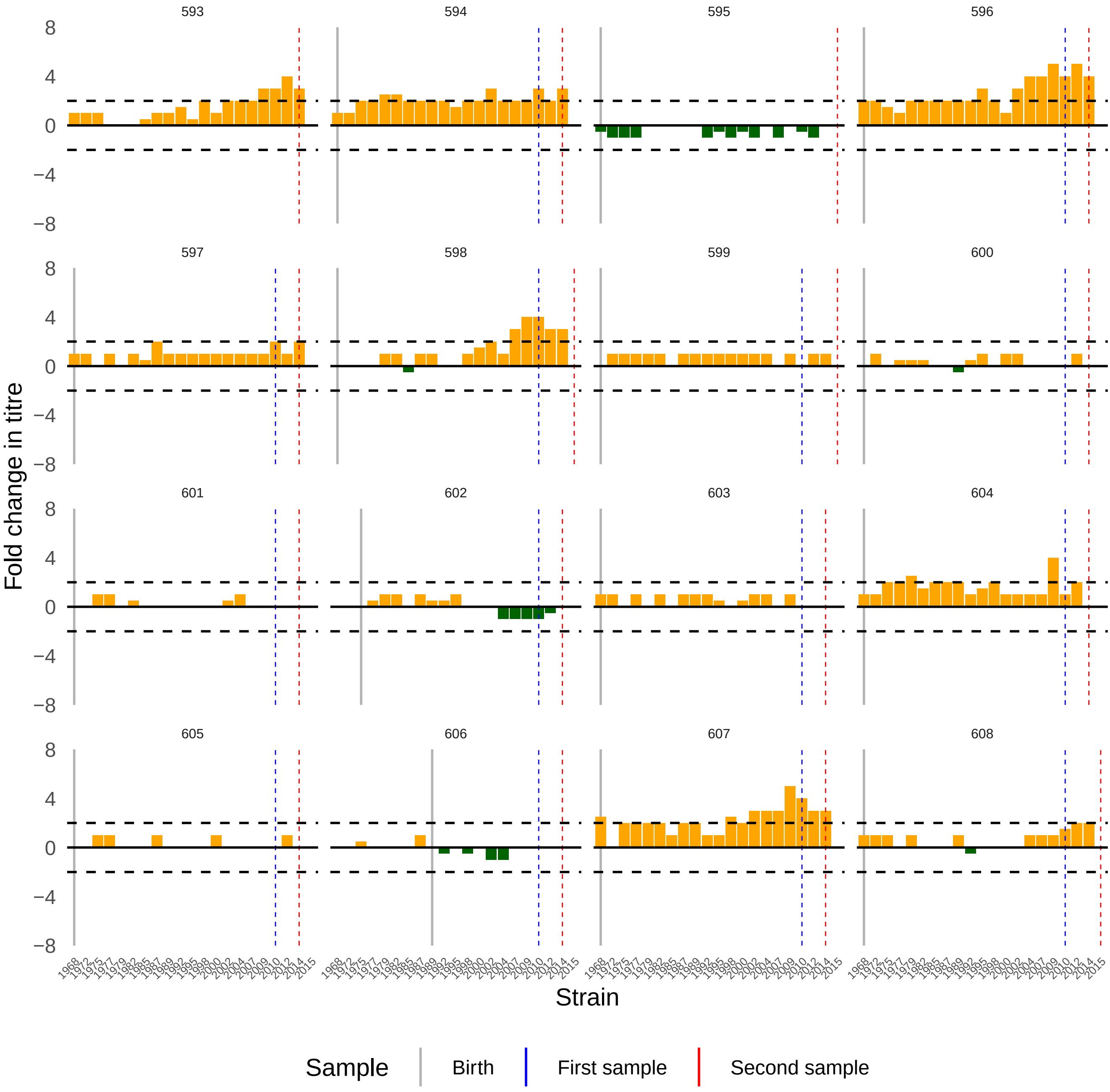

593
594
595
596
597
598
599
600
601
602
603
604
605
606
607
608
Fold change in titre
Strain
Sample
Birth
First sample
Second sample

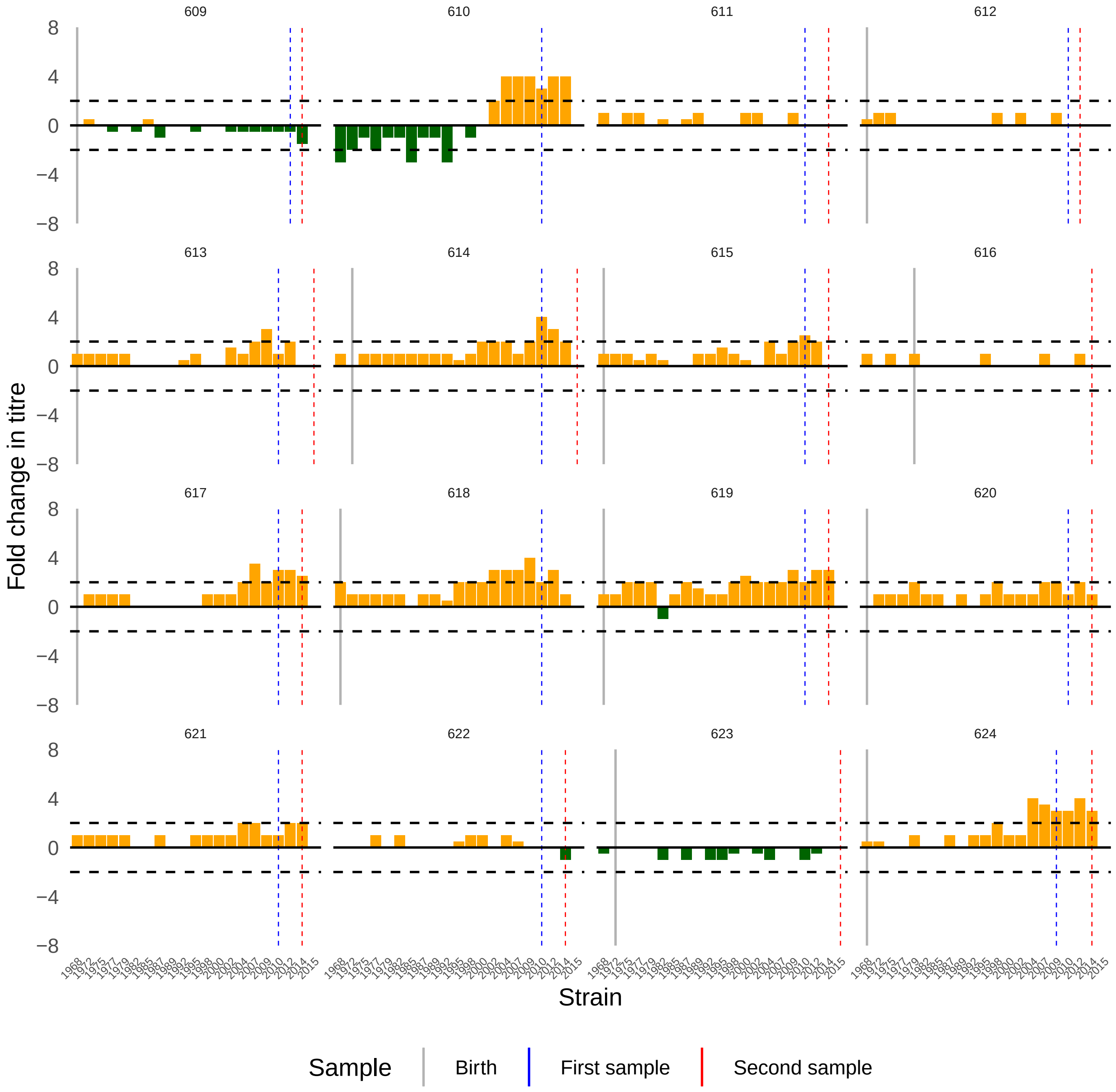
609
610
611
612
613
614
615
616
617
618
619
620
621
622
623
624
Fold change in titre
8
4
0
−4
−8
1968
1972
1975
1977
1979
1982
1985
1987
1989
1992
1995
1998
2000
2002
2004
2007
2009
2010
2012
2014
2015
Strain
Sample
Birth
First sample
Second sample

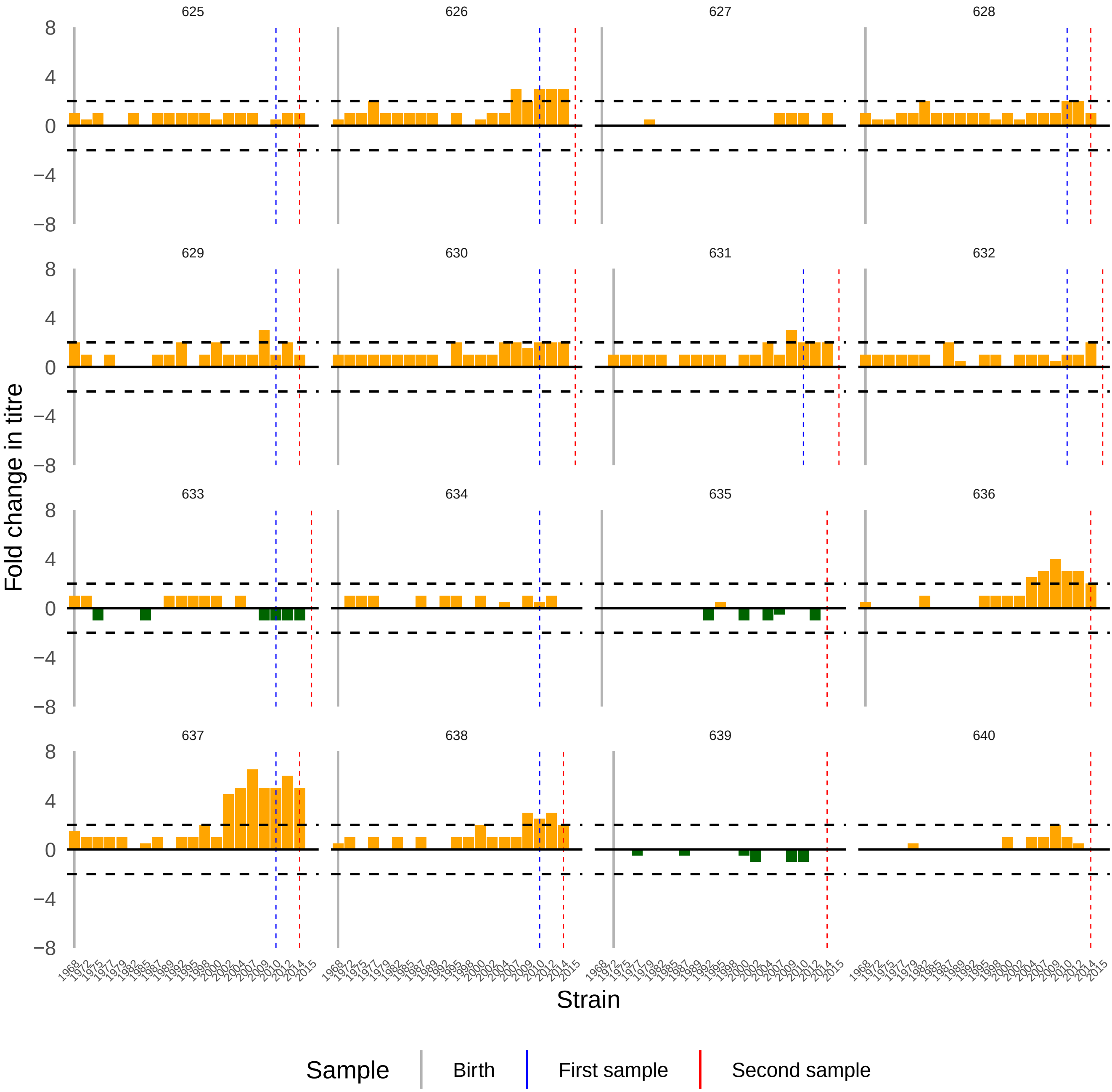
625
626
627
628
629
630
631
632
633
634
635
636
637
638
639
640
8
4
0
−4
−8
Fold change in titre
1968 1972 1975 1977 1979 1982 1985 1987 1989 1992 1995 1998 2000 2002 2004 2007 2009 2010 2012 2014 2015
Strain
Sample
Birth
First sample
Second sample

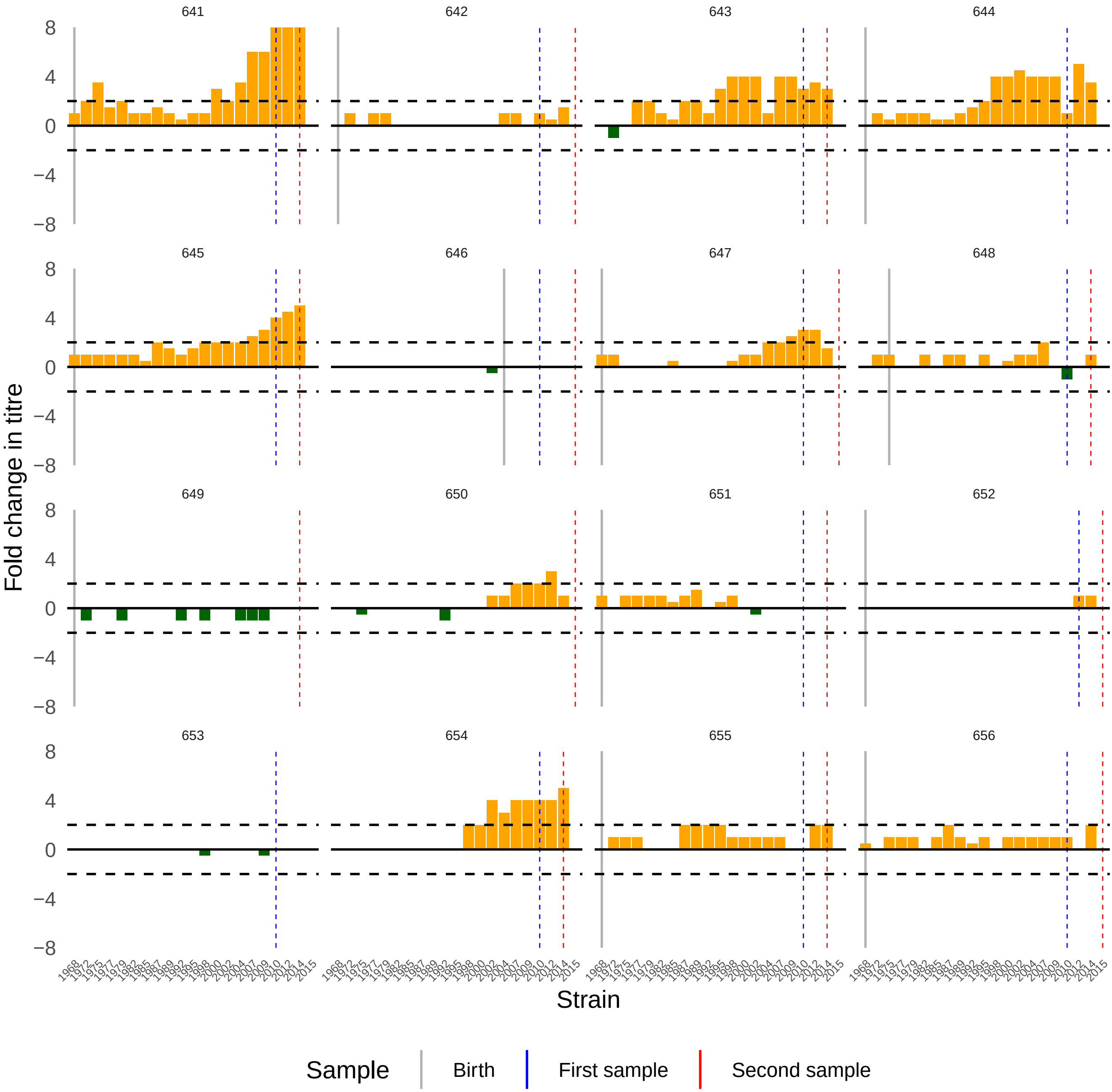
641
642
643
644
645
646
647
648
649
650
651
652
653
654
655
656
8
4
0
−4
−8
Fold change in titre
1968
1972
1975
1977
1979
1982
1985
1987
1989
1992
1995
1998
2000
2002
2004
2007
2009
2010
2012
2014
2015
Strain
Sample
Birth
First sample
Second sample

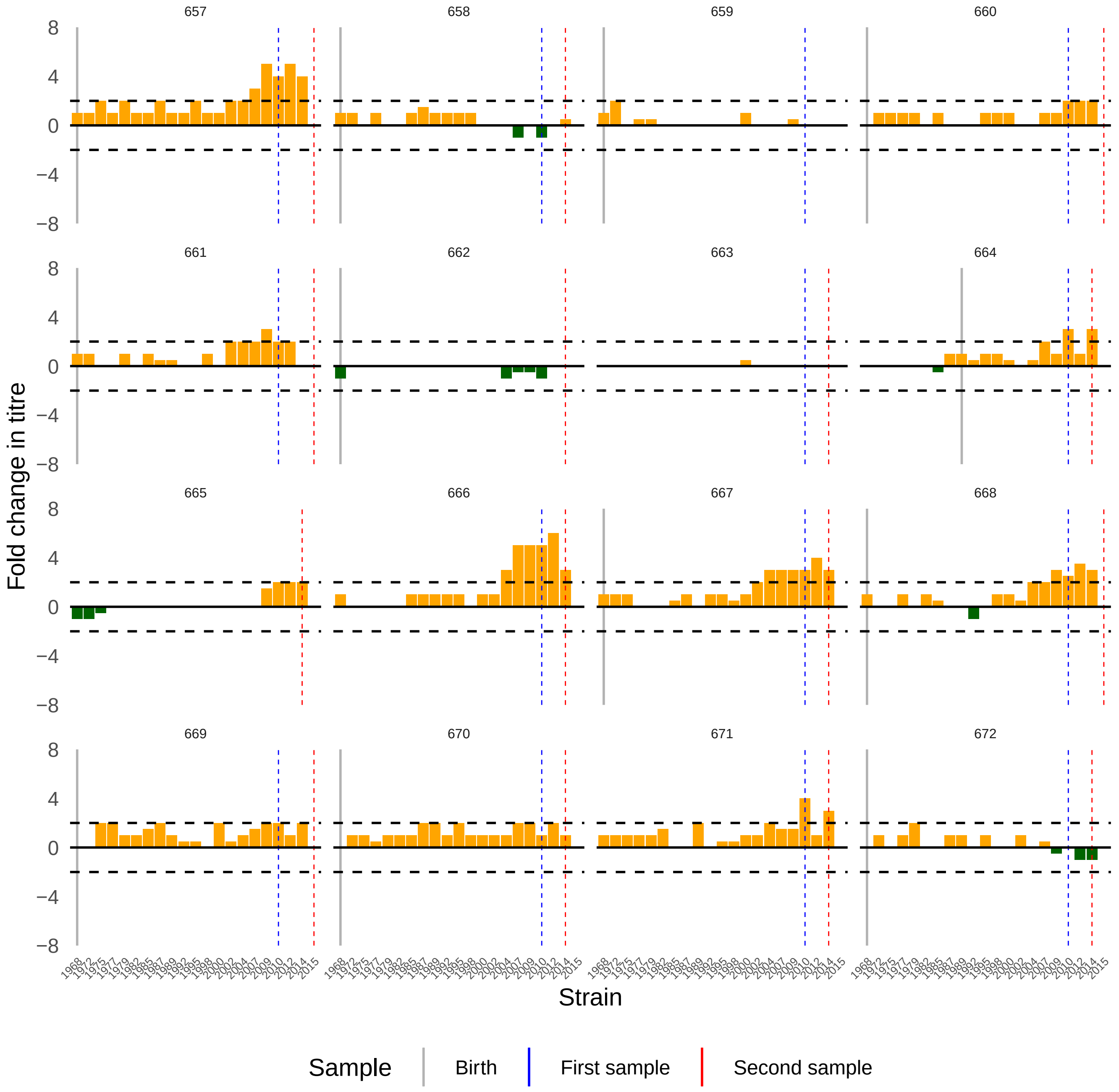

657
658
659
660
661
662
663
664
665
666
667
668
669
670
671
672
8
4
0
−4
−8
Fold change in titre
1968 1972 1975 1977 1979 1982 1985 1987 1989 1992 1995 1998 2000 2002 2004 2007 2009 2010 2012 2014 2015
Strain
Sample
Birth
First sample
Second sample

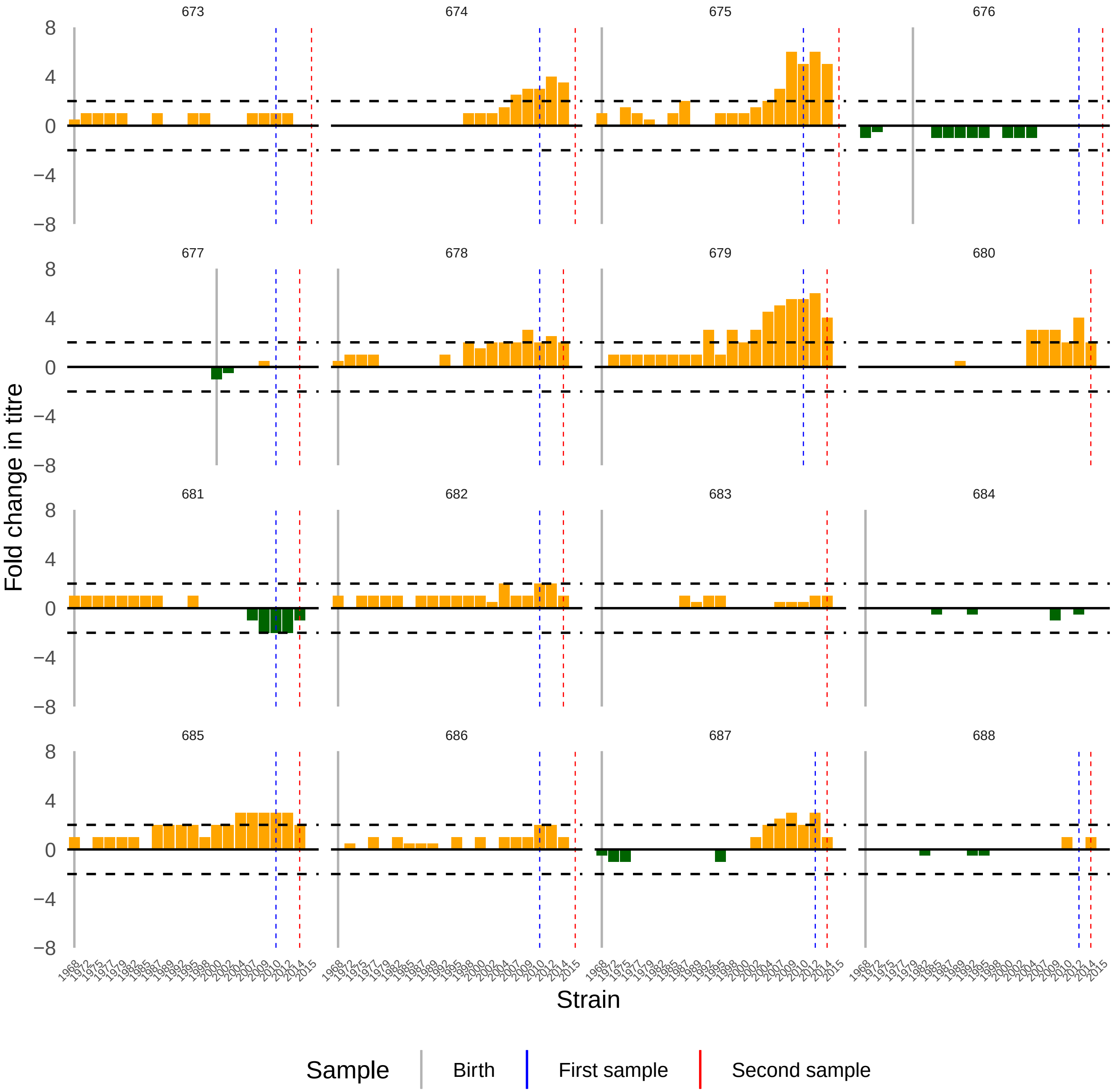

673
674
675
676
677
678
679
680
681
682
683
684
685
686
687
688
Fold change in titre
Strain
1968
1972
1975
1977
1979
1982
1985
1987
1989
1992
1995
1998
2000
2002
2004
2007
2009
2010
2012
2014
2015
Sample
Birth
First sample
Second sample

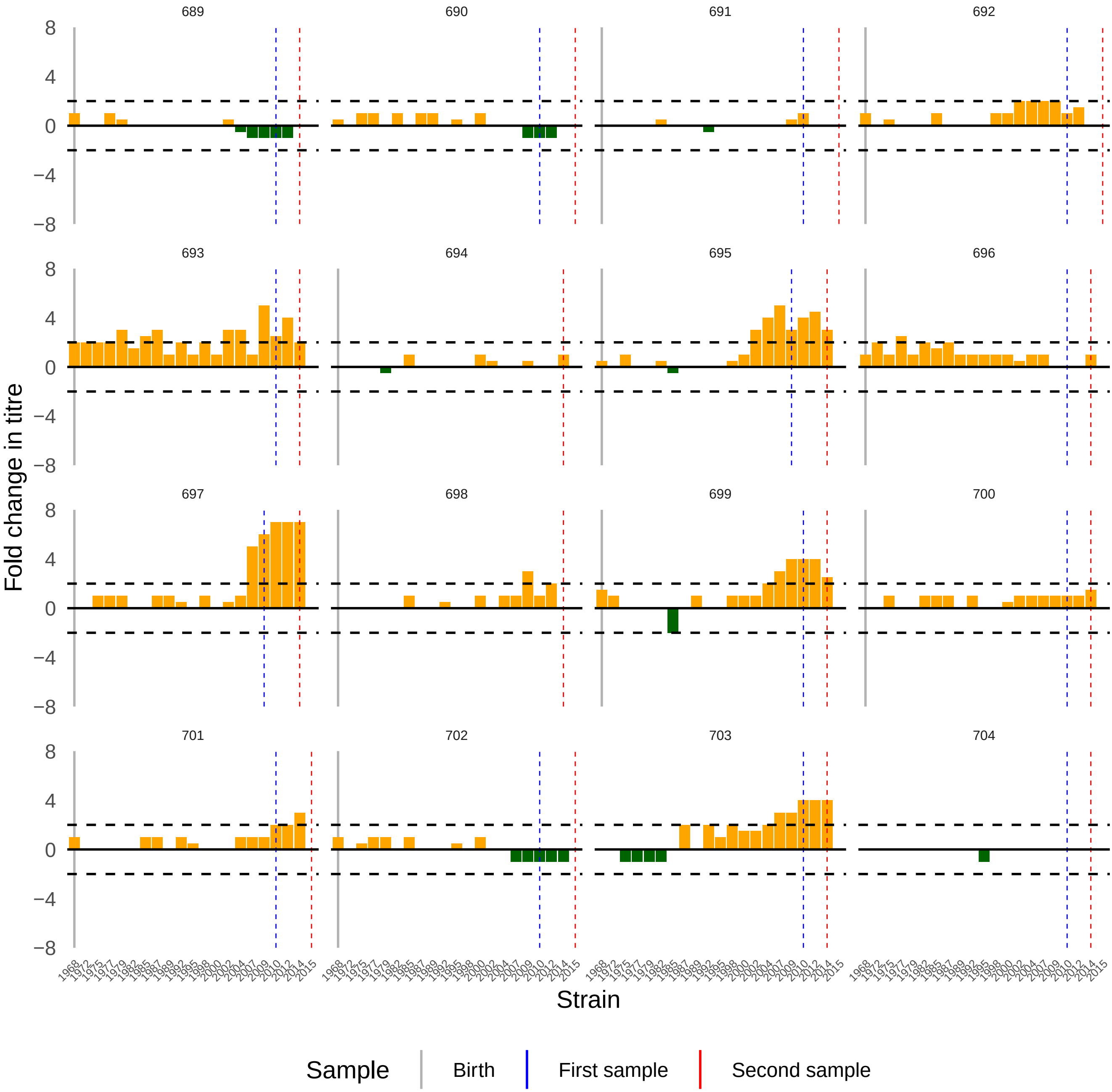
689
690
691
692
693
694
695
696
697
698
699
700
701
702
703
704
8
4
0
−4
−8
1968
1972
1975
1977
1979
1982
1985
1987
1989
1992
1995
1998
2000
2002
2004
2007
2009
2010
2012
2014
2015
Fold change in titre
Strain
Sample
Birth
First sample
Second sample

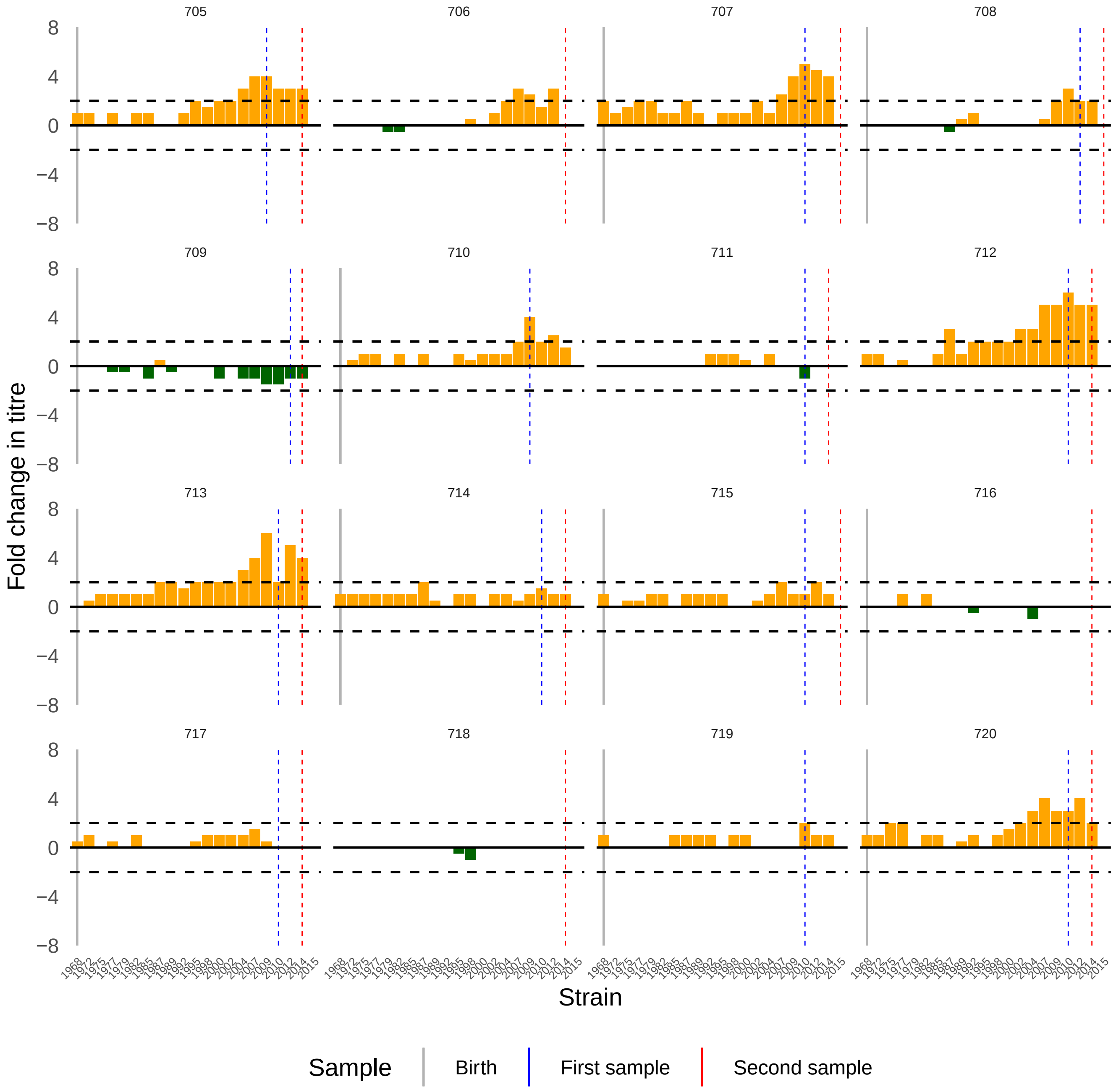

705
706
707
708
709
710
711
712
713
714
715
716
717
718
719
720
Fold change in titre
8
4
0
−4
−8
1968 1972 1975 1977 1979 1982 1985 1987 1989 1992 1995 1998 2000 2002 2004 2007 2009 2010 2012 2014 2015
Strain
Sample
Birth
First sample
Second sample

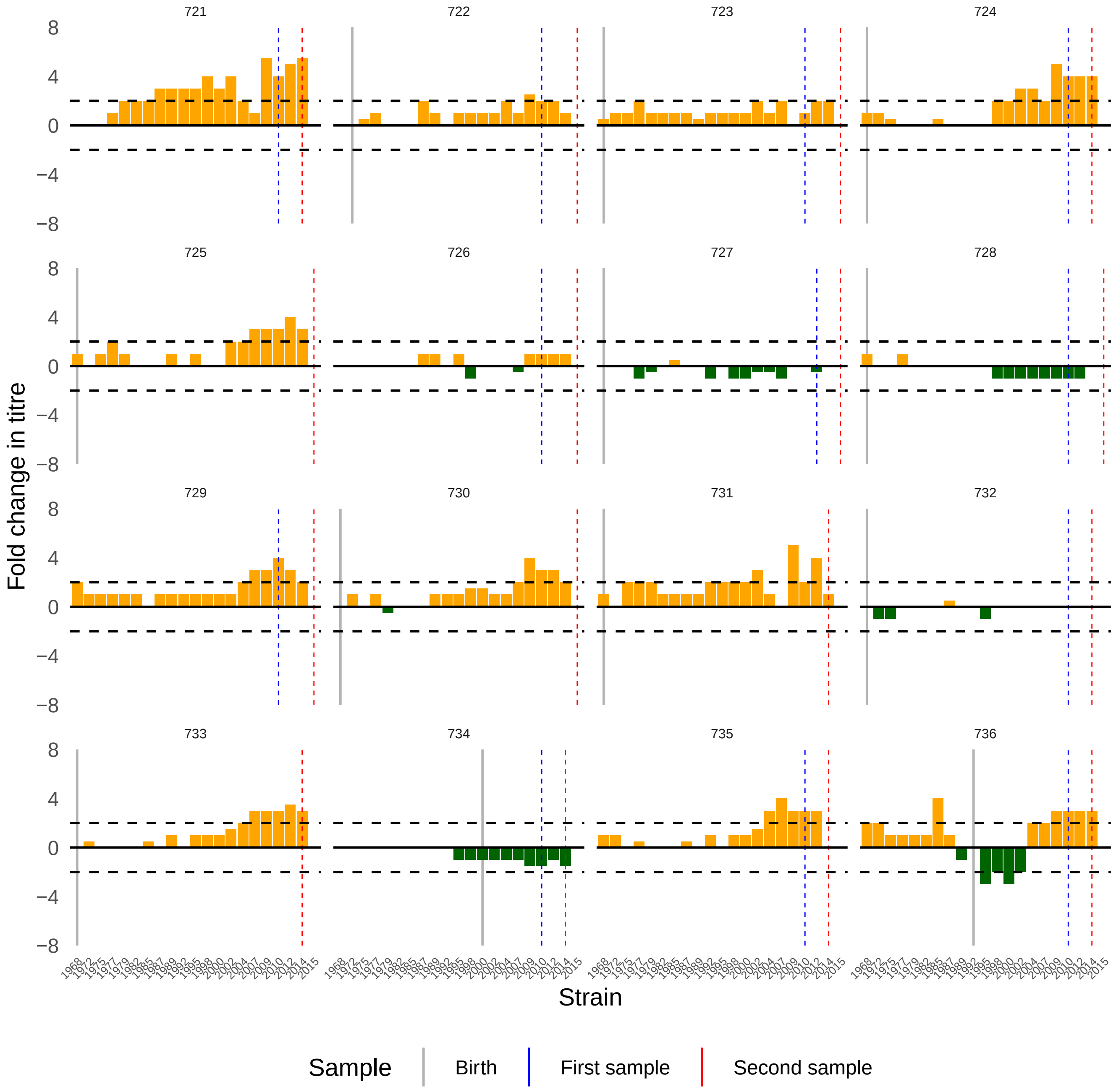

721
722
723
724
725
726
727
728
729
730
731
732
733
734
735
736
8
4
0
−4
−8
Fold change in titre
1968 1972 1975 1977 1979 1982 1985 1987 1989 1992 1995 1998 2000 2002 2004 2007 2009 2010 2012 2014 2015
Strain
Sample
Birth
First sample
Second sample

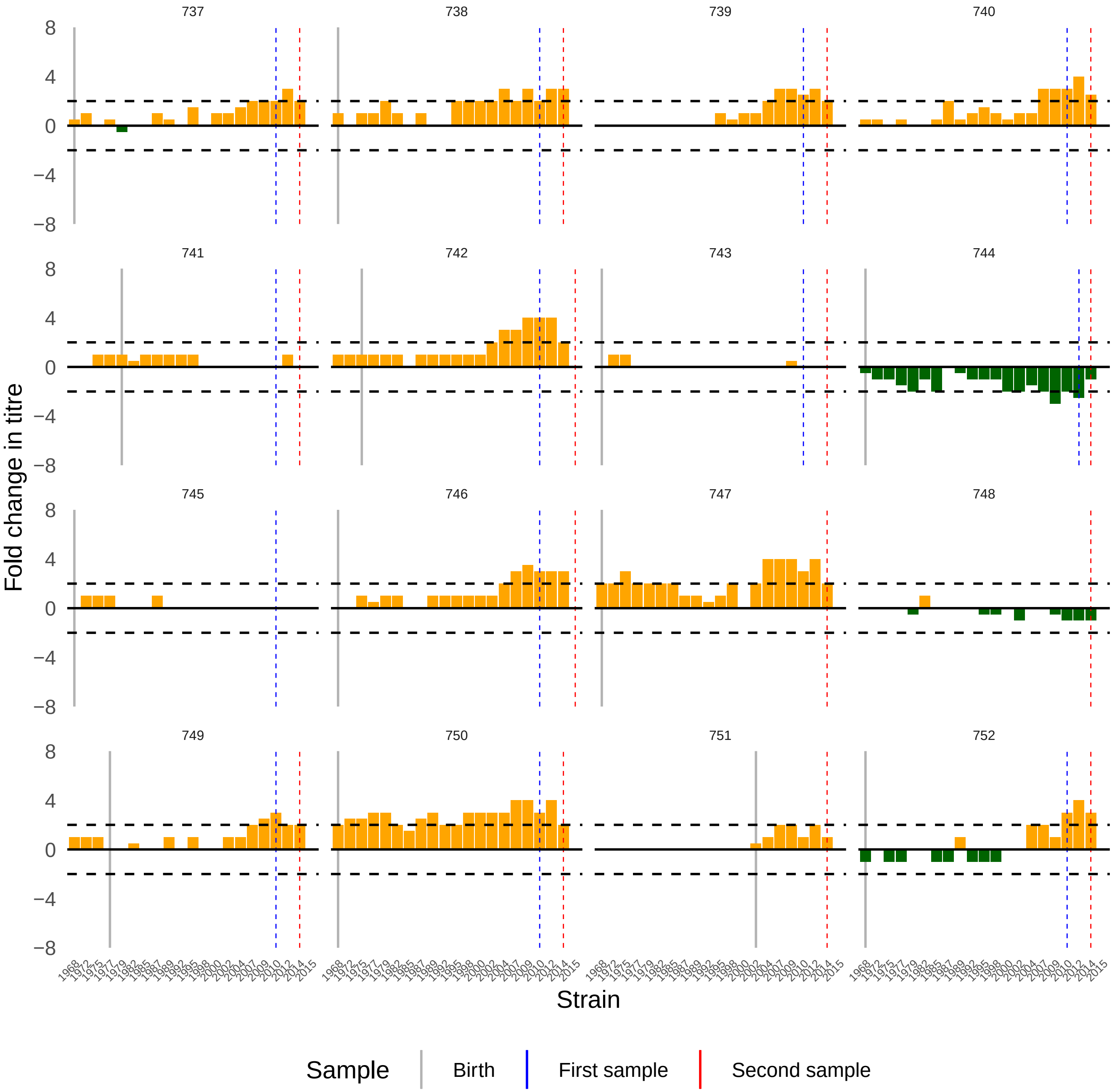
737
738
739
740
741
742
743
744
745
746
747
748
749
750
751
752
Fold change in titre
Strain
Sample
Birth
First sample
Second sample

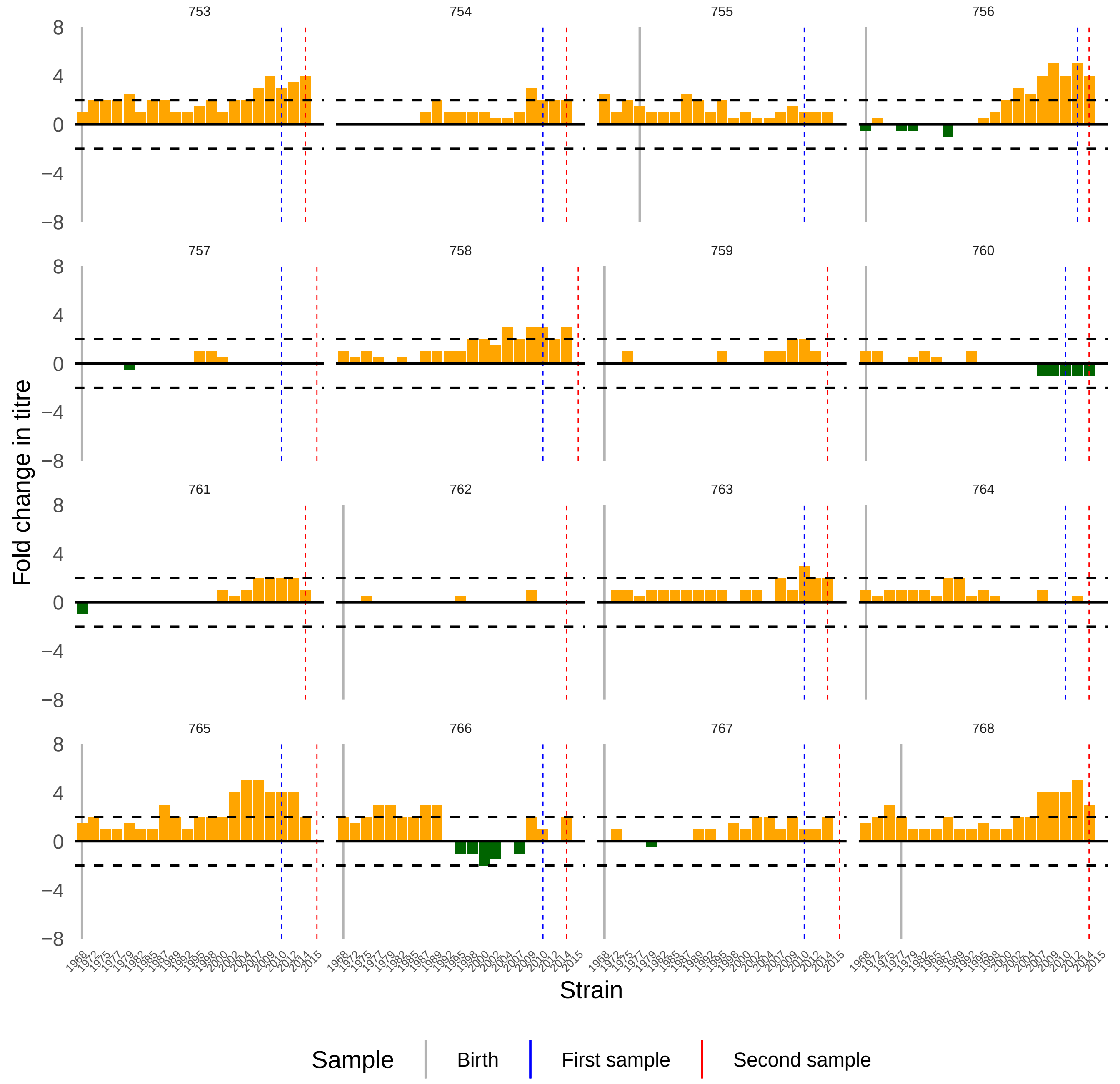

753
754
755
756
757
758
759
760
761
762
763
764
765
766
767
768
Fold change in titre
Strain
Sample
Birth
First sample
Second sample

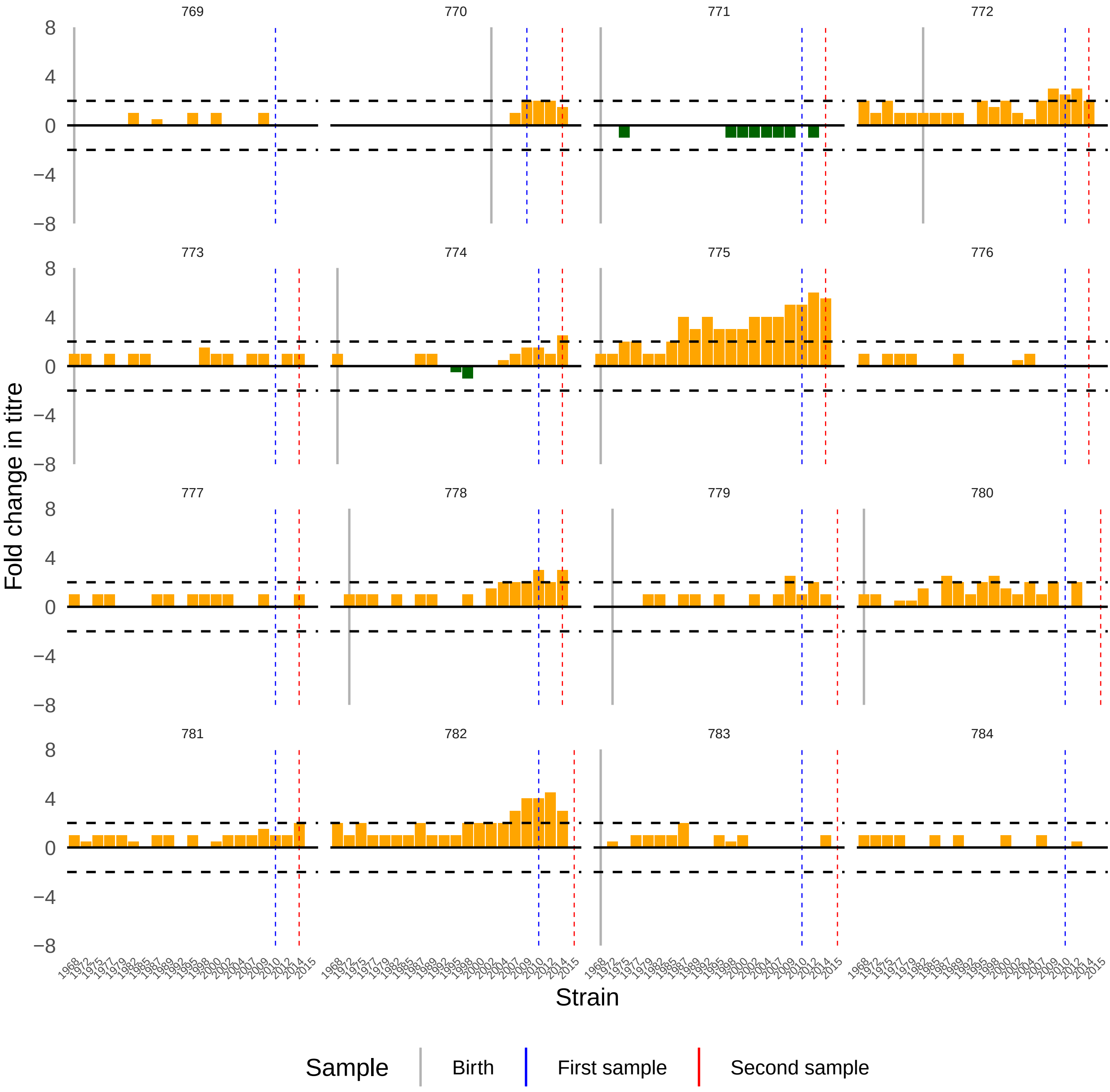

769
770
771
772
773
774
775
776
777
778
779
780
781
782
783
784
8
4
0
−4
−8
Fold change in titre
1968 1972 1975 1977 1979 1982 1985 1987 1989 1992 1995 1998 2000 2002 2004 2007 2009 2010 2012 2014 2015
Strain
Sample
Birth
First sample
Second sample

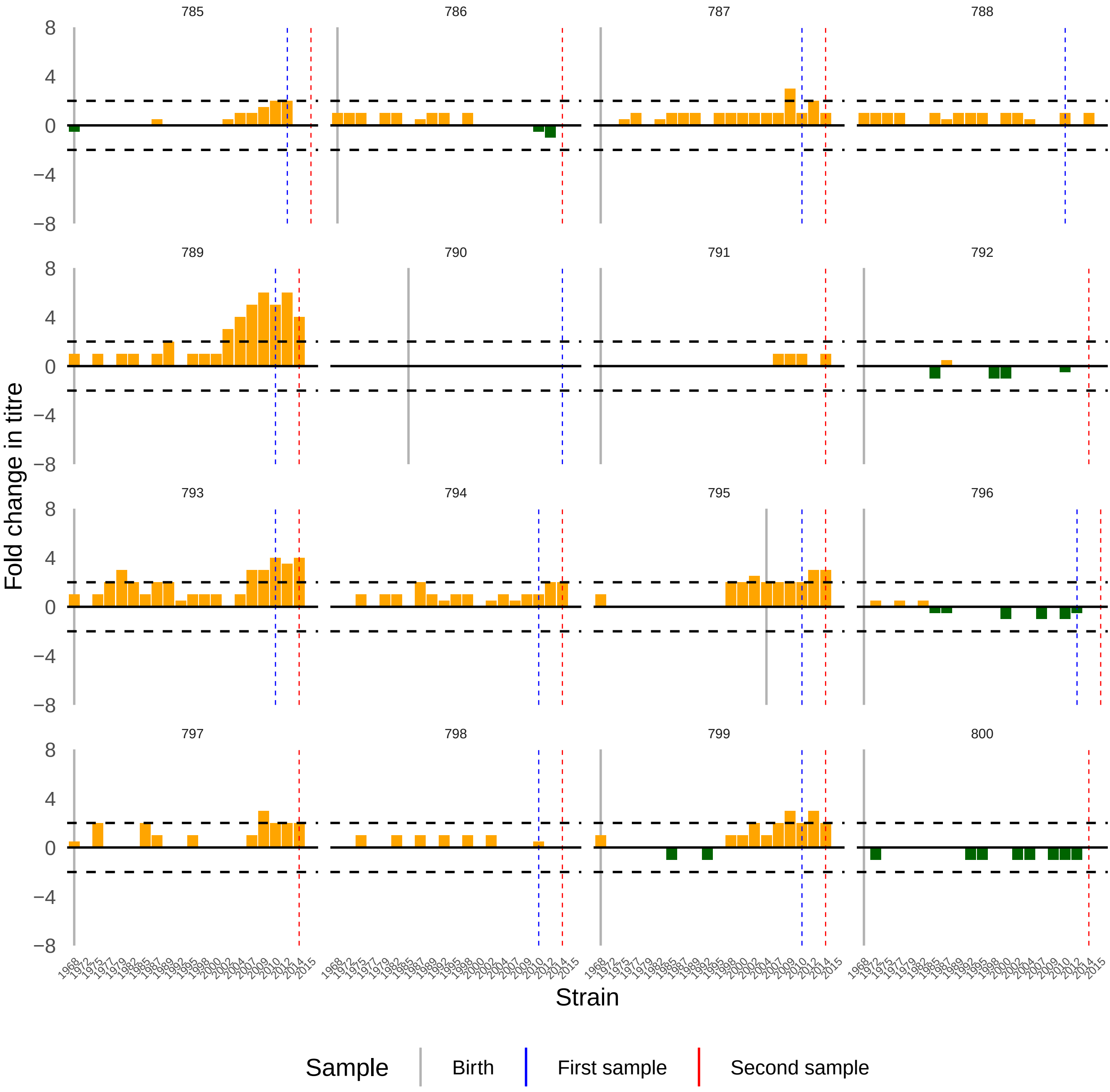

785
786
787
788
789
790
791
792
793
794
795
796
797
798
799
800
8
4
0
−4
−8
Fold change in titre
1968 1972 1975 1977 1979 1982 1985 1987 1989 1992 1995 1998 2000 2002 2004 2007 2009 2010 2012 2014 2015
Strain
Sample
Birth
First sample
Second sample

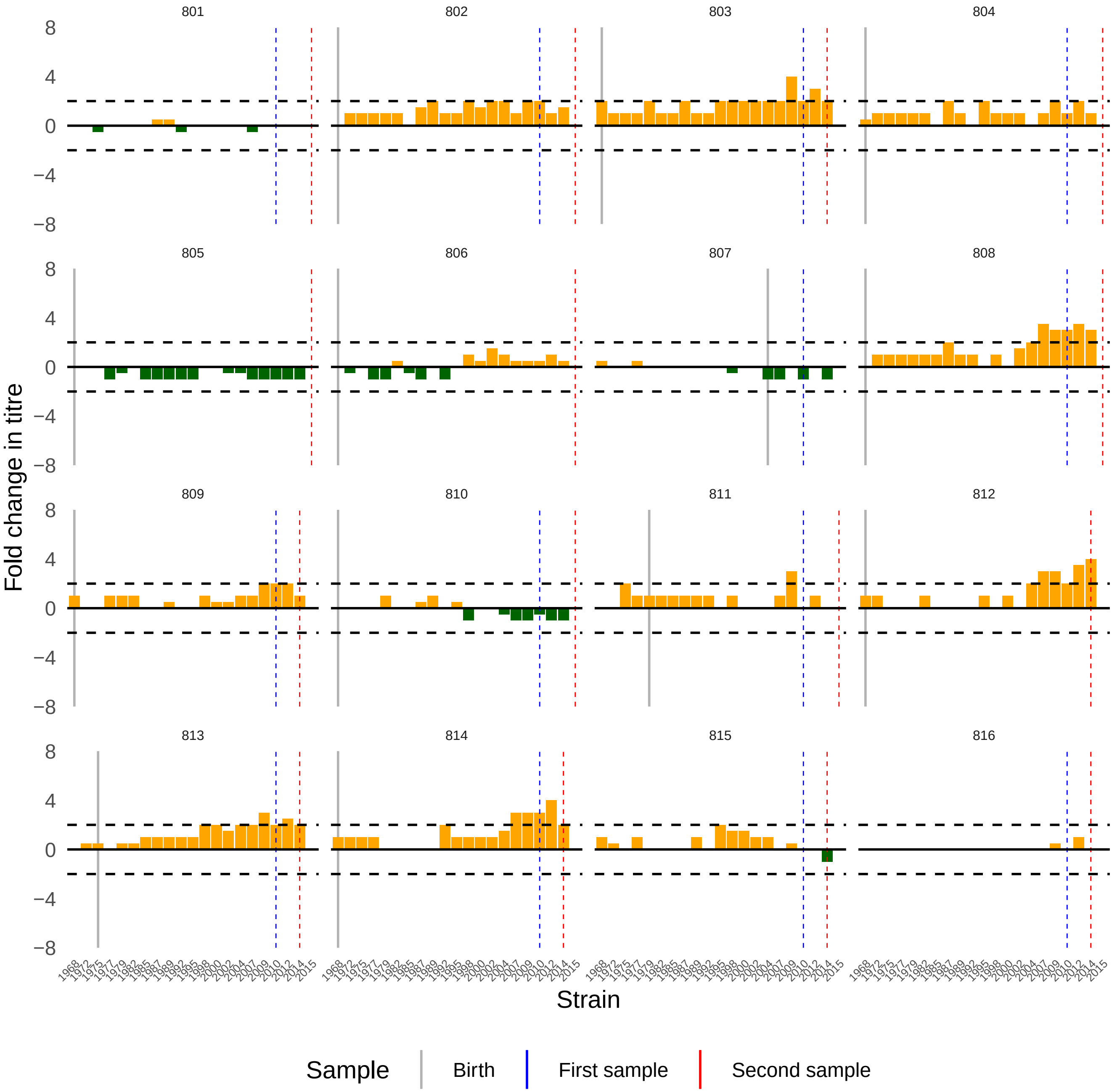

801
802
803
804
805
806
807
808
809
810
811
812
813
814
815
816
Fold change in titre
8
4
0
−4
−8
1968 1972 1975 1977 1979 1982 1985 1987 1989 1992 1995 1998 2000 2002 2004 2007 2009 2010 2012 2014 2015
Strain
Sample
Birth
First sample
Second sample

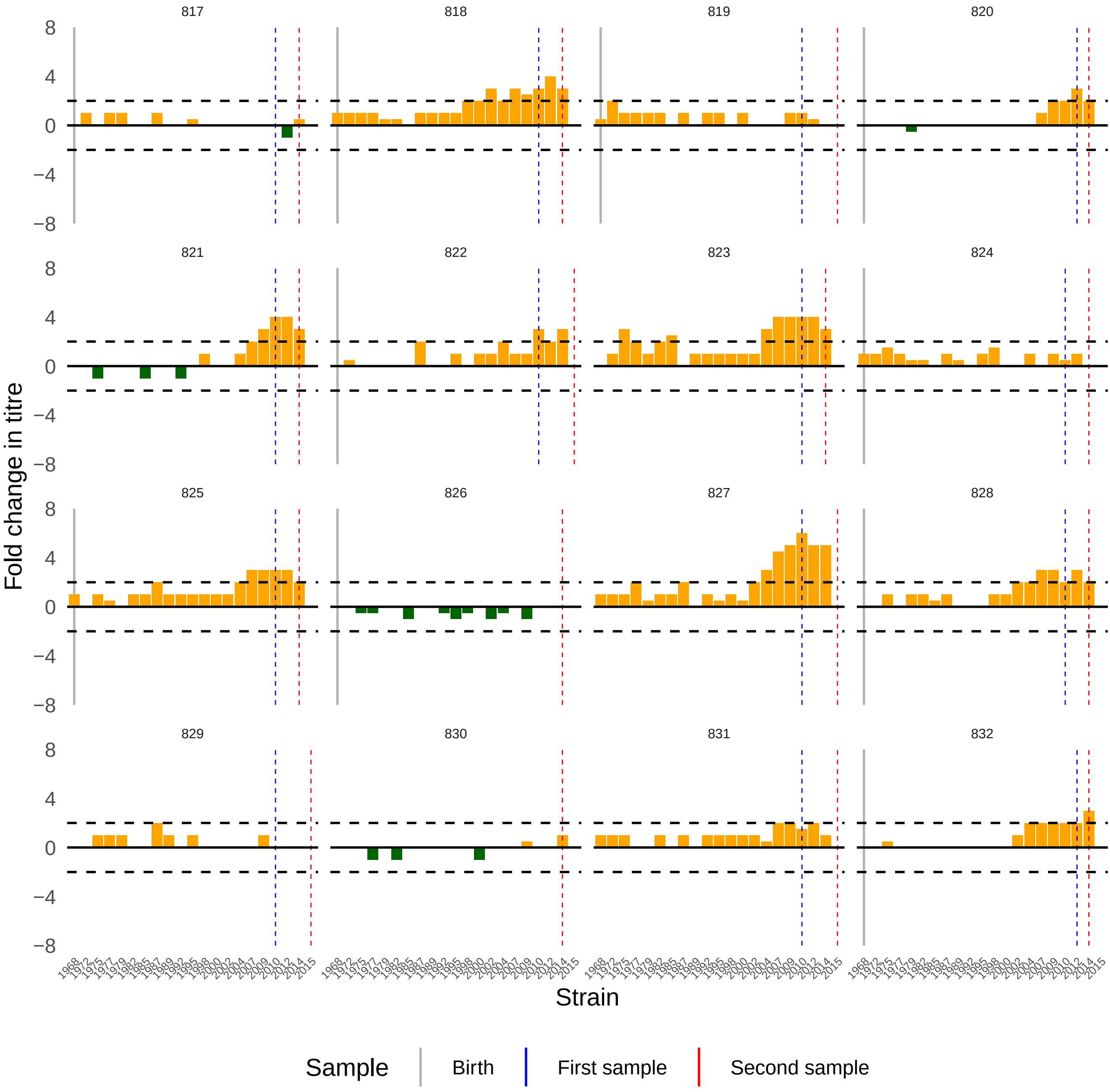

817
818
819
820
821
822
823
824
825
826
827
828
829
830
831
832
8
4
0
−4
−8
Fold change in titre
1968 1972 1975 1977 1979 1982 1985 1987 1989 1992 1995 1998 2000 2002 2004 2007 2009 2010 2012 2014 2015
Strain
Sample
Birth
First sample
Second sample

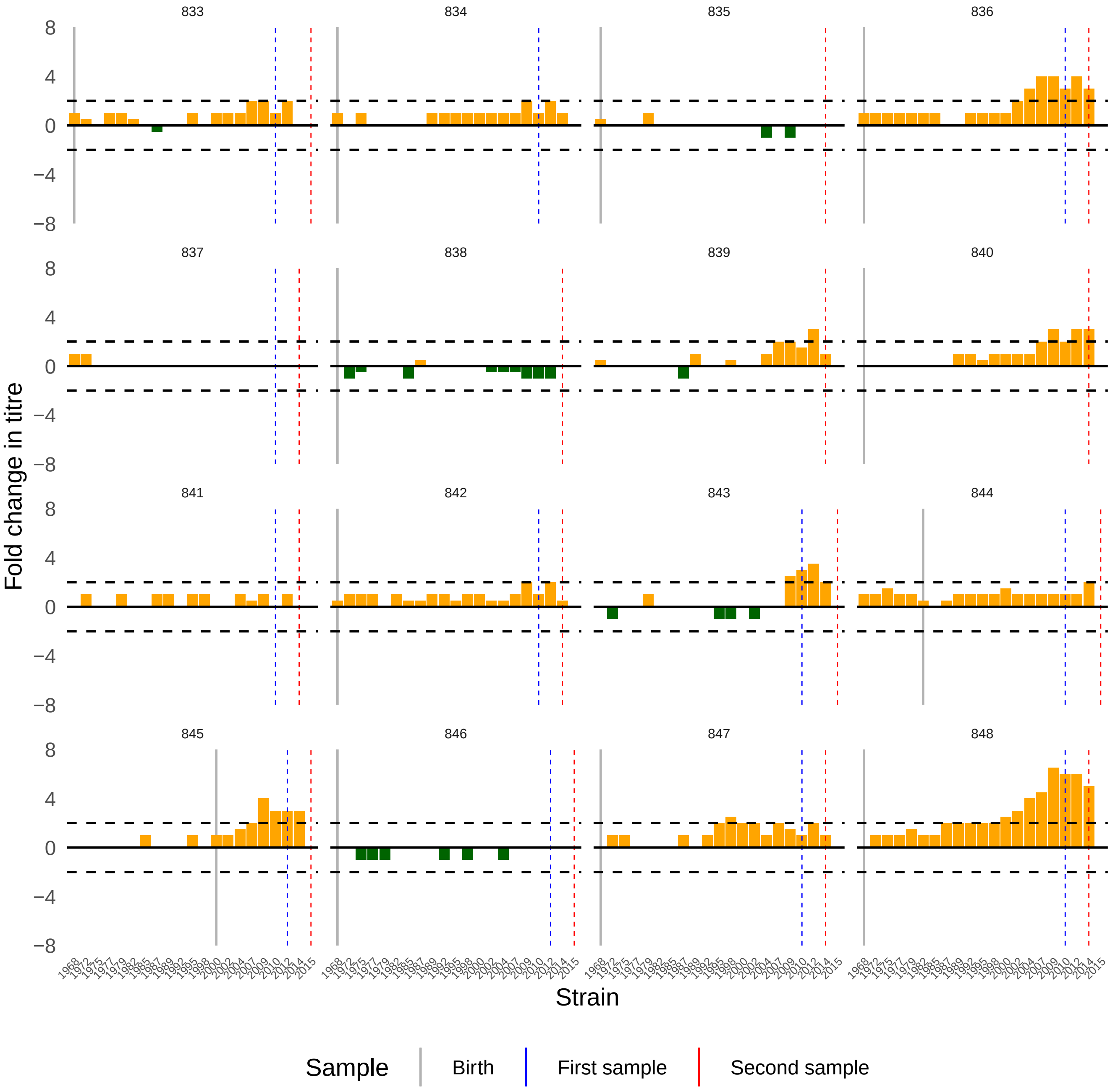

833
834
835
836
837
838
839
840
841
842
843
844
845
846
847
848
8
4
0
−4
−8
Fold change in titre
1968
1972
1975
1977
1979
1982
1985
1987
1989
1992
1995
1998
2000
2002
2004
2007
2009
2010
2012
2014
2015
Strain
Sample
Birth
First sample
Second sample

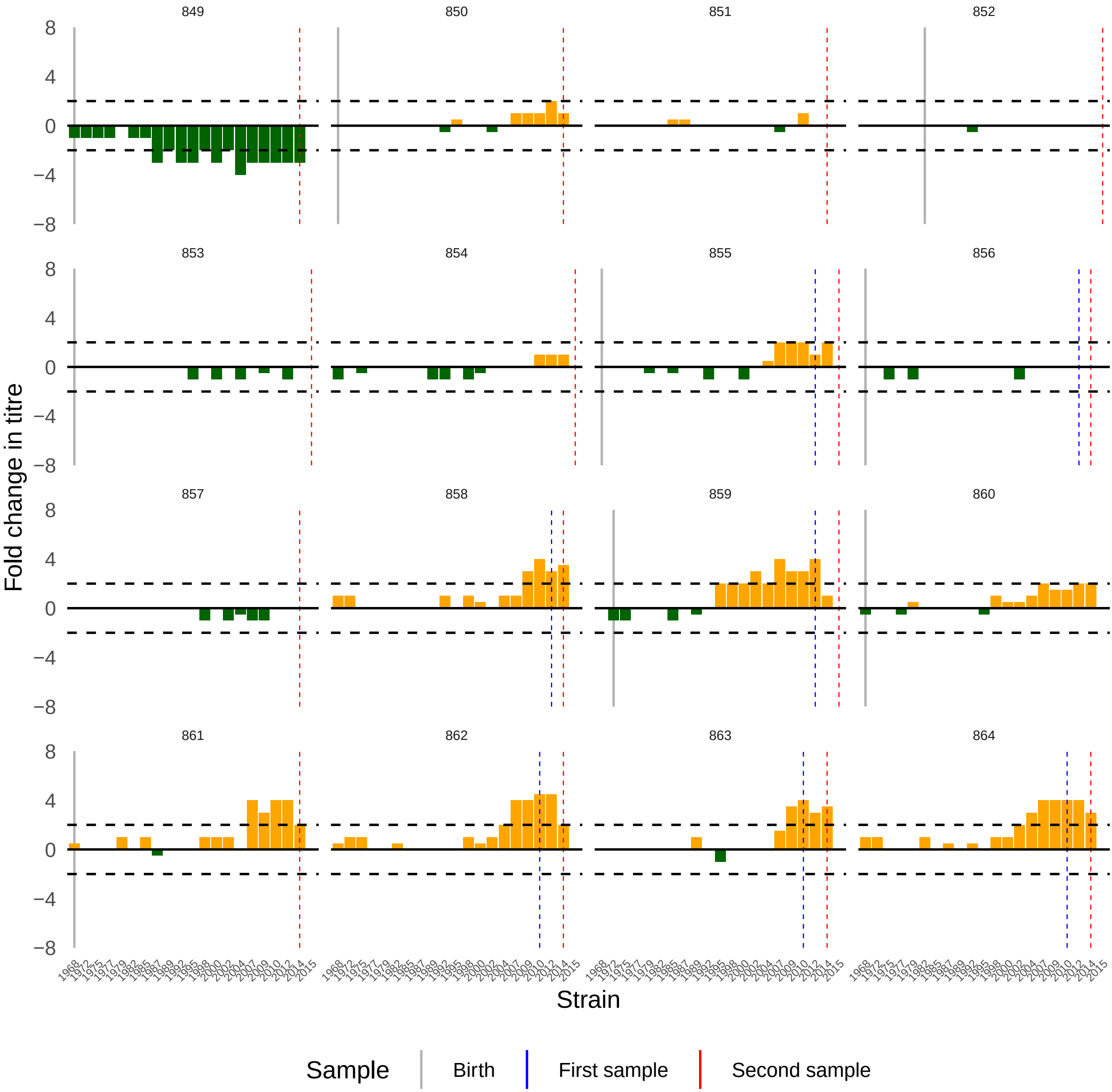
849
850
851
852
853
854
855
856
857
858
859
860
861
862
863
864
Fold change in titre
Strain
Sample
Birth
First sample
Second sample

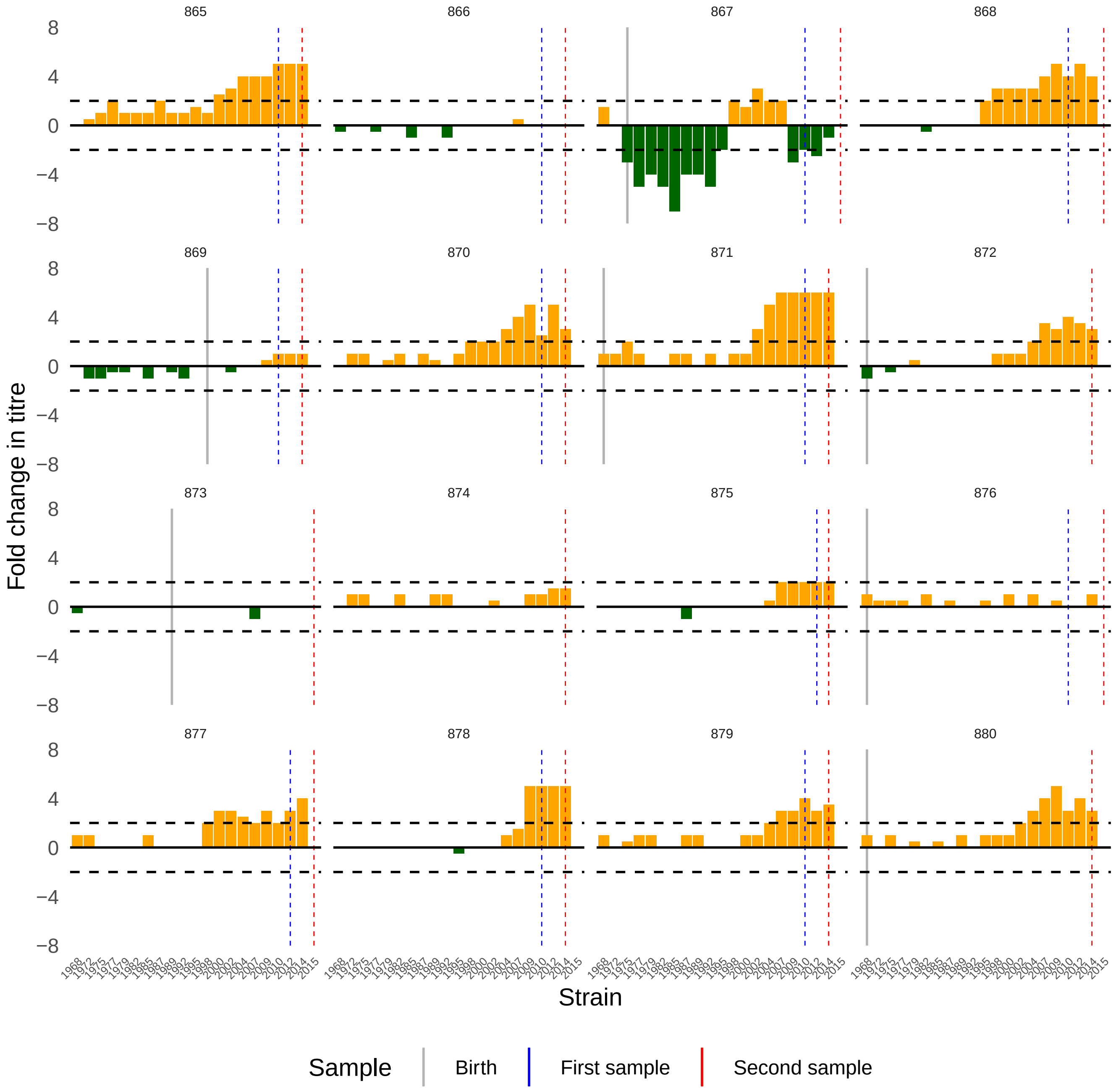

865
866
867
868
869
870
871
872
873
874
875
876
877
878
879
880
Fold change in titre
Strain
Sample
Birth
First sample
Second sample

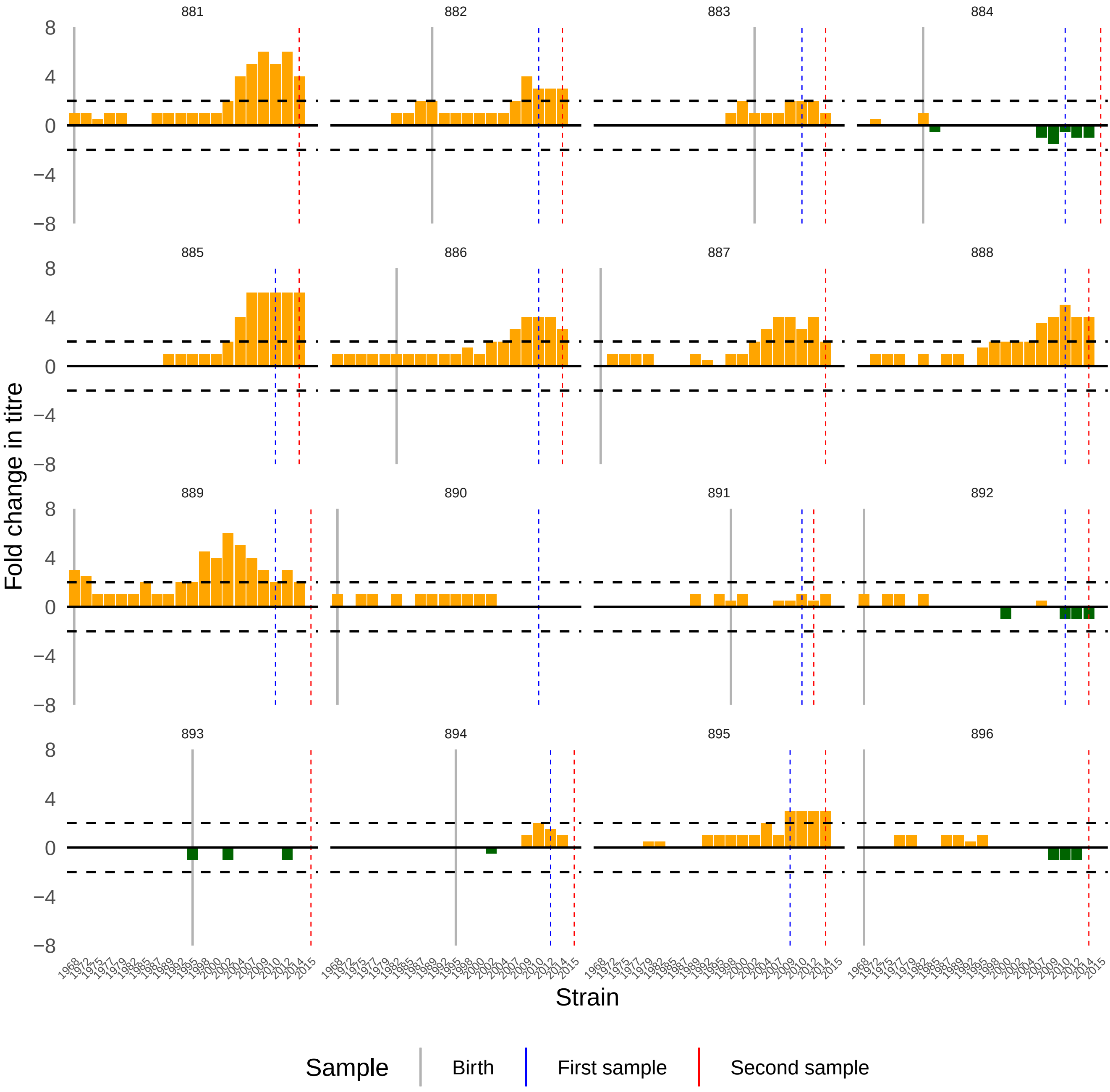

881
882
883
884
885
886
887
888
889
890
891
892
893
894
895
896
Fold change in titre
Strain
Sample
Birth
First sample
Second sample

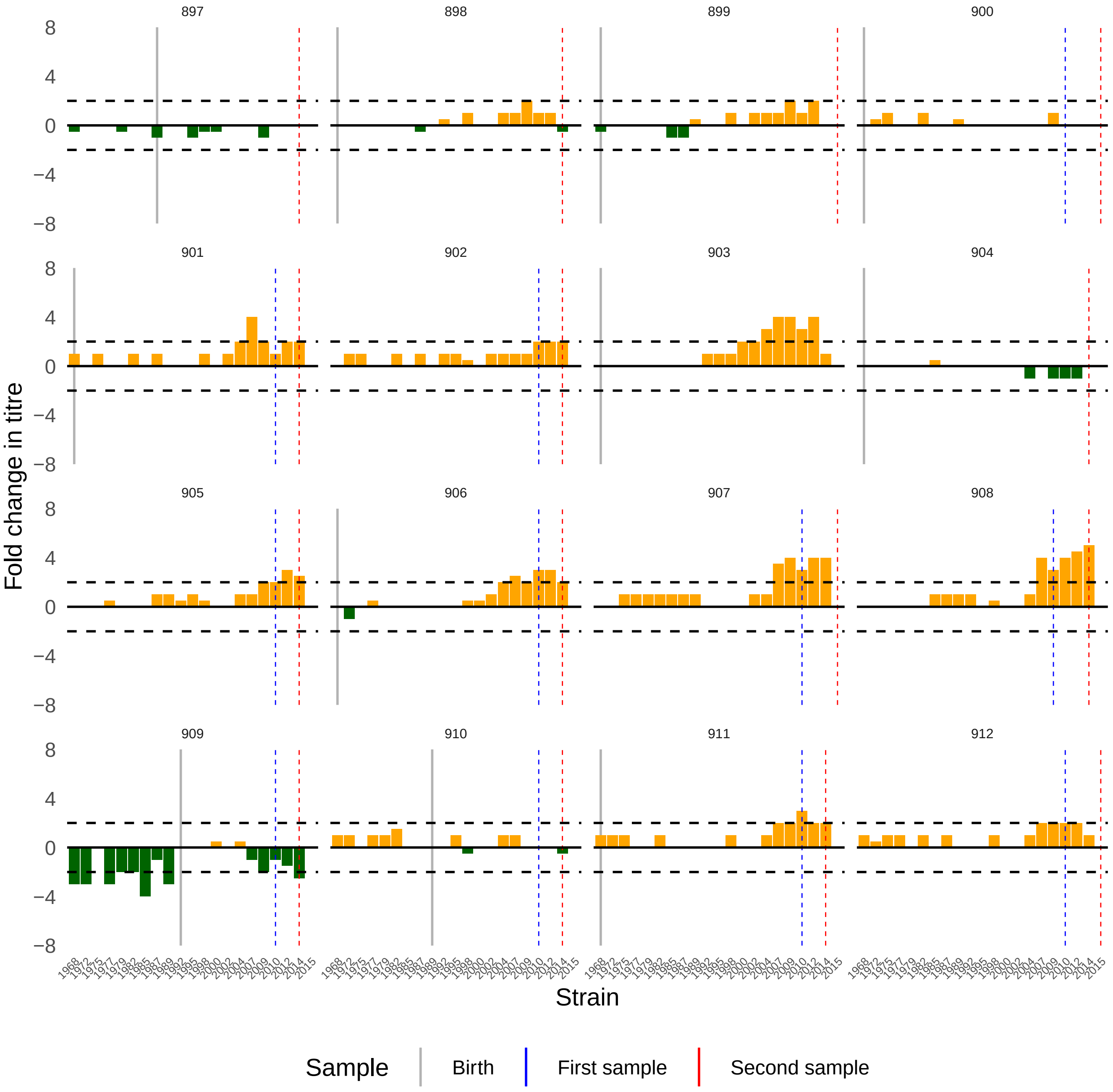

897
898
899
900
901
902
903
904
905
906
907
908
909
910
911
912
8
4
0
−4
−8
Fold change in titre
1968 1972 1975 1977 1979 1982 1985 1987 1989 1992 1995 1998 2000 2002 2004 2007 2009 2010 2012 2014 2015
Strain
Sample
Birth
First sample
Second sample

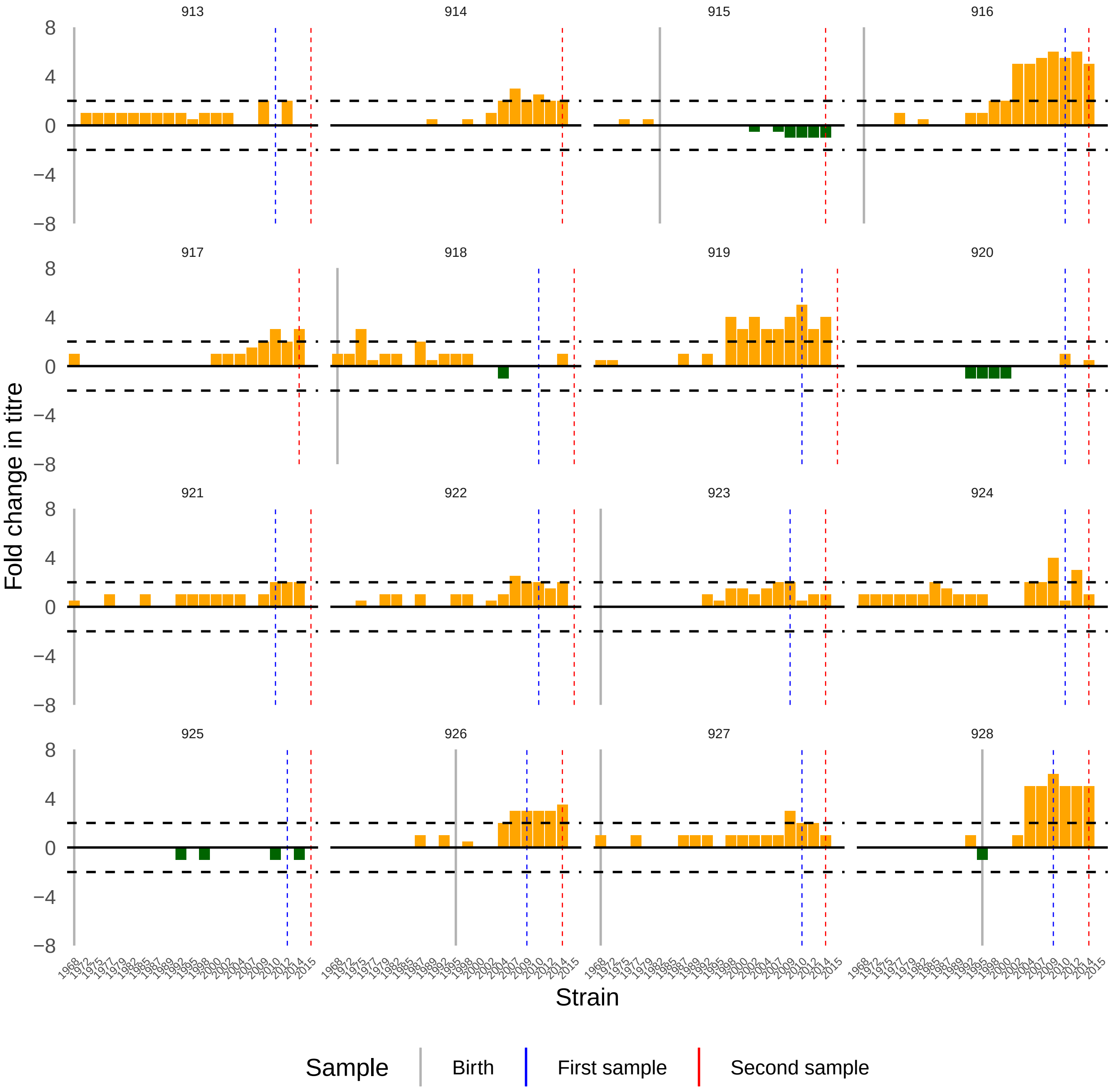
913
914
915
916
917
918
919
920
921
922
923
924
925
926
927
928
Fold change in titre
8
4
0
−4
−8
1968 1972 1975 1977 1979 1982 1985 1987 1989 1992 1995 1998 2000 2002 2004 2007 2009 2010 2012 2014 2015
Strain
Sample
Birth
First sample
Second sample

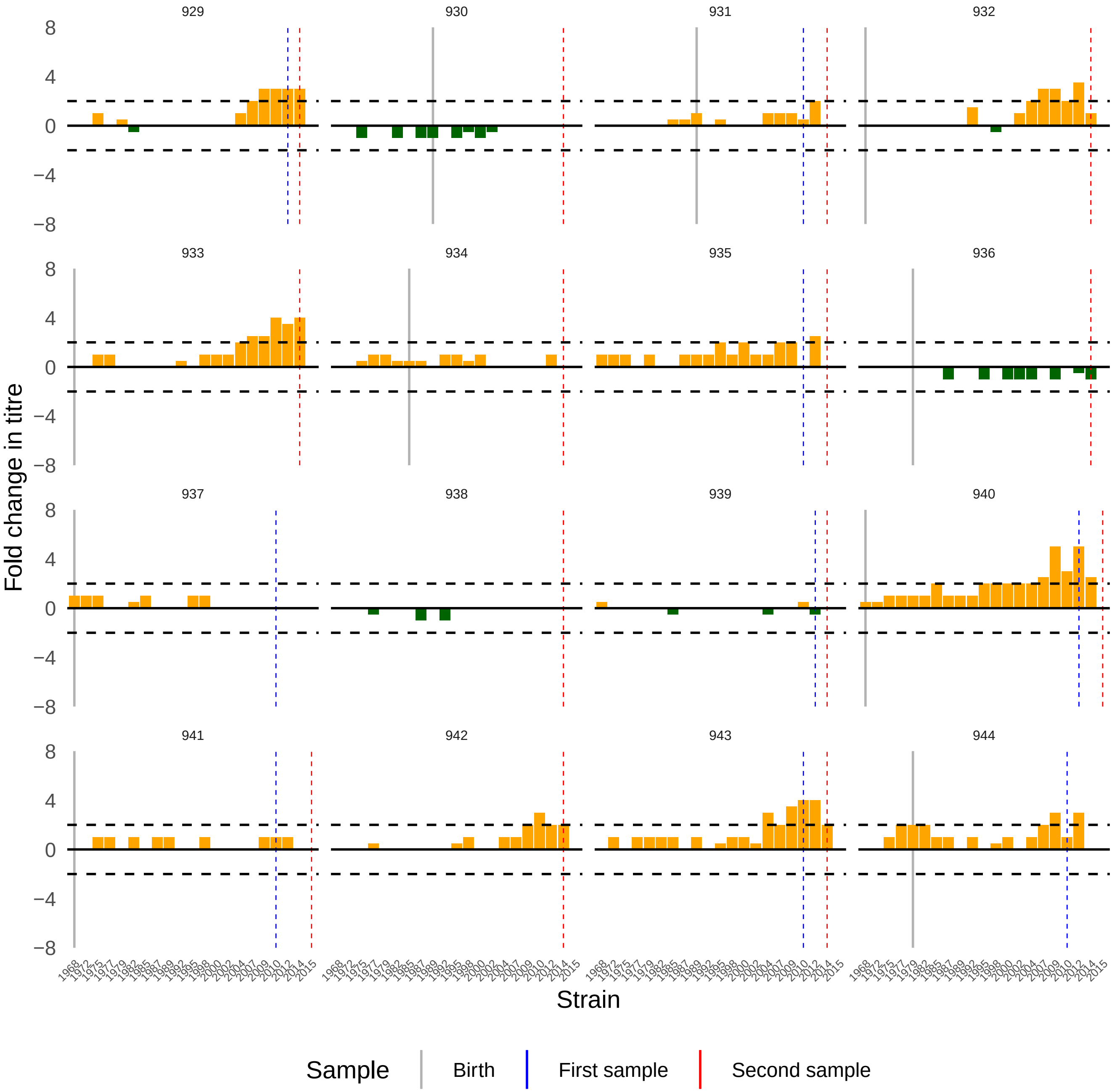

929
930
931
932
933
934
935
936
937
938
939
940
941
942
943
944
Fold change in titre
8
4
0
−4
−8
Strain
1968
1972
1975
1977
1979
1982
1985
1987
1989
1992
1995
1998
2000
2002
2004
2007
2009
2010
2012
2014
2015
Sample
Birth
First sample
Second sample

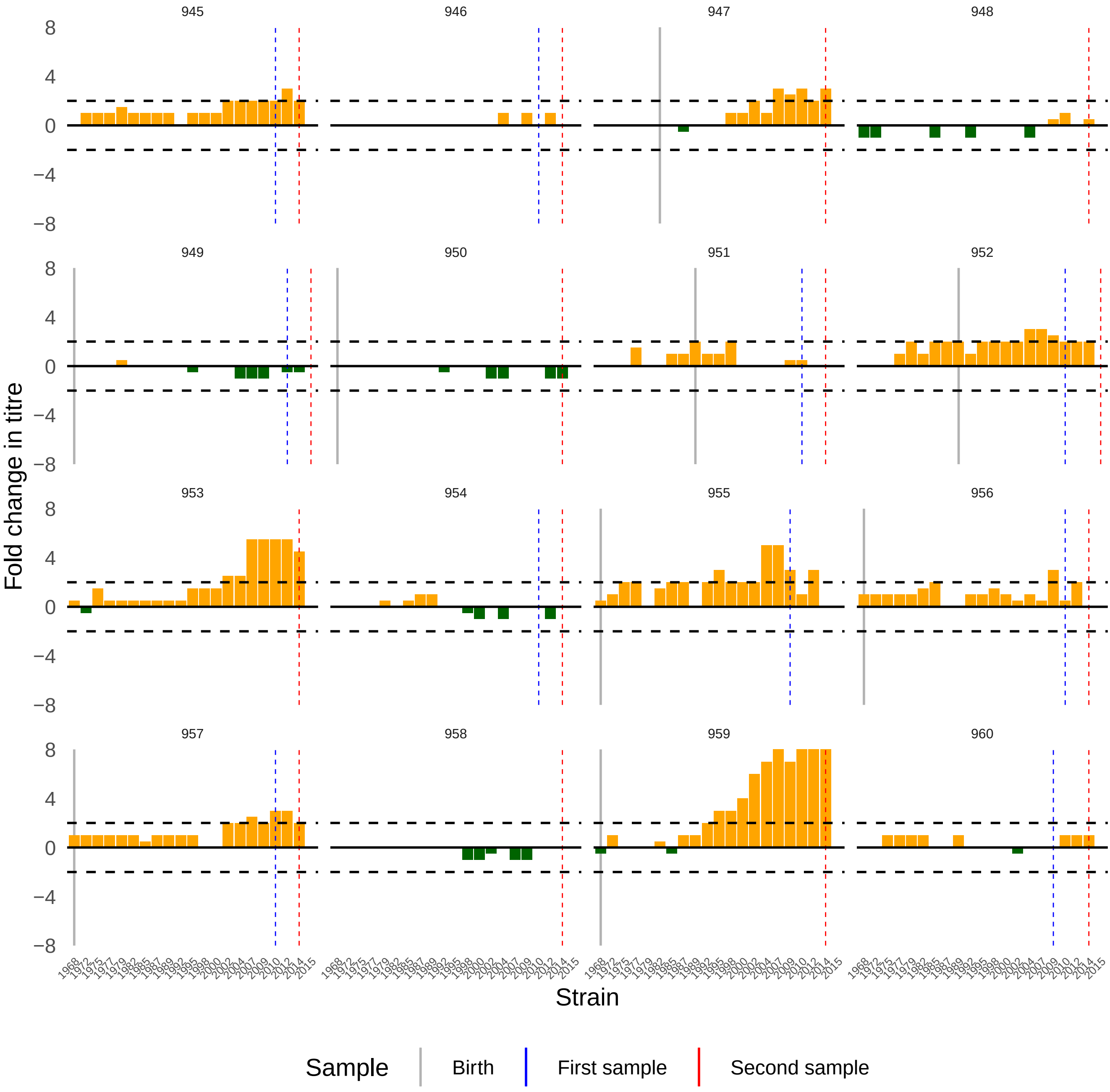

945
946
947
948
949
950
951
952
953
954
955
956
957
958
959
960
Fold change in titre
8
4
0
−4
−8
1968 1972 1975 1977 1979 1982 1985 1987 1989 1992 1995 1998 2000 2002 2004 2007 2009 2010 2012 2014 2015
Strain
Sample
Birth
First sample
Second sample

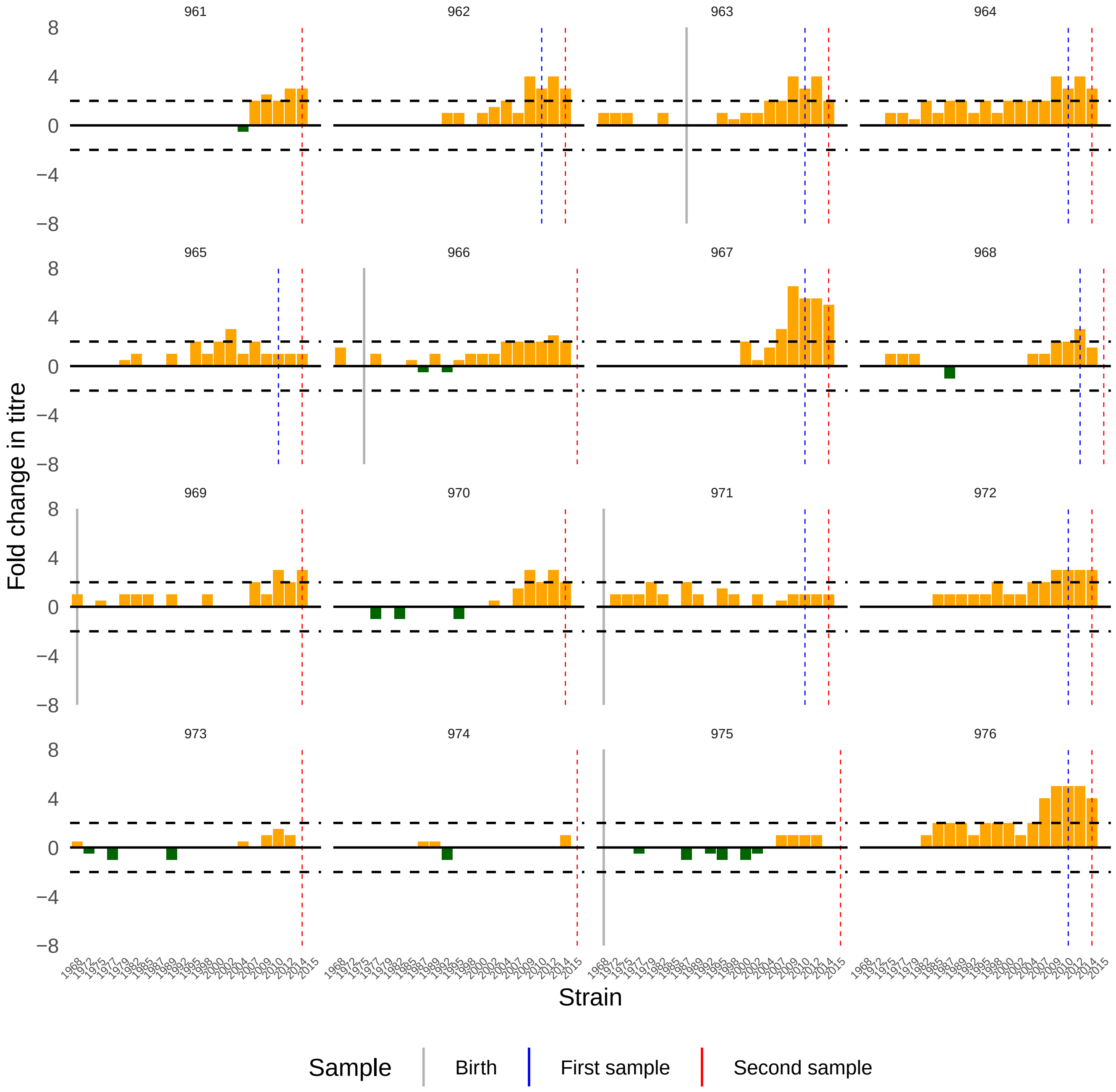

961
962
963
964
965
966
967
968
969
970
971
972
973
974
975
976
Fold change in titre
Strain
Sample
Birth
First sample
Second sample

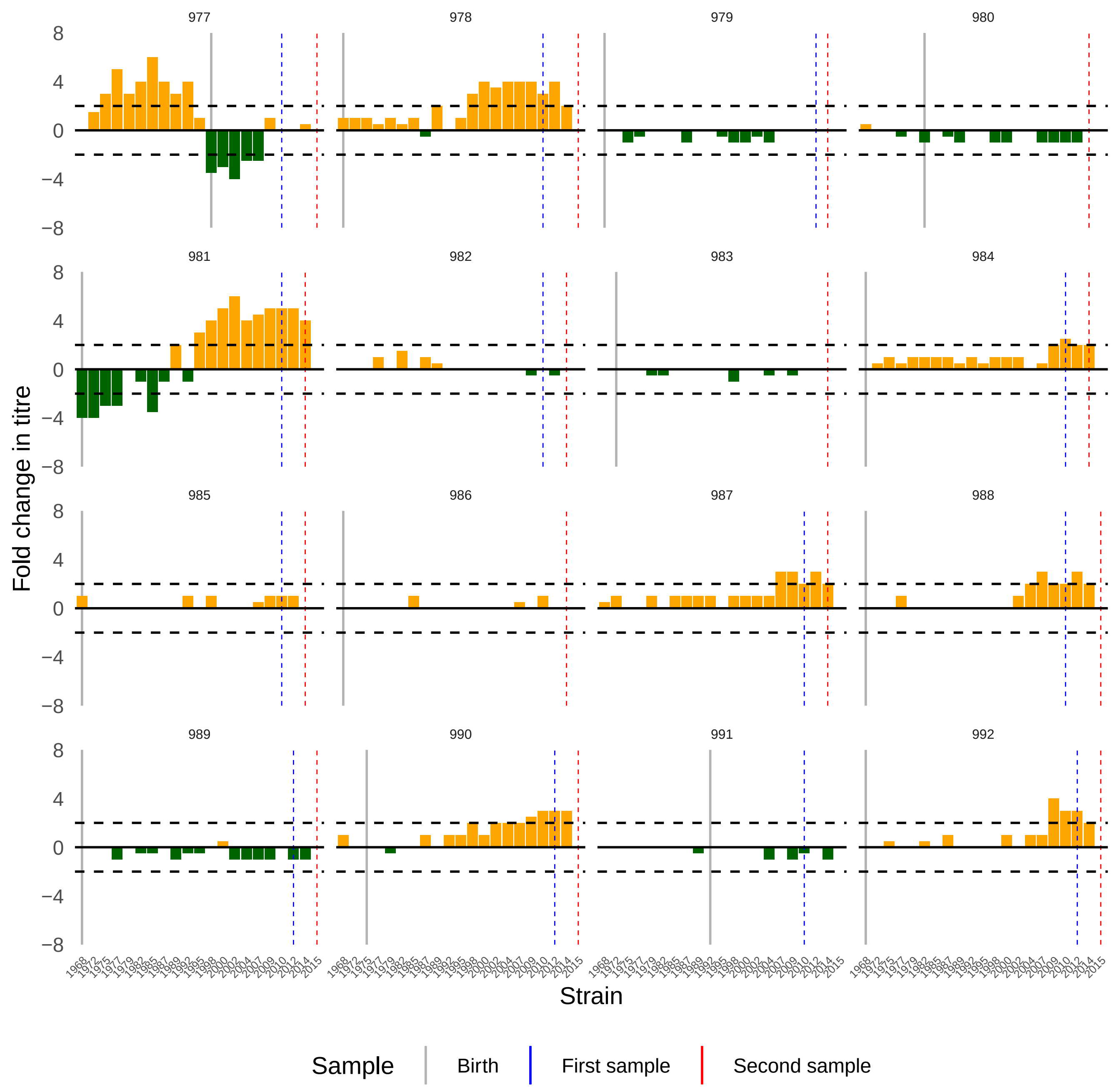
977
978
979
980
981
982
983
984
985
986
987
988
989
990
991
992
8
4
0
−4
−8
Fold change in titre
1968
1972
1975
1977
1979
1982
1985
1987
1989
1992
1995
1998
2000
2002
2004
2007
2009
2010
2012
2014
2015
Strain
Sample
Birth
First sample
Second sample

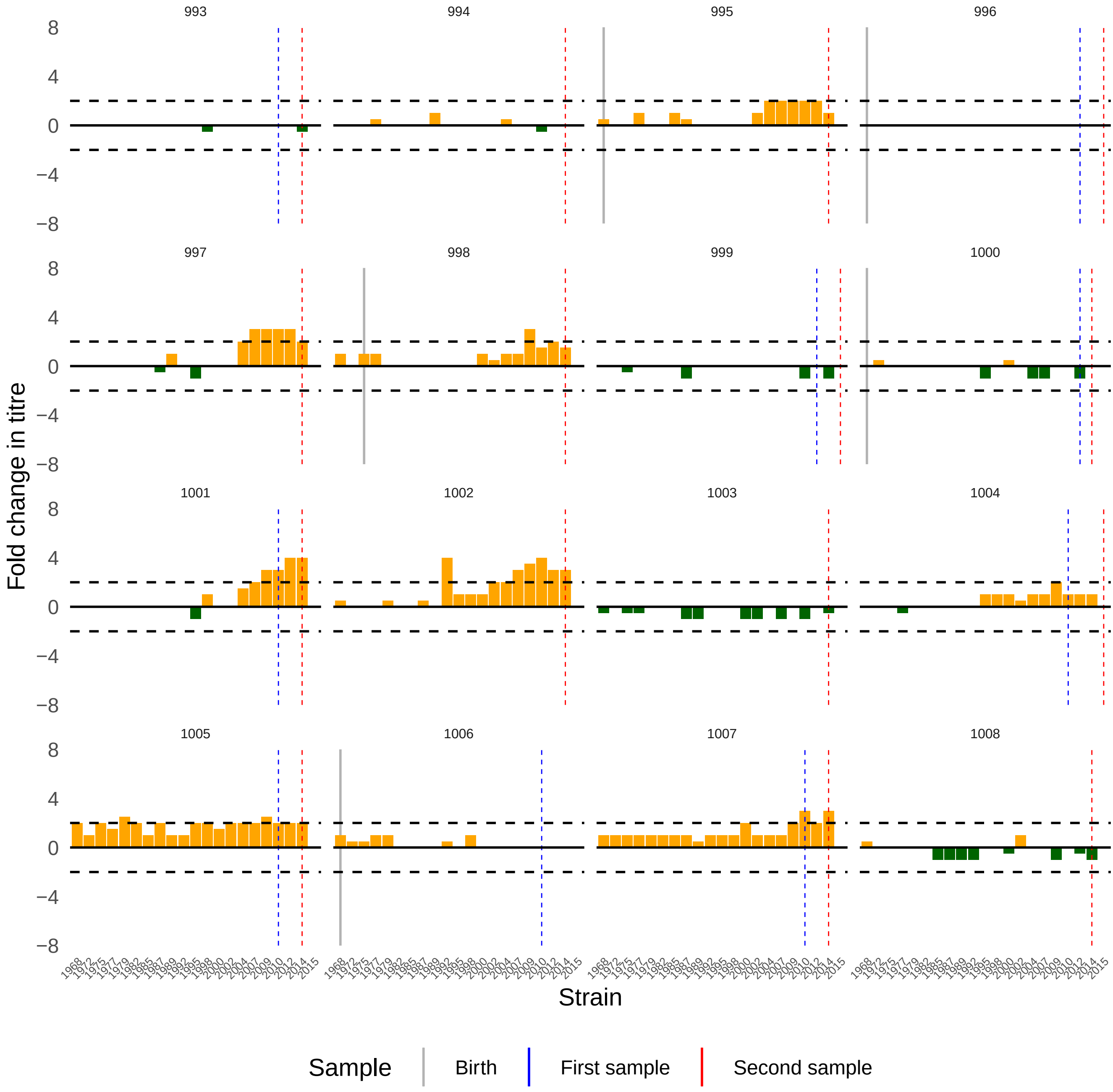

993
994
995
996
997
998
999
1000
1001
1002
1003
1004
1005
1006
1007
1008
Fold change in titre
Strain
Sample
Birth
First sample
Second sample

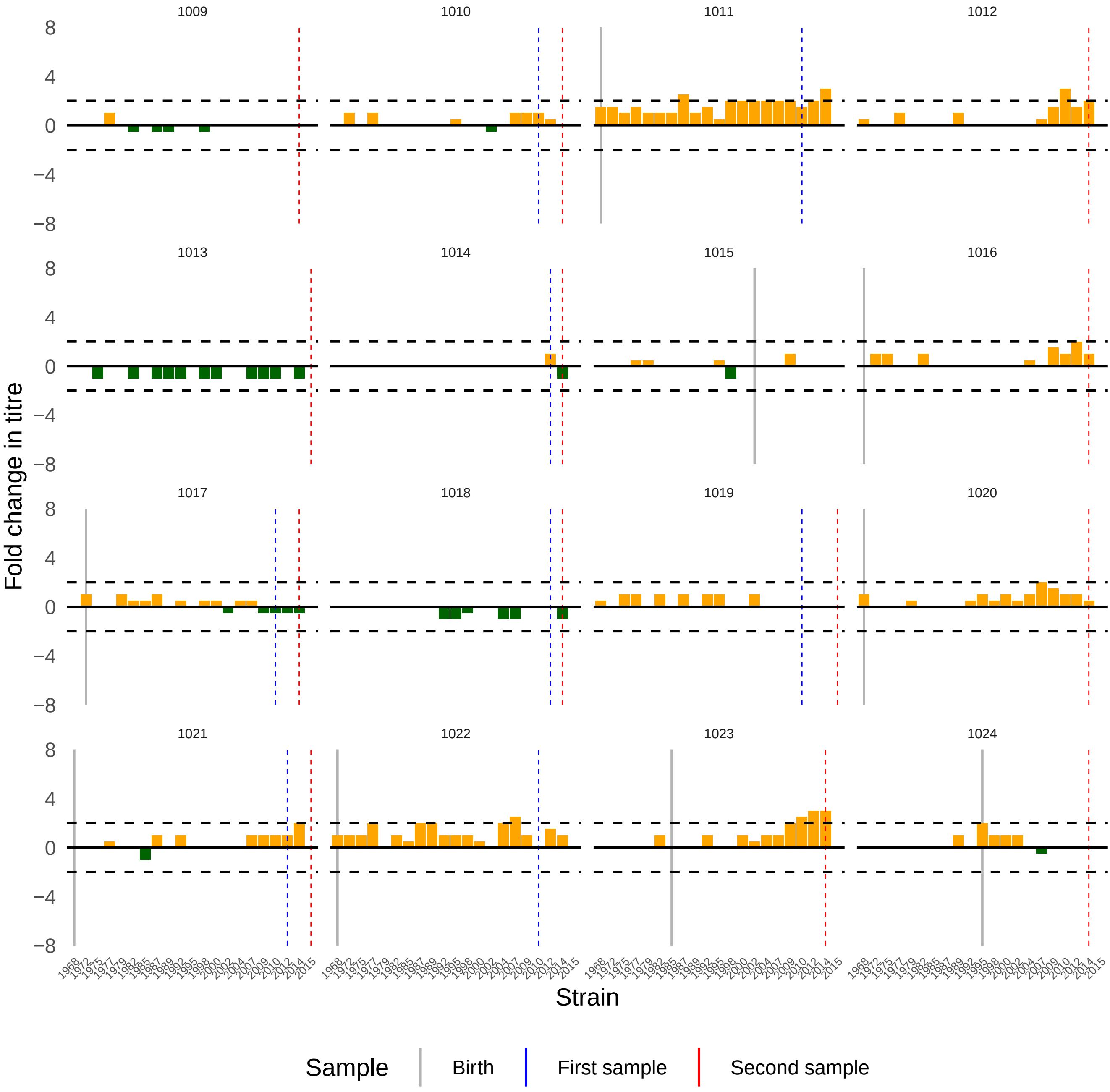

1009
1010
1011
1012
1013
1014
1015
1016
1017
1018
1019
1020
1021
1022
1023
1024
8
4
0
−4
−8
Fold change in titre
1968 1972 1975 1977 1979 1982 1985 1987 1989 1992 1995 1998 2000 2002 2004 2007 2009 2010 2012 2014 2015
Strain
Sample
Birth
First sample
Second sample

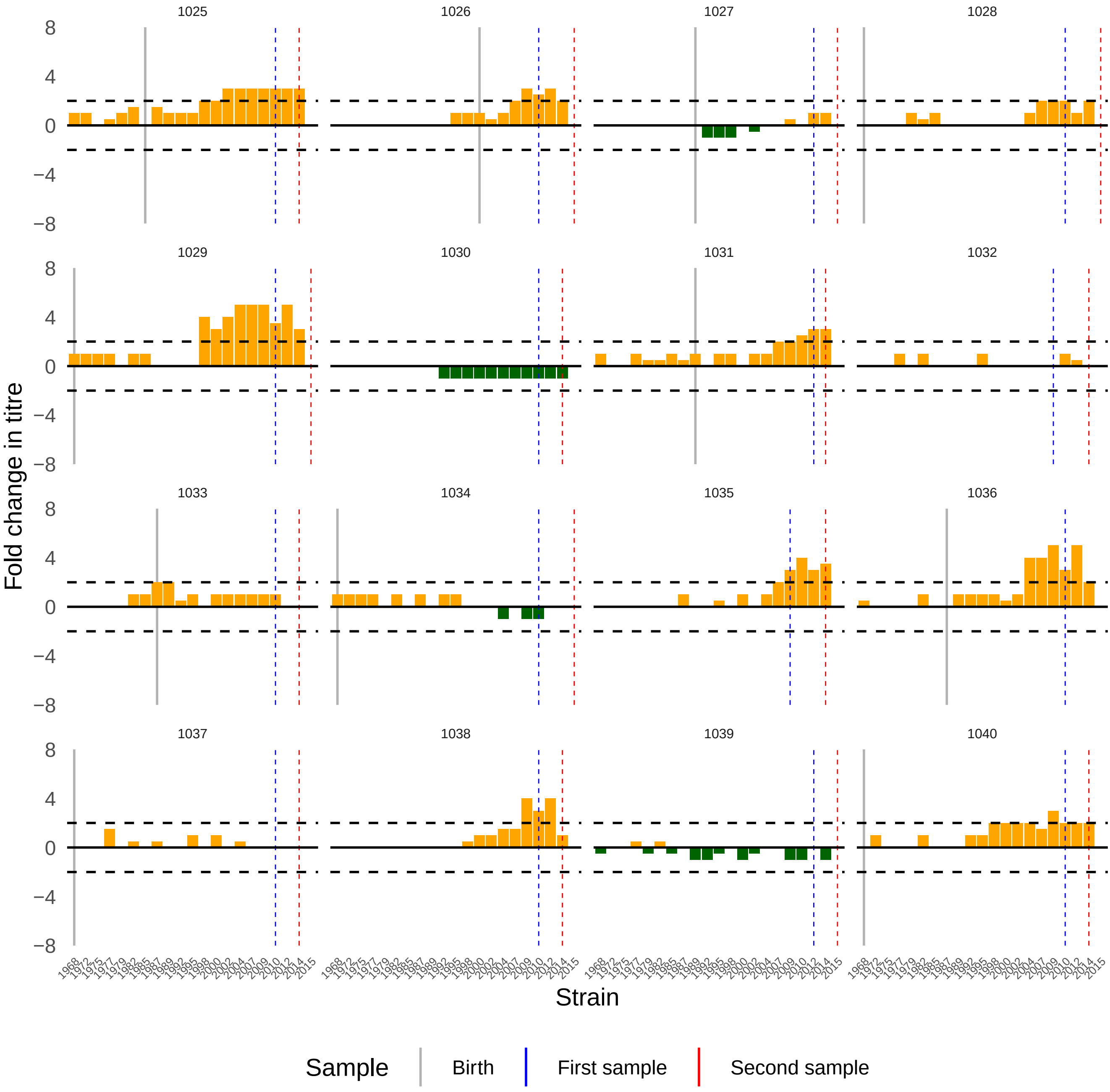

1025
1026
1027
1028
1029
1030
1031
1032
1033
1034
1035
1036
1037
1038
1039
1040
Fold change in titre
8
4
0
−4
−8
Strain
Sample
Birth
First sample
Second sample

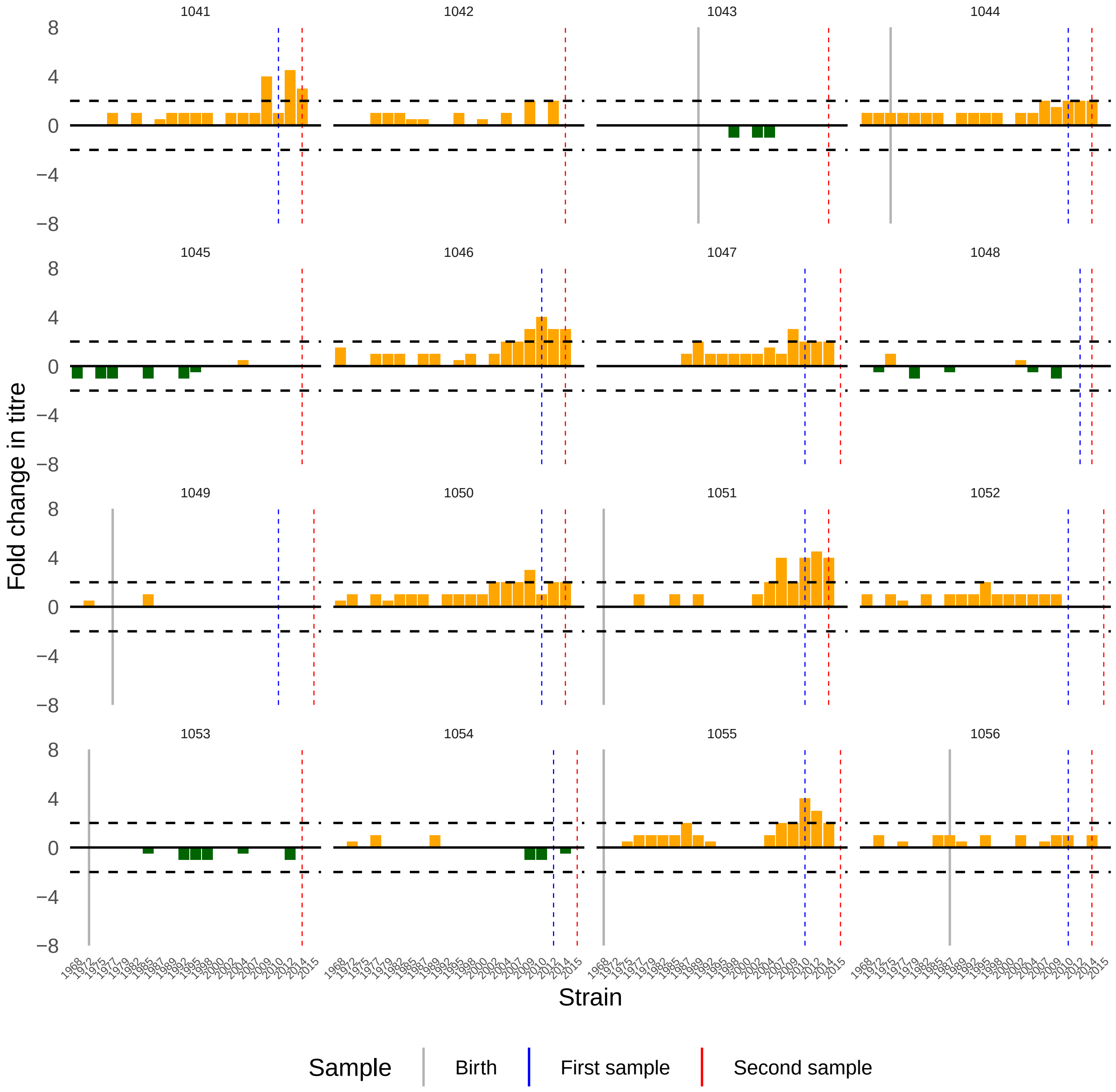

1041
1042
1043
1044
1045
1046
1047
1048
1049
1050
1051
1052
1053
1054
1055
1056
Fold change in titre
Strain
Sample
Birth
First sample
Second sample

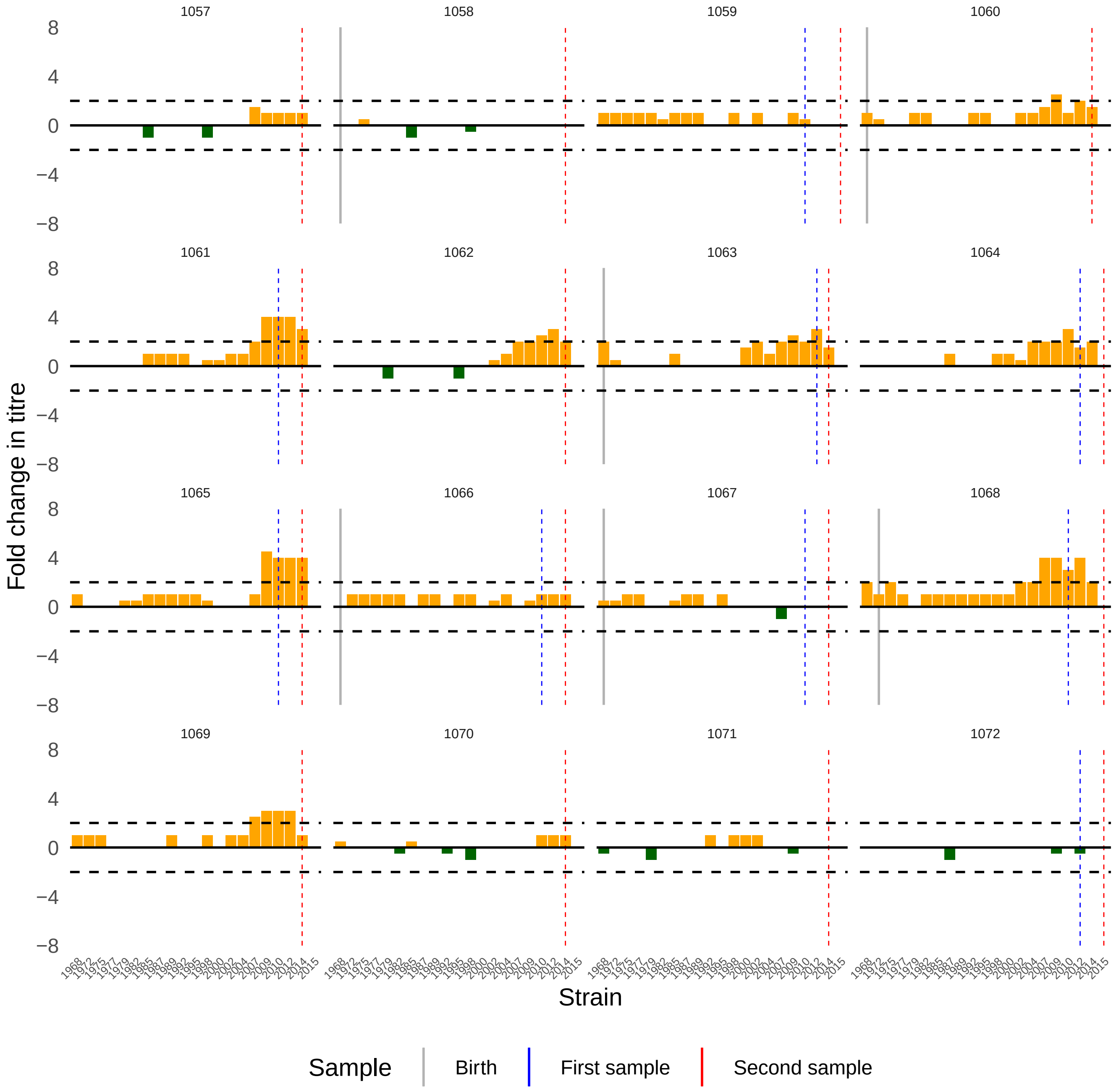
1057
1058
1059
1060
1061
1062
1063
1064
1065
1066
1067
1068
1069
1070
1071
1072
Fold change in titre
Strain
Sample
Birth
First sample
Second sample

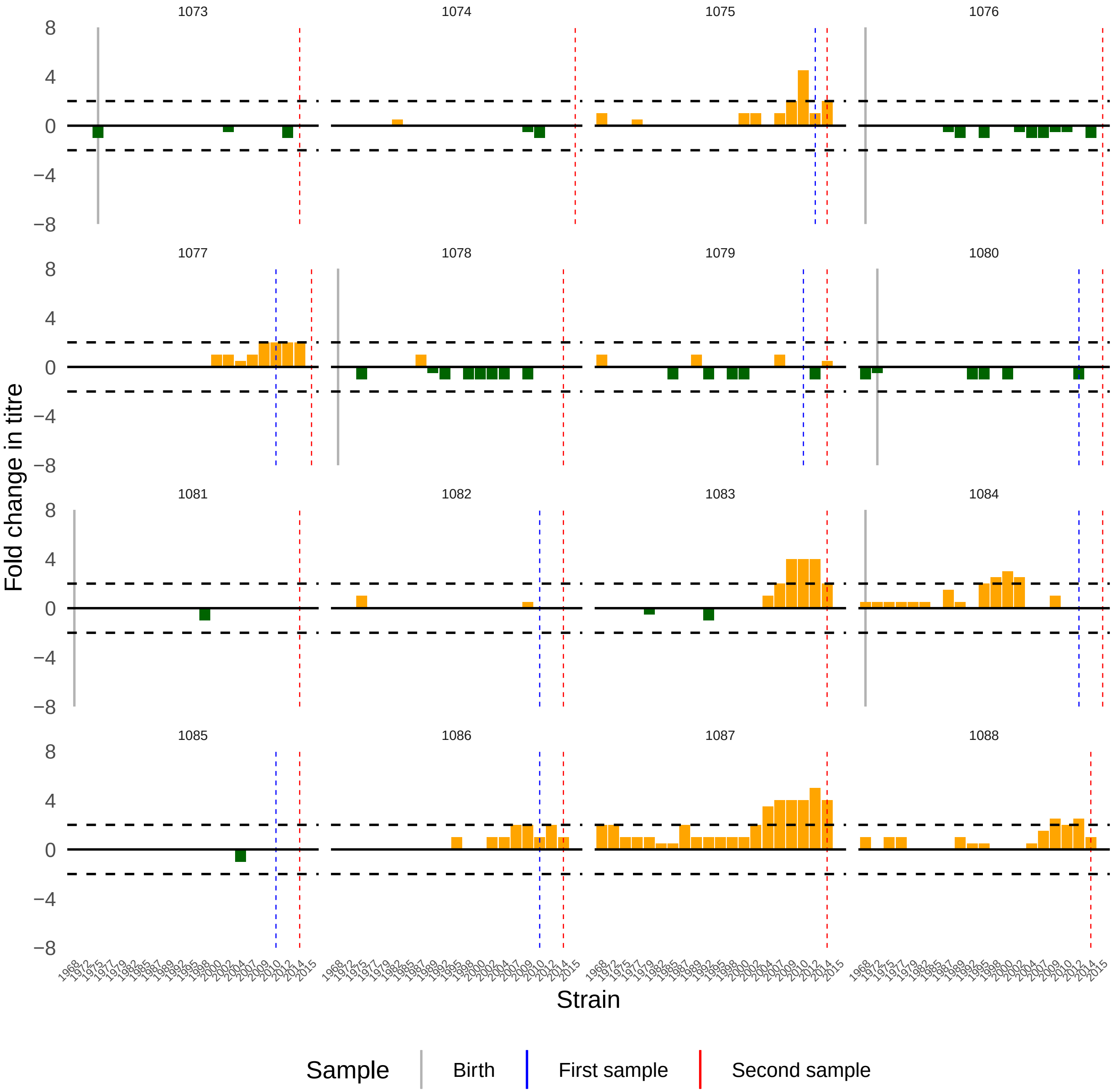

1073
1074
1075
1076
1077
1078
1079
1080
1081
1082
1083
1084
1085
1086
1087
1088
8
4
0
−4
−8
Fold change in titre
1968 1972 1975 1977 1979 1982 1985 1987 1989 1992 1995 1998 2000 2002 2004 2007 2009 2010 2012 2014 2015
Strain
Sample
Birth
First sample
Second sample

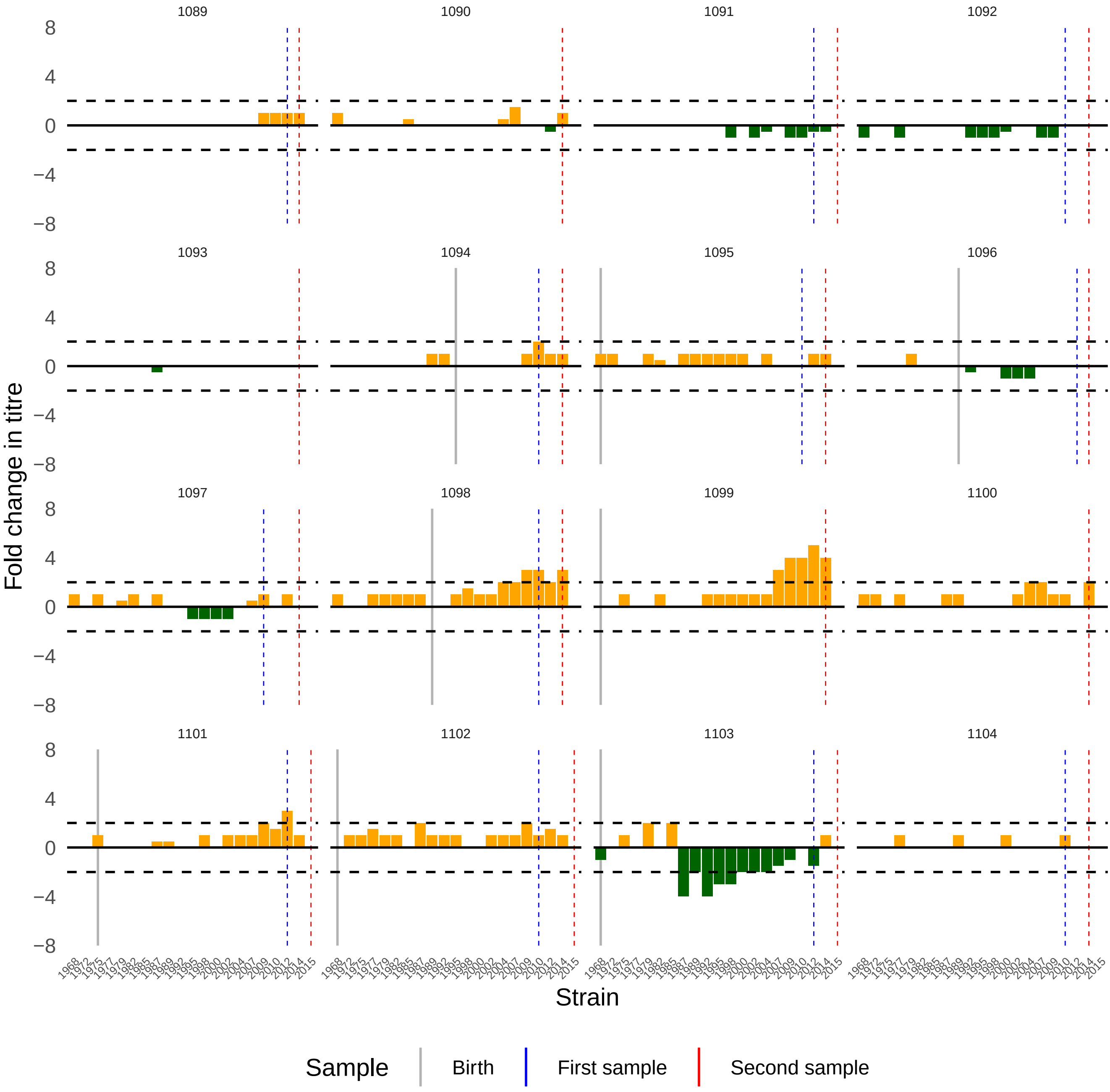
1089
1090
1091
1092
1093
1094
1095
1096
1097
1098
1099
1100
1101
1102
1103
1104
Fold change in titre
8
4
0
−4
−8
1968 1972 1975 1977 1979 1982 1985 1987 1989 1992 1995 1998 2000 2002 2004 2007 2009 2010 2012 2014 2015
Strain
Sample
Birth
First sample
Second sample

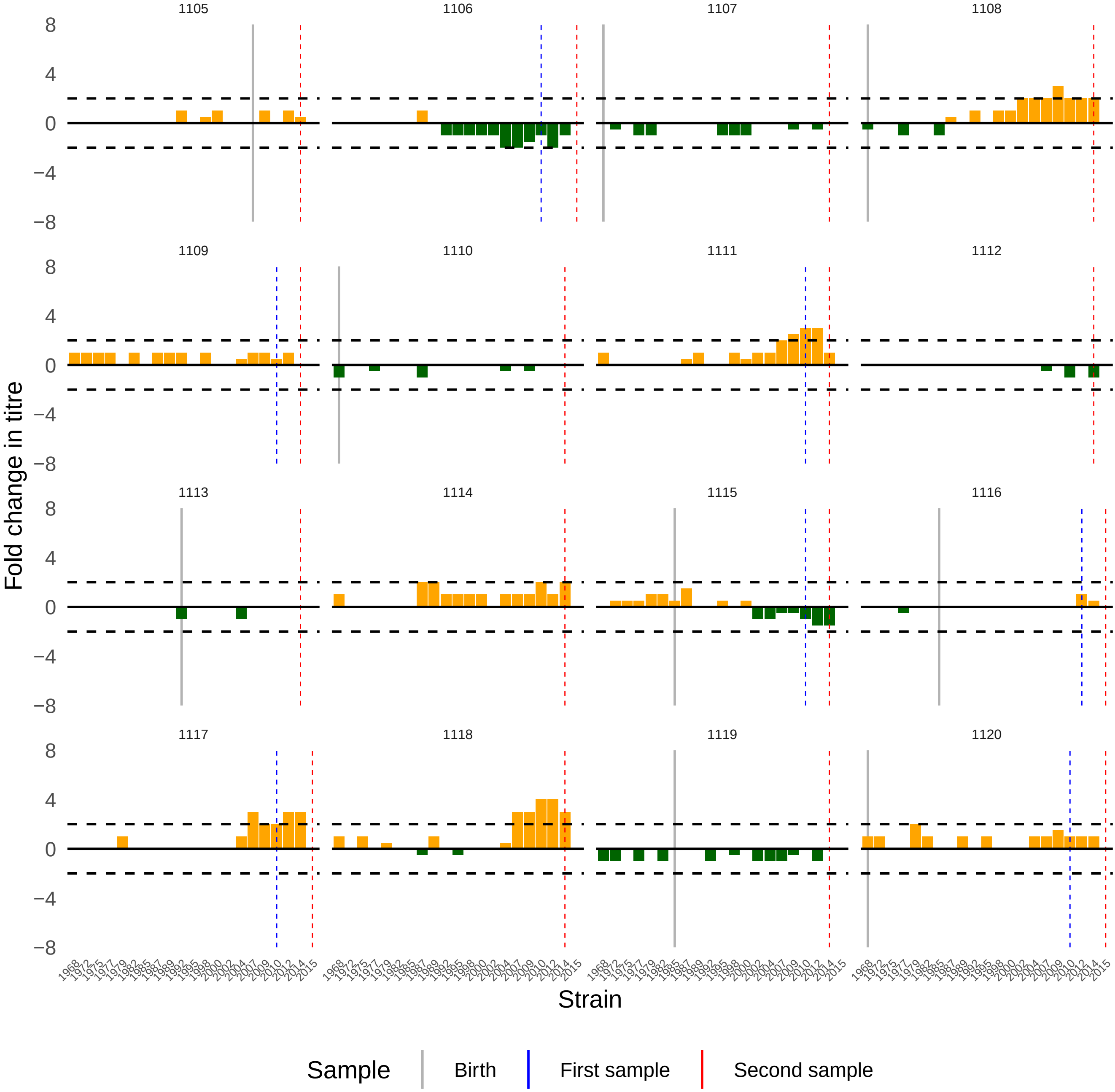

1105
1106
1107
1108
1109
1110
1111
1112
1113
1114
1115
1116
1117
1118
1119
1120
Fold change in titre
8
4
0
−4
−8
Strain
1968 1972 1975 1977 1979 1982 1985 1987 1989 1992 1995 1998 2000 2002 2004 2007 2009 2010 2012 2014 2015
Sample
Birth
First sample
Second sample

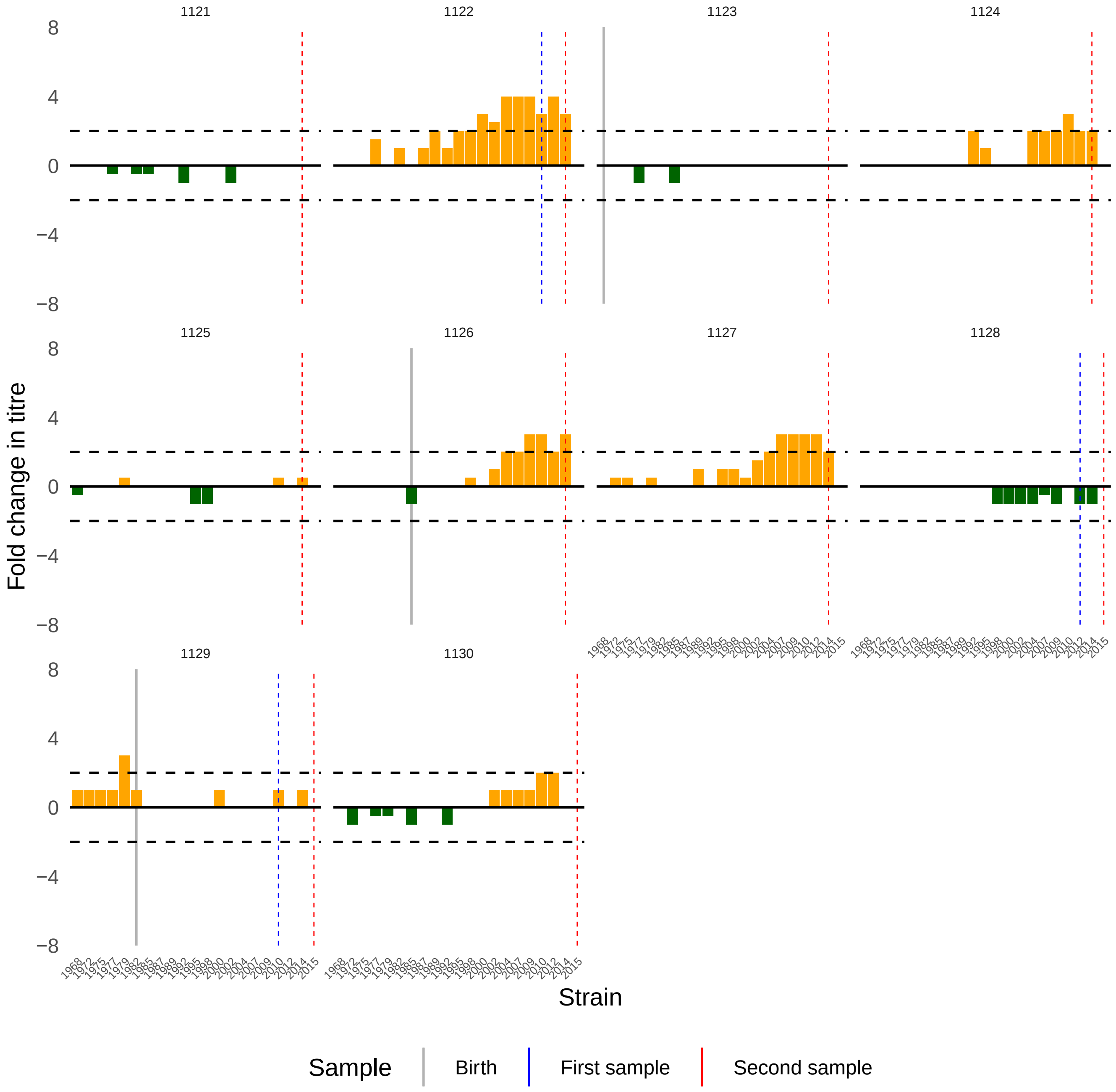
1121
1122
1123
1124
1125
1126
1127
1128
1129
1130
8
4
0
−4
−8
Fold change in titre
Strain
1968 1972 1975 1977 1979 1982 1985 1987 1989 1992 1995 1998 2000 2002 2004 2007 2009 2010 2012 2014 2015
Sample
Birth
First sample
Second sample
