# Supplementary material for: Reconstructed influenza A/H3N2 infection histories reveal variation in incidence and antibody dynamics over the life course
Source: medRxiv. 2024 Mar 18:2024.03.18.24304371. Preprint. [Version 1] doi: 10.1101/2024.03.18.24304371 (PMC10984066; doi:10.1101/2024.03.18.24304371)
Supplement: Supplementary file 1 [file NIHPP2024.03.18.24304371V1-supplement-1.pdf]

medRxiv preprint doi: https://doi.org/10.1101/2024.03.18.24304371; this version posted March 18, 2024. The copyright holder for this preprint (which was not certified by peer review) is the author/funder, who has granted medRxiv a license to display the preprint in perpetuity.
It is made available under a CC-BY 4.0 International license .

Supplementary text, figures and tables

medRxiv preprint doi: https://doi.org/10.1101/2024.03.18.24304371; this version posted March 18, 2024. The copyright holder for this preprint
(which was not certified by peer review) is the author/funder, who has granted medRxiv a license to display the preprint in perpetuity.
It is made available under a CC-BY 4.0 International license .

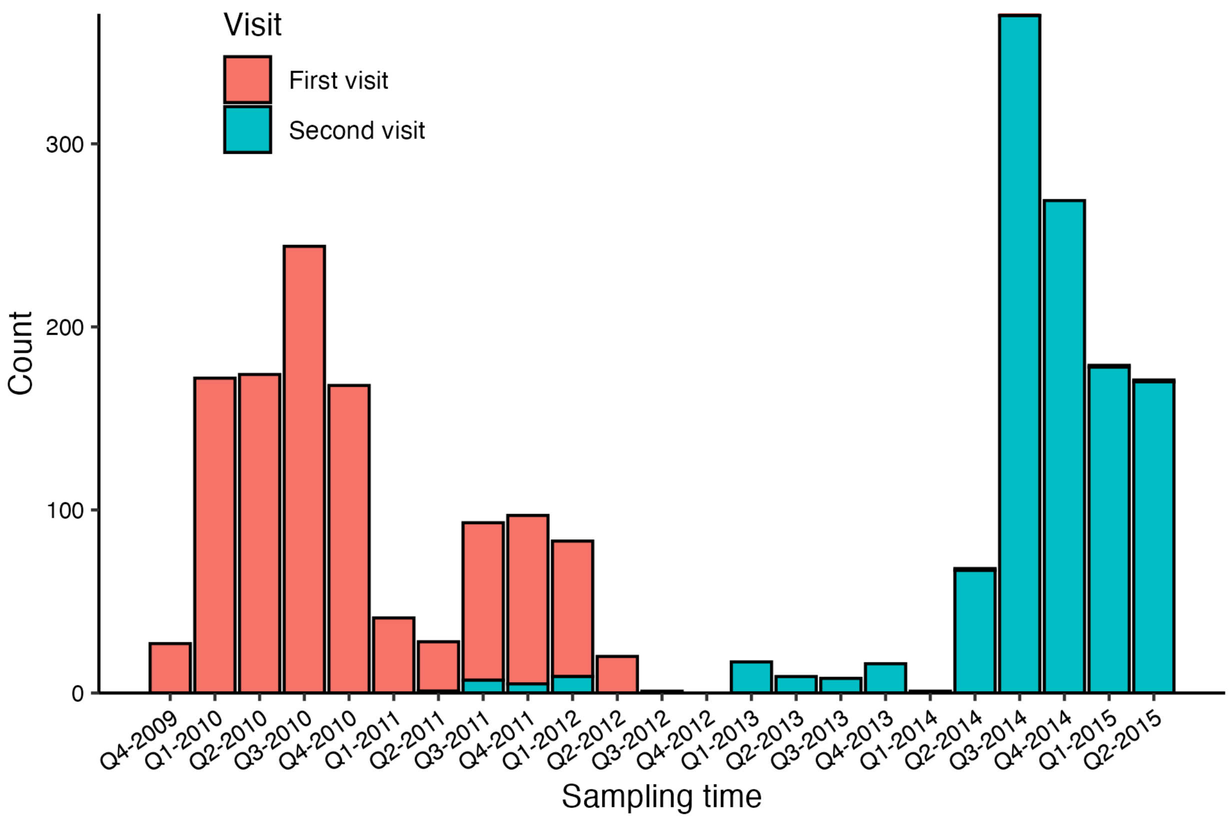

Figure S1: Distribution of serum sampling times from the Fluscape cohort. First and second visits refer to an individual's serum sample order, which may differ from the sample collection round of the overall study.

medRxiv preprint doi: https://doi.org/10.1101/2024.03.18.24304371; this version posted March 18, 2024. The copyright holder for this preprint **(which was not certified by peer review)** is the author/funder, who has granted medRxiv a license to display the preprint in perpetuity.
It is made available under a CC-BY 4.0 International license .

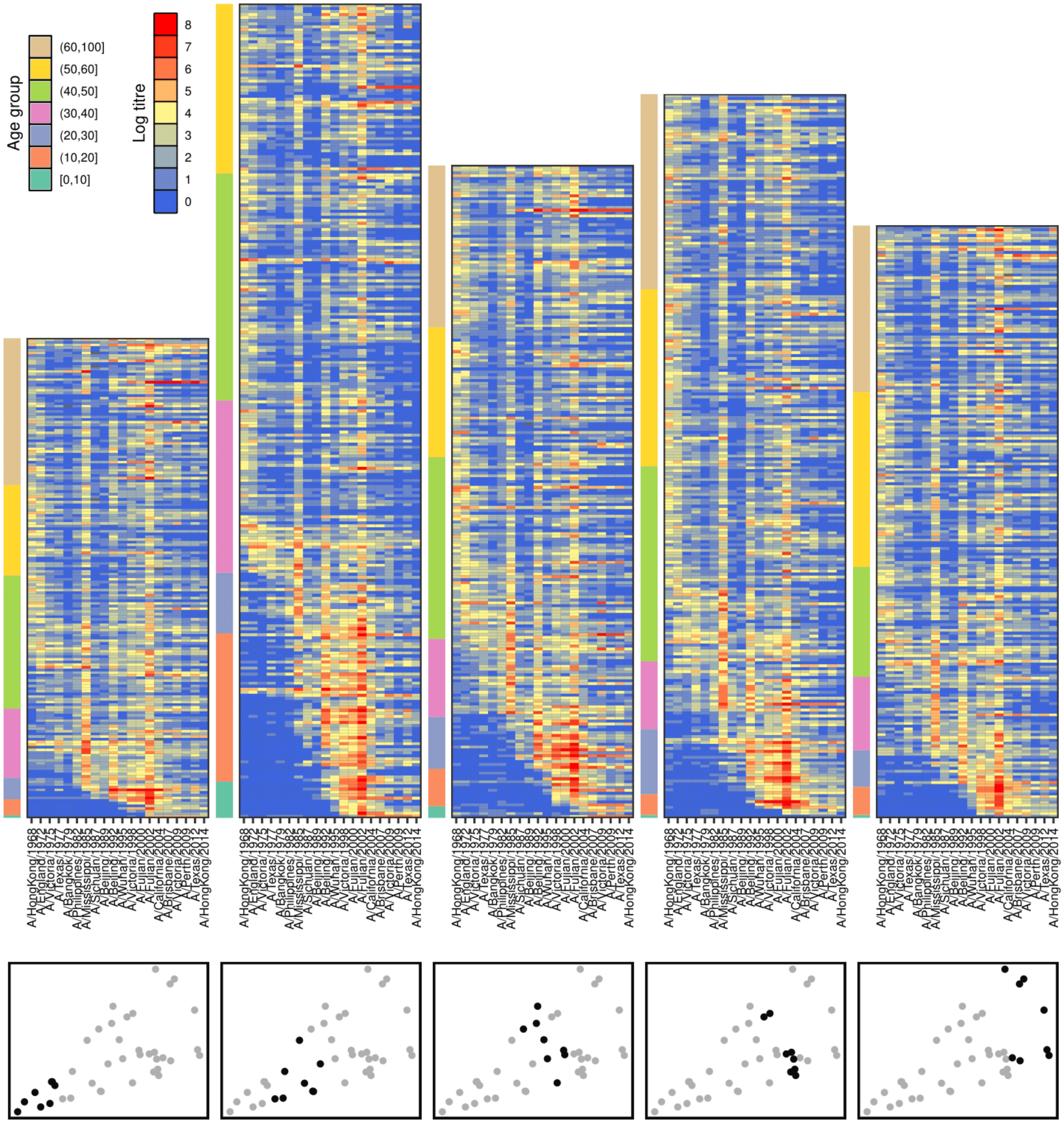

Figure S2: Distribution of log HI titres by study location at first serum sample. Each cell represents the log HI titre for one individual measured against one strain, shown on the x-axis. Locations were grouped into quintiles based on increasing distance from Guangzhou city center (bottom panels). Individuals were grouped by age and plotted with increasing age. Colors to the left of each subplot show age group.

medRxiv preprint doi: https://doi.org/10.1101/2024.03.18.24304371; this version posted March 18, 2024. The copyright holder for this preprint
(which was not certified by peer review) is the author/funder, who has granted medRxiv a license to display the preprint in perpetuity.
It is made available under a CC-BY 4.0 International license .

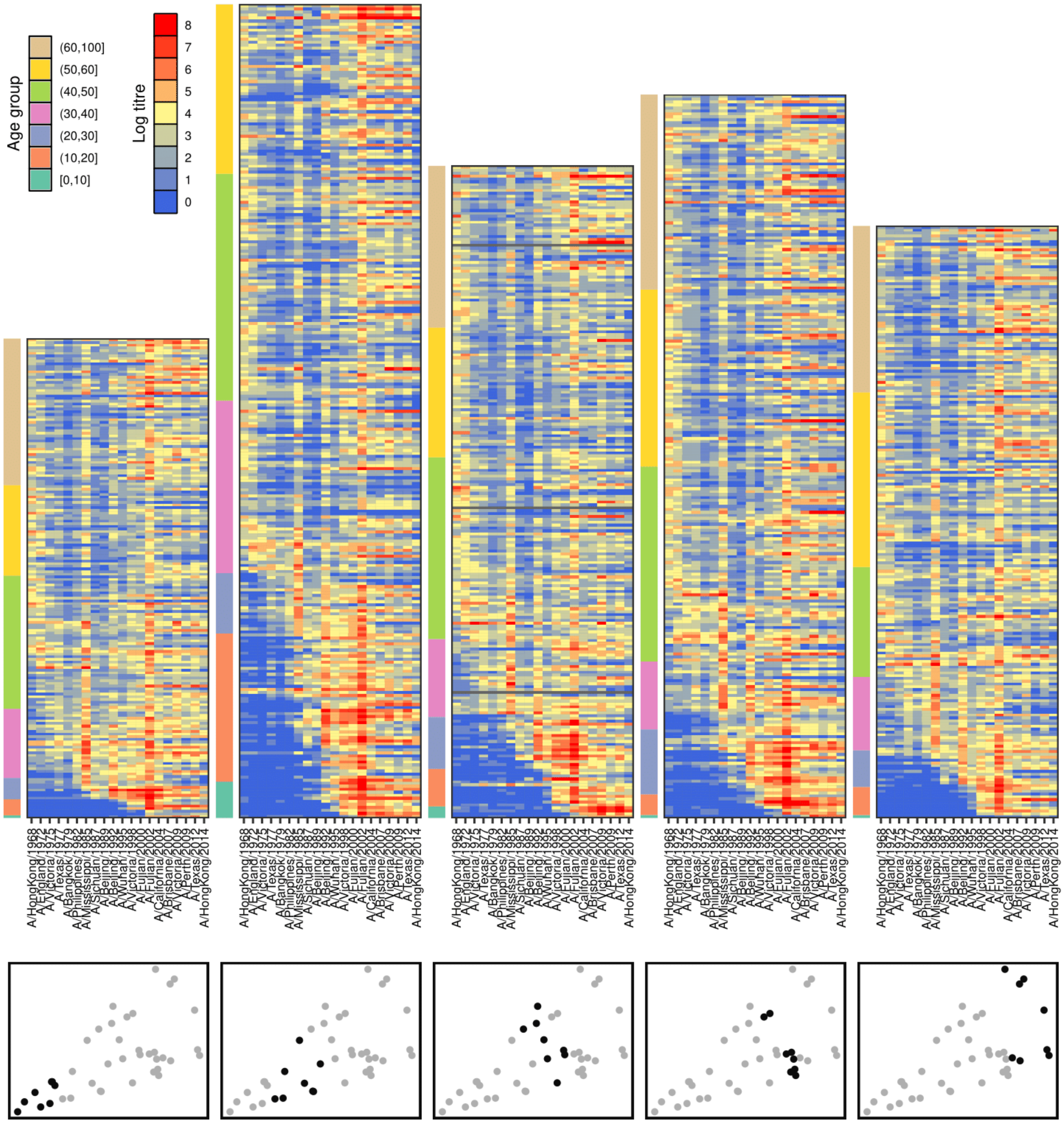

Figure S3: Distribution of log HI titres by study location at second serum sample. Each cell represents the log HI titre for one individual measured against one strain, shown on the x-axis. Locations were grouped into quintiles based on increasing distance from Guangzhou city center (bottom panels). Individuals were grouped by age and plotted with increasing age. Colors to the left of each subplot show age group.

medRxiv preprint doi: https://doi.org/10.1101/2024.03.18.24304371; this version posted March 18, 2024. The copyright holder for this preprint (which was not certified by peer review) is the author/funder, who has granted medRxiv a license to display the preprint in perpetuity.
It is made available under a CC-BY 4.0 International license .

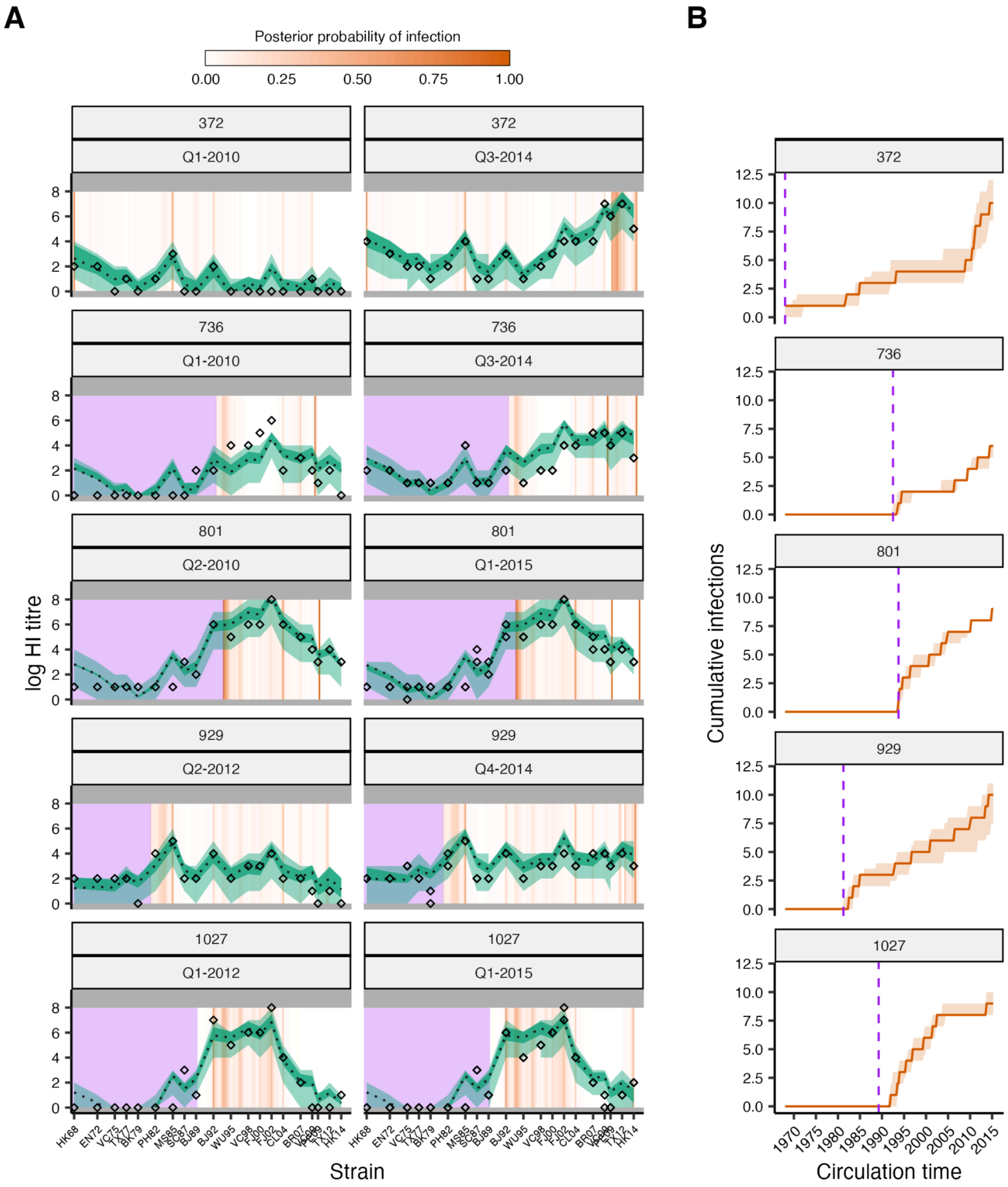

Figure S4: Example inferred latent antibody titres and infection histories. (**A**) Model-predicted titres compared to observed HI titres at each sampling time for 5 randomly selected individuals. Rows represent individuals. Subplots show antibody titres based on serum samples taken at that time. X-axis represents a position along the antigenic summary path. Black diamonds show observed titres. Black line and green shaded regions show posterior median and 95% credible intervals (CrI) on model-predicted latent titres (dark green) and 95% prediction intervals (light green). Orange bars show posterior probability of infection in that 3-month window. Gray rectangles denote the limit of detection of the HI assay. Purple rectangles show time periods before birth. (**B**) Posterior median and 95% CrI for the cumulative number of infections over time from birth (purple dashed line).

medRxiv preprint doi: https://doi.org/10.1101/2024.03.18.24304371; this version posted March 18, 2024. The copyright holder for this preprint (which was not certified by peer review) is the author/funder, who has granted medRxiv a license to display the preprint in perpetuity.
It is made available under a CC-BY 4.0 International license .

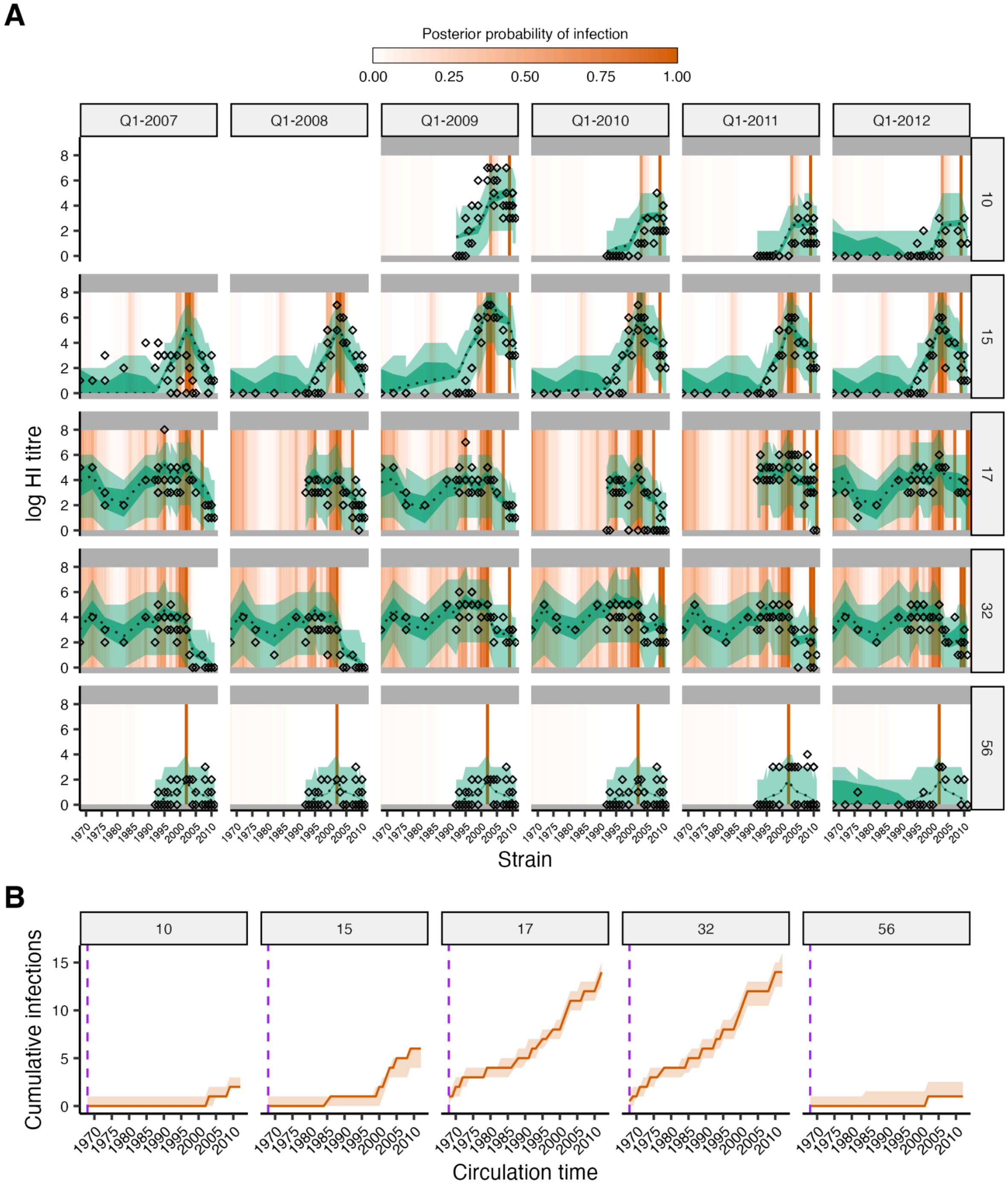

Figure S5: Model fit to data from Ha Nam, Viet Nam. (**A**) Model-predicted titres compared to observed HI titres at each sampling time for five randomly selected individuals, as in Fig S4. Diamonds show titre measurements; green shaded region shows 95% CrI and 95% prediction intervals; dashed line shows posterior median; orange bars show posterior probability of infection in a given time window. (**B**) Posterior median and 95% credible intervals (CrI) for the cumulative number of infections over time from birth (orange). Note that date of birth information was not available for these individuals.

medRxiv preprint doi: https://doi.org/10.1101/2024.03.18.24304371; this version posted March 18, 2024. The copyright holder for this preprint
(which was not certified by peer review) is the author/funder, who has granted medRxiv a license to display the preprint in perpetuity.
It is made available under a CC-BY 4.0 International license .

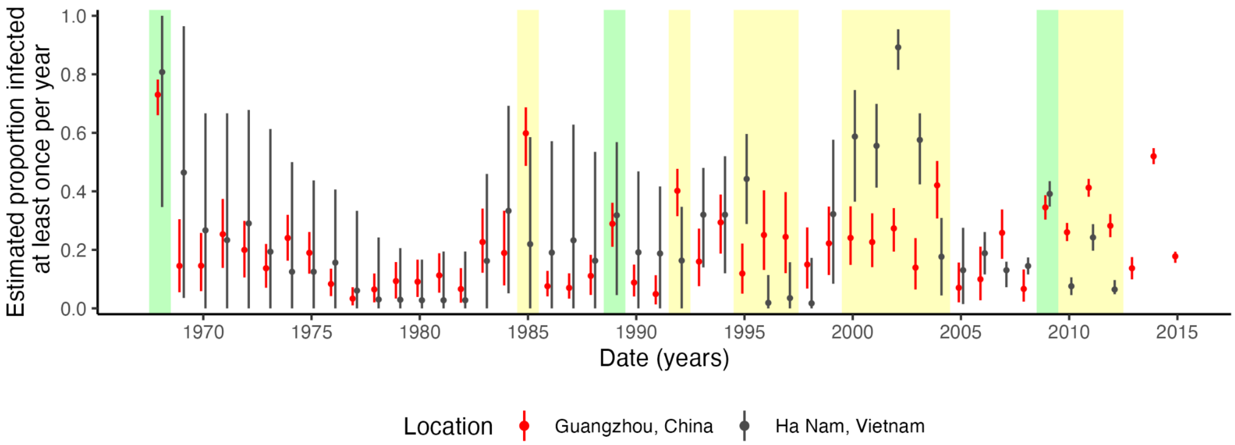

Figure S6: Comparison of annual attack rates using data from Ha Nam, Viet Nam and the Fluscape study in Guangzhou, China. Annual attack rates were defined here as the proportion of individuals who experienced at least one infection per year. Green shaded regions show notable time periods where the two datasets return similar attack rate estimates, whereas yellow shaded regions show time periods with notably different attack rate estimates. Attack rates in 1968, 1989 and 2009 were remarkably similar between the two locations. Some time periods showed high uncertainty for the Ha Nam dataset, as few individuals in the sample were alive during that time (e.g., 1969-1980). Attack rate estimates were very different from 2000 to 2004, with much higher attack rates estimated for the Ha Nam cohort. This might reflect a genuine different in A/H3N2 epidemiology during that time, but may also be partially driven by systematic biases in titre measurements to strains isolated during that time period – the fits to the Fluscape data include a positive offset term for titres against A/Fujian/2002, which leads to lower attack rate estimates in that time period, whereas fits to the Ha Nam data do not. The time period from 2010-2012 is also interesting, showing similar relative patterns but much higher attack rate estimates overall in the Fluscape cohort.

medRxiv preprint doi: https://doi.org/10.1101/2024.03.18.24304371; this version posted March 18, 2024. The copyright holder for this preprint (which was not certified by peer review) is the author/funder, who has granted medRxiv a license to display the preprint in perpetuity.
It is made available under a CC-BY 4.0 International license .

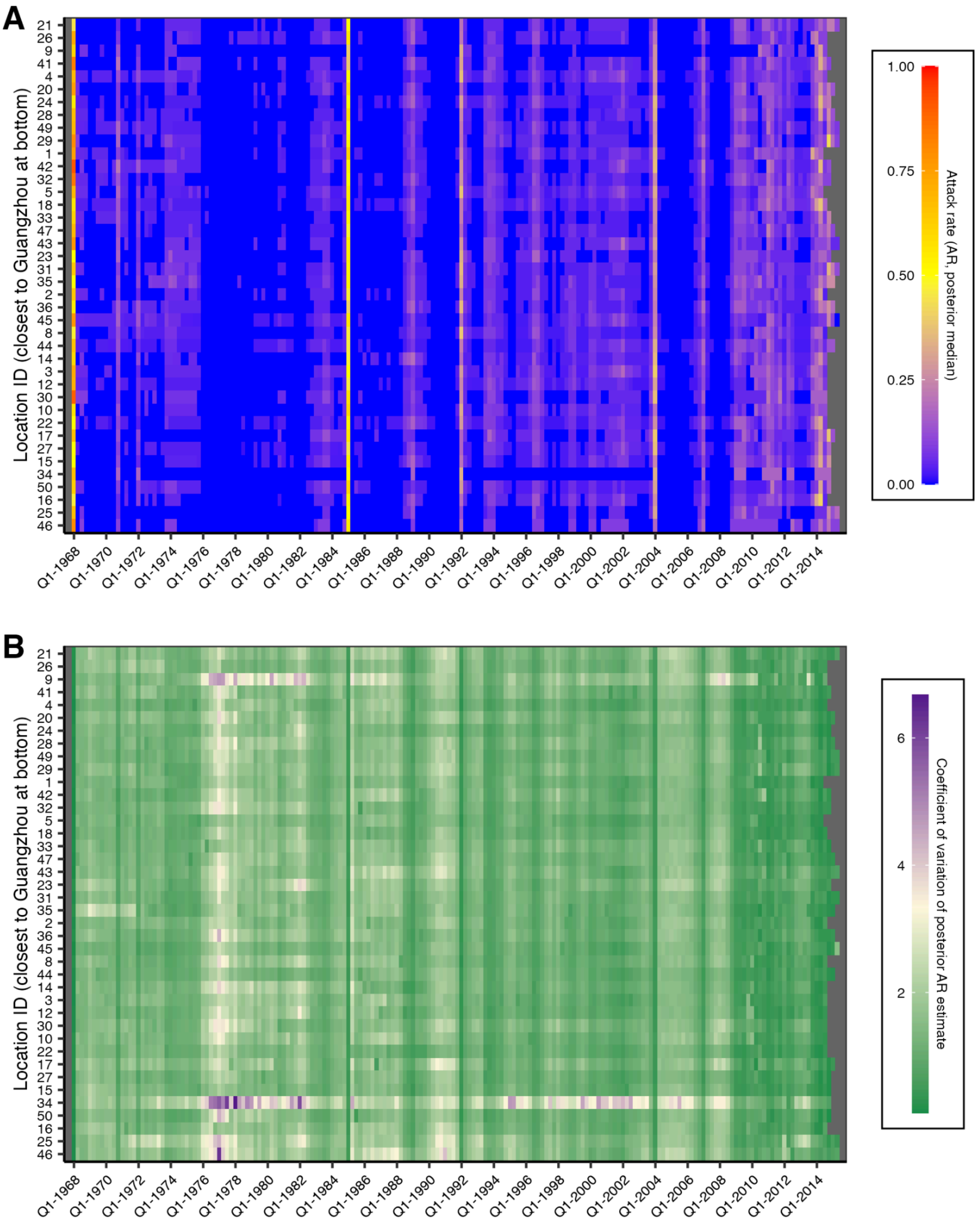

Figure S7: Quarterly attack rates across the 40 Fluscape study locations. Each row represents one study location ordered by increasing distance from Guangzhou city center. Each column represents a 3 month period. Cells are shaded by (**A**) the posterior median inferred attack rate or (**B**) the coefficient of variation of the posterior quarterly attack rate estimate for each location.

medRxiv preprint doi: https://doi.org/10.1101/2024.03.18.24304371; this version posted March 18, 2024. The copyright holder for this preprint
(which was not certified by peer review) is the author/funder, who has granted medRxiv a license to display the preprint in perpetuity.
It is made available under a CC-BY 4.0 International license .

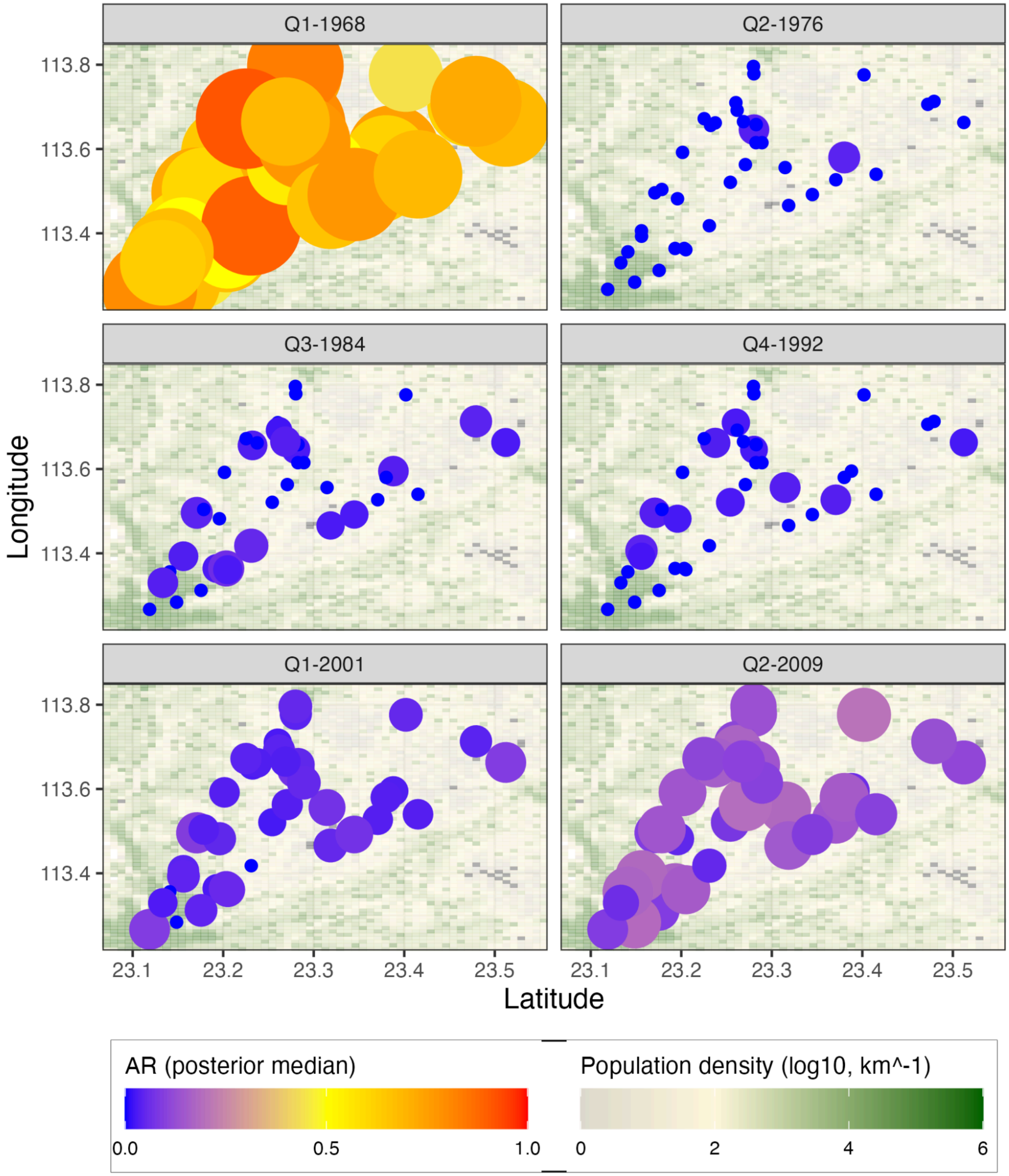

Figure S8: Distribution of quarterly attack rates by location over time. Each panel is one frame from a full animation available in Supplementary Material 3. Each colored point shows the inferred attack rate in each of the 40 locations, with size and shading reflecting the posterior median attack rate. Underlying the plot is a map of the study area, with each grid cell shaded by its log_{10} population density.

medRxiv preprint doi: https://doi.org/10.1101/2024.03.18.24304371; this version posted March 18, 2024. The copyright holder for this preprint (which was not certified by peer review) is the author/funder, who has granted medRxiv a license to display the preprint in perpetuity.
It is made available under a CC-BY 4.0 International license .

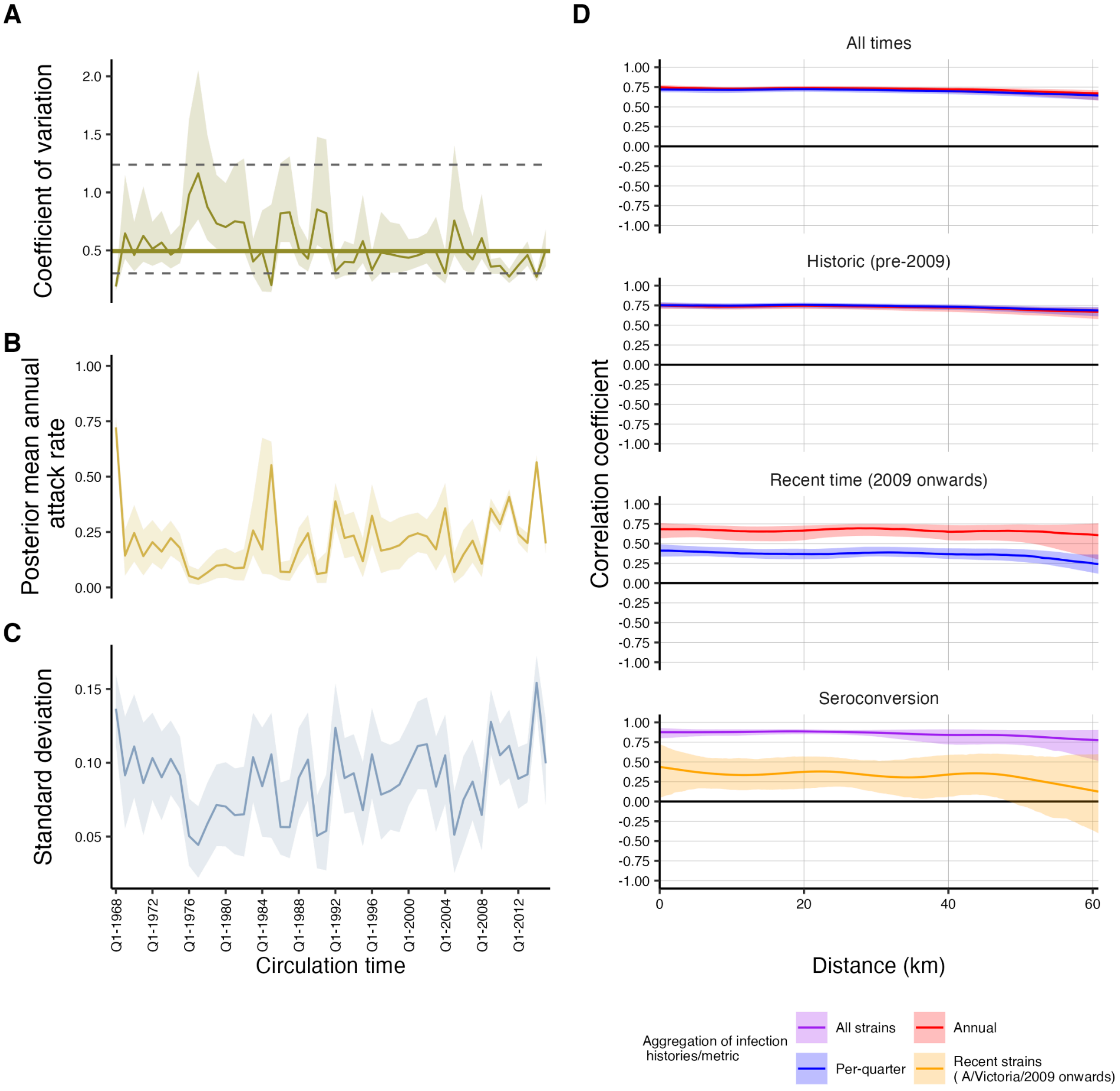

Figure S9: Spatial variation in annual attack rate estimates over time and correlation between nearby locations. (A) Coefficient of variation, (**B**) overall mean and (**C**) standard deviation of posterior median annual attack rate estimates from the 40 study locations. Solid lines and shaded regions show posterior medians and 95% credible intervals. In (**A**), the solid horizontal line shows the overall mean coefficient of variation across all time. Dashed horizontal lines show 95% quantiles of simulated coefficients of variation under the assumption that attack rates are the same across space. (**D**) Fitted spline correlograms showing spatio-temporal correlation in attack rates and proportion seroconverted with increasing distance. The first three plots show the spline correlogram calculated using the *Scnf* function from the *ncf* R-package for each of 100 posterior samples for the 40 location-specific attack rates. Solid lines and shaded regions show median and 95% quantiles of the predicted covariance function for these 100 samples, coloured by the level of aggregation used to calculate the attack rates. Each subplot shows the same calculation using either attack rates from all times, prior to Q1-2009 or Q1-2009 onwards. For the final plot ("Seroconversion"*)*, we calculated the spatial correlation in the proportion seroconverted in each of the 40 study locations, treating strain isolation time as the

medRxiv preprint doi: https://doi.org/10.1101/2024.03.18.24304371; this version posted March 18, 2024. The copyright holder for this preprint (which was not certified by peer review) is the author/funder, who has granted medRxiv a license to display the preprint in perpetuity.
It is made available under a CC-BY 4.0 International license .

time variable. Solid lines and shaded regions show median and 95% quantiles of 1000 bootstrapped observations. We repeated the analysis using either seroconversion to all strains, or only A/Victoria/2009, A/Perth/2009, A/Texas/2012 and A/HongKong/2014.

medRxiv preprint doi: https://doi.org/10.1101/2024.03.18.24304371; this version posted March 18, 2024. The copyright holder for this preprint (which was not certified by peer review) is the author/funder, who has granted medRxiv a license to display the preprint in perpetuity.
It is made available under a CC-BY 4.0 International license .

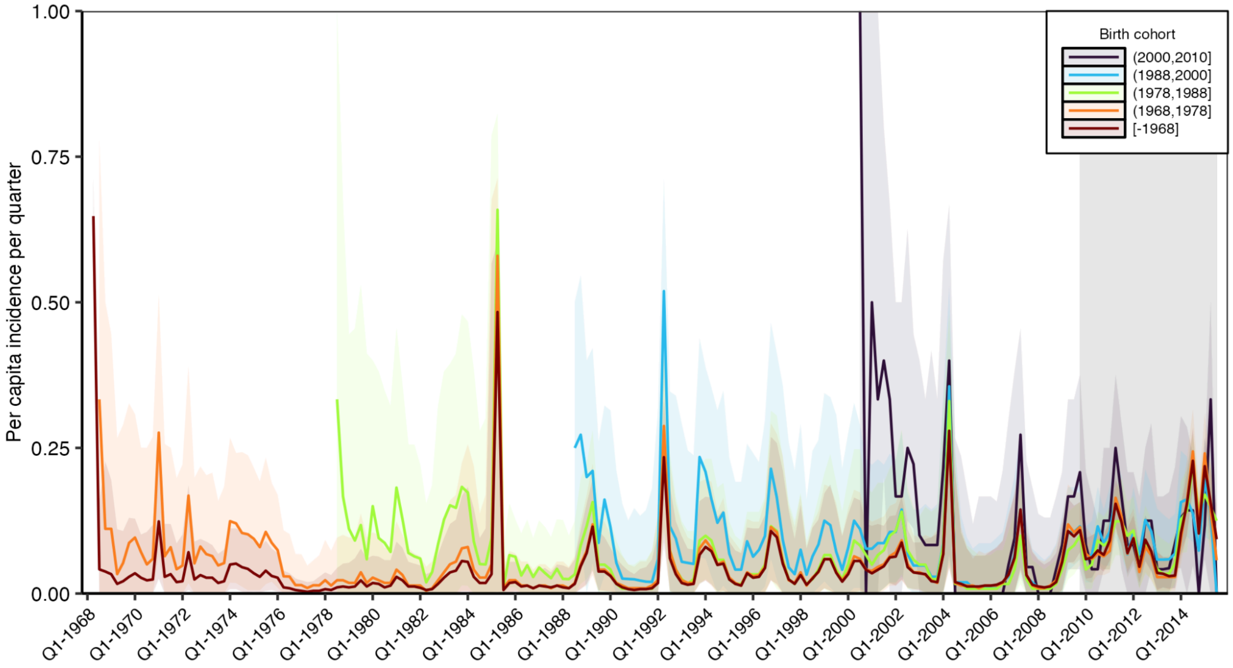

Figure S10: Quarterly attack rates by birth cohort. Model predicted per-capita incidence per quarter stratified into 5 birth cohorts. Attack rates were estimated by dividing the number of inferred infections by the number alive in each birth cohort in each 3 month period. Solid lines show the posterior median estimate from 1000 posterior samples. Shaded regions show 95% credible intervals from 1000 posterior samples. Gray shaded box shows duration of the Fluscape study.

medRxiv preprint doi: https://doi.org/10.1101/2024.03.18.24304371; this version posted March 18, 2024. The copyright holder for this preprint (which was not certified by peer review) is the author/funder, who has granted medRxiv a license to display the preprint in perpetuity.
It is made available under a CC-BY 4.0 International license .

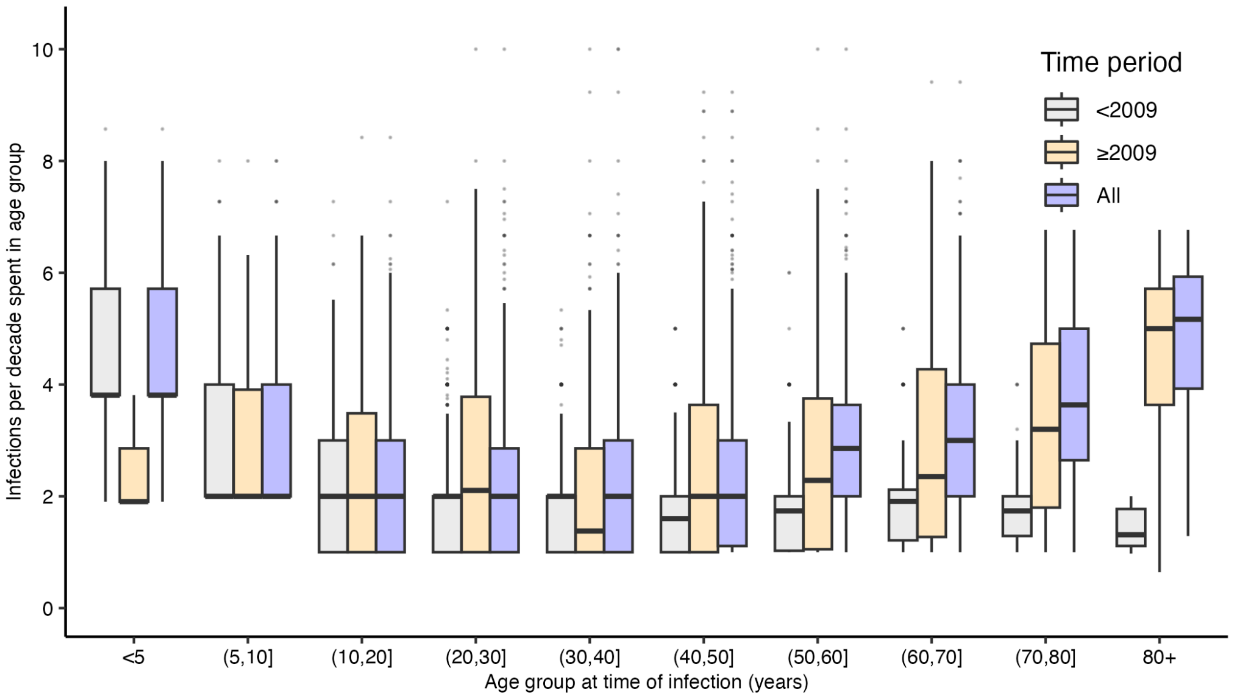

Figure S11: Posterior median number of infections per 10 year period stratified by age group at the time of infection, including either all time periods (blue), time periods during the Fluscape study period (Q4-2009 onward; orange) or only time periods prior to the Fluscape study (prior to Q4-2009; grey). Excludes infection states for individuals who spent less than 2 years in that age group. We present infection rate estimates using only infections from time periods prior to the first serum sample in Q4-2009 in Figure 3C. This is because there are many individuals representing the oldest age group at time of infection for time periods post Q4-2009, but relatively few from pre Q4-2009 (as individuals who were very old in historical time periods are no longer alive). In contrast, younger age groups are better represented across historical time periods (as those individuals are still alive at the time of sampling). Combining this biased representation of older individuals with much higher estimated incidence rates in recent time periods weighs the infection rate estimates for the older age groups much higher simply because most of their infections come from this time period. Therefore, we present age-stratified infection rate estimates using only pre Q4-2009 infections in the main text.

medRxiv preprint doi: https://doi.org/10.1101/2024.03.18.24304371; this version posted March 18, 2024. The copyright holder for this preprint (which was not certified by peer review) is the author/funder, who has granted medRxiv a license to display the preprint in perpetuity.
It is made available under a CC-BY 4.0 International license .

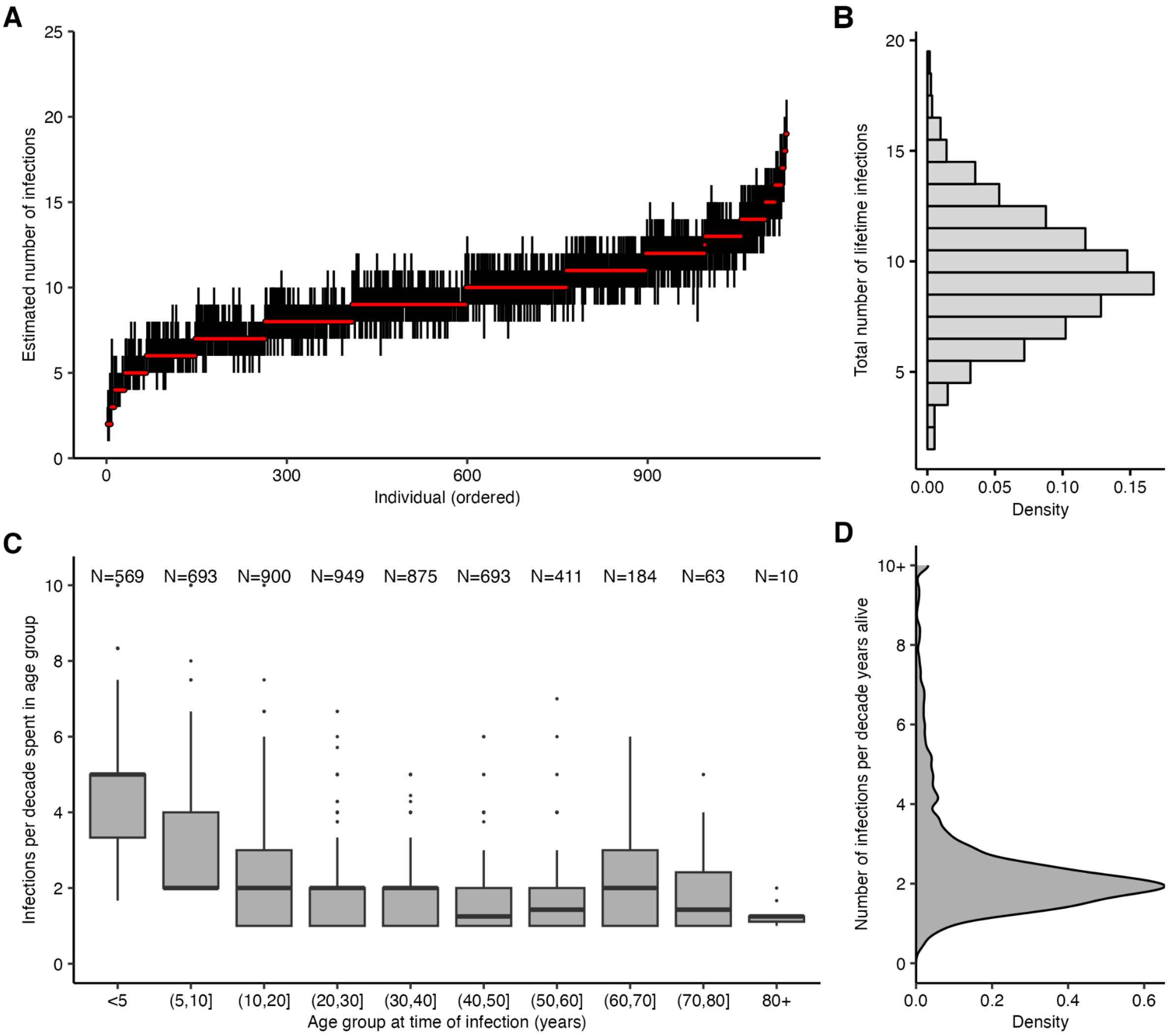

Figure S12: Age-specific patterns of infection under an alternative infection history model using estimates from the model version described in Supplementary Text 1. Results shown are identical to those in Figure 3, but assuming that (i) individuals can only be infected once per year (i.e., annual resolution infection histories rather than quarterly); (ii) the infection history model is placed upon an individual's total number of lifetime infections and not their per-time probability of infection (see [35] for further detail on implications of different prior assumptions); (iii) we did not remove runs of continuous infections from the posteriors. (**A**) Pointrange plot shows median and 95% CrI on the total number of lifetime infections for each individual, ordered by increasing age. (**B**) Distribution of the total number of infections across all individuals based on the posterior median total number of infections. (**C**) Posterior median number of infections per 10 year period stratified by age group at the time of infection, excluding individuals who spent less than 2 years in that age group, and including only time periods prior to Q4-2009. Text shows sample size within each age group – note this does not sum to the number of individuals in the sample, as individuals contribute to multiple age groups during their lifetime. (**D**) Posterior median number of infections per 10 years alive across all individuals. Under this prior, we estimated that individuals are infected 2.08 times per 10 year period (posterior median; 95% CrI: 1.04-7.62).

medRxiv preprint doi: https://doi.org/10.1101/2024.03.18.24304371; this version posted March 18, 2024. The copyright holder for this preprint (which was not certified by peer review) is the author/funder, who has granted medRxiv a license to display the preprint in perpetuity.
It is made available under a CC-BY 4.0 International license .

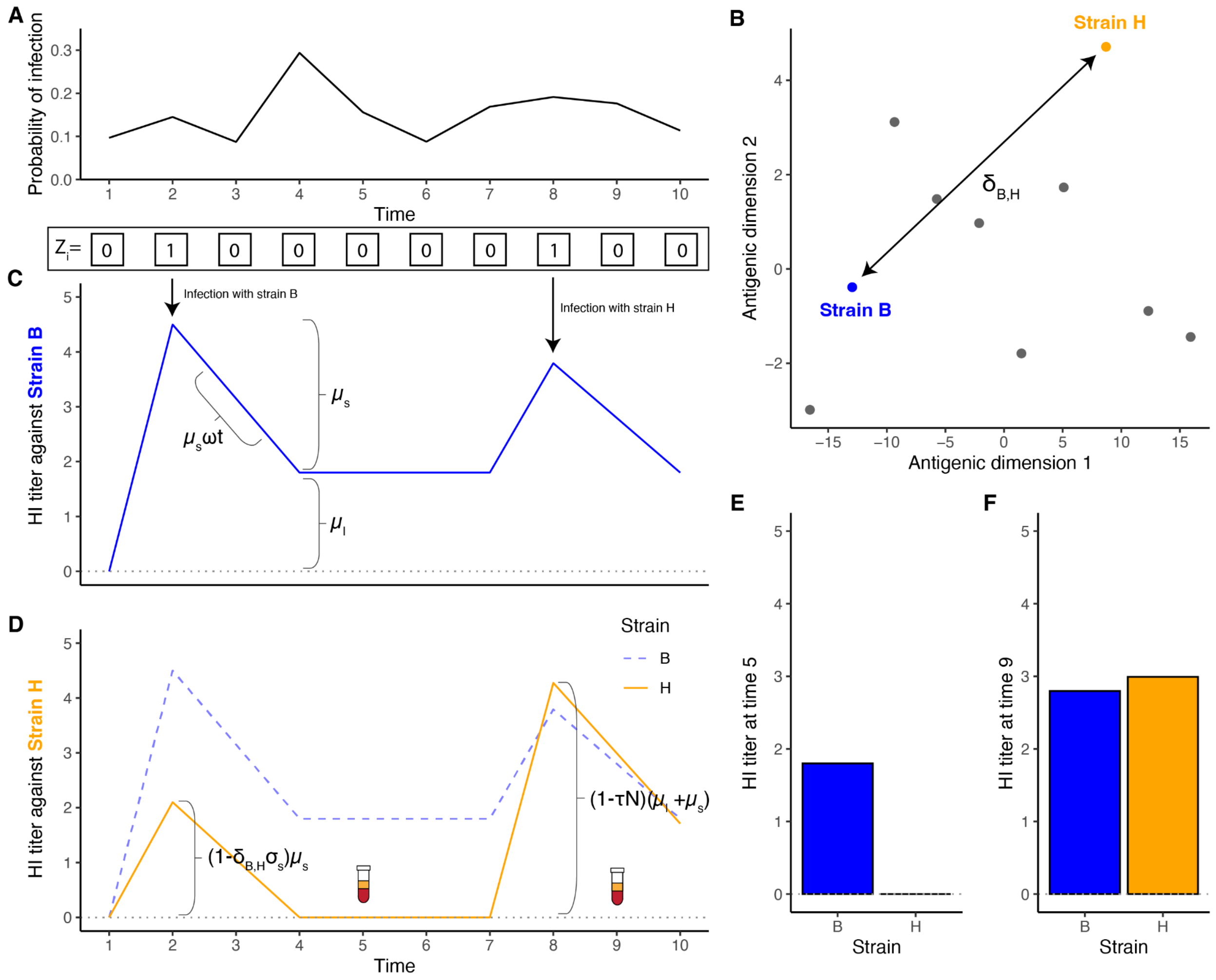

Figure S13: Schematic of the full *serosolver* model representing a single individual infected with two strains, B and H, over a 10 year time period. (**A**) Example, randomly generated population-level infection probabilities. At the population level, the model describes a per-time-period probability of infection applied to the whole whole population. These probabilities are used to simulate a vector of latent binary infection states, Z_i, for each individual as a series of independent Bernoulli trials (shown as a vector of 1s and 0s). (**B**) The antigenic relatedness of A/H3N2 strains is given by an antigenic map, where the degree of cross reactivity between any two strains is given by their euclidean distance on the map. (**C**) Antibody levels against the infecting strain (strain B) are boosted and wane, given by the summation of transient short-term boosting and persistent long-term boosting. (**D**) Infection with strain B also induces cross-reactive antibodies against all other strains, here showing antibody levels to strain H. The degree of cross-reactivity is proportional to the antigenic distance between the infecting and measured strain. Later on, the individual is infected again, this time with strain H, inducing further antibody boosting and waning. An antigenic seniority parameter, τ, reduces each successive boost as a function of the number of previous infections, N. Snapshots of these underlying antibody kinetics are observed through serum samples (blood vials; (**E**) and (**F**)) distributed according to a truncated, discretized normal distribution with standard deviation parameter ε.

medRxiv preprint doi: https://doi.org/10.1101/2024.03.18.24304371; this version posted March 18, 2024. The copyright holder for this preprint (which was not certified by peer review) is the author/funder, who has granted medRxiv a license to display the preprint in perpetuity.
It is made available under a CC-BY 4.0 International license .

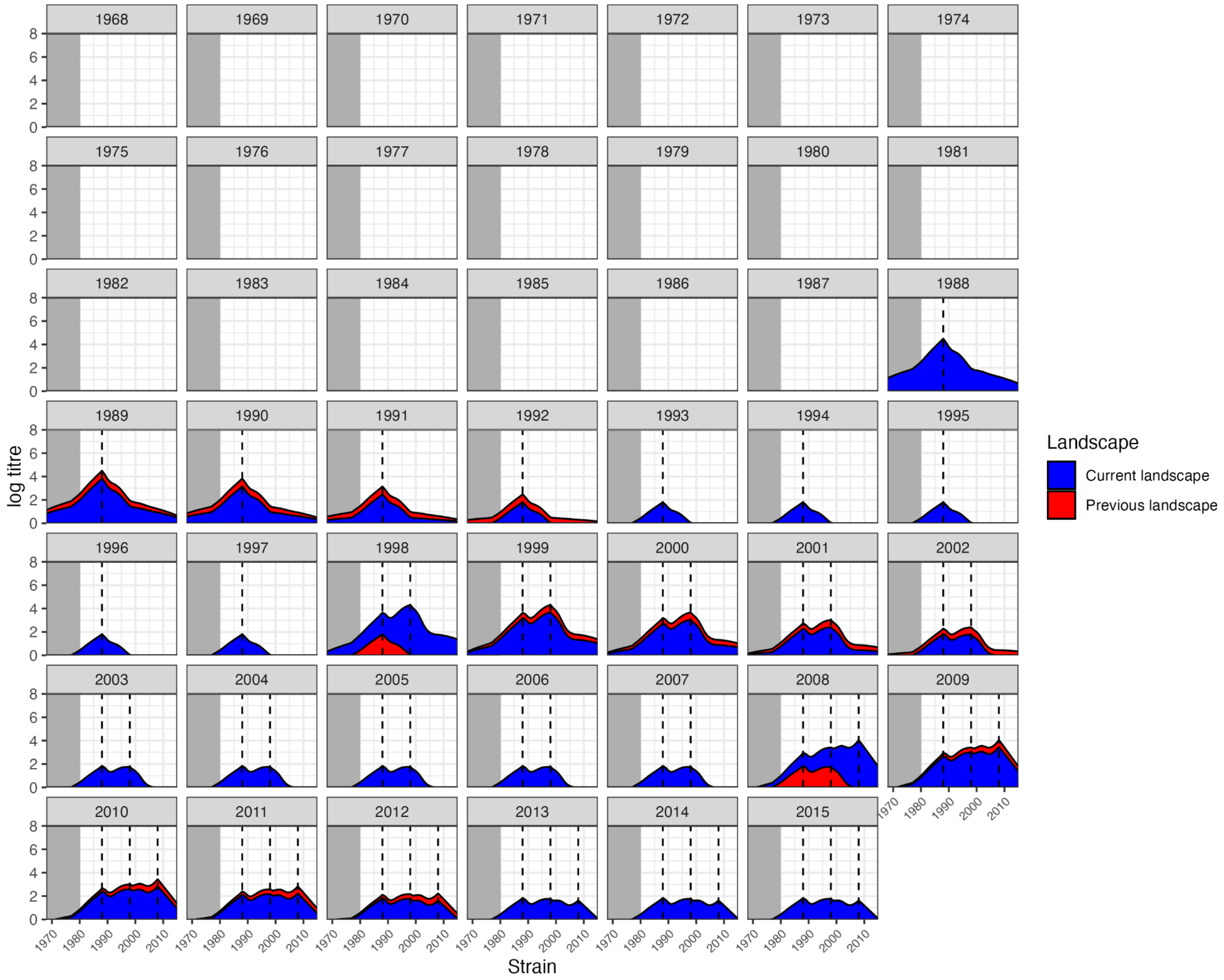

Figure S14: Simulated antibody landscapes and infection histories over time for one individual using the antibody kinetics model. Each subplot shows the antibody landscape for that time period. The blue region gives the antibody landscape in that time period, whereas the red region gives the antibody landscape in the preceding time period. The x-axis of each subplot gives the identity of the strain assumed to be circulating in that year. The grey region shows the time period and thus strains that circulated before the individual was born. Vertical dashed lines give the timing/strains the individual was infected with.

medRxiv preprint doi: https://doi.org/10.1101/2024.03.18.24304371; this version posted March 18, 2024. The copyright holder for this preprint (which was not certified by peer review) is the author/funder, who has granted medRxiv a license to display the preprint in perpetuity.
It is made available under a CC-BY 4.0 International license .

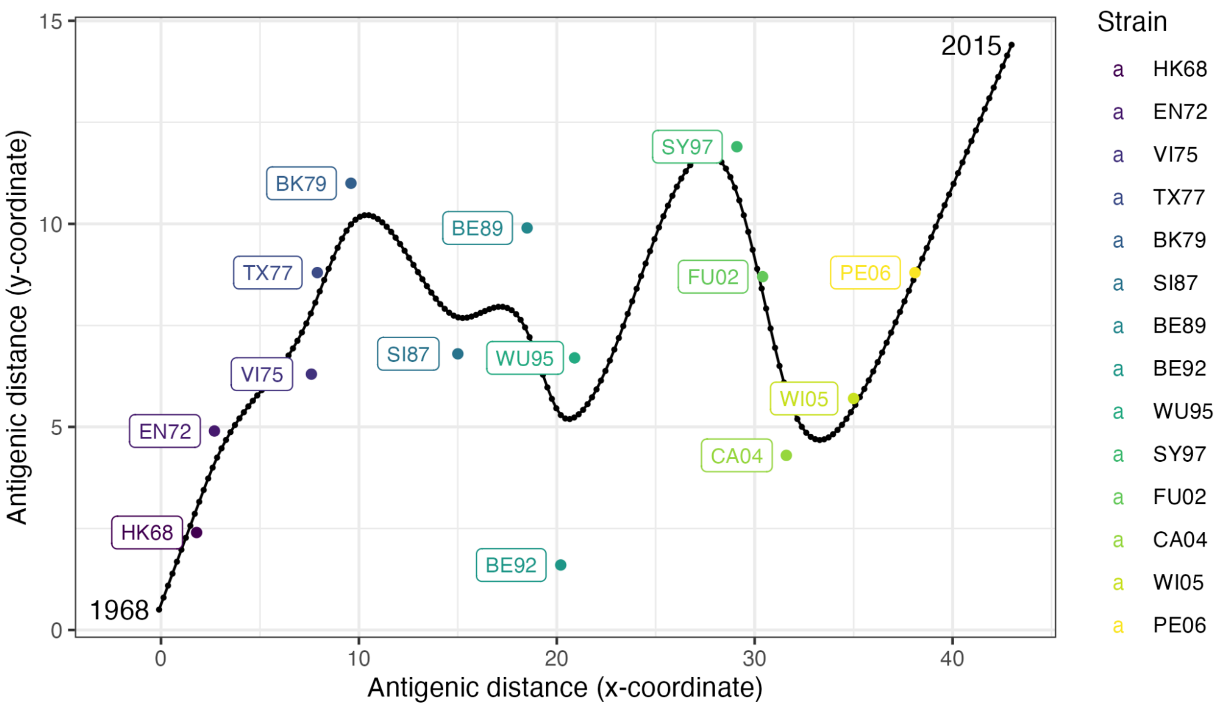

Figure S15: Antigenic coordinates of measured strains and strains assumed to be circulating in each time period. Each colored point shows the location of the labeled strain on the antigenic map given in [37]. Strains which are further apart are less antigenically similar, and therefore exhibit less cross-reactivity following a seroresponse. The smoothing spline shows the inferred coordinates of each strain j assumed to have circulated in each 3-month time period, where each black point shows the assumed location in successive time periods. First, a cubic smoothing spline was fitted to the locations of the measured strain with smoothing parameter 0.3. Second, a linear model was fitted to predict the x-coordinate as a function of the strain isolation time. Finally, we generated predicted x-coordinates for each possible j given the circulation time from the linear model, and then used the predicted x-coordinate to predict the y-coordinate from the fitted smoothing spline. The antigenic distance between each pair of strains k and j was then calculated based on their Euclidean distance.

medRxiv preprint doi: https://doi.org/10.1101/2024.03.18.24304371; this version posted March 18, 2024. The copyright holder for this preprint (which was not certified by peer review) is the author/funder, who has granted medRxiv a license to display the preprint in perpetuity.
It is made available under a CC-BY 4.0 International license .

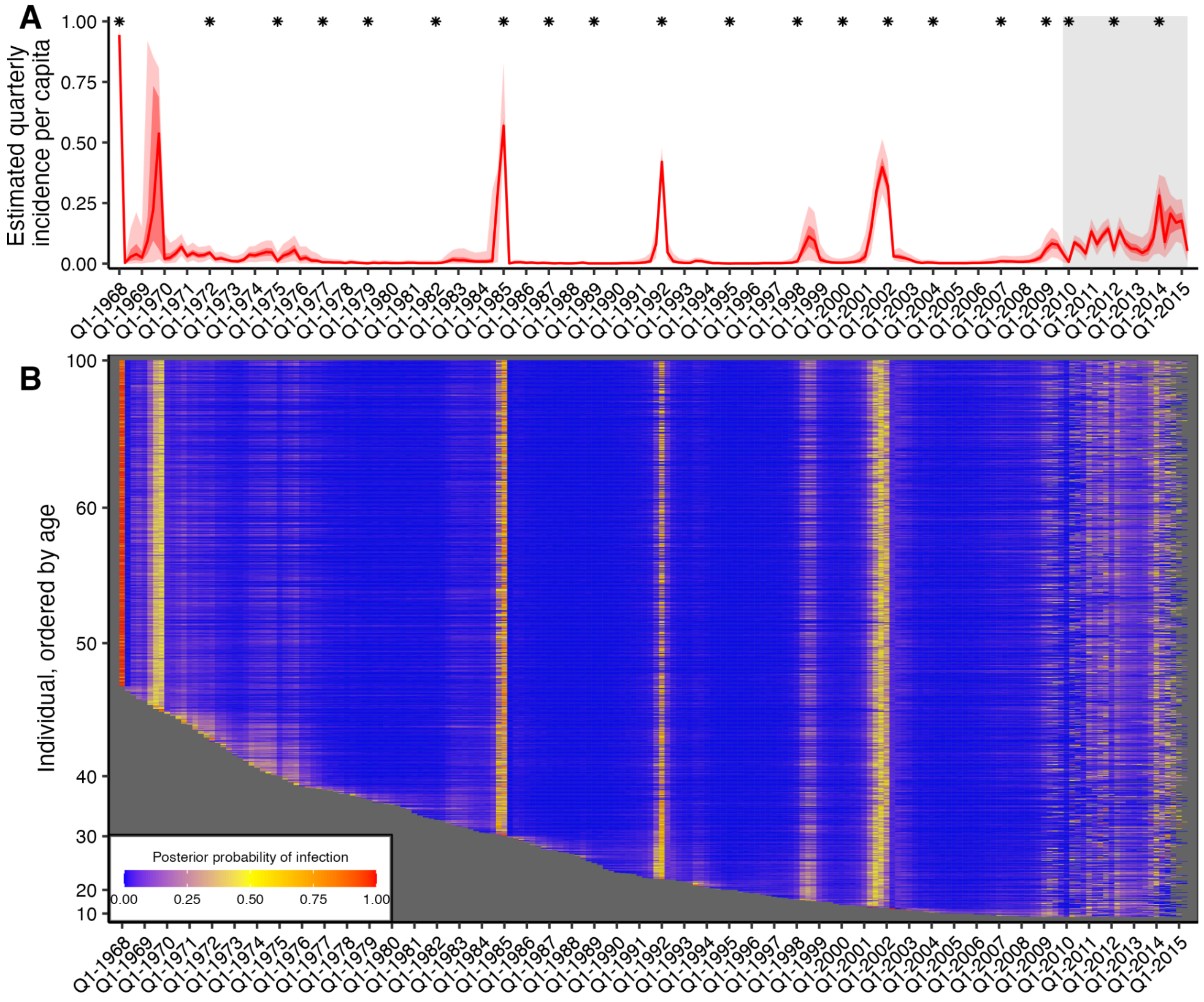

Figure S16: Quarterly incidence and individual infection histories from the Fluscape dataset without strain-specific measurement offsets. Identical to Figure 2, but without the inclusion of strain-specific measurement offsets in the observation model. (**A**) Model predicted per-capita incidence per quarter. Attack rates were estimated by dividing the number of inferred infections by the number alive in each 3 month period. Red line shows the posterior median estimate from 1000 posterior samples. Dark and light red shaded regions show 50% and 95% credible intervals respectively from 1000 posterior samples. Gray shaded box shows duration of the Fluscape study. Asterisks mark times from which a sample circulating strain was tested. (**B**) Inferred infection histories for each individual. Each row represents an individual ordered by increasing age. Each column represents the time of a potential infection. Cells are shaded based on the number of the posterior samples with an infection at that time divided by the total number of posterior samples for that infection state.

medRxiv preprint doi: https://doi.org/10.1101/2024.03.18.24304371; this version posted March 18, 2024. The copyright holder for this preprint (which was not certified by peer review) is the author/funder, who has granted medRxiv a license to display the preprint in perpetuity.
It is made available under a CC-BY 4.0 International license .

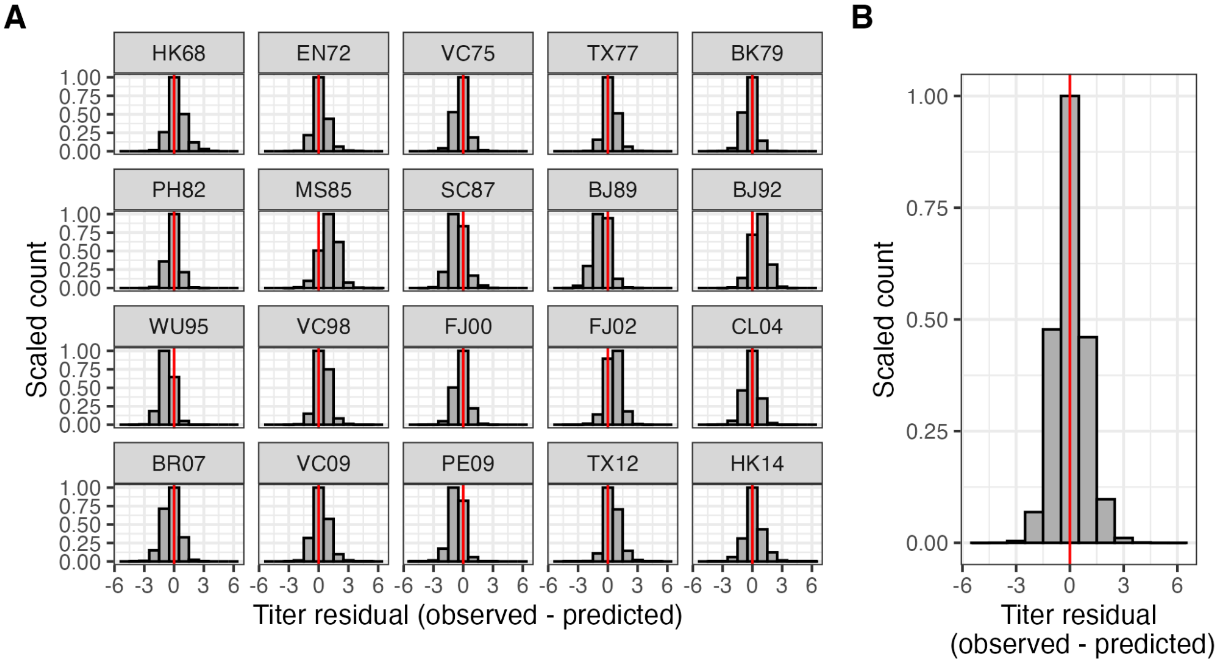

Figure S17: **Distribution of antibody titre prediction errors (observed - model predicted) when fitting the *serosolver* model ignoring strain-specific measurement offsets.** (**A**) Distribution of titre prediction errors stratified by tested A/H3N2 strain. (**B**) Overall distribution of titre prediction errors across all measured viruses. Vertical red line shows *x=0*; buckets to the right of the red line suggest underestimation of titres; histograms to the left of the red line buckets suggest overestimation of titres.

medRxiv preprint doi: https://doi.org/10.1101/2024.03.18.24304371; this version posted March 18, 2024. The copyright holder for this preprint (which was not certified by peer review) is the author/funder, who has granted medRxiv a license to display the preprint in perpetuity.
It is made available under a CC-BY 4.0 International license .

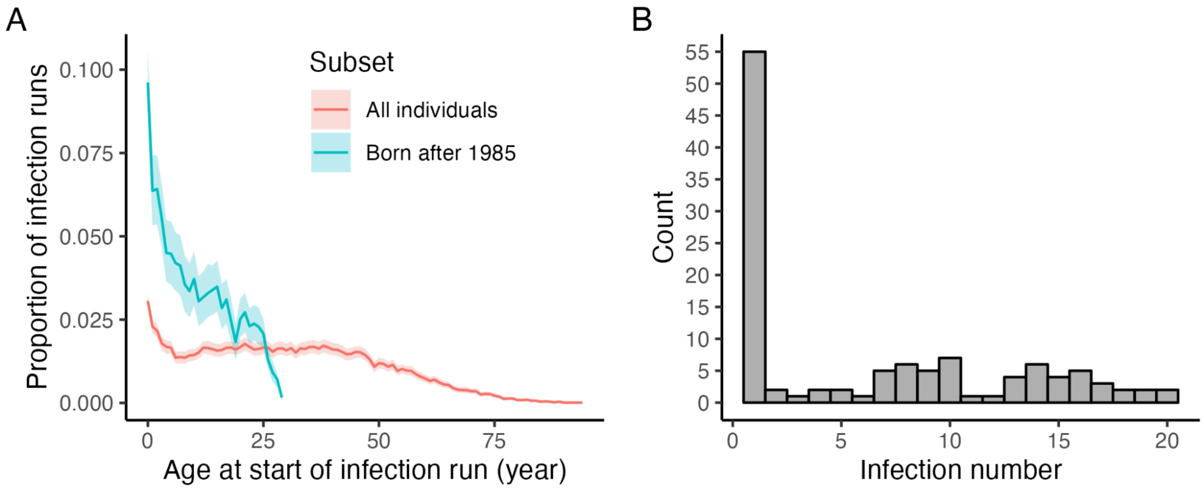

Figure S18: Runs of repeated infections are disproportionately more likely to occur early in life. (**A**) Proportion of infection episodes which are estimated as runs of consecutive infections by age at start of infection run, using either all infection episodes or only those from individuals born after 1985. (**B**) Number of inferred infection episodes which are estimated as runs of consecutive infections stratified by the infection order in each individual's infection history. For example, if the run is the first infection an individual has experienced, this is given an infection number of one. Of the 10,558 distinct infection episodes, 757 (posterior median; 95% CrI: 676-861) were runs of two consecutive infections, 79 (posterior median; 95% CrI: 62-97) were runs of 3 consecutive infections and 19 (posterior median; 95% CrI: 13-27) were runs of 4 or more.

medRxiv preprint doi: https://doi.org/10.1101/2024.03.18.24304371; this version posted March 18, 2024. The copyright holder for this preprint (which was not certified by peer review) is the author/funder, who has granted medRxiv a license to display the preprint in perpetuity.
It is made available under a CC-BY 4.0 International license .

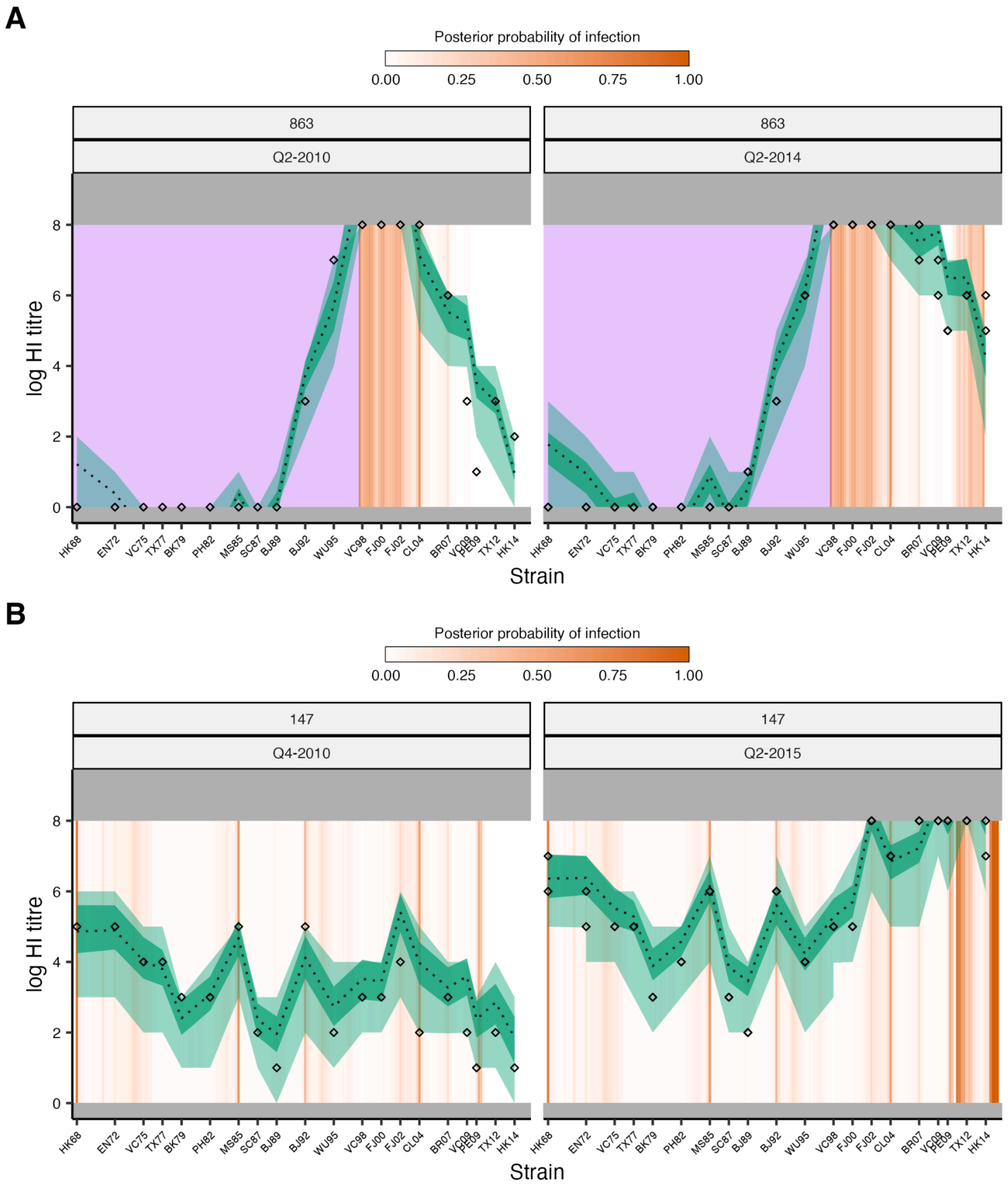

Figure S19: Model fits demonstrating typical profiles where consecutive infection runs were imputed. Model-predicted titres compared to observed HI titres at each sampling time for two individuals, as in Fig S4. Diamonds show titre measurements; green shaded region shows 95% CrI and 95% prediction intervals; dashed line shows posterior median; orange bars show posterior probability of infection in a given time window. Purple rectangles show time periods prior to birth. (**A**) Example of an individual estimated to have experienced multiple consecutive infections immediately following birth to explain high titres. (**B**) Example of an individual estimated to have experienced multiple consecutive infections between the two serum sampling times to explain the drastic increase in titres to recently circulating strains.

medRxiv preprint doi: https://doi.org/10.1101/2024.03.18.24304371; this version posted March 18, 2024. The copyright holder for this preprint (which was not certified by peer review) is the author/funder, who has granted medRxiv a license to display the preprint in perpetuity.
It is made available under a CC-BY 4.0 International license .

<< Separate file >>

Figure S20: Observed antibody profiles for all individuals in the Fluscape cohort. Each subplot shows antibody levels measured against each of the 20 H3N2 strains. The x-axis shows the isolation year of the measured strain. The areas are shaded by sample number, showing titre measurements from the first (blue) and second (red) samples. Grey rectangles mark strains which circulated before that individual was born. The vertical colored lines show the timing of the serum samples relative to the strain isolation times. Plots where the red region extends above the blue region reflect antibody boosting between the first and second serum sample. Where multiple titres were measured against the same strain from the same serum sample, we plotted the mean of the log titres.

<< Separate file >>

Figure S21: Observed changes in antibody titre for all individuals in the Fluscape cohort. As in Fig S20, but showing fold-change in titre against each strain between samples. Each subplot shows the change in antibody levels measured against each of the 20 H3N2 strains. The x-axis gives the isolation year of the measured strain. The vertical grey line shows the timing of birth or 1968, whichever was later. Bars are shaded orange to denote antibody boosting and green to denote antibody waning. Horizontal dash lines indicate 2-fold boosting or waning.

medRxiv preprint doi: https://doi.org/10.1101/2024.03.18.24304371; this version posted March 18, 2024. The copyright holder for this preprint (which was not certified by peer review) is the author/funder, who has granted medRxiv a license to display the preprint in perpetuity.
It is made available under a CC-BY 4.0 International license .

Table S1: self-reported vaccination status at time of first study visit (top) and between study visits (bottom). Percentages exclude individuals who declined to answer, were unsure, or had missing data.

**Self-reported vaccination at time of first serum sample (N=1005)**				
Never vaccinated	This year	Last year	2-5 years	5+ years
88.3%	1.09%	3.08%	2.78%	4.77%

**Reported vaccination since last study visit**		
Visit 2 (N=822)	Visit 3 (N=1020)	Visit 4 (N=1119)
1.70%	1.57%	0.536%

medRxiv preprint doi: https://doi.org/10.1101/2024.03.18.24304371; this version posted March 18, 2024. The copyright holder for this preprint (which was not certified by peer review) is the author/funder, who has granted medRxiv a license to display the preprint in perpetuity.
It is made available under a CC-BY 4.0 International license .

Table S2: Estimated attack rates and infection patterns 2010-2014 prior to removing runs of consecutive infections. Percentages shown are posterior median and 95% credible intervals. Attack rate was defined as the proportion of individuals who were infected at least once in that year. "Reinfected" gives the percentage of people that were infected more than once in a year. Bottom table shows the percentage of individuals that were infected 0, 1, 2, 3, 4 or 5 times between 2010-2014 inclusive.

Year	Measured strain	Estimated attack rate (%)	Reinfected within same year (%)
2010	A/Perth/2009	28.8% (25.2%-32.1%)	4.25% (3.01%-5.4%)
2011	-	42.9% (39.9%-46.2%)	5.01% (3.49%-6.98%)
2012	A/Texas/2012	24.7% (20.9%-28.6%)	2.26% (1.17%-3.61%)
2013	-	20.8% (16.5%-24.9%)	8.41% (7.09%-9.97%)
2014	A/Hong Kong/2014	57.9% (55.0%-60.5%)	6.02% (5.00%-7.16%)

Proportion of individuals with different total number of infections from 2010-2014					
0	1	2	3	4	5
12.2% (11.2%-13.4%)	32.7% (30.7%-34.7%)	32.7% (30.8%-35.2%)	16.8% (15.0%-18.6%)	4.78% (3.67%-5.93%)	0.531% (0.177%-0.973 %)

medRxiv preprint doi: https://doi.org/10.1101/2024.03.18.24304371; this version posted March 18, 2024. The copyright holder for this preprint (which was not certified by peer review) is the author/funder, who has granted medRxiv a license to display the preprint in perpetuity.
It is made available under a CC-BY 4.0 International license .

Supplementary Text 1

1. Estimation of strain-specific measurement offsets

Our initial approach was to fit the full *serosolver* model exactly as described in the Materials and Methods, but without the inclusion of strain-specific measurement offsets (i.e., set all χ_j to 0). Although convergence diagnostics were acceptable, we noted that estimated attack rates were extremely high (or low) in some time periods (Fig S16). These unusually high or low estimates were associated with systematic under- or over-estimation of expected log HI titres compared to the observed values (Fig S17). For example, model predicted titres are systematically lower than observations for the viruses that circulated in years 1985 and 2002, but systematically higher for years 1995 and 2010. These strain-specific biases are potentially problematic for the attack rate estimates, as the model can only account for elevated titres against a particular strain through adding more infections. If some viruses have systematically higher antibody titres simply due to measurement bias, then the model may incorrectly infer more infections than occurred.

These systematic measurement biases have a number of possible biological explanations. Serum potency and virus avidity, resulting in different haemagglutinin (HA) reactivity [88,89], are known sources of variation when performing antigenic cartography using ferret sera. This effect was noted by Fonville et al. when using locally-weighted multiple linear regression to fit antibody landscape surfaces to antibody titre data. Fonville et al. found that some viruses had systematically under-predicted values (e.g., NL/620/89) whereas others were systematically overestimated (e.g., Victoria/361/11) [37]. This phenomenon was also described by Bedford et al. when simultaneously performing antigenic cartography alongside phylogenetic tree reconstruction using both genetic and HI titre data [90]. Bedford et al. found models that explicitly estimated parameters for “virus avidity” to represent the contribution of virus-specific effects to observed titres were better supported than models that did not.

Including a strain-specific offset term as part of the observation process enables the antibody kinetics and infection model described here to account for these systematic biases. In lieu of generating these strain-specific offset terms from experimental data, we would ideally jointly infer these offsets alongside the infection histories and antibody kinetics parameters. However, our attempts to do so using the infection history prior used in the main text were unsuccessful

medRxiv preprint doi: https://doi.org/10.1101/2024.03.18.24304371; this version posted March 18, 2024. The copyright holder for this preprint (which was not certified by peer review) is the author/funder, who has granted medRxiv a license to display the preprint in perpetuity.
It is made available under a CC-BY 4.0 International license .

and we could not generate converged chains. This is likely because the offset terms and attack rate estimates were highly correlated; elevated titres against a strain that circulated in a given year may be explained either by high seroresponse rates or systematic overestimation of titres to that strain.

Instead, we generated estimates for the measurement offsets using a modified version of the main text model. We fit the same antibody kinetics and infection histories to the full Fluscape dataset, but with three changes: (1) the observation model included an additional, estimated strain-specific offset parameter, χ_j, for each measured strain as described in the Materials and Methods; (2) we estimated infection histories at an annual rather than 3-monthly resolution to reduce the parameter space to be explored; (3) we used a different infection history prior (prior version 3 in *serosolver*) which led to much better convergence and identifiability of the offset terms at the expense of less interpretable attack rate estimates. This alternative prior version places a Beta-Binomial prior on each individual's expected number of lifetime infections and, unlike the prior used in the main text, assumes that an individual's probability of infection in a given time period is unrelated to any other individual:

$$P(\mathbf{Z}) = \prod_{i=1}^{n} P(\mathbf{Z_i}) = \prod_{i=1}^{n} \int_0^1 P(\mathbf{Z_i}|\mathbf{\Lambda_i} = \lambda)P(\mathbf{\Lambda_i} = \lambda)d\lambda = \prod_{i=1}^{n} \frac{B(\alpha + k_i, \beta + m_i + k_i)}{B(\alpha, \beta)}$$

Where k_i is the total number of infections experienced by individual i, and m_i is the number of time periods individual i could be infected. This prior is suitable for fitting antibody landscapes to each individual's antibody profile, but generates less interpretable attack rate estimates [35]. Model fitting was exactly the same as described in the main text, but 6 MCMC chains were run for only 12,000,000 iterations with the first 2,000,000 discarded as burn-in. Each strain-specific offset term was estimated under a multi-level model, assuming that each offset term in $\boldsymbol{\chi}$ was drawn from the same normal distribution with mean 0 and unknown standard deviation.

Fig S22 shows that the model-predicted titres provided a better fit to the data with the added offset terms. We used the maximum posterior probability estimates for each χ as a fixed parameter for the model used in the main text (Table S3). Note that the model without the offset terms produced very similar antibody kinetics parameter estimates (Table S4), as well as age- and location-specific attack rate patterns, to those shown in the main text.

medRxiv preprint doi: https://doi.org/10.1101/2024.03.18.24304371; this version posted March 18, 2024. The copyright holder for this preprint (which was not certified by peer review) is the author/funder, who has granted medRxiv a license to display the preprint in perpetuity.
It is made available under a CC-BY 4.0 International license .

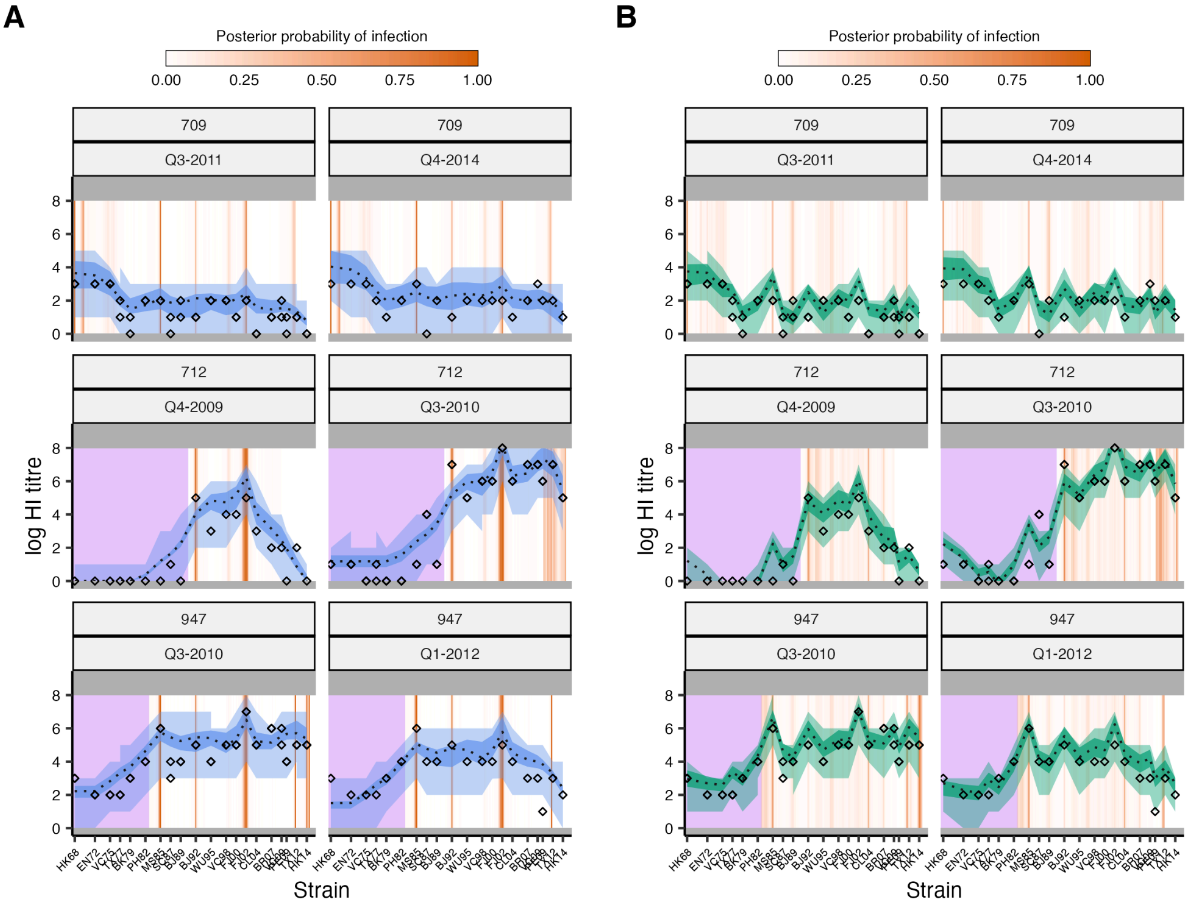

Figure S22: Comparison of estimated antibody landscapes from the same model fit with and without strain-specific measurement offsets. Rows represent individuals. Subplots show antibody titres based on serum samples taken at that time. X-axis represents a position along the antigenic summary path. Black diamonds show observed titres. Black line and blue or green shaded regions show posterior median and 95% credible intervals (CrI) on model-predicted latent titres (dark blue/green) and 95% prediction intervals (light blue/green). Orange bars show posterior probability of infection in that 3-month window. Grey rectangles denote the limit of detection of the HI assay. Purple rectangles show time periods prior to birth. (**A**) Model-predicted titres compared to observed HI titres at each sampling time for three randomly selected individuals, as in Fig S5, but from fitting the model without strain-specific measurement offsets. (**B**) as in (**A**), but with the estimated strain-specific measurement offsets included.

medRxiv preprint doi: https://doi.org/10.1101/2024.03.18.24304371; this version posted March 18, 2024. The copyright holder for this preprint (which was not certified by peer review) is the author/funder, who has granted medRxiv a license to display the preprint in perpetuity.
It is made available under a CC-BY 4.0 International license .

Table S3: Strain-specific measurement offset terms used in the main model fits. Values shown are maximum posterior probability estimates from a less flexible version of the model fit to the same data (described in Supplementary Text 1). Values shown to three significant figures.

Strain	Fixed value
A/HongKong/1968	1.216
A/England/1972	0.387
A/Victoria/1975	-0.643
A/Texas/1977	-0.476
A/Bangkok/1979	-1.392
A/Philippines/1982	-1.139
A/Mississippi/1985	0.139
A/Sichuan/1987	-1.941
A/Beijing/1989	-2.302
A/Beijing/1992	-0.386
A/Wuhan/1995	-1.847
A/Victoria/1998	-0.387
A/Fujian/2000	-0.573
A/Fujian/2002	0.746
A/California/2004	-0.515
A/Brisbane/2007	-0.655
A/Victoria/2009	0.028
A/Perth/2009	-0.984
A/Texas/2012	0.217
A/HongKong/2014	0.027

medRxiv preprint doi: https://doi.org/10.1101/2024.03.18.24304371; this version posted March 18, 2024. The copyright holder for this preprint (which was not certified by peer review) is the author/funder, who has granted medRxiv a license to display the preprint in perpetuity.
It is made available under a CC-BY 4.0 International license .

Table S4: Estimated antibody kinetics parameters under the model without strain-specific measurement offsets.

Parameter	Description	Estimate (posterior median; 95% credible intervals)
μ_l	Long-term boosting	1.54 (1.50-1.58)
μ_s	Short-term boosting	1.95 (1.82-2.07)
τ	Antigenic seniority term	0.0329 (0.0307-0.0357)
ω	Waning rate parameter for the short-term response (per 3-months)	0.872 (0.776-1.02)
σ_l	Long-term cross reactivity	0.0896 (0.0889-0.0898)
σ_s	Short-term cross reactivity	5.17e-05 (5.7e-06-0.000278)
ε	Standard deviation of observations	0.874 (0.868-0.881)

medRxiv preprint doi: https://doi.org/10.1101/2024.03.18.24304371; this version posted March 18, 2024. The copyright holder for this preprint (which was not certified by peer review) is the author/funder, who has granted medRxiv a license to display the preprint in perpetuity.
It is made available under a CC-BY 4.0 International license .

Supplementary Text 2

2. Scenario analyses using simulation-recovery experiments

2.1 Overview

The full *serosolver* model framework is complex, with many components and parameters to be estimated or fixed. A concern is therefore the identifiability of the estimated model parameters and the potential biases introduced by inappropriate assumptions for the fixed components. To assess the ability of the *serosolver* framework to accurately recover infection histories and antibody kinetics parameters, we simulated a dataset matching the dimensions of the Fluscape survey and performed extensive simulation-recovery experiments testing the robustness of the framework to each step of our inference pipeline. The aim of this analysis was to test how simplifying assumptions and model misspecification might bias our estimates relative to the known ground truth.

2.2 Simulation settings

We generated a simulated serosurvey of 1000 individuals of random ages uniformly sampled between 5 and 75 years. We simulated attack rates in 3-month time windows over a 46-year time period loosely based on estimates from the real data – per-quarter attack rates were randomly drawn from a log-normal distribution with mean 0.0375 and standard deviation 1.5 (on the natural scale). We assumed an attack rate of 0.6 in the first time period to represent the pandemic wave of H3N2 in 1968. Random infection histories for each individual were then simulated for each 3-month time period as Bernoulli trials with infection probability given by the simulated attack rates. Based on these infection histories, we then simulated each individual's latent antibody kinetics using assumed model parameter values (Table S5). To reflect the Fluscape serosurvey, we assumed that each individual had two serum samples taken at random times from the last 24 time periods of the simulation, with two titre measurements at each sample against 24 strains uniformly distributed across the simulation period. A crucial addition to the simulation is the inclusion of strain-specific measurement offsets – measurements against each strain were assumed to be shifted relative to the true titre, where these measurement shifts were normally distributed with mean 0 and standard deviation 0.5. We generated random measurement shifts for each measured strain drawn from this distribution. Finally, we used the same antigenic map as described in the main text Materials and Methods.

medRxiv preprint doi: https://doi.org/10.1101/2024.03.18.24304371; this version posted March 18, 2024. The copyright holder for this preprint (which was not certified by peer review) is the author/funder, who has granted medRxiv a license to display the preprint in perpetuity.
It is made available under a CC-BY 4.0 International license .

Table S5: Description of antibody kinetics parameter values used for the simulation. Uniform priors were used for all parameters.

Parameter	Description	Value	Prior lower bound	Prior upper bound	Estimate using the correctly specified model (posterior median; 95% credible intervals)
μ_l	Long-term boosting	1.80	0	8	1.74 (1.71-1.77)
μ_s	Short-term boosting	2.70	0	8	2.74 (2.53-2.91)
τ	Antigenic seniority term	0.05	0	1	0.0511 (0.0486-0.0541)
ω	Waning rate parameter for the short-term response (per 3-months)	0.20	0	1	0.216 (0.188-0.231)
σ_l	Long-term cross reactivity	0.10	0	1	0.101 (0.100-0.102)
σ_s	Short-term cross reactivity	0.03	0	1	0.0310 (0.0293-0.0329)
ε	Standard deviation of observations	1.00	0	25	1.00 (0.995-1.01)

medRxiv preprint doi: https://doi.org/10.1101/2024.03.18.24304371; this version posted March 18, 2024. The copyright holder for this preprint (which was not certified by peer review) is the author/funder, who has granted medRxiv a license to display the preprint in perpetuity.
It is made available under a CC-BY 4.0 International license .

2.3 Scenario analyses

2.3.1 Re-estimating the strain-specific measurement offsets

The first step of the inference pipeline is to estimate the strain-specific measurement offset terms, where some strains have systematically higher or lower titres measured in the HI assay after accounting for differences in time-since-infection, exposure history and random measurement error. Following the same approach as outlined in Supplementary Text 1, we fitted the *serosolver* model to the simulated data to re-estimate the strain-specific measurement offsets. The recovered parameter estimates were close to those used to simulate the data, though there were some systematic biases (Fig S23). Most notably the total number of infections was overestimated, the antibody boosting parameters were underestimated and the cross-reactivity parameters were overestimated. The estimated measurement offset parameters were also biased but all in the right direction (Fig S24). Relative to the prior ranges (uniform between -3 and 3), the parameter estimates were close to their true values, giving us confidence that our estimates for the measurement offset terms were still informative and could thus be used as fixed values for subsequent model fits.

medRxiv preprint doi: https://doi.org/10.1101/2024.03.18.24304371; this version posted March 18, 2024. The copyright holder for this preprint (which was not certified by peer review) is the author/funder, who has granted medRxiv a license to display the preprint in perpetuity.
It is made available under a CC-BY 4.0 International license .

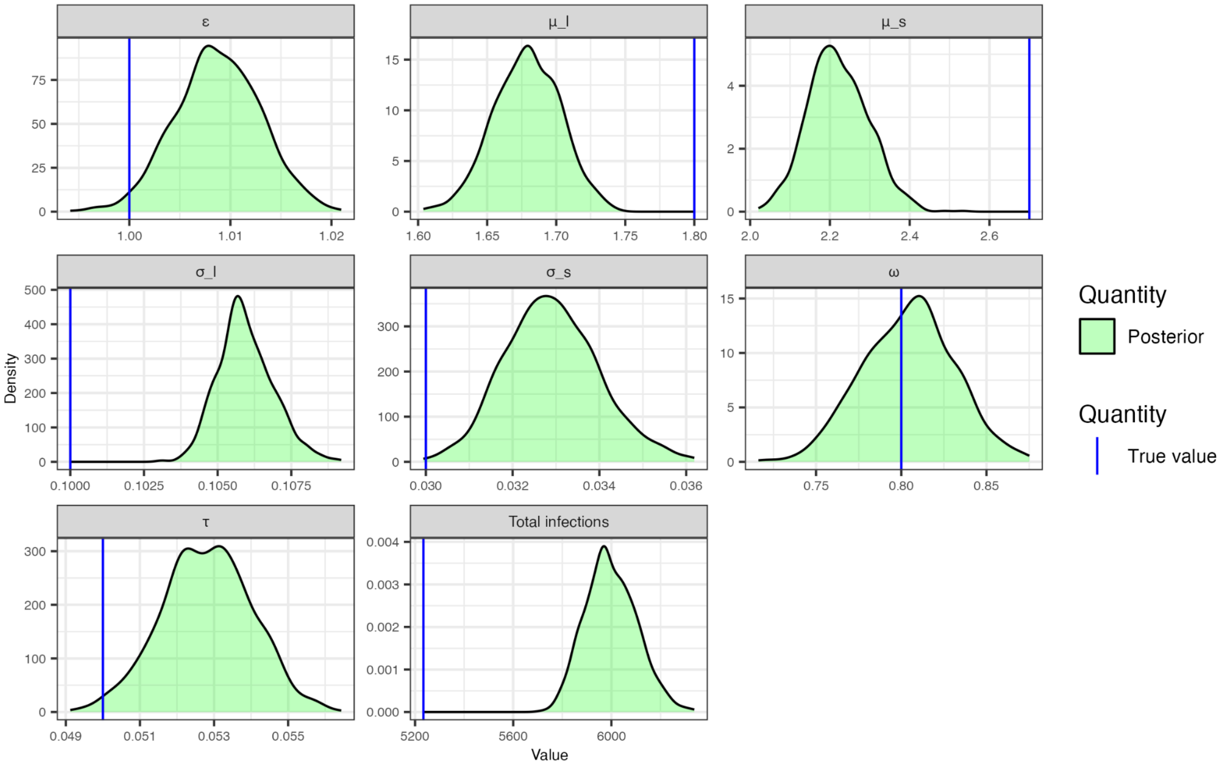

Figure S23: Estimated posterior distributions for antibody kinetics parameters (green shaded region) using the fitted model described in Supplementary Text 1 compared to true values used in the simulation (blue line).

medRxiv preprint doi: https://doi.org/10.1101/2024.03.18.24304371; this version posted March 18, 2024. The copyright holder for this preprint (which was not certified by peer review) is the author/funder, who has granted medRxiv a license to display the preprint in perpetuity.
It is made available under a CC-BY 4.0 International license .

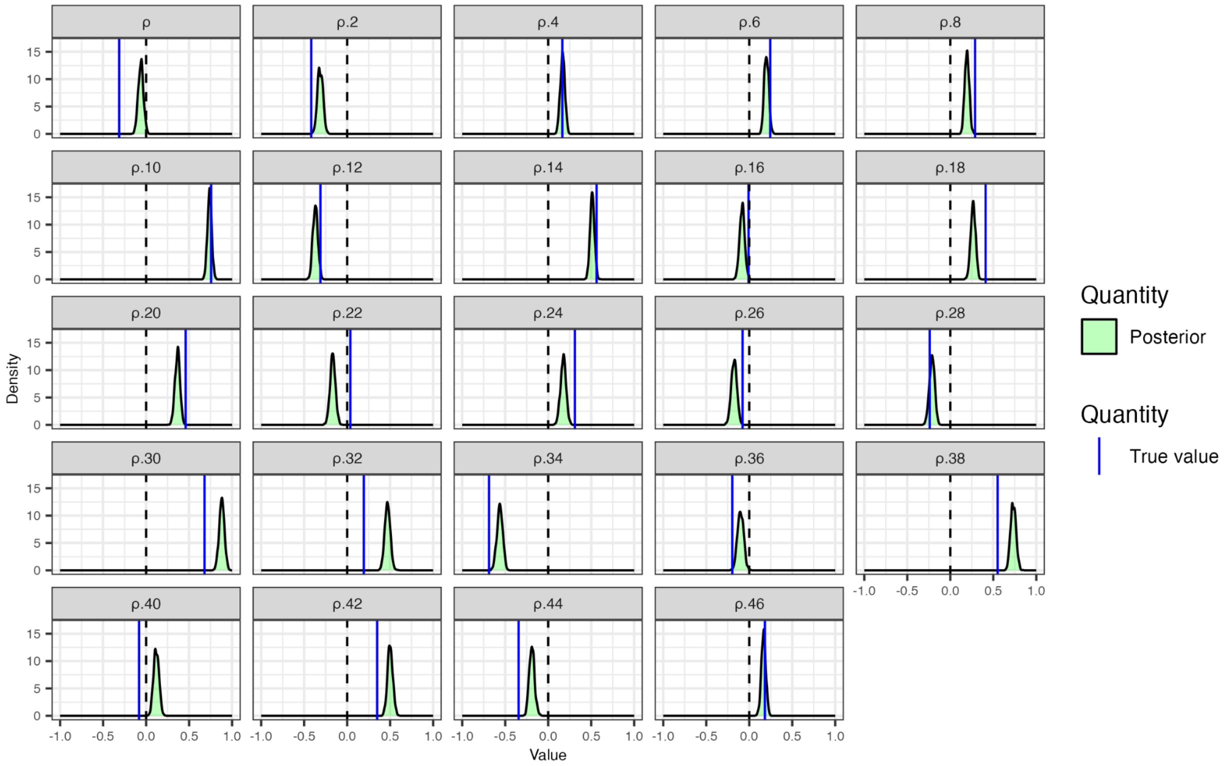

Figure S24: Estimated posterior distributions for strain-specific measurement offsets (green shaded region) using the fitted model described in Supplementary Text 1 compared to true values used in the simulation (blue line).

medRxiv preprint doi: https://doi.org/10.1101/2024.03.18.24304371; this version posted March 18, 2024. The copyright holder for this preprint (which was not certified by peer review) is the author/funder, who has granted medRxiv a license to display the preprint in perpetuity.
It is made available under a CC-BY 4.0 International license .

2.3.2 Correctly specified model

Using the re-estimated strain-specific measurement offsets estimated from Section 2.3.1, we fit the *serosolver* model to the simulated data under the assumption that the model was correctly specified. This provides a sense check that the inference framework is able to accurately recover the antibody kinetics parameter estimates, attack rates and infection histories when the true generative model is known. 5 MCMC chains were run for 1,000,000 iterations with a 200,000 iteration burn in period. The model accurately re-estimated the true simulation parameter values (Table S5), though with a slight underestimation of the long-term boosting parameter (Fig S25).

medRxiv preprint doi: https://doi.org/10.1101/2024.03.18.24304371; this version posted March 18, 2024. The copyright holder for this preprint (which was not certified by peer review) is the author/funder, who has granted medRxiv a license to display the preprint in perpetuity.
It is made available under a CC-BY 4.0 International license .

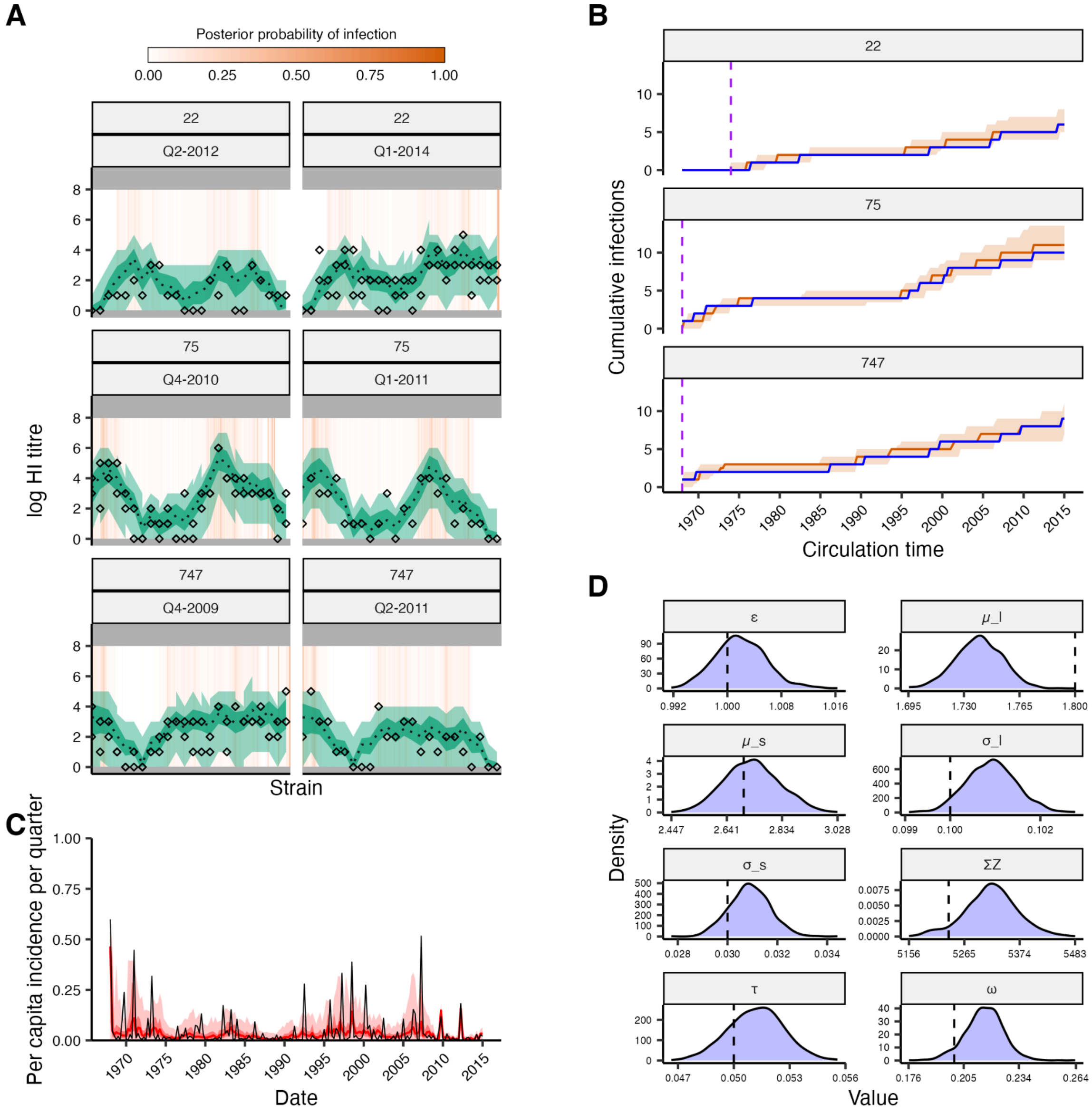

Figure S25: Assessment of model fitting accuracy based on simulated data. Results shown are from fitting the full model to simulated infection histories and antibody titres with known parameters. (**A**) Model-predicted titres compared to observed HI titres at each sampling time for three individuals. (**B**) Posterior median and 95% credible intervals (CrI) for the cumulative number of infections over time from birth (orange). Blue solid line shows the true, known cumulative number of infections. Purple dashed line shows the time of birth. (**C**) Posterior estimated per-capita per-3-month attack rates. Red line and shaded region shows posterior median and 95% CrI. Grey line shows the true values used for the simulation. (**D**) Shaded regions show posterior distributions of estimated antibody kinetics parameters. Dashed lines show the true value used for simulation. Note the x-axis range is small relative to the prior ranges in Table S4.

medRxiv preprint doi: https://doi.org/10.1101/2024.03.18.24304371; this version posted March 18, 2024. The copyright holder for this preprint (which was not certified by peer review) is the author/funder, who has granted medRxiv a license to display the preprint in perpetuity.
It is made available under a CC-BY 4.0 International license .

2.3.3 Fitting the model ignoring strain-specific measurement offsets

To demonstrate the importance of considering the strain-specific measurement offsets, we compare the model estimates from Section 2.3.2 to results from fitting the model without accounting for the strain-specific measurement offsets, matching the workflow of the main text model. Both versions of the model were able to accurately re-estimate individual-level infection histories and fit to the antibody data well (Fig S25A/B & Fig S26A/B). However, the version without the measurement offset term led to biased attack rate estimates in some time periods (Fig S26C, e.g., 1969, 1975-1980, 1995-2000, 2005-2010). Furthermore, the version without the measurement offset terms led to greater bias in the estimated antibody kinetics parameters, particularly the observation error parameter, ε (Fig S26D). Overall, these results demonstrate that attempting to account for strain-specific measurement biases, even if incompletely, leads to parameter estimates which are closer to the true values than if these biases are ignored.

medRxiv preprint doi: https://doi.org/10.1101/2024.03.18.24304371; this version posted March 18, 2024. The copyright holder for this preprint (which was not certified by peer review) is the author/funder, who has granted medRxiv a license to display the preprint in perpetuity.
It is made available under a CC-BY 4.0 International license .

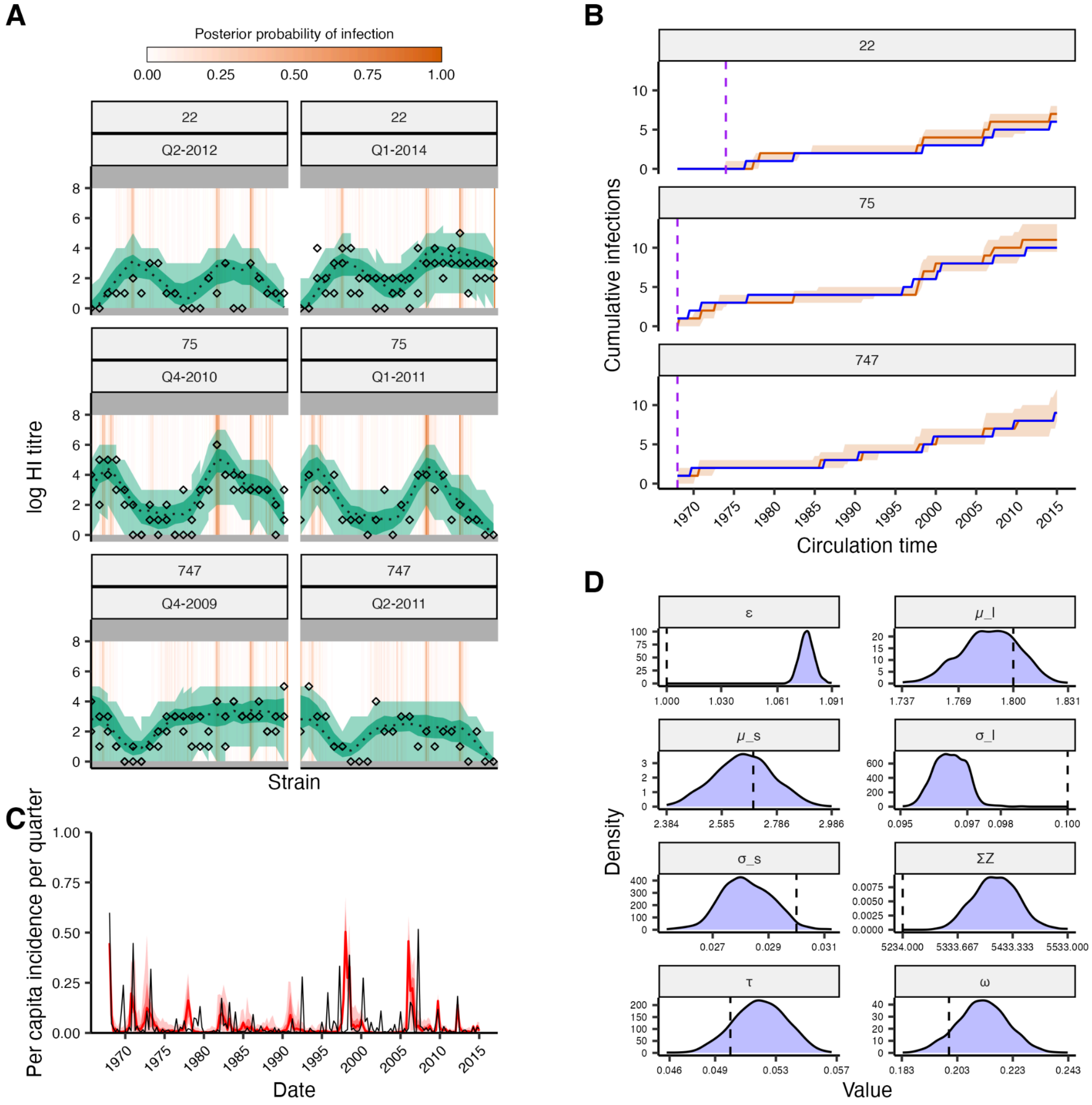

Figure S26: Assessment of model fitting accuracy based on simulated data when strain-specific measurement offsets are ignored. Results shown are from fitting the full model to simulated infection histories and antibody titres with known parameters where strain-specific measurement offsets are used in the simulation, but ignored in the fitted model. (**A**) Model-predicted titres compared to observed HI titres at each sampling time for three individuals. (**B**) Posterior median and 95% credible intervals (CrI) for the cumulative number of infections over time from birth (orange). Blue solid line shows the true, known cumulative number of infections. Purple dashed line shows the time of birth. (**C**) Posterior estimated per-capita per-3-month attack rates. Red line and shaded region shows posterior median and 95% CrI. Grey line shows the true values used for the simulation. (**D**) Shaded regions show posterior distributions of estimated antibody kinetics parameters. Dashed lines show the true value used for simulation. Note the x-axis range is small relative to the prior ranges in Table S5.

medRxiv preprint doi: https://doi.org/10.1101/2024.03.18.24304371; this version posted March 18, 2024. The copyright holder for this preprint (which was not certified by peer review) is the author/funder, who has granted medRxiv a license to display the preprint in perpetuity.
It is made available under a CC-BY 4.0 International license .

2.3.6 Misspecifying the antigenic map

We do not know the strains which each individual was potentially exposed to, only the most likely antigenic cluster circulating in each time period. Thus, an individual's infection history inferred using *serosolver* depends on the assumed strain an individual could be infected with in each time period, and how it contributes cross-reactive antibodies to their antibody profile. In theory, we might jointly estimate the antigenic map coordinates alongside the other model parameters, but at present this is computationally infeasible and thus we assume a fixed antigenic map for model fitting. As our estimates rely on the assumed position of each strain on the antigenic map, we performed sensitivity analyses to test how our estimates are affected by misspecifying the antigenic map.

First, we refit the model as described in Section 2.3.1 to the same simulated dataset, but instead of using the antigenic map used to simulate the data, we used an alternative antigenic map produced by Bedford et al for model fitting [90] (comparison in Fig S27). As with the antigenic map used in the main text, to generate a single antigenic position representing each strain we fitted a cubic smoothing spline through the antigenic coordinates of all strains on the map (here, smoothing parameter = 0.8). This scenario tests the assumption that the antigenic map used for fitting does not match the antigenic map underlying the data generating process. Parameter estimates, infection histories and attack rates were all largely accurate despite using the wrong antigenic map, though the timing of some elevated attack rates were slightly off (e.g., 1973, 1992, 2007). There were some small biases in the long-term boosting, cross-reactivity and observation error parameters (Fig S28). This suggests that although our quantitative estimates are affected by the assumed antigenic map, the overall trends are largely preserved.

medRxiv preprint doi: https://doi.org/10.1101/2024.03.18.24304371; this version posted March 18, 2024. The copyright holder for this preprint (which was not certified by peer review) is the author/funder, who has granted medRxiv a license to display the preprint in perpetuity.
It is made available under a CC-BY 4.0 International license .

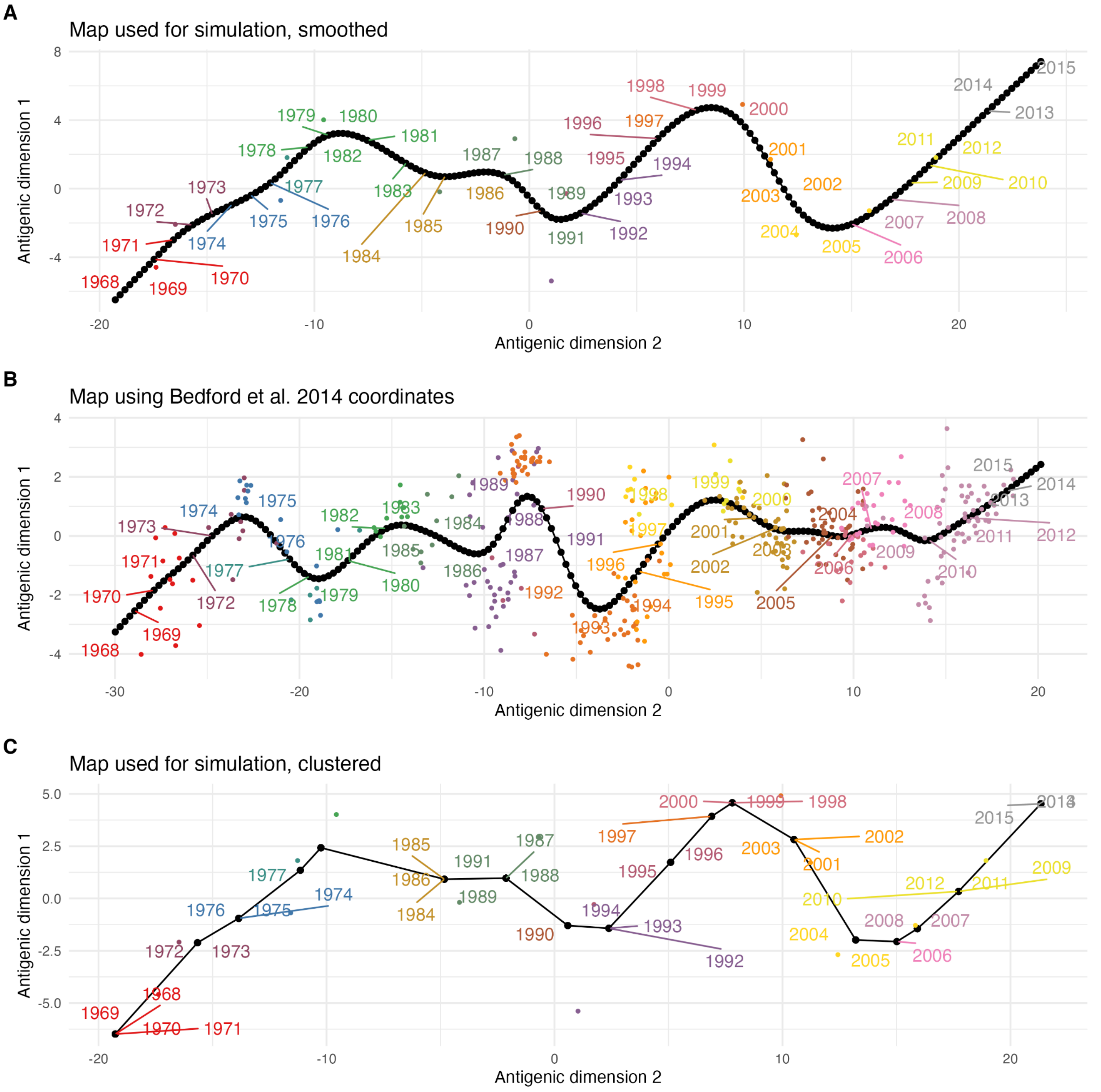

Figure S27: Antigenic maps used for the simulation (A) compared to the map produced using data from Bedford et al. (B), and the map which assumes a punctuated path through antigenic space (C). Axes show arbitrary antigenic dimensions. Coloured points show the position of individual strains – all labels of the same color correspond to the same antigenic cluster. Black line shows the fitted antigenic summary path for each map. Black dots show the antigenic position of the strain corresponding to each time period; (**A**) and (**B**) assume continuous evolution through antigenic space whereas (**C**) assumes that the same position is used for all strains within a cluster.

medRxiv preprint doi: https://doi.org/10.1101/2024.03.18.24304371; this version posted March 18, 2024. The copyright holder for this preprint
(which was not certified by peer review) is the author/funder, who has granted medRxiv a license to display the preprint in perpetuity.
It is made available under a CC-BY 4.0 International license .

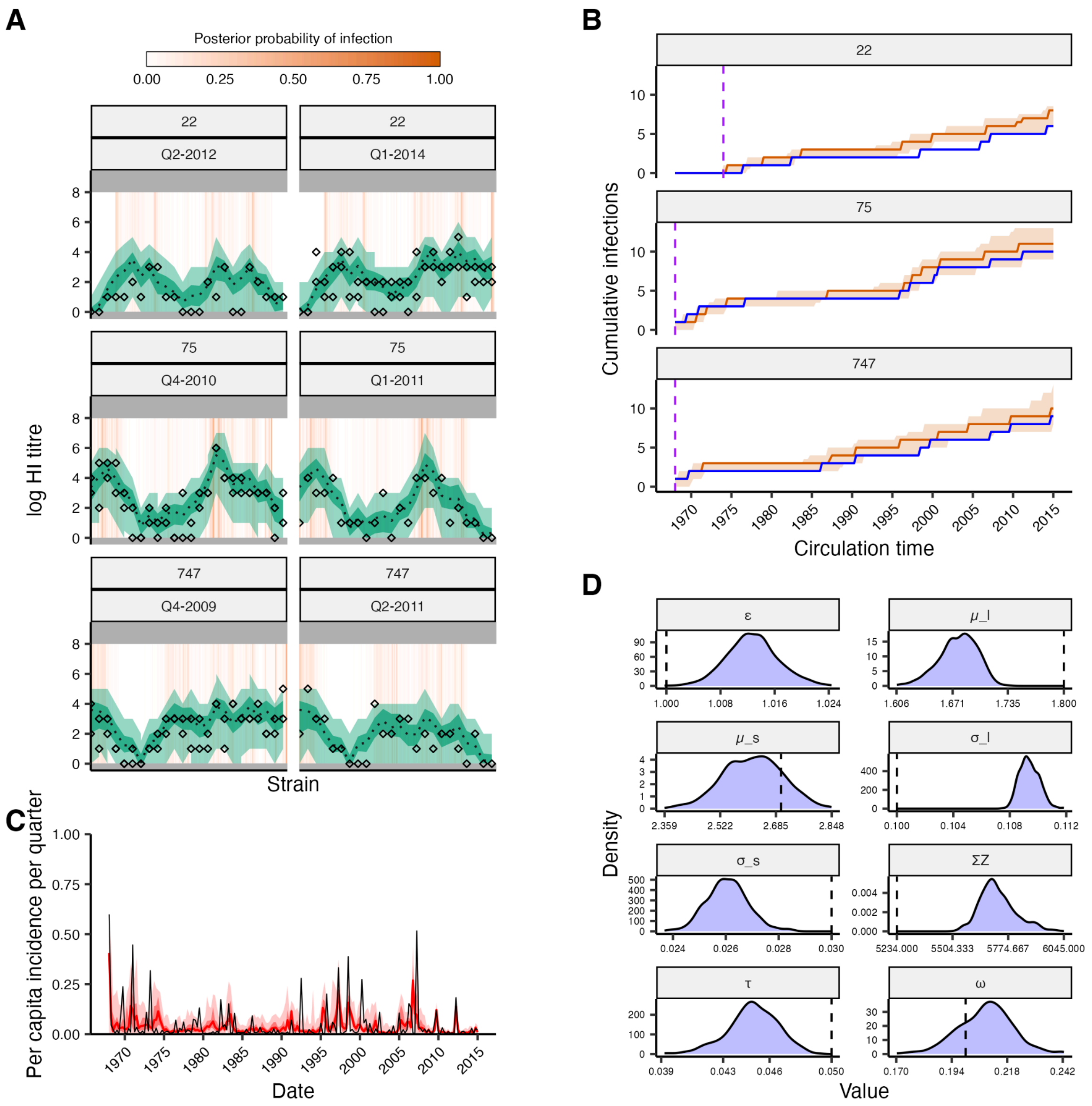

Fig S28: Assessment of model fitting accuracy when the antigenic map used for model fitting does not match the one used for simulation. Results shown are from fitting the full model to simulated infection histories and antibody titres with known parameters. (**A**) Model-predicted titres compared to observed HI titres at each sampling time for three individuals. (**B**) Posterior median and 95% credible intervals (CrI) for the cumulative number of infections over time from birth (orange). Blue solid line shows the true, known cumulative number of infections. Purple dashed line shows the time of birth. (**C**) Posterior estimated per-capita per-3-month attack rates. Red line and shaded region shows posterior median and 95% CrI. Grey line shows the true values used for the simulation. (**D**) Shaded regions show posterior distributions of estimated antibody kinetics parameters. Dashed lines show the true value used for simulation. Note the x-axis range is small relative to the prior ranges in Table S4.

medRxiv preprint doi: https://doi.org/10.1101/2024.03.18.24304371; this version posted March 18, 2024. The copyright holder for this preprint (which was not certified by peer review) is the author/funder, who has granted medRxiv a license to display the preprint in perpetuity.
It is made available under a CC-BY 4.0 International license .

Second, we generated an alternative simulated dataset using an antigenic map where antigenic evolution was assumed to follow punctuated jumps between clusters with all strains belonging to the same cluster having the same antigenic coordinates matching the first strain isolated from each cluster (Fig S27C), and then refit the model described in Section 2.3.1 using the smoothed antigenic map rather than the clustered map used for the simulation. This scenario tests the impact of our assumption that sequential strains follow a smooth and continuous path through antigenic space despite the true data generating process following punctuated jumps between clusters. We used the estimates from Du et al. to determine the time periods of cluster dominance – Du et al. predicted which A/H3N2 antigenic cluster likely circulated in China in each time period based on HA sequence data. For example, where Du et al. present a time range e.g., BE92 (1992-1995), we assumed that cluster circulated up to and not including the final time point in the range e.g., assume that BE92 circulated from January 1992 up to and including December 1994. We do not have cluster information after the PE09 cluster, and thus we assumed a new cluster emerged in 2013 (i.e., the PE09 cluster dominated for 4 years). Du et al. demonstrate that clusters are almost entirely dominant in the period in which they are circulating, though they rarely reach 100% frequency.

Parameter, infection history and attack rate estimates were mostly close to their true values despite misspecifying the antigenic map, with some differences in the estimated attack rates. Infection histories and model fits matched their true values well (Fig S29A&B), though the timing of elevated attack rates did not always align (Fig S29C). For example, the model missed the high attack rate in 1971, likely attributing these infections to the consecutive high attack rate periods from 1968. The high attack rates in 1992 and 2007 were also slightly misaligned, though the model was able to detect high incidence around those time periods. This misalignment is expected given how the antigenic map was misspecified. In the clustered map, we assumed that all strains in a cluster had the antigenic coordinates matching the first strain. However, in the smoothed map, each strain was instead assumed to be antigenically different from the previous one. Thus, when fitting with the smooth map, the model tends to attribute infections in a cluster to the first strain, as its antigenic coordinates are the closest to their true values used in the simulation. This tends to shift attack rates in those cluster periods earlier. Reassuringly, the model was still able to accurately recover the total number of infections in the simulation.

Some of the antibody kinetics parameters were slightly biased, with notable overestimation of the observation error parameter and waning rate, and slight underestimation of the long-term

medRxiv preprint doi: https://doi.org/10.1101/2024.03.18.24304371; this version posted March 18, 2024. The copyright holder for this preprint (which was not certified by peer review) is the author/funder, who has granted medRxiv a license to display the preprint in perpetuity.
It is made available under a CC-BY 4.0 International license .

cross-reactivity parameter (Fig S29D). Bias in the waning rate parameter estimates is unsurprising given that attack rates were estimated to be slightly later than the truth for recent time periods which provide the model with information on these short-term kinetics. If infections are estimated later than their true timing, then a higher waning rate is required to reach the same low titres in a shorter time since infection. Bias in the observation error parameter is also unsurprising, as bias from model misspecification in the measurement offset term estimation stage (Section 2.3.1) becomes compounded when fitting the full model.

medRxiv preprint doi: https://doi.org/10.1101/2024.03.18.24304371; this version posted March 18, 2024. The copyright holder for this preprint (which was not certified by peer review) is the author/funder, who has granted medRxiv a license to display the preprint in perpetuity.
It is made available under a CC-BY 4.0 International license .

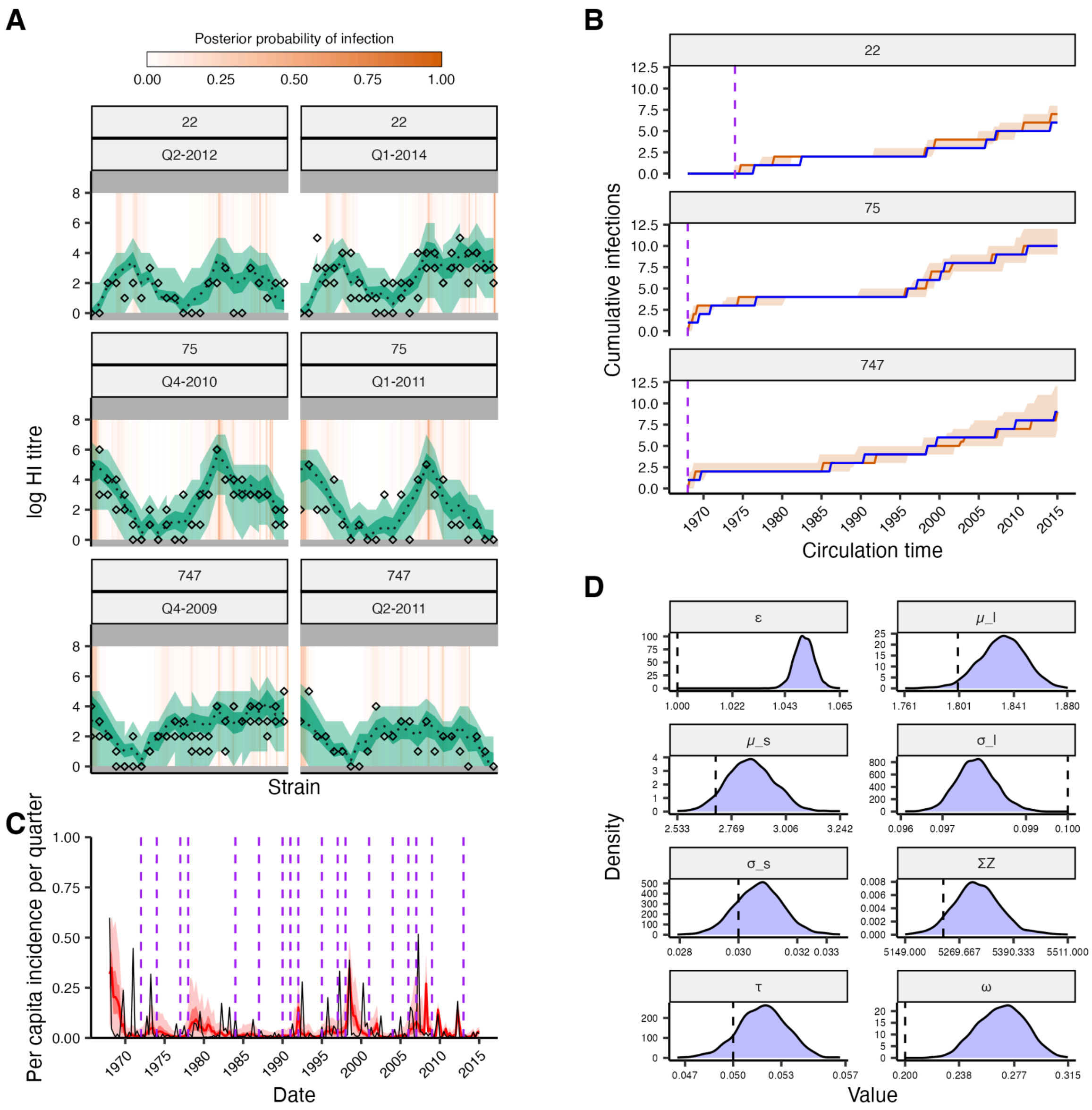

Fig S29: Assessment of model fitting accuracy based when fitting a model with a smoothed antigenic map to data simulated using a punctuated antigenic map. (**A**) Model-predicted titres compared to observed HI titres at each sampling time for three individuals. (**B**) Posterior median and 95% credible intervals (CrI) for the cumulative number of infections over time from birth (orange). Blue solid line shows the true, known cumulative number of infections. Purple dashed line shows the time of birth. (**C**) Posterior estimated per-capita per-3-month attack rates. Red line and shaded region shows posterior median and 95% CrI. Grey line shows the true values used for the simulation. Purple dashed lines show cluster transitions. (**D**) Shaded regions show posterior distributions of estimated antibody kinetics parameters. Dashed lines show the true value used for simulation. Note the x-axis range is small relative to the prior ranges in Table S5.

medRxiv preprint doi: https://doi.org/10.1101/2024.03.18.24304371; this version posted March 18, 2024. The copyright holder for this preprint (which was not certified by peer review) is the author/funder, who has granted medRxiv a license to display the preprint in perpetuity.
It is made available under a CC-BY 4.0 International license .

<< Separate file >>

Supplementary Material 3: Distribution of quarterly attack rates by location over time. Each colored point shows the inferred attack rate in each of the 40 locations, with size and shading reflecting the posterior median attack rate. Underlying the plot is a map of the study area, with each grid cell shaded by its log10 population density.
